# Stereoselective Polyglucuronic Acid Assembly via the Iron-Catalyzed Stereospecific Glycosylation with Glycal Epoxides

**DOI:** 10.1021/acs.orglett.6c03166

**Published:** 2026-09-23

**Authors:** Xiao-Wen Zhang, Zixiang Jiang, Hao Xu

**Affiliations:** Department of Chemistry, 8244 Brandeis University, 415 South Street, Waltham, Massachusetts 02453, United States

## Abstract

Polyglucuronic acids (PGAs) are directly involved in essential biological processes, but catalytic methods for stereoselective PGA synthesis are underdeveloped because glucuronic acid (GlcA) donors are intrinsically difficult to activate. We report herein a convergent and stereoselective PGA assembly method via iron-catalyzed iterative and stereospecific glycosylation with glycal epoxides. This method is effective in the assembly of an array of GlcA-β-1,4-GlcA linkages in complex glycans and readily provides PGA octasaccharides in high stereoselectivity.

## Graphical abstract

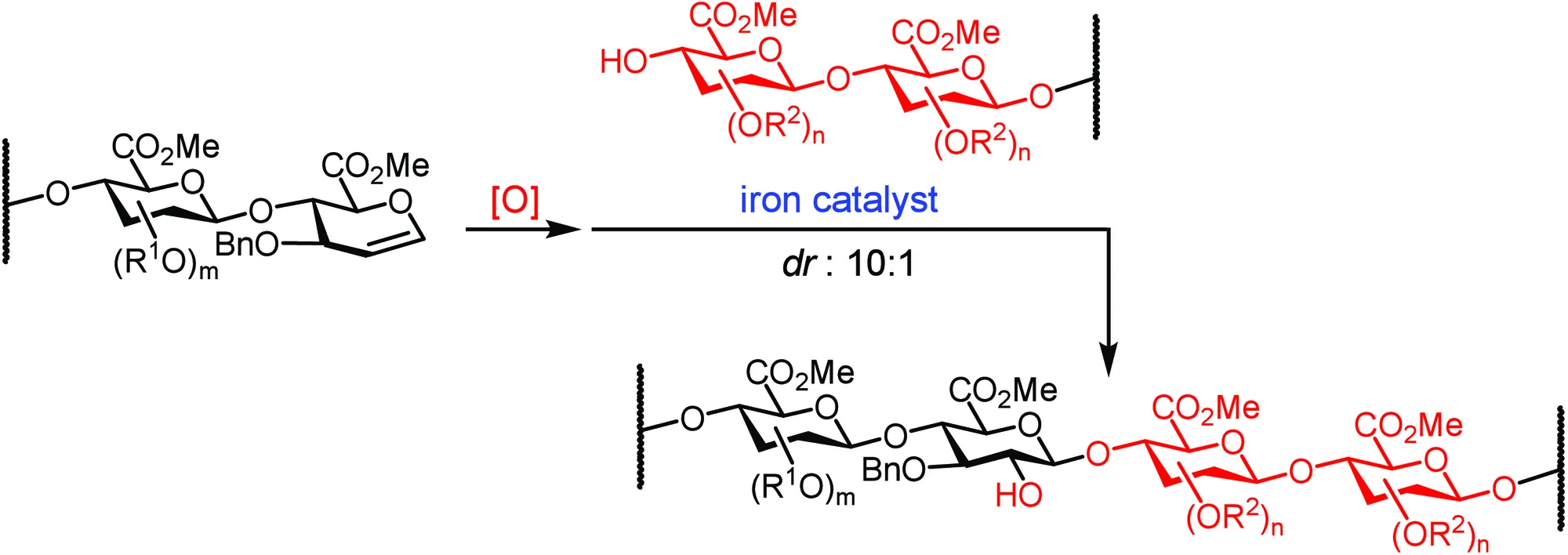

Polysaccharides participate in a variety of essential biological processes,^1^ and polyuronic acids play critical roles in maintaining cellular structures, enabling ion binding, and facilitating molecular recognition.^2,3^ While synthetic approaches to poly-β-(1–4)-d-mannuronic acid (ManA) and α-l-guluronic acid (GulA) have been established,^4–8^ methods for rapid and stereoselective synthesis of poly-β-(1–4)-d-glucuronic acid (GlcA) remain underdeveloped.^9^ This is presumably due to the intrinsically low reactivity of electron-deficient GlcA donors, which make them difficult to activate with a catalyst.^10–14^ Furthermore, the nucleophilicity of the C4-OH group of a GlcA acceptor is attenuated by the strongly electron-withdrawing uronic ester moiety. As exceptions, Seeberger recently reported an automated synthetic approach to polyglucuronic acids (PGAs) that leverages iterative glycosylation with glycosyl phosphates (Figure 1A).^15^ This effective approach extends the backbone one sugar at a time. Zhu also developed a convergent PGA assembly method that efficiently provides PGAs up to 32-mer (Figure 1B).^16^ Therefore, new convergent and robust approaches for stereoselective PGA assembly are still highly desirable.

**Figure 1:**
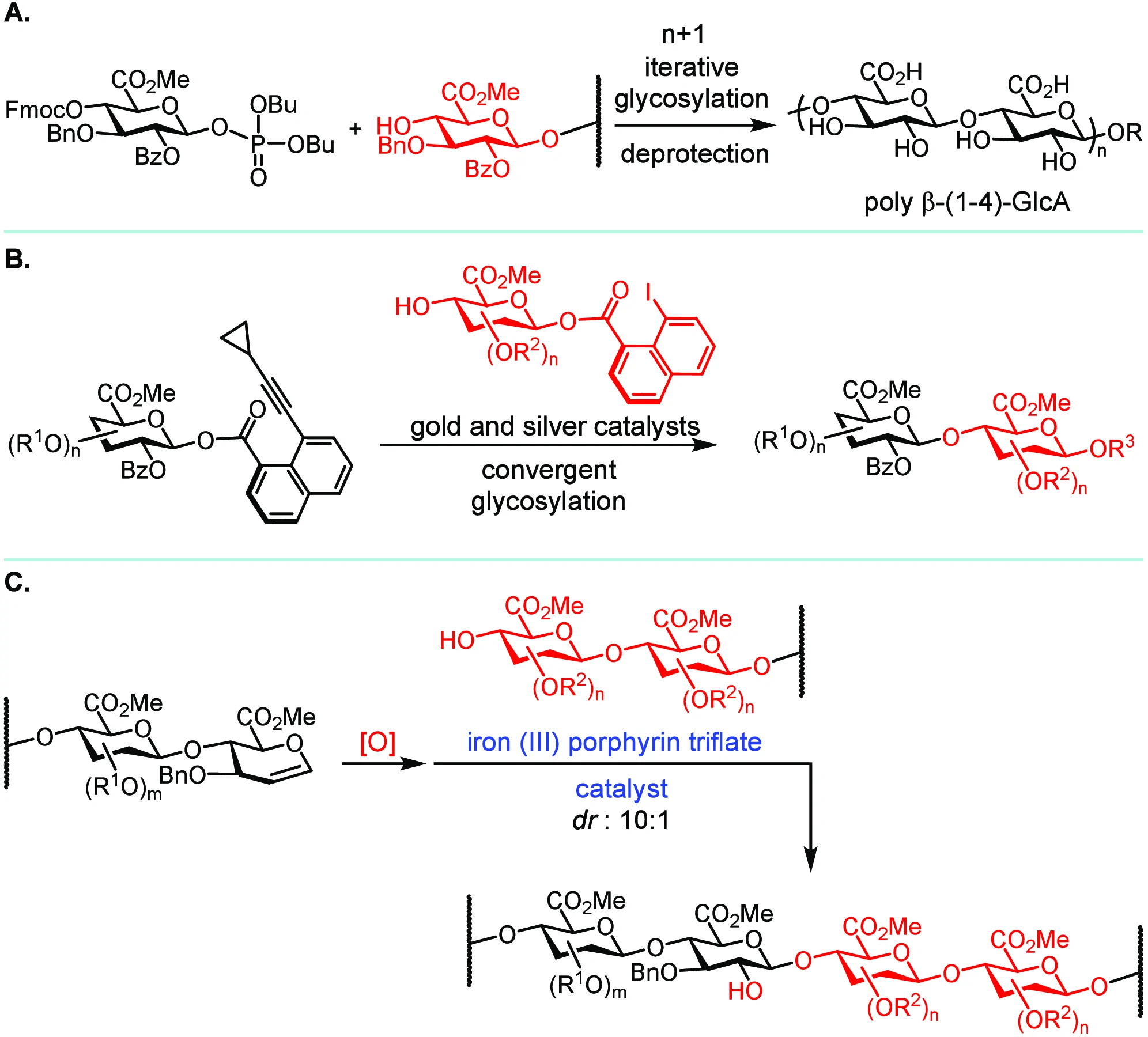
(A) Polyglucuronic acid (PGA) synthesis via iterative glycosylation with glycosyl phosphates. (B) PGA synthesis via gold-catalyzed convergent glycosylation. (C) Stereoselective PGA assembly via the iron-catalyzed stereospecific glycosylation with glycal epoxides (this work).

In principle, iterative and stereospecific glycosylation with glucuronic ester epoxides could readily afford PGAs. However, most existing stereospecific methods for glucuronic ester epoxide activation are incompatible with sugar acceptors, especially hindered secondary sugar acceptors.^17–19^ Recently, Jacobsen^20^ and our group^21–23^ have developed metal-catalyzed, stereospecific methods to activate glucuronic ester epoxides toward a variety of nucleophiles. We discovered that iron­(III) porphyrin triflate catalysts^24^ induce highly stereospecific glycosylation of an array of hindered, secondary sugar acceptors with various glycal epoxides, which was previously challenging.^25–31^

Building upon this discovery, we envisioned that executing this iron-catalyzed stereospecific glycosylation between a glucuronic ester epoxide module and a C4-OH glucuronic ester acceptor module in an iterative and convergent manner would rapidly assemble PGAs (Figure 1C). Herein, we report the discovery of iron-catalyzed stereospecific and iterative glycosylation with these two challenging modules for stereoselective PGA synthesis.

We selected glucuronals **2** and **3** as the building blocks for catalyst discovery (Figure 2). Epoxidation of a 4-O-silylated glucuronal **2** in a biphasic reaction medium with Oxone^32^ quantitatively afforded glucuronic ester α-epoxide **S1** (Figure S1; dr >20:1; ^3^ *J*_H1–H2_ = 2.4 Hz),^33^ which was azeotropically dried and used directly. Although a variety of hindered C4-OH acceptors are excellent substrates for this iron-catalyzed stereospecific glycosylation,^21^ C4-OH glucuronal acceptor **3** is a particularly difficult substrate. Extensive exploration of catalysts and other reaction conditions revealed that readily available, hemin-derived iron catalyst **1a** ^21^ (7 mol %) is optimal in promoting the glycosylation of **3** with the glucuronic ester epoxide derived from **2** at 0 °C, affording **4a**/**4b** in good combined yield on a multigram scale (entry 1, 9.5 mmol scale, 74% yield, dr 4.6:1; for **4a**, ^3^ *J*_H1–H2_ = 7.6 Hz and ^1^ *J* ^13^_C1–H1_ = 159.2 Hz; for **4b**, ^3^ *J*_H1–H2_ = 3.4 Hz and ^1^ *J* ^13^_C1–H1_ = 171.3 Hz). Fortunately, acetylation of **4a**/**4b** furnished **5a** and **5b**, which can be readily separated with a flash column. There are several noteworthy observations. First, glycosylation under cryogenic conditions (at −20 or −40 °C) does not improve the dr (entry 2), but it does induce further glycosylation of the newly generated C2-OH group in **4a**/**4b** with the glucuronic ester epoxide, thereby decreasing the yield of **4a**/**4b** (Figure 2, entry 2). Next, hemin-derived catalysts^21^ **1b** and **1c** with more coordinating sulfonate counteranions surprisingly led to decreased dr values (entries 3 and 4, respectively; dr from 1.6:1 to 2.5:1). We also observed that the use of TMSOTf induced significant glycal epoxide decomposition (entry 5, dr 2.4:1) and that AgOTf resulted in evidently lower reactivity and dr (entry 6, <40% conversion, dr 2.6:1). Other synthetically less accessible iron porphyrin triflate catalysts, including **1d**/**1e**,^21^ were as effective as **1a** (entry 7).

**Figure 2:**
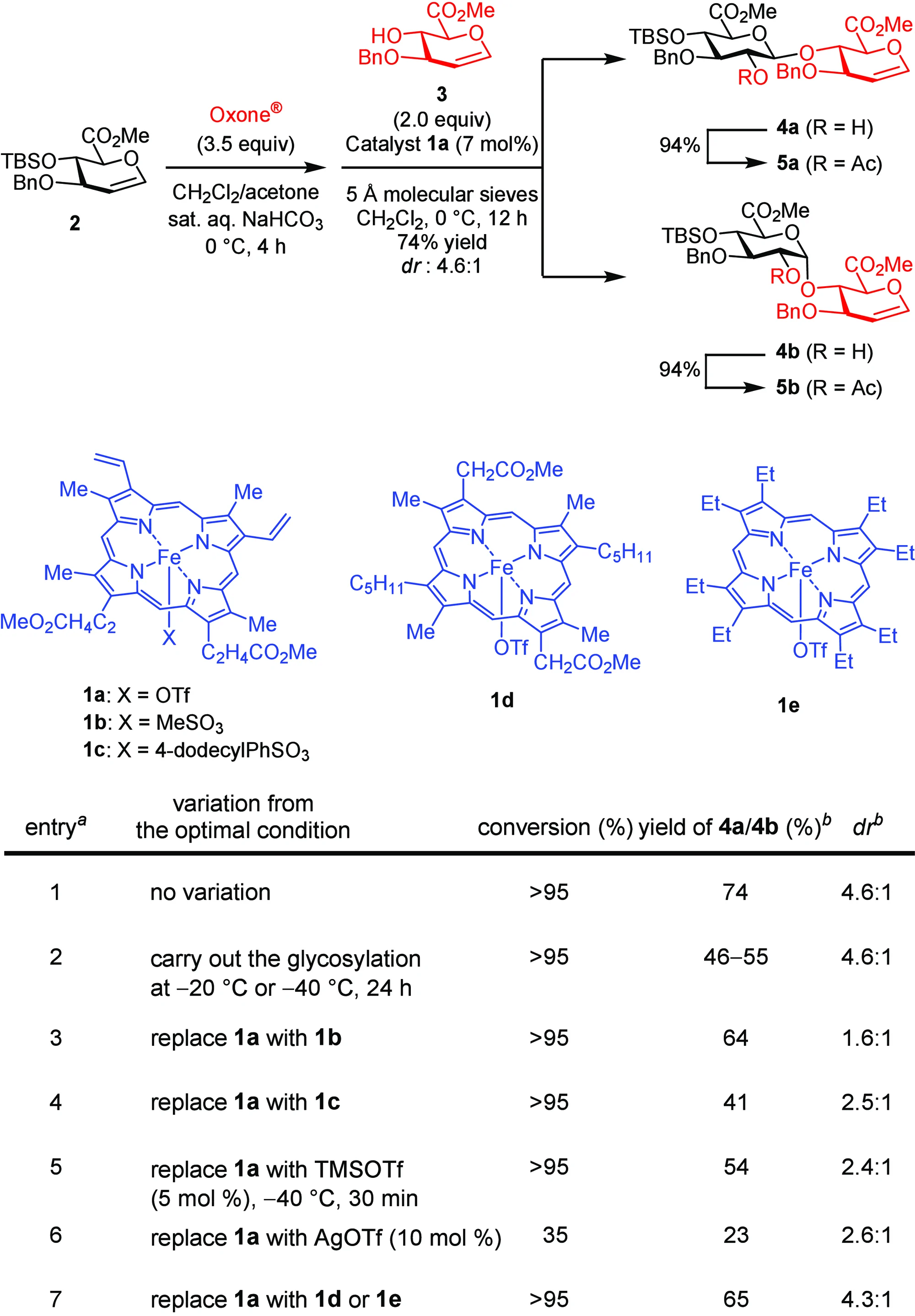
Catalyst discovery for the iron­(III) porphyrin sulfonate-catalyzed glycosylation of glucuronals with glucuronic ester epoxides. ^*a*^Epoxidation was carried out in a biphasic reaction medium with Oxone and acetone. The glycal epoxide was dried azeotropically with toluene, assayed by ^1^H NMR (dr >20:1), and then directly used. The glycosylation was carried out at 0 °C in CH_2_Cl_2_. Acetylation of **4a**/**4b** was achieved using Ac_2_O/pyridine to afford **5a** and **5b**. ^*b*^Isolated yield. The dr was determined by ^1^H NMR analysis. Iron­(III) porphyrin catalysts **1a**–**1e** were formed in situ from the corresponding iron porphyrin chloride catalysts (10 mol %) and AgOTf (7 mol %) to prevent the formation of an excess amount of AgOTf.

To evaluate the potential of this method for GlcA-β-1,4-GlcA assembly in complex substrates, we explored an array of synthetically valuable, GlcA-based glycosyl donors and acceptors (Figure 3). We observed that a 3-O-silylated glucuronal epoxide is an excellent donor for both C4-OH glucuronal and GlcA acceptors (Figure 3, products **6** and **7**, dr from 10:1 to >20:1). To our delight, iron catalyst **1a** promotes entirely stereospecific glycosylation with the epoxides generated from disaccharides **5a** (GlcA-β-1,4-glucuronal) and **5b** (GlcA-α-1,4-glucuronal), affording triglucuronic esters in good yields (products **8** and **9**, dr >20:1). Notably, several other readily available, disaccharide-based GlcA donors, including the epoxides derived from GlcN-α-1,4-GlcA, GlcN-β-1,4-GlcA, and GalN-β-1,4-GlcA, are excellent substrates for this iron-catalyzed glycosylation, furnishing single diastereomeric trisaccharides (products **10**–**13**, dr >20:1). The observed high β-selectivity is presumably due to the stereospecific glycosylation with α-glycal epoxides obtained in high stereoselectivity. As we previously reported, this method is effective for highly stereospecific glycosylation for GlcA epoxide activation toward nitrogen-containing glycosyl acceptors.^22^

**Figure 3:**
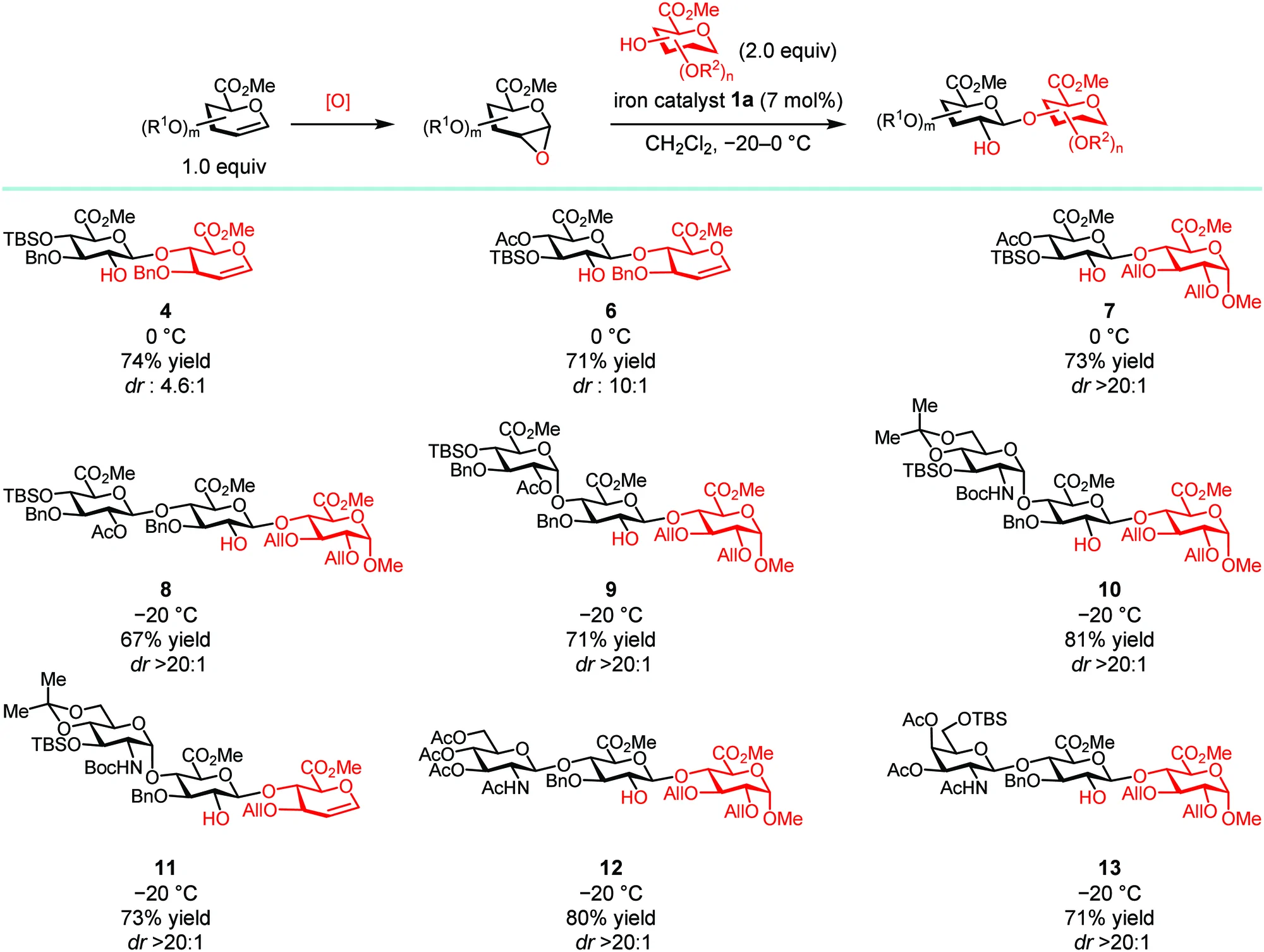
Substrate scope for GlcA-β-1,4-GlcA assembly via iron-catalyzed stereospecific glycosylation with glycal epoxides. All yields are isolated yields. See the Supporting Information for experimental details.

With four disaccharide modules, **5a**, **5b**, **14a**, and **14b** (through desiylation using TBAF/AcOH, 90% yield) in hand, we were ready to explore the iterative glycosylation for PGA synthesis (Scheme 1a). Epoxidation of GlcA-β-1,4-glucuronal **5a** using Oxone almost quantitatively afforded glucuronic ester α-epoxide **S9** (Figure S4; dr >20:1; ^3^ *J*_H1–H2_ = 2.4 Hz). We discovered that iron catalyst **1a** induces highly stereospecific glycosylation of the C4-OH group from a GlcA-β-1,4-glucuronal module **14a** with this epoxide, affording tetrasaccharide **15** in good yield (65% yield, dr 10:1; for **15**, ^3^ *J*_H1–H2_ = 7.7 Hz and ^1^ *J* ^13^_C1–H1_ = 162.4 Hz).

**Scheme 1:**
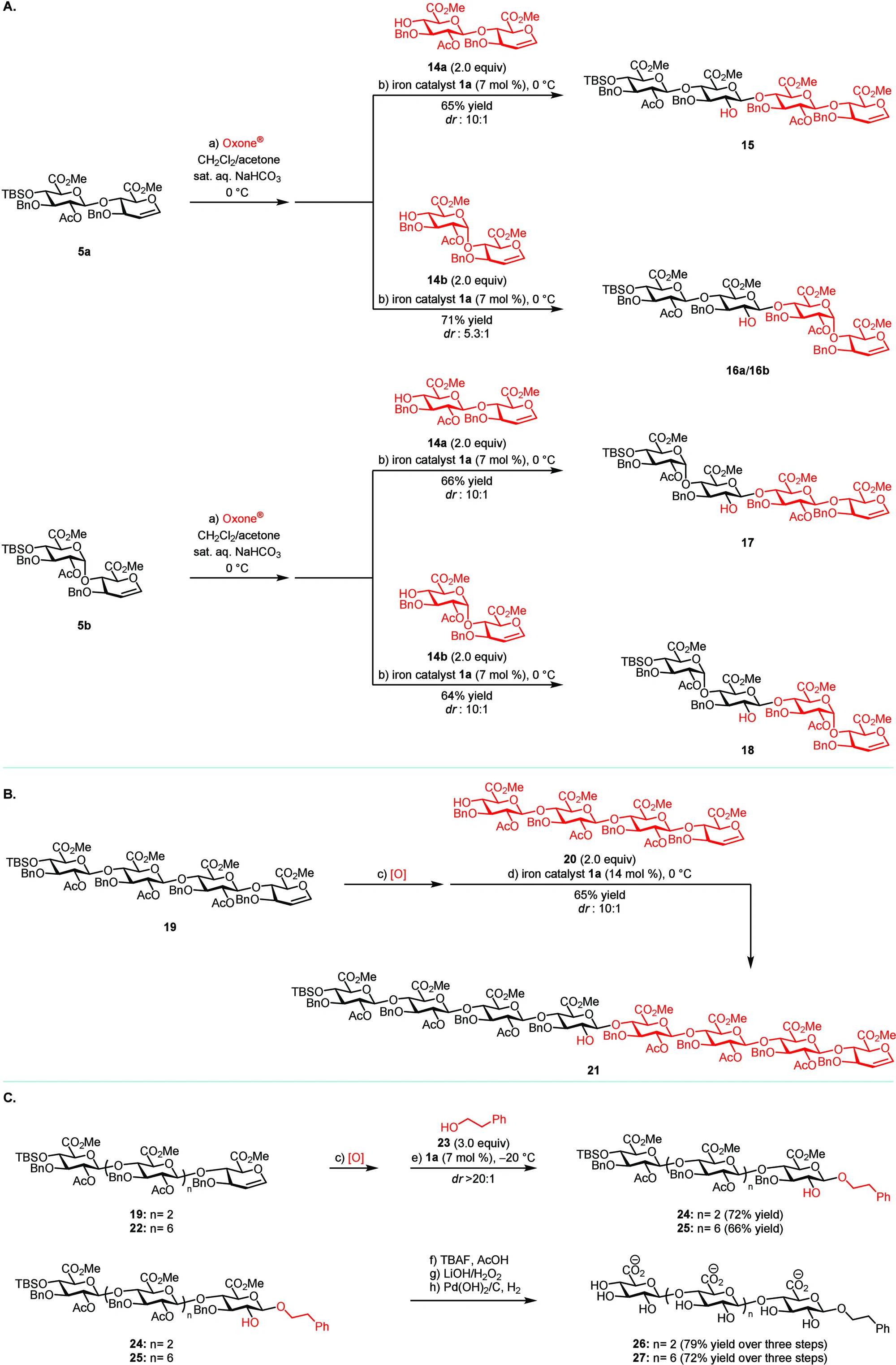
Iron-Catalyzed Stereospecific Glycosylation with Glycal Epoxides for PGA Tetrasaccharide and Octasaccharide Synthesis^a^

a Conditions: (a) Oxone (7.0 equiv for **5a** and 5.0 equiv for **5b**), 5:1 CH_2_Cl_2_/acetone, saturated NaHCO_3_ solution, 0 °C; (b) iron catalyst **1a** (7 mol %) and **14a**/**14b** (2.0 equiv) in CH_2_Cl_2_ at 0 °C; (c) DMDO (1.25 equiv for **19** and 1.5 equiv for **22**), CH_2_Cl_2_/acetone, −10 °C; (d) iron catalyst **1a** (14 mol %), **20** (2.0 equiv), CH_2_Cl_2_, 0 °C; (e) iron catalyst **1a** (7 mol %), **23** (3.0 equiv), CH_2_Cl_2_, −20 °C; (f) TBAF (3 equiv), AcOH (3 equiv), THF, 22 °C; (g) H_2_O_2_ (30% aqueous solution, 75 equiv for **24** and 150 equiv for **25**), LiOH (1 M aqueous solution, 25 equiv for **24** and 50 equiv for **25**), THF, 22 °C; (h) Pd­(OH)_2_/C, H_2_, 1 atm, MeOH/NaH_2_PO_4_-Na_2_HPO_4_ buffer (pH 7, 1:1), 22 °C. All yields are isolated yields. See the Supporting Information for details.

Surprisingly, iron-catalyzed glycosylation of GlcA-α-1,4-glucuronal module **14b** with the same glycal epoxide is only moderately stereospecific, affording an inseparable diastereomeric **16a**/**16b** mixture in excellent combined yield (71% combined yield, dr 5.3:1; for **16a**, ^3^ *J*_H1–H2_ = 7.8 Hz and ^1^ *J* ^13^_C1–H1_ = 162.8 Hz; for **16b**, ^3^ *J*_H1–H2_ = 3.7 Hz and ^1^ *J* ^13^_C1–H1_ = 170.6 Hz). Analogously, epoxidation of GlcA-α-1,4-glucuronal **5b** quantitatively afforded glucuronic ester α-epoxide **S10** (Figure S6; dr >20:1; ^3^ *J*_H1–H2_ = 2.5 Hz). We observed that iron catalyst **1a** is effective in inducing highly stereospecific glycosylation of both GlcA-β-1,4-glucuronal module **14a** and GlcA-α-1,4-glucuronal module **14b** with this epoxide, furnishing tetrasaccharides **17** and **18** in good yields (66% yield for **17**, dr 10:1; for **17**, ^3^ *J*_H1–H2_ = 7.6 Hz and ^1^ *J* ^13^_C1–H1_ = 162.9 Hz; 64% yield for **18**, *dr* 10:1; for **18**, ^3^ *J*_H1–H2_ = 7.3 Hz and ^1^ *J* ^13^_C1–H1_ = 162.3 Hz).

Acetylation of **15** almost quantitatively generated tetrasaccharide donor **19**, which can be smoothly desilylated (TBAF/AcOH, 86% yield) to afford its corresponding acceptor, **20** (Scheme 1B). Epoxidation of **19** furnished a glucuronic ester α-epoxide in >90% yield (dr >20:1; ^3^ *J*_H1–H2_ = 2.4 Hz), which glycosylated tetrasaccharide **20** in the presence of catalyst **1a**, delivering octasaccharide **21** in good yield (Scheme 1B, 65% yield, dr 10:1).

As a proof of concept, we selected tetrasaccharide **19** and octasaccharide **22** (>95% yield through acetylation of **21**) for derivatizaion to provide PGAs (Scheme 1c). Epoxidation of **19** and **22** afforded the corresponding glucuronic ester α-epoxides in high yields (>90% yield; dr >20:1; ^3^ *J*_H1–H2_ = 2.4 Hz), both of which readily glycosylated primary alcohol linker **23** with a chromophore, furnishing **24** and **25**, respectively (66–72% yield, dr >20:1). Desilylation of **24** and **25** with TBAF in AcOH, followed by hydrolysis (LiOH with H_2_O_2_) and global hydrogenolysis of benzyl groups, ultimately led to PGAs **26** and **27**, respectively, in good yields (72–79%).

In conclusion, we have developed a convergent and stereoselective polyglucuronic acid assembly method via iron-catalyzed stereospecific glycosylation with glycal epoxides. This iterative and stereospecific glycosylation is effective for the synthesis of an array of GlcA-β-1,4-GlcA linkages in complex glycans, and it rapidly provides PGA tetrasaccharides and octasaccharides. Our current effort focuses on applications of this method for assembly of other synthetically challenging, uronic acid-containing complex glycans.

## Supplementary Material



## Data Availability

The data underlying this study are available in the published article and its Supporting Information.

## Notes

Experimental procedure, characterization data for all new compounds, and selected NMR spectra (PDF)
