# Supplementary material for: Stereoselective Polyglucuronic Acid Assembly via the Iron-Catalyzed Stereospecific Glycosylation with Glycal Epoxides
Source: Org Lett. Author manuscript; Available in PMC 2026 Oct 24. (PMC13640433; doi:10.1021/acs.orglett.6c03166)
Supplement: Supplementary file 1 [file NIHMS2212388-supplement-Supplementary_Material.pdf]

Supporting Information

Stereoselective Polyglucuronic Acid Assembly via the Iron-Catalyzed Stereospecific Glycosylation with Glycal Epoxides

Xiao-Wen Zhang, Zixiang Jiang, and Hao Xu*

haohxu@brandeis.edu

Department of Chemistry, Brandeis University, 415 South Street, Waltham, Massachusetts 02453, United States

A. General Information

B. Catalyst Discovery for the Iron-Catalyzed Stereospecific Glycosylation of C4-OH Glucuronals with Glucuronic Ester Epoxides

C. General Procedures of the Iron-Catalyzed Stereospecific Glycosylation for GlcA-β-1,4-GlcA Assembly

D. Iron-Catalyzed Stereospecific Glycosylation with Glycal Epoxides for PGA Tetrasaccharide Synthesis

E. Iron-Catalyzed Stereospecific Glycosylation with Glycal Epoxides for PGA Octasaccharide Synthesis

F. References

G. NMR Spectra

A. General Information

General Procedures. All reactions were performed in oven-dried or flame-dried round-bottom flasks and vials. Stainless steel syringes and cannula were used to transfer air- and moisture-sensitive liquids. Flash chromatography was performed using silica gel 60 (230−400 mesh) from Sigma–Aldrich.

Materials. Commercial reagents were purchased from Sigma–Aldrich, TCI, Oakwood Chemicals, Combi-Blocks, Chem-Impex, Thermo Fischer Scientific and used as received. All solvents were used after being freshly distilled unless otherwise noted.

Instrumentation. Proton nuclear magnetic resonance (^{1}H NMR) spectra and carbon nuclear magnetic resonance (^{13}C NMR) spectra were recorded on Advance NEO 400 (400 MHz) and Varian 400-MR (400 MHz). Chemical shifts for protons are reported in parts per million downfield from tetramethylsilane and are referenced to the NMR solvent residual peak ($CHCl_3$ δ 7.26, CD_3OD δ 3.31, acetone-d_6 δ 2.05, D_2O δ 4.79). Chemical shifts for carbons are reported in parts per million downfield from tetramethylsilane and are referenced to the carbon resonances of the NMR solvent ($CDCl_3$ δ 77.0, CD_3OD δ 49.0, acetone-d_6 δ 29.8). Data are represented as follows: chemical shift, multiplicity (br = broad, s = singlet, d = doublet, t = triplet, q = quartet, quint = quintet, m = multiplet), coupling constants in Hertz (Hz), and integration. The mass spectroscopic data were obtained using a Bruker timsTOF Pro instrument by electrospray ionization (ESI). Infrared (IR) spectra were obtained using a Nicolet IR200 spectrometer with a diamond ATR. Data are represented as follows: frequency of absorption (cm^{-1}) and absorption strength (s = strong, m = medium, w = weak). Structural assignments were made with additional information from gCOSY, gHSQC, and gHMBC experiments.

Abbreviations Used: THF–tetrahydrofuran, EtOAc–ethyl acetate, Et_2O–diethyl ether, CH_2Cl_2–dichloromethane, TMSOTf–trimethylsilyl trifluoromethanesulfonate, TLC–thin layer chromatography, Ac_2O−acetic anhydride, TBAF−tetra-*n*-butylammonium fluoride, OTf−trifluoromethanesulfonate, $AgSO_3C_6H_4C_{12}H_{25}$−silver 4-dodecylbenzenesulfonate (mixture of isomers), DMDO−dimethyldioxirane, All−allyl.

B. Catalyst Discovery for the Iron-Catalyzed Stereospecific Glycosylation of C4-OH Glucuronals with Glucuronic Ester Epoxides

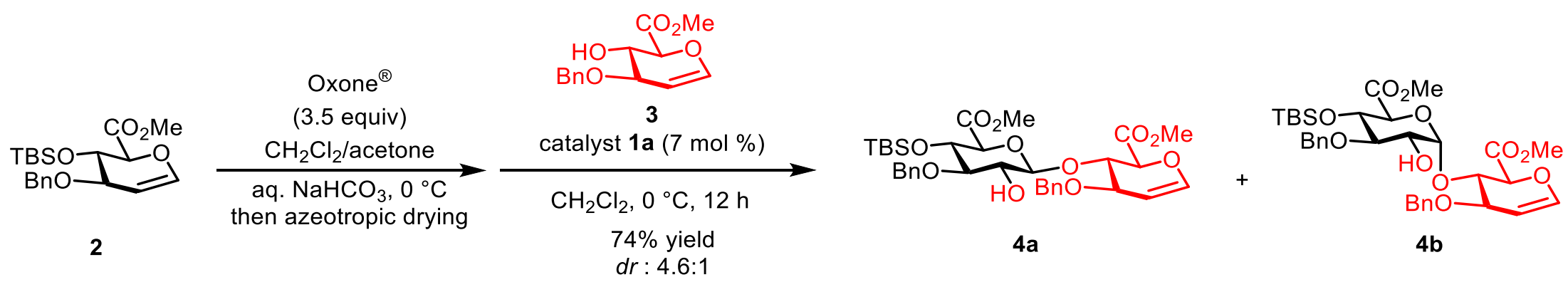

a. General Procedures for Catalyst Discovery

The Procedure for Glycal Epoxidation

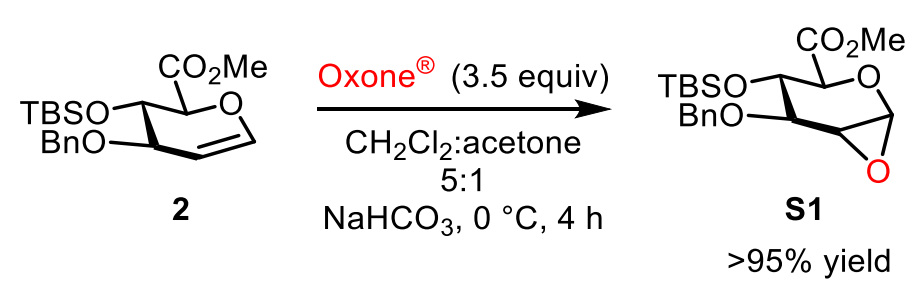

To a 500 mL flask equipped with a stir bar at 0 °C were added glycal **2**[1] (3.59 g, 9.5 mmol, 1.0 equiv) in CH_2Cl_2/acetone mixture (v/v: 5:1, 95 mL) and saturated aqueous $NaHCO_3$ solution (150 mL), followed by addition of Oxone® ($KHSO_5 \cdot 0.5KHSO_4 \cdot 0.5K_2SO_4$) (20.4 g, 332.5 mmol, 3.5 equiv) in H_2O (100 mL).[2] After stirring vigorously at 0 °C for 4 h, the reaction mixture was extracted with CH_2Cl_2 (50 mL × 3). The combined organic phase was dried over anhydrous Na_2SO_4 and concentrated *in vacuo*. The residue was further azeotropically dried with anhydrous toluene (1.0 mL × 3). The obtained glycal epoxide **S1** was assayed by ^{1}H NMR to get the diastereomeric ratio (*dr* > 20:1) and directly used in the next step.

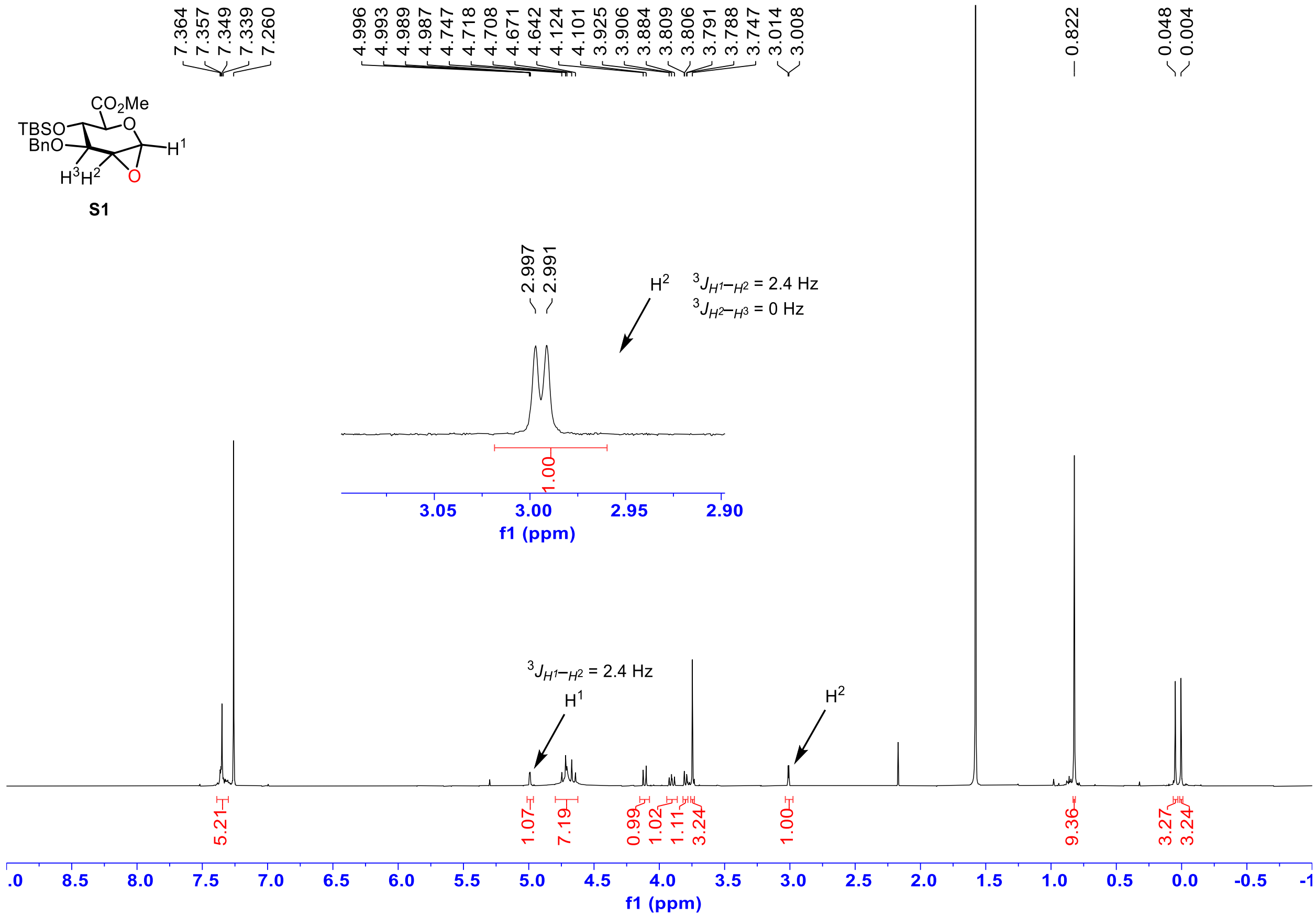

Figure S1. Stereochemistry Determination of Glycal Epoxide **S1**.

The stereochemistry of the glycal epoxide **S1** was determined by ^{1}H NMR analysis. It is known that the C(2)–H chemical shift of a glucal α-epoxide is around 3.0 ppm, whereas that of a glucal β-epoxide is around 3.3 ppm.[3]

Additionally, the $^{3}J_{H1-H2}$ value for a typical glucal α-epoxide is around 2.4 Hz, reflecting the dihedral angle ($\Phi_{1,2}$) that approaches 0°, whereas the $^{3}J_{H2-H3}$ value is < 0.5 Hz because the dihedral angle ($\Phi_{2,3}$) approaches 90°, characteristic of a *trans*-relationship between H^2 and H^3 that is pseudo-axial.[3] As a result, the H^2 signal of a glucal α-epoxide appears as a doublet.

In contrast, while a glucal β-epoxide retains a similar $\Phi_{1,2}$ and thus a comparable $^3J_{H1-H2}$, its $\Phi_{2,3}$ is around 45°, leading to a $^3J_{H2-H3}$ around 1.7 Hz.[3] This causes the H^2 signal to appear as a doublet of doublets (though often observed as a triplet due to the broadening line width).

Based on these data, we have assigned glycal epoxide **S1** as an α-epoxide (*dr* > 20:1) with $^3J_{H1-H2}$ = 2.4 Hz and $^3J_{H2-H3}$ = 0 Hz. This assignment was further corroborated by the stereochemistry analysis of the iron-catalyzed glycosylation products **4a** and **4b**.

Procedures for Preparation of Iron(III) Porphyrin Triflate and Other Sulfonate Catalysts

To a flame-dried sealable 2-dram vial equipped with a stir bar were added an iron(III) porphyrin chloride catalyst **S2** (0.012 mmol, 10 mol %) and AgOTf (2.2 mg, 0.0084 mmol, 7 mol %). After the vial was evacuated and backfilled with N_2 three times, anhydrous CH_2Cl_2 (0.3 mL) was added and the solution was stirred at room temperature for 10 min.

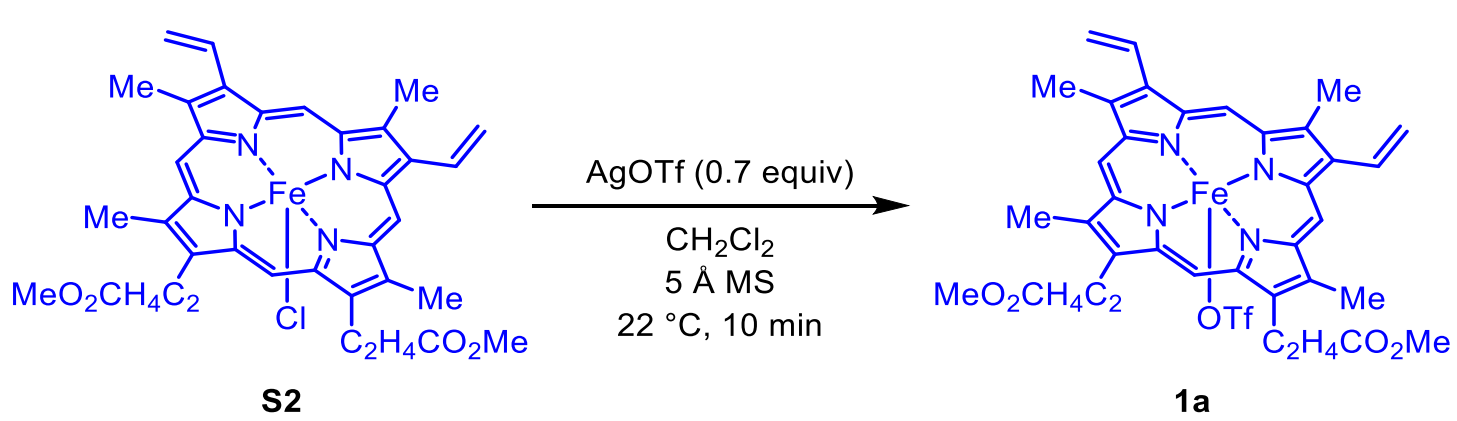

To prepare other iron(III) porphyrin sulfonate catalysts, the corresponding silver sulfonate (0.0084 mmol, 7 mol %) was used instead of AgOTf, and the solution was stirred for 30–60 min at room temperature.

The Procedure for Catalyst Discovery for the Iron-Catalyzed Stereospecific Glycosylation of C4-OH Glucuronals with Glucuronic Ester Epoxides

To a flame-dried sealable 2-dram vial equipped with a stir bar were added glycal epoxide **S1** (0.1 mmol, 1.0 equiv) and freshly activated 5 Å molecular sieves, powder (*ca.* 100 mg). After the vial was evacuated and backfilled with N_2 three times, glycosyl acceptor **3**[4] (0.2 mmol, 2.0 equiv)

in anhydrous CH_2Cl_2 (0.4 mL) was added at −40 °C dropwise into the mixture. After the mixture was stirred at −40 °C for 5 min, iron catalyst (0.007 mmol, 7 mol %) in anhydrous CH_2Cl_2 (0.1 mL) was added dropwise. The reaction mixture was kept at −40 °C for an additional 5 min before being warmed up to 0 °C. The reaction mixture was stirred at 0 °C for 12 h and then quenched with imidazole (8.5 mg in 1 mL CH_2Cl_2) at the same temperature. The mixture was then filtered through a piece of cotton and eluted with EtOAc (2 mL × 2). The organic layer was concentrated *in vacuo*. The *dr* was determined based on the ^{1}H NMR analysis of the crude reaction mixture. The residue was purified through a silica gel flash column (hexanes/EtOAc: from 20:1 to 2:1) to afford the desired glycosylation products **4a/4b** as white foam.

Table S1. Catalyst Discovery for the Iron-Catalyzed Stereospecific Glycosylation of C4-OH Glucuronals with Glucuronic Ester Epoxides

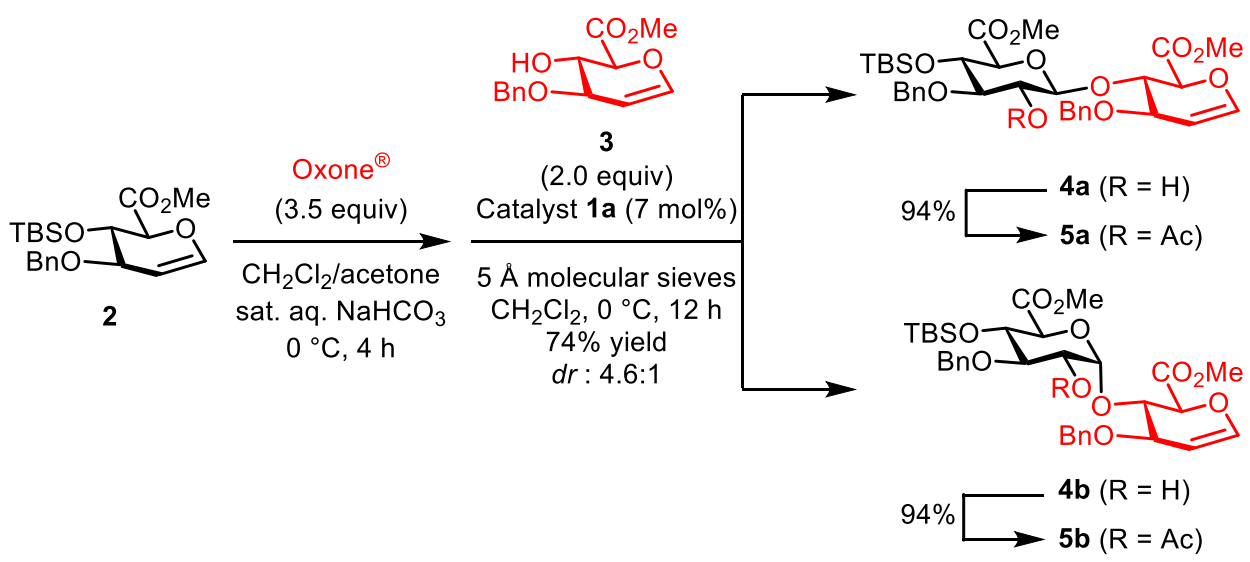

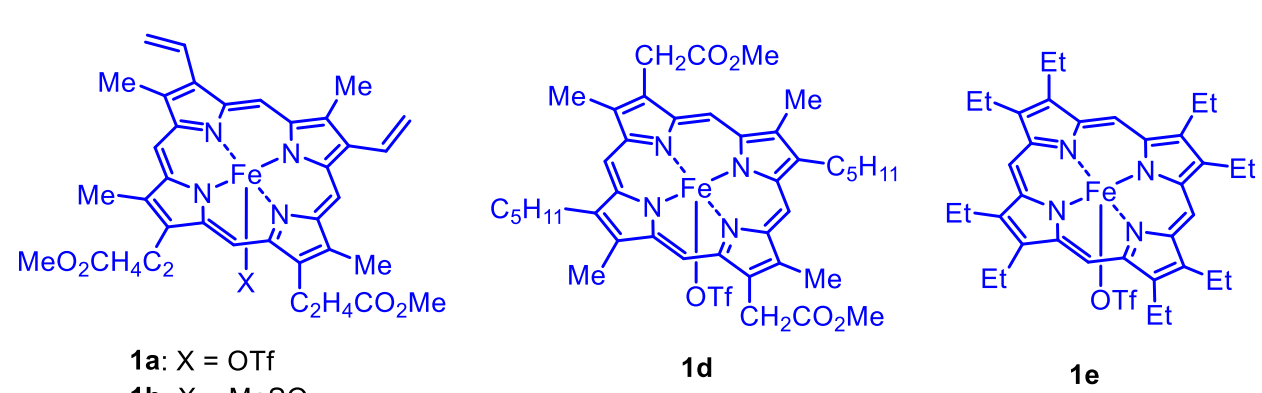

entry[a]	variation from the optimal condition	conversion (%)	yield of **4a**/**4b** (%)[b]	*dr*[b]
1	no variation	>95	74	4.6:1
2	carry out the glycosylation at –20 °C or –40 °C, 24 h	>95	46–55	4.6:1
3	replace **1a** with **1b**	>95	64	1.6:1
4	replace **1a** with **1c**	>95	41	2.5:1
5	replace **1a** with TMSOTf (5 mol %), –40 °C, 30 min	>95	54	2.4:1
6	replace **1a** with AgOTf (10 mol %)	35	23	2.6:1
7	replace **1a** with **1d** or **1e**	>95	65	4.3:1

[a]Epoxidation was carried out in a biphasic reaction medium with Oxone® and acetone. The glycal epoxide was dried azeotropically with toluene, assayed by ^{1}H NMR, and then directly used. The glycosylation was carried out at 0 °C in CH_2Cl_2. [b]Isolated yield; *dr* was determined by ^{1}H NMR analysis. Iron(III) porphyrin catalysts **1a**–**1e** were formed in situ from the corresponding iron porphyrin chloride (10 mol %) and AgOTf (7 mol %) to avoid AgOTf in excess.

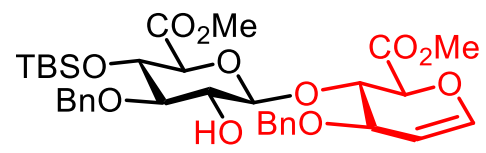

4a

Methyl 3-*O*-benzyl-4-*O*-(methyl 3-*O*-benzyl-4-*O*-*tert*-butyldimethylsilyl-β-D-glucopyranosyluronate)-D-glucuronal (**4a**): IR ν_{max} (neat)/cm^{-1}: 3499 (w), 2952 (w), 2927 (w), 2855 (w), 1753 (s), 1651 (m), 1249 (m), 1066 (s), 838 (m); ^{1}H NMR (400 MHz, $CDCl_3$) δ 7.41 – 7.23 (m, 10H), 6.59 (d, *J* = 6.3 Hz, 1H), 5.02 – 4.94 (m, 2H), 4.79 – 4.66 (m, 2H), 4.55 (d, *J* = 7.6 Hz, 1H), 4.53 – 4.42 (m, 3H), 3.97 – 3.88 (m, 3H), 3.76 (s, 3H), 3.63 – 3.53 (m, 1H), 3.57 (s, 3H), 3.43 (ddd, *J* = 8.7, 5.7, 2.5 Hz, 1H), 2.41 (d, *J* = 2.7 Hz, 1H), 0.84 (s, 9H), 0.02 (s, 3H), -0.02 (s, 3H); ^{13}C NMR (100 MHz, $CDCl_3$) δ 168.6, 168.1, 144.9, 138.6, 137.8, 128.3 (2C), 128.2 (2C), 127.9 (2C), 127.7, 127.5 (2C), 127.4, 102.3, 98.8, 83.4, 76.7, 74.6, 74.3, 73.2, 72.5, 71.7, 69.8, 68.1, 52.4, 52.3, 25.7 (3C), 17.9, -4.0, -5.4; HRMS: m/z (ESI) calcd for $C_{34}H_{47}O_{11}Si^{+}$, [M + H]$^{+}$, 659.2882, found 659.2896. $^{1}J^{13}_{C1-H1}$ = 159.2 Hz.

Stereochemistry Determination of Glycosylation Product 4a

The stereochemistry of newly formed anomeric center (C1) of **4a** was determined by measuring $^{1}J^{13}_{C1-H1}$ (159.2 Hz) through un-decoupled HSQC experiments. The C2 stereochemistry was determined by measuring $^{3}J_{H1-H2}$ (7.6 Hz).

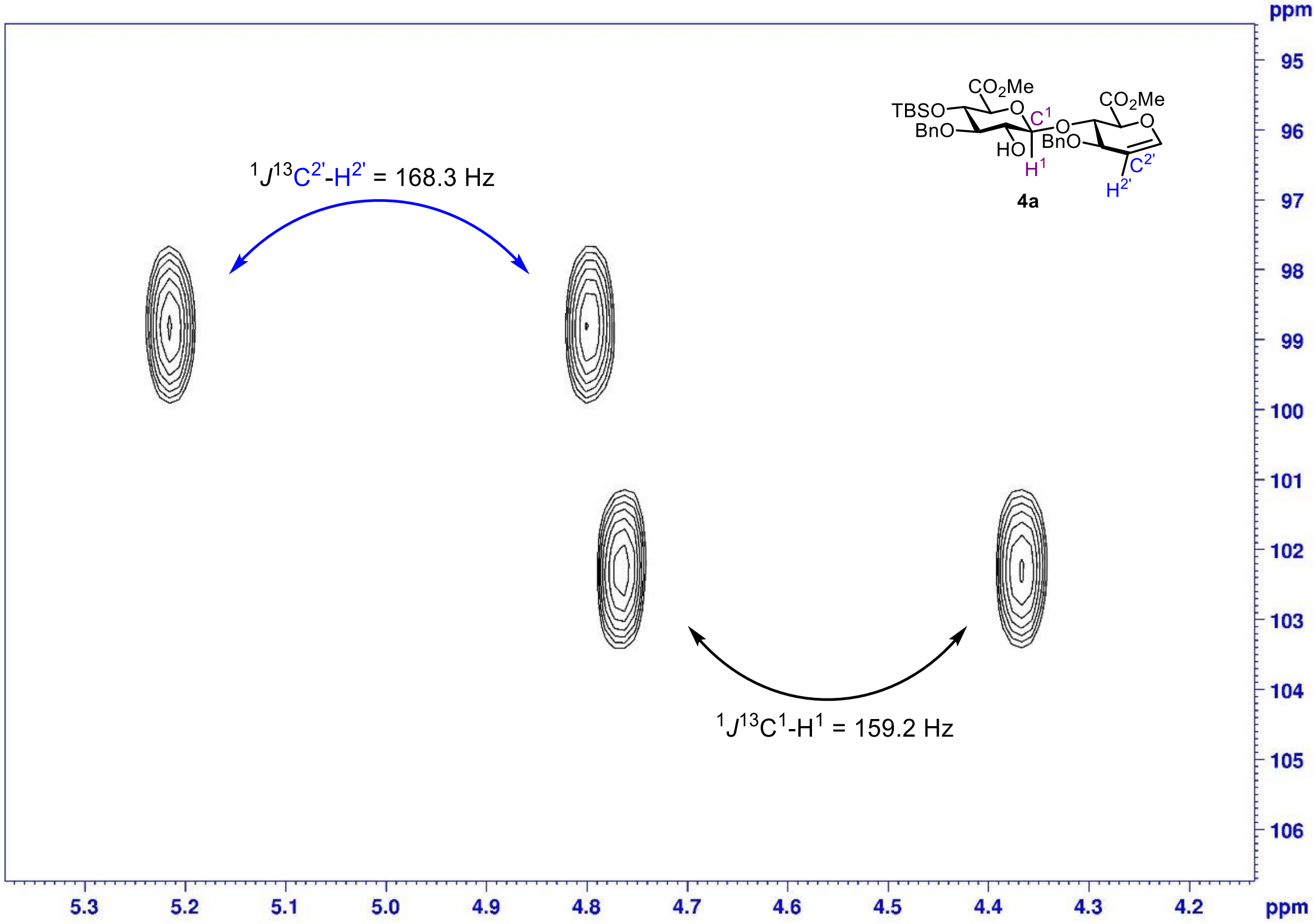

Figure S2. Un-decoupled HSQC Analysis to Determine Stereochemistry of **4a**.

A $^{1}J^{13}{}_{C1-H1}$ value of 159.2 Hz suggested that the newly formed glycosidic bond is in equatorial position. The $^{3}J_{H1-H2}$ value of 7.6 Hz suggested that the H2 is in axial position and corroborated the exclusive formation of the α-epoxide in the previous step of glycal epoxidation.

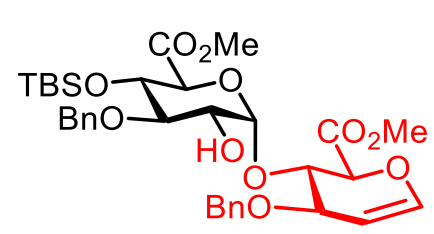

4b

Methyl 3-*O*-benzyl-4-*O*-(methyl 3-*O*-benzyl-4-*O*-*tert*-butyldimethylsilyl-α-D-glucopyranosyluronate)-D-glucuronal (4b): IR ν_{max} (neat)/cm^{-1}: 3522 (w), 2951 (w), 2928 (w), 2856 (w), 1751 (s), 1649 (m), 1248 (m), 1056 (s), 838 (m); ^{1}H NMR (400 MHz, $CDCl_3$) δ 7.37 –

7.25 (m, 10H), 6.67 (d, J = 6.3 Hz, 1H), 5.16 (d, J = 3.4 Hz, 1H), 5.01 (t, J = 5.6 Hz, 1H), 4.93 (d, J = 3.1 Hz, 1H), 4.75 (ABq, $\Delta\nu_{AB}$ = 63.8 Hz, J = 11.4 Hz, 2H), 4.57 – 4.49 (m, 2H), 4.46 (d, J = 11.3 Hz, 1H), 4.27 (d, J = 7.7 Hz, 1H), 3.95 (t, J = 7.5 Hz, 1H), 3.85 (d, J = 4.5 Hz, 1H), 3.75 (s, 3H), 3.75 – 3.65 (m, 1H), 3.59 – 3.52 (m, 1H), 3.57 (s, 3H), 2.21 (d, J = 10.5 Hz, 1H), 0.85 (s, 9H), 0.02 (s, 3H), -0.00 (s, 3H); ^{13}C NMR (100 MHz, $CDCl_3$) δ 169.6, 168.1, 145.5, 138.1, 137.6, 128.4 (2C), 128.3 (2C), 127.9 (2C), 127.8, 127.6 (two peaks overlapped, 3C), 98.3, 98.0, 80.9, 74.4, 74.1, 73.9, 72.8, 71.6, 71.0, 69.6, 67.2, 52.4, 52.2, 25.7 (3C), 17.9, -4.3, -5.3; HRMS: m/z (ESI) calcd for $C_{34}H_{47}O_{11}Si^+$, $[M + H]^+$, 659.2882, found 659.2901. $^{1}J_{^{13}C1–H1}$ = 171.3 Hz.

Stereochemistry Determination of Glycosylation Product 4b

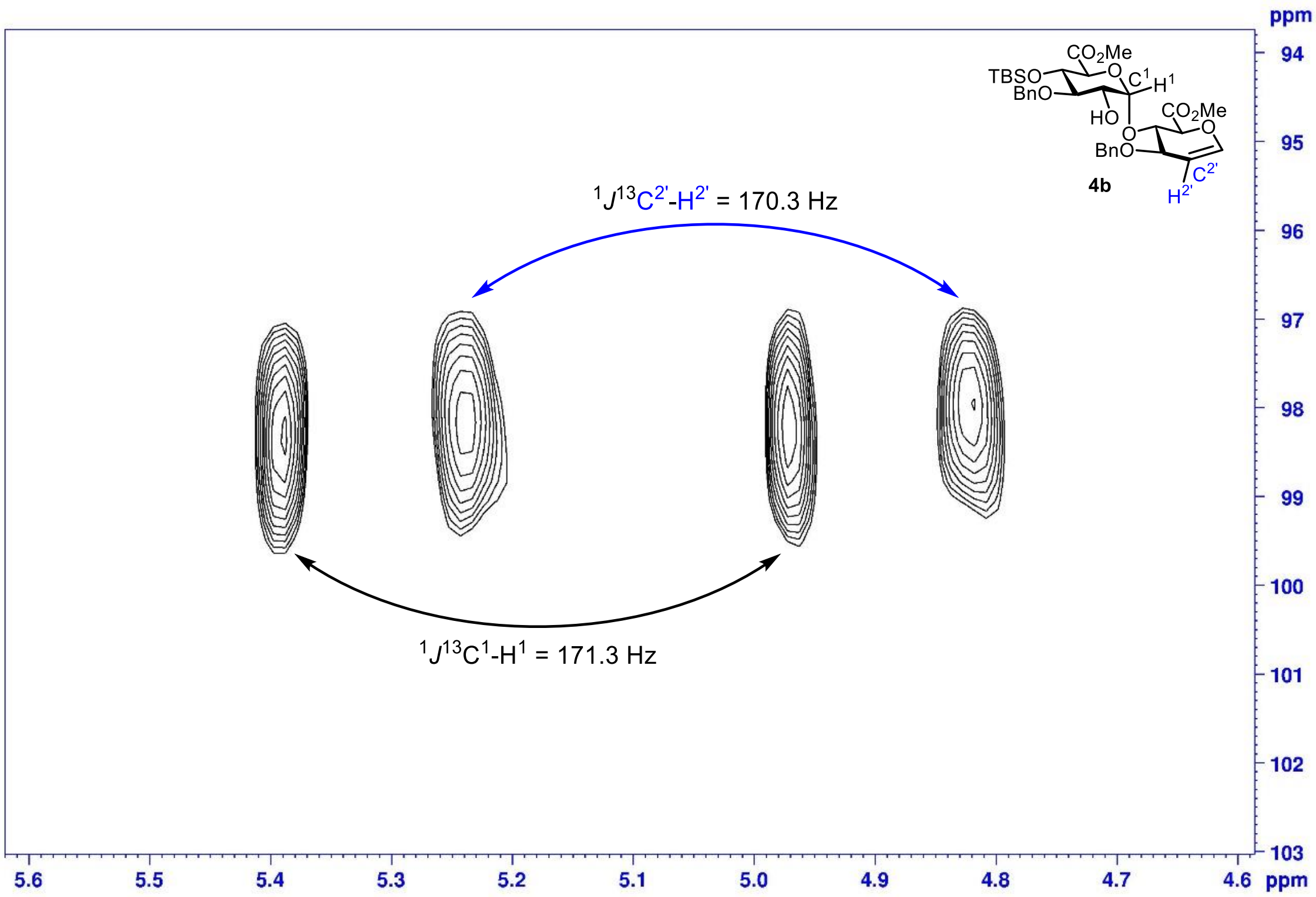

Figure S3. Un-decoupled HSQC Analysis to Determine Stereochemistry of **4b**.

The stereochemistry of newly formed anomeric center (C1) of **4b** was determined by measuring $^{1}J^{13}_{C1\text{–}H1}$ (171.3 Hz) through un-decoupled HSQC experiments. The C2 stereochemistry was corroborated by measuring $^{3}J_{\mathrm{H1–H2}}$ (3.4 Hz).

A $^{1}J^{13}_{C1\text{–}H1}$ value of 171.3 Hz suggested that the newly formed glycosidic bond is in axial position.

b. The Procedure for Multigram-Scale Glycosylation

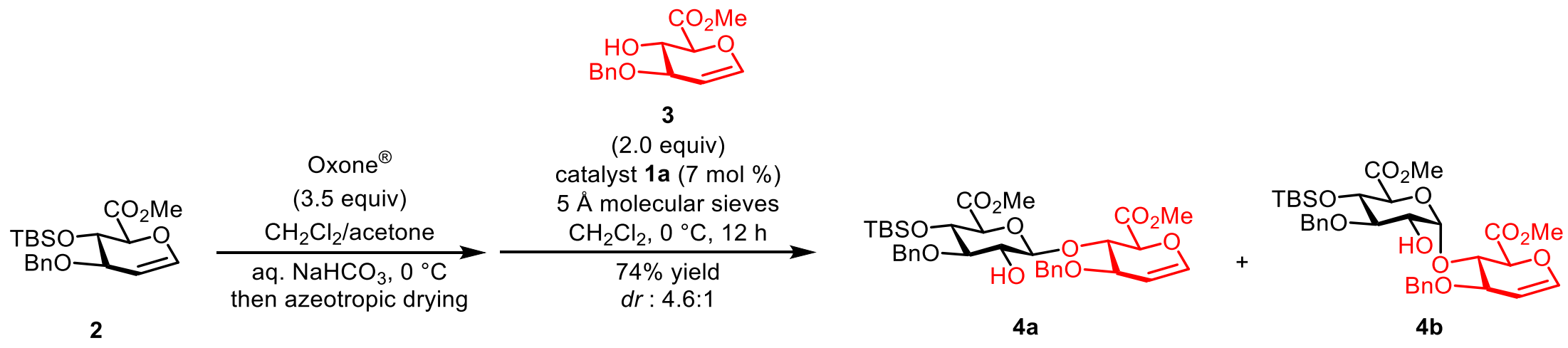

To a flame-dried 100 mL flask equipped with a stir bar were added a glycal epoxide **S1** (3.73 g, 9.5 mmol, 1.0 equiv) and freshly activated 5 Å molecular sieves, powder (*ca.* 5.0 g). After the vial was evacuated and backfilled with N_2 three times, a glycosyl acceptor **3** (5.1 g, 19.0 mmol, 2.0 equiv) in anhydrous CH_2Cl_2 (30 mL) was added at −40 °C dropwise into the mixture. After the mixture was stirred at −40 °C for 5 min, iron catalyst **1a** (0.67 mmol, 7 mol %) in anhydrous CH_2Cl_2 (17.5 mL) was added dropwise. The reaction mixture was kept at −40 °C for an additional 5 min before being warmed up to 0 °C. The reaction mixture was stirred at 0 °C for 12 h and then quenched with imidazole at the same temperature. The mixture was then filtered and eluted with EtOAc (20 mL × 2). The organic layer was concentrated *in vacuo*. The desired product was separated through column chromatography (hexanes/EtOAc: from 20:1 to 2:1) as mixture of **4a**/**4b** as white foam (4.63 g, 74% yield, *dr* : 4.6:1).

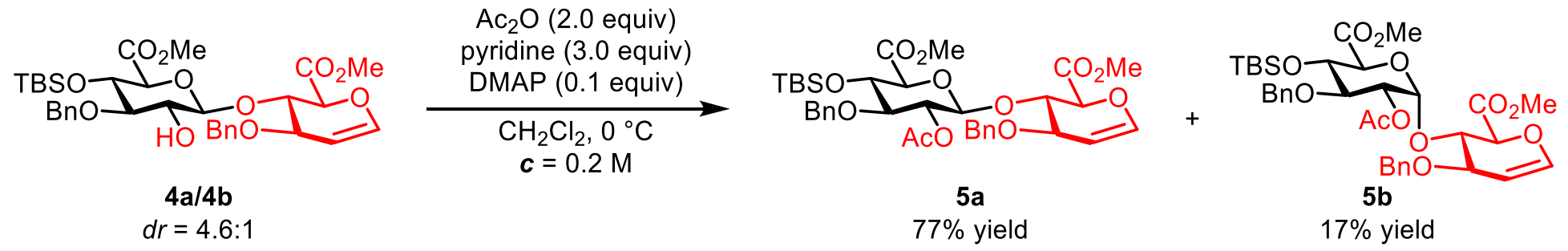

To a solution of **4a/4b** (4.63 g, 7.0 mmol, 1.0 equiv) and DMAP (85.4 mg, 0.7 mmol, 0.1 equiv) in anhydrous CH_2Cl_2 (25 mL) was added pyridine (1.69 mL, 21.0 mmol, 3.0 equiv) at 0 °C. After stirring at 0 °C for 10 min, Ac_2O (1.32 mL, 14.0 mmol, 2.0 equiv) was added dropwise. The reaction mixture was kept at 0 °C and stirred for 3 h, with progress monitored by TLC until completion. The reaction was then quenched with saturated aqueous $NaHCO_3$ solution (5 mL). The organic phase was separated from the aqueous one, which was then extracted with EtOAc (5 mL × 3). The combined organic layer was washed with brine, dried over anhydrous Na_2SO_4, and then concentrated *in vacuo*. The desired product was separated through column chromatography (hexanes/acetone: from 20:1 to 4:1) as white foam for **5a** (3.77 g, 77% yield) and white foam for **5b** (0.83 g, 17% yield).

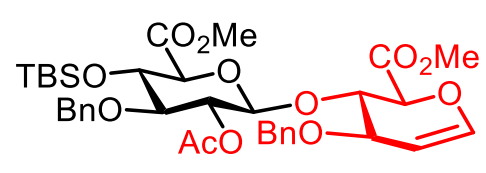

5a

Methyl 3-*O*-benzyl-4-*O*-(methyl 2-*O*-acetyl-3-*O*-benzyl-4-*O*-*tert*-butyldimethylsilyl-β-D-glucopyranosyluronate)-D-glucuronal (**5a**): IR ν_{max} (neat)/cm^{-1}: 2952 (w), 2928 (w), 2856 (w), 1749 (s), 1361 (m), 1219 (s), 1066 (s), 1042 (s), 837 (s); ^{1}H NMR (400 MHz, $CDCl_3$) δ 7.36 – 7.21 (m, 10H), 6.53 (d, *J* = 6.3 Hz, 1H), 5.02 (dd, *J* = 8.8, 7.7 Hz, 1H), 4.97 (ddd, *J* = 6.5, 5.3, 1.5 Hz, 1H), 4.74 – 4.67 (m, 2H), 4.67 – 4.60 (m, 2H), 4.51 (d, *J* = 11.3 Hz, 1H), 4.47 – 4.39 (m, 2H), 4.02 (dd, *J* = 9.0, 8.1 Hz, 1H), 3.94 (d, *J* = 9.0 Hz, 1H), 3.92 – 3.88 (m, 1H), 3.78 (s, 3H), 3.54 (s, 3H), 3.52 (t, *J* = 8.4 Hz, 1H), 1.88 (s, 3H), 0.85 (s, 9H), -0.00 (s, 3H), -0.03 (s, 3H); ^{13}C NMR (100 MHz, $CDCl_3$) δ 169.2, 168.5, 168.2, 144.7, 137.91, 137.88, 128.3 (2C), 128.2 (2C), 127.9 (2C), 127.61, 127.55, 127.4 (2C), 99.4, 98.5, 82.3, 76.8, 74.7, 72.53, 72.49, 72.1, 72.0, 69.7, 67.9, 52.4, 52.2, 25.7 (3C), 20.7, 17.9, -4.1, -5.3; HRMS: m/z (ESI) calcd for $C_{36}H_{49}O_{12}Si^+$, $[M + H]^+$, 701.2988, found 701.3007. $^1J_{^{13}C1–H1}$ = 162.2 Hz.

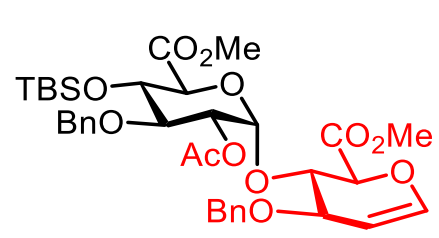

5b

Methyl 3-*O*-benzyl-4-*O*-(methyl 2-*O*-acetyl-3-*O*-benzyl-4-*O*-*tert*-butyldimethylsilyl-α-D-glucopyranosyluronate)-D-glucuronal (**5b**): IR ν_{max} (neat)/cm^{-1}: 2953 (w), 2929 (w), 2858 (w), 1752 (s), 1360 (m), 1218 (s), 1069 (s), 1036 (s), 837 (s); ^{1}H NMR (400 MHz, $CDCl_3$) δ 7.36 – 7.23 (m, 10H), 6.69 (d, *J* = 6.3 Hz, 1H), 5.36 (d, *J* = 3.9 Hz, 1H), 4.98 (ddd, *J* = 6.6, 5.2, 1.6 Hz, 1H), 4.87 (dd, *J* = 2.8, 1.4 Hz, 1H), 4.82 – 4.70 (m, 3H), 4.45 (ABq, $\Delta\nu_{AB}$ = 25.7 Hz, *J* = 11.4 Hz, 2H), 4.37 (q, *J* = 2.4 Hz, 1H), 4.23 (d, *J* = 9.5 Hz, 1H), 3.92 (dd, *J* = 9.5, 8.7 Hz, 1H), 3.78 (s, 3H), 3.81 – 3.75 (m, 1H), 3.70 – 3.65 (m, 1H), 3.55 (s, 3H), 1.79 (s, 3H), 0.85 (s, 9H), -0.01 (s, 3H), -0.02 (s, 3H); ^{13}C NMR (100 MHz, $CDCl_3$) δ 170.1, 169.4, 167.9, 145.4, 138.7, 137.6, 128.3 (2C), 128.2 (2C), 127.9 (2C), 127.8, 127.3, 126.7 (2C), 98.0, 96.5, 79.7, 75.0, 73.7, 73.6, 72.9, 72.8, 72.1, 69.4, 66.7, 52.4, 52.1, 25.7 (3C), 20.5, 17.9, -4.1, -5.3; HRMS: m/z (ESI) calcd for $C_{36}H_{49}O_{12}Si^+$, $[M + H]^+$, 701.2988, found 701.2998. $^{1}J_{^{13}C1-H1}$ = 170.6 Hz.

C. General Procedures of the Iron-Catalyzed Stereospecific Glycosylation for GlcA-β-1,4-GlcA Assembly

a. General Procedures for Glycal Epoxidation

Procedure A

To a 25 mL flask equipped with a stir bar at 0 °C were added a glycal (0.1 mmol, 1.0 equiv) in CH_2Cl_2/acetone mixture (v/v: 5:1, 1.7 mL) and saturated aqueous $NaHCO_3$ solution (2.8 mL), followed by addition of Oxone® ($KHSO_5 \cdot 0.5KHSO_4 \cdot 0.5K_2SO_4$) (215.5 mg, 0.35 mmol, 3.5 equiv) in H_2O (1.8 mL) dropwise. After stirring vigorously at 0 °C for 2 h, the reaction mixture was extracted with CH_2Cl_2 (3 mL × 3). The combined organic phase was dried over anhydrous Na_2SO_4 and concentrated *in vacuo*. The residue was further azeotropically dried with anhydrous toluene (1.0 mL × 3). The obtained glycal epoxide was assayed by ^{1}H NMR to get the diastereomeric ratio and directly used in the next step.

Procedure B

For glycals that are hydrophilic, an alternative epoxidation procedure has been applied.

To a 25 mL flask equipped with a stir bar at 0 °C were added a glycal (0.1 mmol, 1.0 equiv) in CH_2Cl_2, followed by the addition of freshly prepared dimethyldioxirane (DMDO in acetone, 0.06 M, 2.5 mL, 0.15 mmol, 1.5 equiv)[5] dropwise. After stirring at 0 °C for 2 h, the reaction mixture was concentrated *in vacuo*, then the residue was re-dissolved in anhydrous CH_2Cl_2 (2 mL) and dried over anhydrous Na_2SO_4. The organic phase concentrated *in vacuo*. The residue was further dried azeotropically with anhydrous toluene (1.0 mL × 3). The obtained glycal epoxide was assayed by ^{1}H NMR to get the diastereomeric ratio and directly used in the next step.

b. The Procedure of the Iron-Catalyzed Stereospecific Glycosylation for GlcA-β-1,4-GlcA Assembly

Procedure C

To a flame-dried sealable 2-dram vial equipped with a stir bar were added a glycal epoxide (0.1 mmol, 1.0 equiv) and freshly activated 5 Å molecular sieves, powder (*ca.* 100 mg). After the vial was evacuated and backfilled with N_2 three times, a glycosyl acceptor (0.2 mmol, 2.0 equiv) in anhydrous CH_2Cl_2 (0.4 mL) was added at −40 °C dropwise into the mixture. After the mixture was stirred at −40 °C for 5 min, iron catalyst **1a** (0.007 mmol, 7 mol %) in anhydrous CH_2Cl_2 (0.1 mL) was added dropwise. The reaction mixture was kept at −40 °C for an additional 5 min before being warmed up to −20 or 0 °C. The reaction mixture was stirred at −20 for 24 h or 0 °C for 12 h and then quenched with imidazole (8.5 mg in 1 mL CH_2Cl_2) at the same temperature. The mixture was then filtered through a piece of cotton and eluted with EtOAc (2 mL × 2). The organic layer was concentrated *in vacuo*. The *dr* was determined based on the ^{1}H NMR analysis of the crude reaction mixture. The residue was purified through a silica gel flash column to afford the desired glycosylation product.

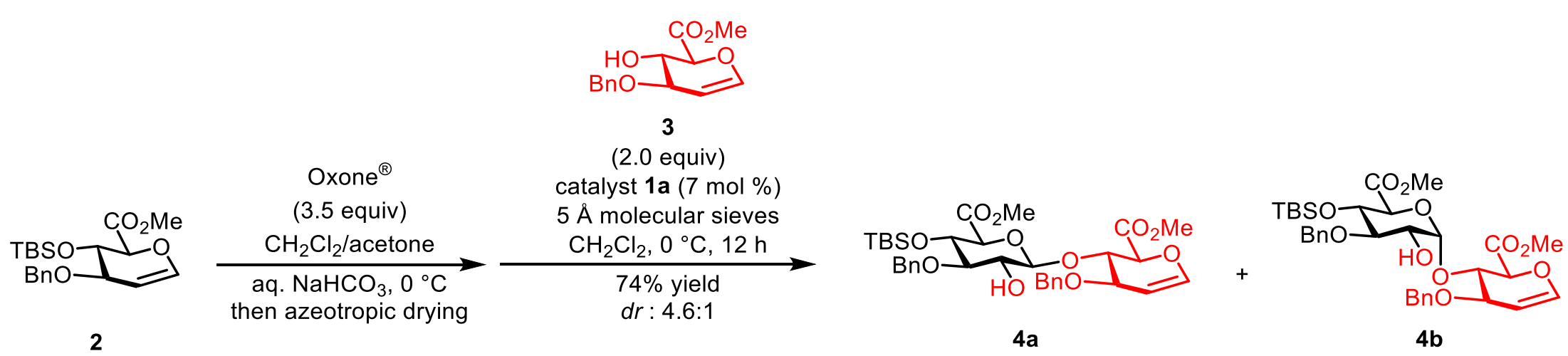

The reaction was carried out on a 9.3 mmol scale by following **Procedure A** for epoxidation and **Procedure C** for the iron-catalyzed stereospecific glycosylation at 0 °C. The *dr* of the corresponding glycal α-epoxide is >20:1. The *dr* of the glycosylation product determined based on crude reaction mixture is 4.6:1. The desired product was purified through a silica gel flash column (hexanes/EtOAc: from 20:1 to 2:1) as white foam for **4a**/**4b** (4.63 g, 74% yield, *dr* : 4.6:1).

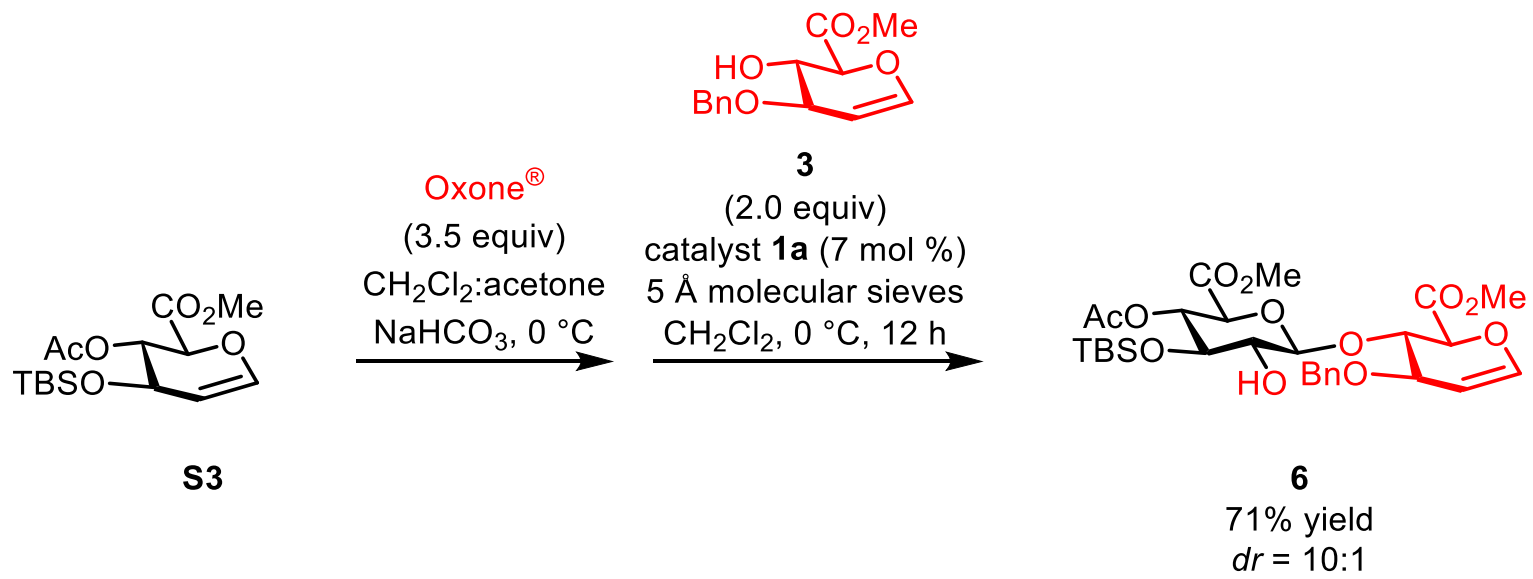

S3 was synthesized according to a literature procedure.[6]

The reaction was carried out on a 0.1 mmol scale by following **Procedure A** for epoxidation and **Procedure C** for the iron-catalyzed stereospecific glycosylation at 0 °C. The *dr* of the corresponding glycal α-epoxide is >20:1. The *dr* of the glycosylation product determined based on crude reaction mixture is 10:1. The desired product **6** was purified through a silica gel flash column (hexanes/acetone: from 20:1 to 4:1) as colorless oil (43.3 mg, 71% yield).

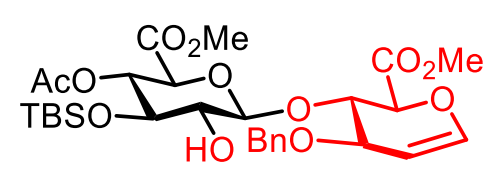

6

Methyl 3-*O*-benzyl-4-*O*-(methyl 4-*O*-acetyl-3-*O*-*tert*-butyldimethylsilyl-β-D-glucopyranosyluronate)-D-glucuronal (**6**): IR ν_{max} (neat)/cm^{-1}: 3521 (w), 2953 (w), 2928 (w), 2856 (w), 1751 (s), 1232 (s), 1104 (s), 1032 (s), 838 (s); ^{1}H NMR (400 MHz, $CDCl_3$) δ 7.35 – 7.21 (m, 5H), 6.59 (d, *J* = 6.3 Hz, 1H), 5.03 – 4.93 (m, 2H), 4.75 (d, *J* = 2.9 Hz, 1H), 4.55 – 4.39 (m, 4H), 4.00 – 3.95 (m, 1H), 3.88 (d, *J* = 10.0 Hz, 1H), 3.72 (t, *J* = 9.0 Hz, 1H), 3.70 (s, 3H), 3.56 (s, 3H), 3.44 (td, *J* = 8.4, 2.8 Hz, 1H), 2.43 (d, *J* = 2.9 Hz, 1H), 2.05 (s, 3H), 0.84 (s, 9H), 0.10 (s, 3H), 0.06 (s, 3H); ^{13}C NMR (100 MHz, $CDCl_3$) δ 169.7, 168.1, 167.7, 144.9, 137.8, 128.3 (2C), 127.9 (2C), 127.7, 102.0, 98.8, 74.3, 73.9, 73.5, 72.9, 72.6, 71.8, 69.8, 68.1, 52.7, 52.3, 25.6 (3C), 20.8, 18.0, -4.4, -4.9; HRMS: m/z (ESI) calcd for $C_{29}H_{43}O_{12}Si^+$, $[M + H]^+$, 611.2518, found 611.2530. $^1J_{^{13}C1\text{–}H1}$ = 159.0 Hz.

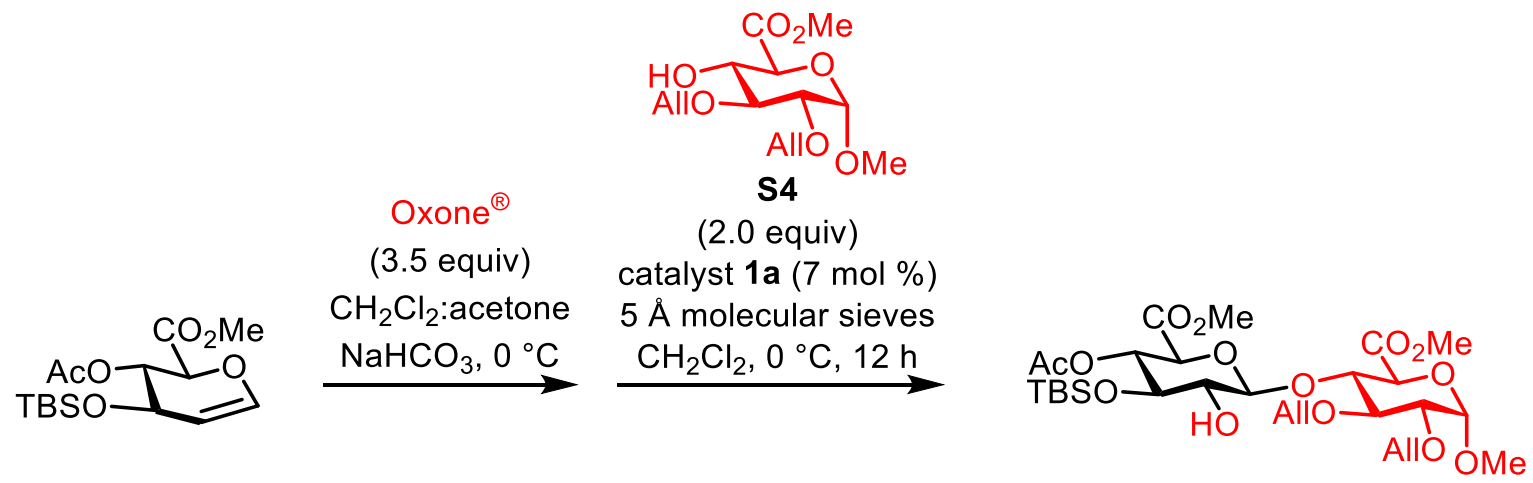

S4 was synthesized according to a literature procedure.[4]

The reaction was carried out on a 0.1 mmol scale by following **Procedure A** for epoxidation and **Procedure C** for the iron-catalyzed stereospecific glycosylation at 0 °C. The *dr* of the corresponding glycal α-epoxide is >20:1. The *dr* of the glycosylation product determined based on crude reaction mixture is >20:1. The desired product **7** was purified through a silica gel flash column (hexanes/acetone: from 20:1 to 4:1) as colorless oil (47.3 mg, 73% yield).

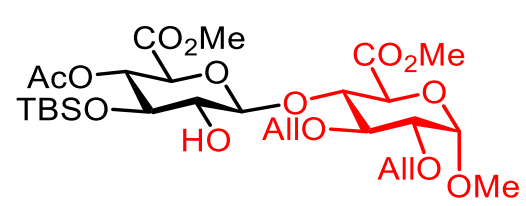

7

Methyl (methyl 4-*O*-acetyl-3-*O*-*tert*-butyldimethylsilyl-β-D-glucopyranosyluronate)-(1→4)-(methyl 2,3-di-allyl-α-D-glucopyranosid)uronate (**7**): IR ν_{max} (neat)/cm^{-1}: 3501 (w), 2953 (w), 2928 (w), 2856 (w), 1745 (s), 1369 (m), 1230 (m), 1033 (s), 837 (s); ^{1}H NMR (400 MHz, $CDCl_3$) δ 5.95 – 5.81 (m, 2H), 5.30 – 5.15 (m, 3H), 5.09 (d, *J* = 10.2 Hz, 1H), 4.92 (t, *J* = 9.6 Hz, 1H), 4.78 (d, *J* = 3.5 Hz, 1H), 4.51 (d, *J* = 7.7 Hz, 1H), 4.39 – 4.25 (m, 2H), 4.21 – 4.15 (m, 2H), 4.10 (dd, *J* = 12.8, 6.4 Hz, 1H), 3.89 (t, *J* = 9.2 Hz, 1H), 3.85 – 3.77 (m, 2H), 3.82 (s, 3H), 3.75 – 3.63 (m, 1H), 3.69 (s, 3H), 3.45 (s, 3H), 3.40 (dd, *J* = 9.4, 3.4 Hz, 1H), 3.37 – 3.27 (m, 2H), 2.04 (s, 3H), 0.84 (s, 9H), 0.12 (s, 3H), 0.06 (s, 3H); ^{13}C NMR (100 MHz, $CDCl_3$) δ 170.6, 169.8, 167.8, 135.2, 134.5, 118.1, 115.5, 104.0, 98.6, 79.5, 79.4, 78.9, 74.6, 74.4, 74.0, 73.1, 72.8, 71.9, 69.2, 55.7, 53.0, 52.5, 25.7 (3C), 20.9, 18.1, -4.3, -5.0; HRMS: m/z (ESI) calcd for $C_{29}H_{49}O_{14}Si^+$, [M + H]$^+$, 649.2886, found 649.2901. $^1J_{^{13}C1-H1}$ = 168.2 Hz, 162.3 Hz.

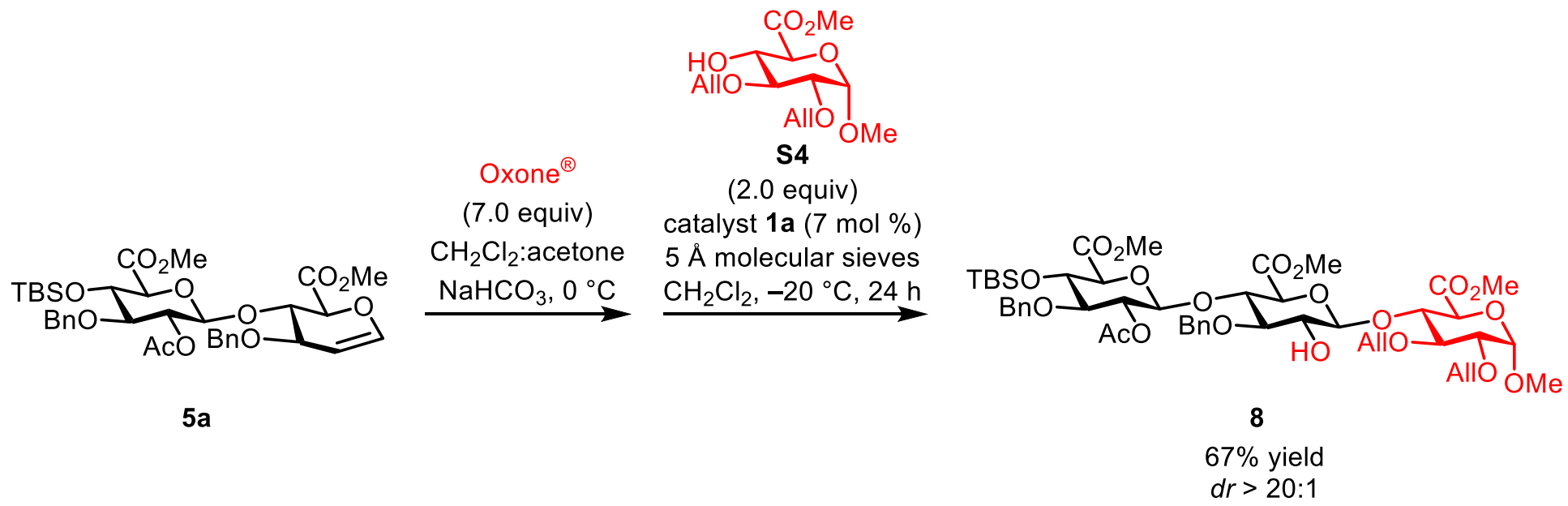

The reaction was carried out on a 0.05 mmol scale by following **Procedure A** for epoxidation using 7.0 equiv of Oxone and **Procedure C** for the iron-catalyzed stereospecific glycosylation at –20 °C. The *dr* of the corresponding glycal α-epoxide is >20:1. The *dr* of the glycosylation product determined based on crude reaction mixture is >20:1. The desired product **8** was purified through a silica gel flash column (hexanes/acetone: from 20:1 to 3:1) as white foam (34.1 mg, 67% yield).

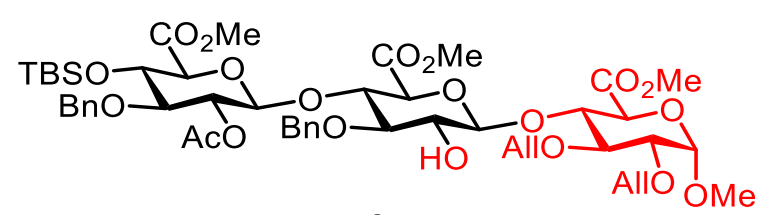

8

Methyl (methyl 2-*O*-acetyl-3-*O*-benzyl-4-*O*-*tert*-butyldimethylsilyl-β-D-glucopyranosyluronate)-(1→4)-(methyl 3-*O*-benzyl-β-D-glucopyranosyluronate)-(1→4)-(methyl 2,3-di-allyl-α-D-glucopyranosid)uronate (**8**): IR ν_{max} (neat)/cm^{-1}: 3520 (w), 2953 (w), 2928 (w), 2855 (w), 1749 (s), 1361 (m), 1220 (m), 1040 (s), 838 (m); ^{1}H NMR (400 MHz, $CDCl_3$) δ 7.40 (d, *J* = 7.0 Hz, 2H), 7.38 – 7.21 (m, 8H), 5.94 – 5.81 (m, 2H), 5.31 – 5.14 (m, 3H), 5.09 (dd, *J* = 10.6, 1.9 Hz, 1H), 4.96 (t, *J* = 8.4 Hz, 1H), 4.87 (ABq, $\Delta\nu_{AB}$ = 36.2 Hz, *J* = 11.4 Hz, 2H), 4.77 (d, *J* = 3.5 Hz, 1H), 4.71 – 4.64 (m, 2H), 4.61 (d, *J* = 11.6 Hz, 1H), 4.49 (d, *J* = 7.7 Hz, 1H), 4.30 – 4.23 (m, 2H), 4.22 – 4.06 (m, 3H), 4.04 – 3.93 (m, 2H), 3.87 (dd, *J* = 9.9, 8.6 Hz, 1H), 3.82 – 3.71 (m, 3H), 3.74 (s, 6H), 3.62 (s, 3H), 3.51 (t, *J* = 8.8 Hz, 1H), 3.48 – 3.35 (m, 3H), 3.43 (s, 3H), 3.08 (d, *J* = 2.1 Hz, 1H), 1.90 (s, 3H), 0.82 (s, 9H), -0.04 (s, 3H), -0.07 (s, 3H); ^{13}C NMR (100 MHz, $CDCl_3$) δ 170.3, 169.6, 168.4, 168.3, 139.0, 138.0, 135.3, 134.5, 128.3 (two peaks overlapped, 4C), 127.7 (2C), 127.5, 127.4, 127.2 (2C), 118.0, 115.8, 103.8, 100.6, 98.6, 82.4, 81.7, 79.3, 79.1, 78.8, 78.2, 76.7, 74.70, 74.66, 74.5, 74.1, 74.0, 73.1, 72.8, 72.1, 69.4, 55.6,

52.9, 52.4, 52.2, 25.7 (3C), 20.8, 17.8, -4.2, -5.3; HRMS: m/z (ESI) calcd for $C_{50}H_{71}O_{20}Si^+$, [M + H]$^+$, 1019.4302, found 1019.4327. $^{1}J_{^{13}C1-H1}$ = 170.4 Hz, 162.8 Hz, 162.6 Hz.

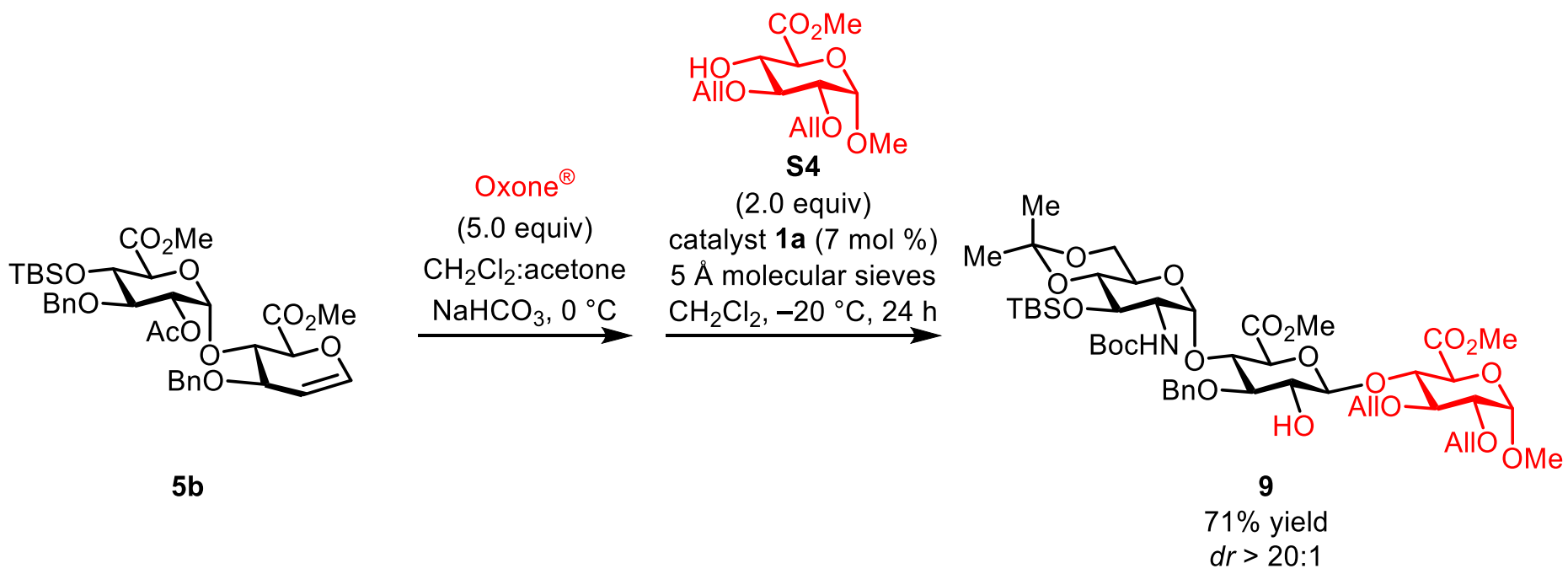

The reaction was carried out on a 0.05 mmol scale by following **Procedure A** for epoxidation using 5.0 equiv of Oxone and **Procedure C** for the iron-catalyzed stereospecific glycosylation at –20 °C. The *dr* of the corresponding glycal α-epoxide is >20:1. The *dr* of the glycosylation product determined based on crude reaction mixture is >20:1. The desired product **9** was purified through a silica gel flash column (hexanes/acetone: from 20:1 to 3:1) as white foam (36.1 mg, 71% yield).

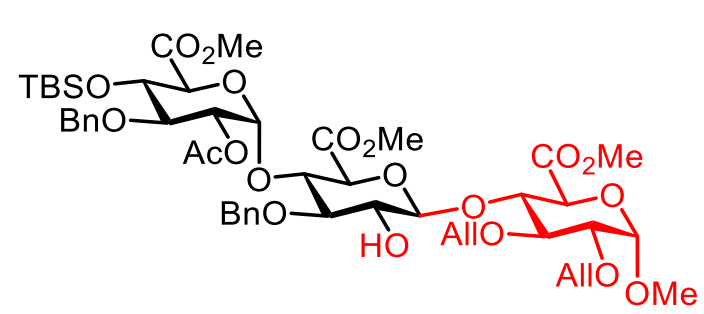

Methyl (methyl 2-*O*-acetyl-3-*O*-benzyl-4-*O*-*tert*-butyldimethylsilyl-α-D-glucopyranosyluronate)-(1→4)-(methyl 3-*O*-benzyl-β-D-glucopyranosyluronate)-(1→4)-(methyl 2,3-di-allyl-α-D-glucopyranosid)uronate (**9**): IR ν_{max} (neat)/cm^{-1}: 3521 (w), 2954 (w), 2926 (w), 2856 (w), 1751 (s), 1365 (m), 1230 (m), 1040 (s), 837 (m); ^{1}H NMR (400 MHz, $CDCl_3$) δ 7.33 – 7.20 (m, 10H), 5.98 – 5.83 (m, 2H), 5.54 (d, *J* = 3.7 Hz, 1H), 5.32 – 5.23 (m, 2H), 5.19 (dd, *J* = 10.4, 1.5 Hz, 1H), 5.14 (dd, *J* = 10.5, 1.9 Hz, 1H), 5.01 – 4.89 (m, 2H), 4.80 – 4.74 (m, 2H), 4.72 – 4.64 (m, 2H), 4.56 (d, *J* = 7.5 Hz, 1H), 4.32 – 4.27 (m, 2H), 4.22 – 4.15 (m, 2H), 4.14 – 4.08 (m, 1H), 4.03 (dd, *J* = 9.6, 8.1 Hz, 1H), 3.96 (ABq, $\Delta\nu_{AB}$ = 7.2 Hz, *J* = 9.4 Hz,

2H), 3.92 – 3.83 (m, 2H), 3.82 – 3.77 (m, 1H), 3.79 (s, 3H), 3.78 (s, 3H), 3.76 – 3.68 (m, 1H), 3.75 (s, 3H), 3.57 (t, J = 8.6 Hz, 1H), 3.52 (td, J = 8.3, 2.1 Hz, 1H), 3.45 (s, 3H), 3.43 – 3.38 (m, 2H), 1.72 (s, 3H), 0.84 (s, 9H), -0.03 (s, 3H), -0.06 (s, 3H); ^{13}C NMR (100 MHz, $CDCl_3$) δ 170.6, 169.9, 169.3, 168.0, 138.4, 138.1, 135.2, 134.5, 128.34 (2C), 128.25 (2C), 127.60, 127.57 (2C), 127.3, 126.8 (2C), 118.1, 115.9, 103.9, 98.6, 96.4, 83.0, 79.4, 79.2, 79.1, 78.9, 75.4, 75.1, 75.0, 74.5, 74.2, 74.1, 73.0, 72.8, 72.3, 72.2, 69.3, 55.7, 53.0, 52.6, 52.2, 25.7 (3C), 20.6, 17.8, -4.1, -5.4; HRMS: m/z (ESI) calcd for $C_{50}H_{71}O_{20}Si^+$, $[M + H]^+$, 1019.4302, found 1019.4319. $^1J^{13}{}_{C1\text{–}H1}$ = 177.6 Hz, 170.3 Hz, 162.5 Hz.

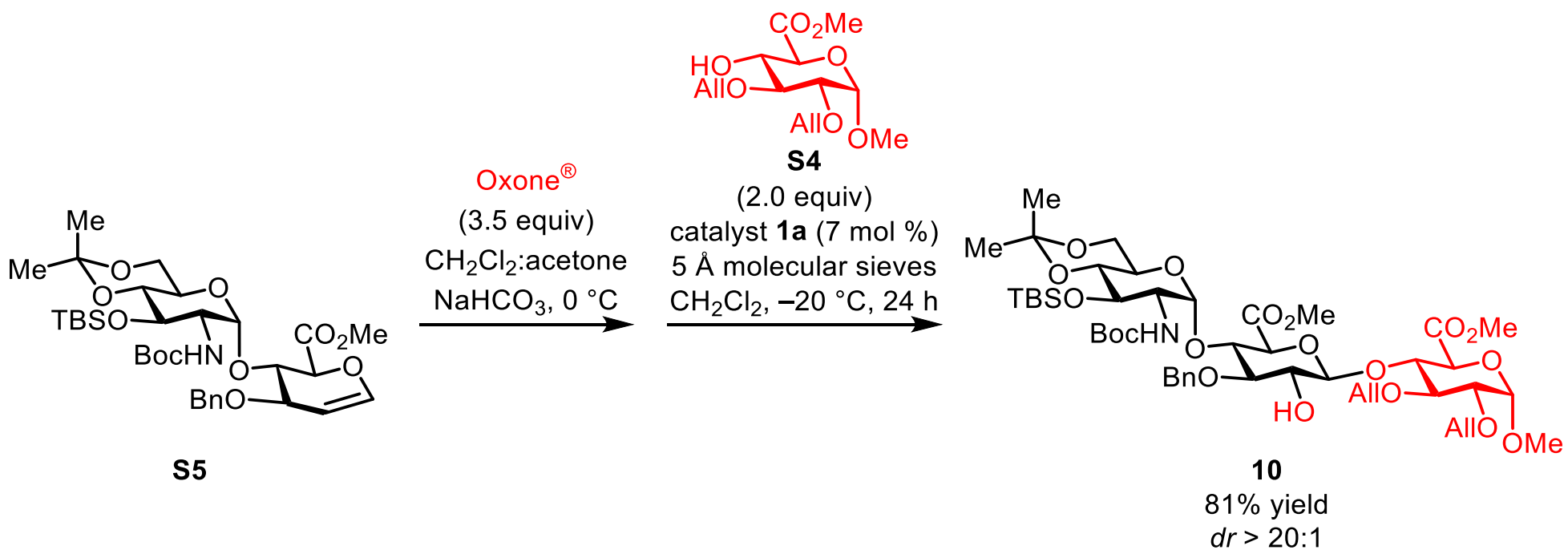

S5 was synthesized according to a literature procedure.[4, 7]

The reaction was carried out on a 0.1 mmol scale by following **Procedure A** for epoxidation and **Procedure C** for the iron-catalyzed stereospecific glycosylation at –20 °C. The *dr* of the corresponding glycal α-epoxide is >20:1. The *dr* of the glycosylation product determined based on crude reaction mixture is >20:1. The desired product **10** was purified through a silica gel flash column (hexanes/EtOAc: from 20:1 to 3:2) as white foam (80.8 mg, 81% yield).

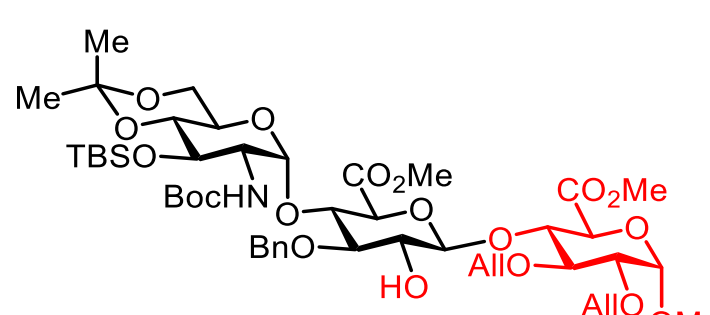

10

Methyl (2-*tert*-butoxycarbonylamino-3-*O*-*tert*-butyldimethylsilyl-4,6-*O*-isopropylidene-2-deoxy-α-D-glucopyranosyl)-(1→4)-(methyl 3-*O*-benzyl-β-D-glucopyranosyluronate)-(1→4)-(methyl 2,3-di-allyl-α-D-glucopyranosid)uronate (**10**): IR ν_{max} (neat)/cm^{-1}: 3455 (w), 2931 (w), 2361 (w), 1744 (s), 1370 (s), 1232 (s), 1026 (s), 987 (s), 874 (m); ^{1}H NMR (400 MHz, $CDCl_3$) δ 7.39 – 7.29 (m, 4H), 7.28 – 7.23 (m, 1H), 5.96 – 5.83 (m, 2H), 5.36 (d, *J* = 3.9 Hz, 1H), 5.31 – 5.23 (m, 2H), 5.19 (dd, *J* = 10.3, 1.4 Hz, 1H), 5.14 (dd, *J* = 10.5, 1.8 Hz, 1H), 5.01 – 4.88 (m, 2H), 4.78 (d, *J* = 3.4 Hz, 1H), 4.70 (d, *J* = 10.4 Hz, 1H), 4.55 (d, *J* = 7.6 Hz, 1H), 4.35 – 4.21 (m, 2H), 4.22 – 4.13 (m, 2H), 4.10 (dd, *J* = 12.7, 6.5 Hz, 1H), 4.02 – 3.93 (m, 1H), 3.94 – 3.82 (m, 3H), 3.82 – 3.72 (m, 3H), 3.80 (s, 3H), 3.77 (s, 3H), 3.63 (d, *J* = 10.8 Hz, 1H), 3.58 (d, *J* = 8.4 Hz, 1H), 3.54 – 3.46 (m, 3H), 3.44 (s, 3H), 3.40 (dd, *J* = 9.5, 3.5 Hz, 1H), 3.21 (td, *J* = 9.9, 5.1 Hz, 1H), 1.43 (s, 3H), 1.35 (s, 3H), 1.34 (s, 9H), 0.87 (s, 9H), 0.07 (s, 3H), 0.05 (s, 3H); ^{13}C NMR (100 MHz, $CDCl_3$) δ 170.8, 168.5, 155.2, 137.9, 135.3, 134.6, 128.7 (2C), 128.6 (2C), 128.0, 118.2, 116.1, 104.3, 99.4, 99.3, 98.7, 83.5, 79.7, 79.6, 79.3, 79.1, 75.2, 75.1, 74.8, 74.6, 74.2, 74.1, 72.9, 71.6, 69.4, 64.3, 62.0, 55.9, 55.5, 53.2, 52.7, 29.2, 28.6 (3C), 25.9 (3C), 19.0, 18.4, -3.9, -4.9; HRMS: m/z (ESI) calcd for $C_{48}H_{76}NO_{19}Si^+$, [M + H]$^+$, 998.4775, found 998.4797. $^1J_{^{13}C-^1H}$ = 176.1 Hz, 170.5 Hz, 163.4 Hz.

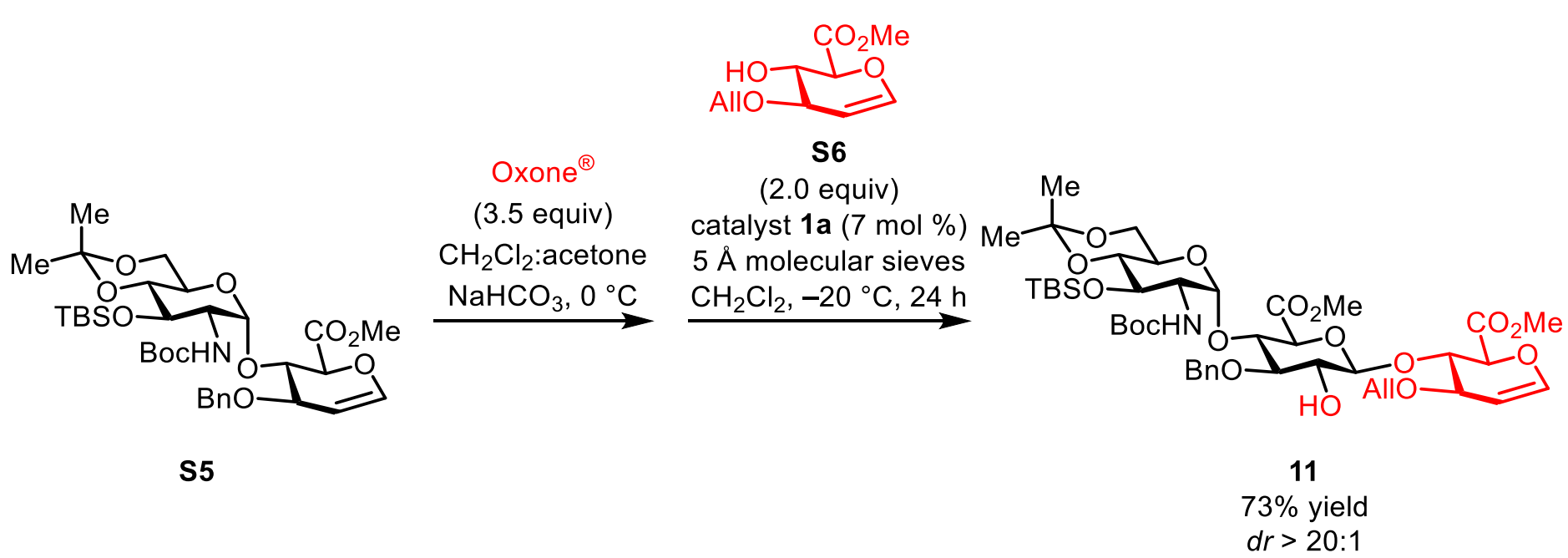

S6 was synthesized according to a literature procedure.[4]

The reaction was carried out on a 0.1 mmol scale by following **Procedure A** for epoxidation and **Procedure C** for the iron-catalyzed stereospecific glycosylation at –20 °C. The *dr* of the corresponding glycal α-epoxide is >20:1. The *dr* of the glycosylation product determined based

on crude reaction mixture is >20:1. The desired product **11** was purified through a silica gel flash column (hexanes/EtOAc: from 20:1 to 2:1) as white foam (66.3 mg, 73% yield).

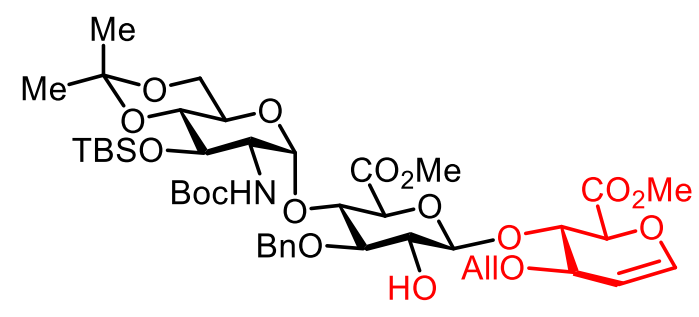

11

Methyl 3-*O*-allyl-4-*O*-[(2-*tert*-butoxycarbonylamino-3-*O*-*tert*-butyldimethylsilyl-4,6-*O*-isopropylidene-2-deoxy-α-D-glucopyranosyl)-(1→4)-(methyl 3-*O*-benzyl-β-D-glucopyranosyluronate)]-D-glucuronal (**11**): IR ν_{max} (neat)/cm^{-1}: 3454 (w), 2929 (w), 2360 (w), 1741 (s), 1367 (s), 1232 (s), 1026 (s), 987 (s), 874 (m); ^{1}H NMR (400 MHz, $CDCl_3$) δ 7.38 – 7.25 (m, 5H), 6.56 (d, *J* = 6.3 Hz, 1H), 5.82 (ddt, *J* = 17.2, 10.3, 5.6 Hz, 1H), 5.34 (d, *J* = 3.9 Hz, 1H), 5.22 (dd, *J* = 17.2, 1.7 Hz, 1H), 5.14 (dd, *J* = 10.4, 1.5 Hz, 1H), 4.99 – 4.90 (m, 2H), 4.81 (ABq, $\Delta\nu_{AB}$ = 54.4 Hz, *J* = 10.5 Hz, 2H), 4.71 (d, *J* = 3.4 Hz, 1H), 4.52 (d, *J* = 7.2 Hz, 1H), 4.41 – 4.34 (m, 1H), 4.02 (dd, *J* = 9.6, 8.1 Hz, 1H), 4.01 – 3.89 (m, 3H), 3.89 – 3.82 (m, 2H), 3.82 (s, 3H), 3.78 (dd, *J* = 10.6, 5.2 Hz, 1H), 3.73 (s, 3H), 3.68 – 3.53 (m, 3H), 3.52 (t, *J* = 9.1 Hz, 1H), 3.46 (t, *J* = 9.0 Hz, 1H), 3.24 (td, *J* = 10.0, 5.2 Hz, 1H), 2.58 – 2.46 (m, 1H), 1.44 (s, 3H), 1.36 (s, 3H), 1.35 (s, 9H), 0.87 (s, 9H), 0.08 (s, 3H), 0.06 (s, 3H); ^{13}C NMR (100 MHz, $CDCl_3$) δ 168.4, 168.2, 155.2, 144.9, 137.7, 134.5, 128.7 (2C), 128.6 (2C), 128.1, 117.4, 102.5, 99.5, 99.4, 99.1, 83.4, 79.8, 75.1, 75.0, 74.6, 74.4 (two peaks overlapped, 2C), 73.7, 72.7, 71.5, 69.0, 68.3, 64.4, 62.0, 55.5, 52.9, 52.6, 29.2, 28.6 (3C), 25.9 (3C), 19.0, 18.4, -3.9, -4.9; HRMS: m/z (ESI) calcd for $C_{44}H_{68}NO_{17}Si^{+}$, $[M + H]^{+}$, 910.4251, found 910.4265. $^{1}J_{^{13}C^{1}-^{1}H^{1}}$ = 177.1 Hz, 159.8 Hz.

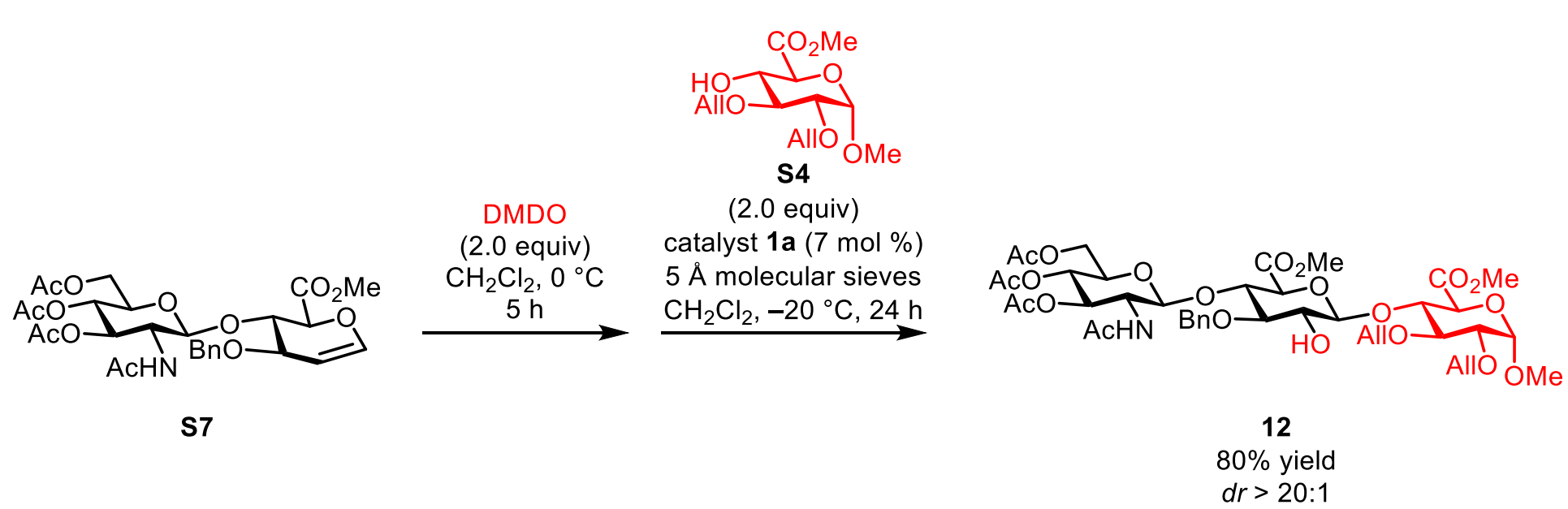

S7 was synthesized according to a literature procedure.[1]

The reaction was carried out on a 0.1 mmol scale by following **Procedure B** for epoxidation and **Procedure C** for the iron-catalyzed stereospecific glycosylation at –20 °C with the modifications: epoxidation was carried out with DMDO (0.06 M in acetone, 3.7 mL, 2.0 equiv) at 0 °C for 5 h. The *dr* of the corresponding glycal α-epoxide is >20:1. The *dr* of the glycosylation product determined based on crude reaction mixture is >20:1. The desired product **12** was purified through a silica gel flash column (hexanes/acetone: from 20:1 to 3:2) as white foam (72.9 mg, 80% yield).

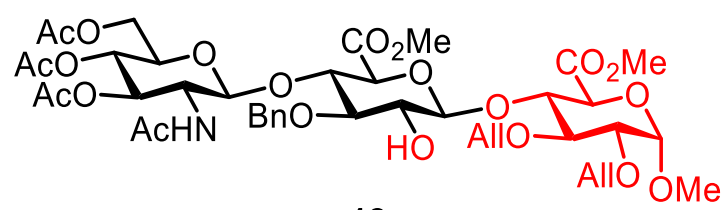

12

Methyl (2-acetamido-3,4,6-tri-*O*-acetyl-2-deoxy-β-D-glucopyranosyl)-(1→4)-(methyl 3-*O*-benzyl-β-D-glucopyranosyluronate)-(1→4)-(methyl 2,3-di-allyl-α-D-glucopyranosid)uronate (**12**): IR ν_{max} (neat)/cm^{-1}: 3476 (w), 3303 (w), 2956 (w), 1734 (s), 1665 (m), 1370 (m), 1220 (s), 1035 (s), 911 (m); ^{1}H NMR (400 MHz, $CDCl_3$) δ 7.38 – 7.21 (m, 5H), 5.93 – 5.81 (m, 2H), 5.65 (d, *J* = 9.0 Hz, 1H), 5.29 – 5.15 (m, 3H), 5.11 – 4.97 (m, 3H), 4.88 (ABq, $\Delta\nu_{AB}$ = 49.5 Hz, *J* = 11.6 Hz, 2H), 4.77 (d, *J* = 3.4 Hz, 1H), 4.57 (d, *J* = 8.4 Hz, 1H), 4.52 (d, *J* = 7.7 Hz, 1H), 4.36 – 4.23 (m, 2H), 4.23 – 4.06 (m, 4H), 4.00 (q, *J* = 9.1 Hz, 1H), 3.96 – 3.83 (m, 4H), 3.80 (s, 3H), 3.76 (t, *J* = 9.5 Hz, 1H), 3.75 (s, 3H), 3.55 – 3.37 (m, 4H), 3.43 (s, 3H), 3.19 (d, *J* = 2.0 Hz, 1H), 2.00 (s, 3H), 1.97 (s, 3H), 1.95 (s, 6H); ^{13}C NMR (100 MHz, $CDCl_3$) δ 170.9, 170.6, 170.33, 170.31, 169.4, 169.2, 138.8, 135.3, 134.4, 128.3 (2C), 127.4, 127.3 (2C), 118.1, 115.5, 103.7, 100.8, 98.5, 81.8, 79.4, 79.2, 78.8, 78.4, 74.8, 74.5, 73.91, 73.86, 73.1, 72.8, 71.9, 69.3, 68.0, 61.7, 55.6, 54.2, 52.9, 52.8, 23.2, 20.62, 20.59, 20.5; HRMS: m/z (ESI) calcd for $C_{42}H_{58}NO_{21}^+$, $[M + H]^+$, 912.3496, found 912.3522. $^1J_{^{13}C1-H1}$ = 170.6 Hz, 161.0 Hz, 162.6 Hz.

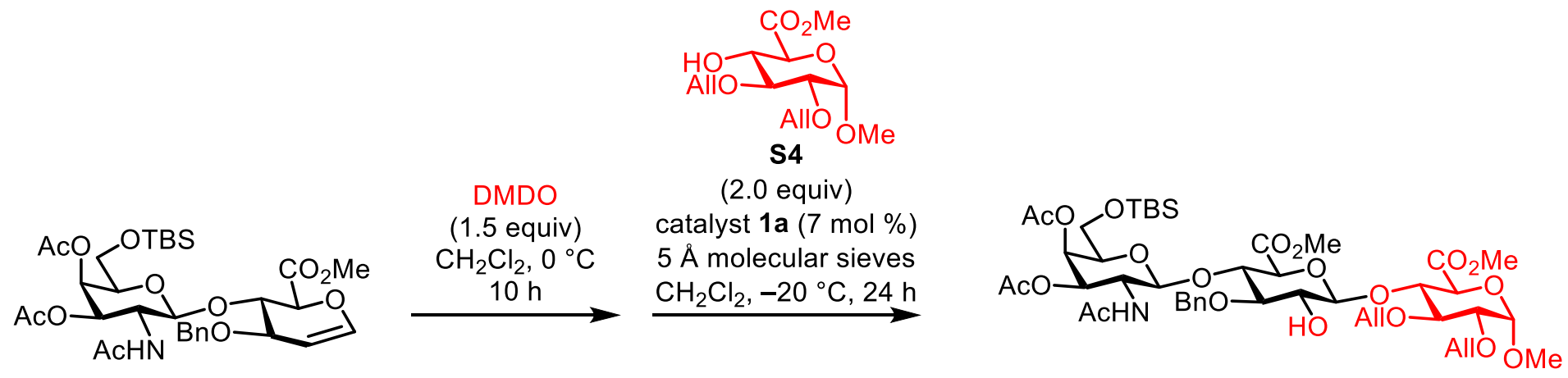

S8

13
71% yield
dr > 20:1

S8 was synthesized according to a literature procedure.[1]

The reaction was carried out on a 0.1 mmol scale by following **Procedure B** for epoxidation and **Procedure C** for the iron-catalyzed stereospecific glycosylation at –20 °C with the modifications: epoxidation was carried out with DMDO (0.06 M in acetone, 2.5 mL, 1.5 equiv) at 0 °C for 10 h. The *dr* of the corresponding glycal α-epoxide is >20:1. The *dr* of the glycosylation product determined based on crude reaction mixture is >20:1. The desired product **13** was purified through a silica gel flash column (hexanes/acetone: from 20:1 to 2:1) as white foam (70.1 mg, 71% yield).

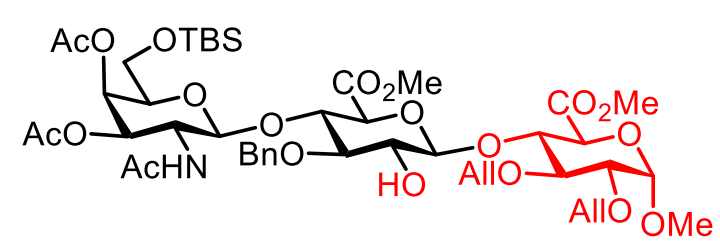

13

Methyl (2-acetamido-3,4-di-*O*-acetyl-6-*O*-*tert*-butyldimethylsilyl-2-deoxy-β-D-galactopyranosyl)-(1→4)-(methyl 3-*O*-benzyl-β-D-glucopyranosyluronate)-(1→4)-(methyl 2,3-di-allyl-α-D-glucopyranosid)uronate (**13**): IR ν_{max} (neat)/cm^{-1}: 3415 (w), 2954 (w), 2929 (w), 2857 (w), 1749 (s), 1362 (m), 1219 (s), 1039 (s), 837 (s); ^{1}H NMR (400 MHz, $CDCl_3$) δ 7.40 (d, *J* = 6.7 Hz, 2H), 7.31 (t, *J* = 7.3 Hz, 2H), 7.26 (t, *J* = 3.7 Hz, 1H), 5.95 – 5.80 (m, 2H), 5.49 (d, *J* = 9.2 Hz, 1H), 5.37 (d, *J* = 3.3 Hz, 1H), 5.32 – 5.16 (m, 3H), 5.09 (dd, *J* = 10.5, 1.8 Hz, 1H), 5.01 – 4.90 (m, 2H), 4.84 (d, *J* = 11.1 Hz, 1H), 4.78 (d, *J* = 3.5 Hz, 1H), 4.53 (d, *J* = 7.7 Hz, 1H), 4.49 (d, *J* = 8.4 Hz, 1H), 4.35 – 4.22 (m, 2H), 4.23 – 4.04 (m, 4H), 3.99 – 3.86 (m, 3H), 3.82 – 3.76 (m, 1H), 3.79 (s, 3H), 3.78 (s, 3H), 3.55 – 3.46 (m, 3H), 3.48 – 3.37 (m, 3H), 3.44 (s,

3H), 3.28 (d, J = 2.0 Hz, 1H), 2.09 (s, 3H), 1.98 (s, 3H), 1.97 (s, 3H), 0.81 (s, 9H), -0.06 (s, 3H), -0.07 (s, 3H); ^{13}C NMR (100 MHz, $CDCl_3$) δ 170.7, 170.5, 170.4, 169.9, 169.3, 138.8, 135.3, 134.4, 128.2 (2C), 127.5 (2C), 127.4, 118.1, 115.6, 103.8, 101.1, 98.6, 81.9, 79.4, 79.1, 78.9, 77.6, 75.0, 74.7, 74.0, 73.9, 73.4, 72.8, 71.2, 69.3, 66.2, 59.9, 55.7, 53.0, 52.7, 51.2, 25.7 (3C), 23.4, 20.8, 20.7, 18.0, -5.7, -5.8; HRMS: m/z (ESI) calcd for $C_{46}H_{70}NO_{20}Si^+$, $[M + H]^+$, 984.4255, found 984.4266. $^1J_{^{13}C1-H1}$ = 170.3 Hz, 162.2 Hz, 162.7 Hz.

D. **Iron-Catalyzed Stereospecific Glycosylation with Glycal Epoxides for PGA Tetrasaccharide Synthesis**

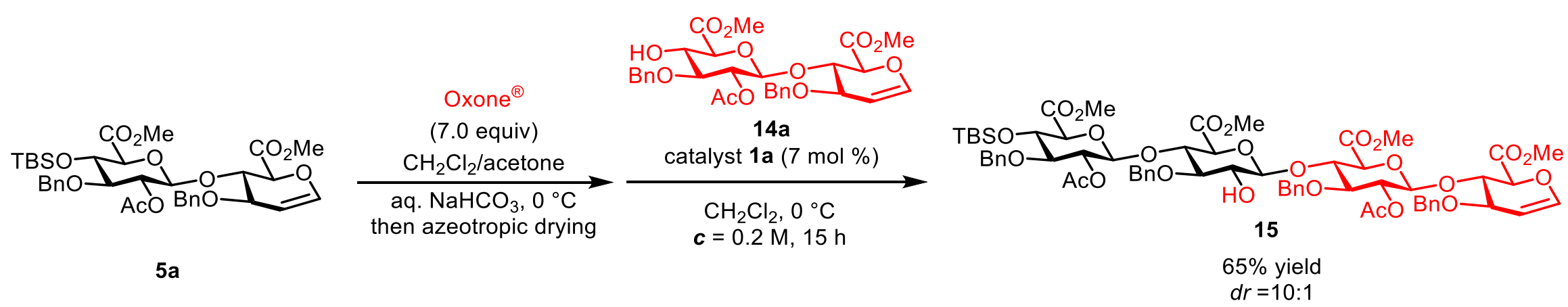

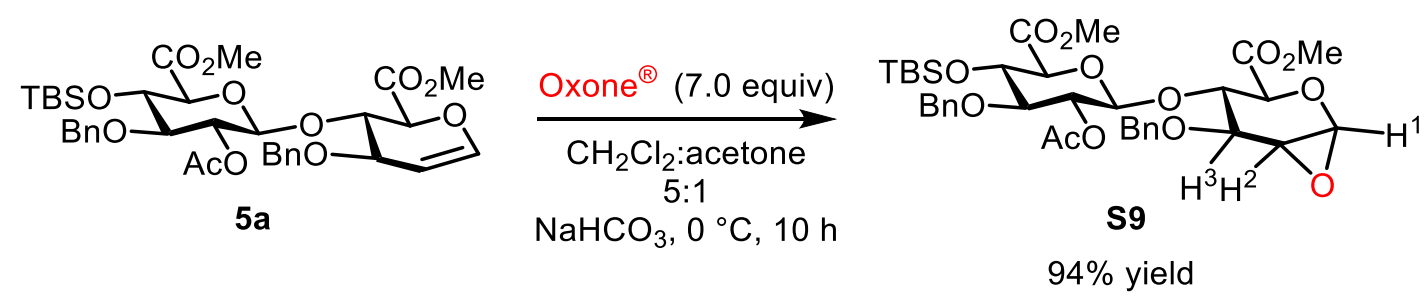

To a 25 mL flask equipped with a stir bar at 0 °C were added a glycal **5a** (0.7 g, 1.0 mmol, 1.0 equiv) in CH_2Cl_2/acetone mixture (v/v: 5:1, 10 mL) and saturated aqueous $NaHCO_3$ solution (24 mL), followed by addition of Oxone® ($KHSO_5 \cdot 0.5KHSO_4 \cdot 0.5K_2SO_4$) (2.35 g, 3.5 mmol, 3.5 equiv) in H_2O (11 mL) dropwise. After stirring vigorously at 0 °C for 5 h, the conversion was determined as 50% by ^{1}H NMR analysis. Additional Oxone® ($KHSO_5 \cdot 0.5KHSO_4 \cdot 0.5K_2SO_4$) (2.35 g, 3.5 mmol, 3.5 equiv) in H_2O (5 mL) was added dropwise and stirred at 0 °C for another 5 h. The reaction mixture was extracted with CH_2Cl_2 (20 mL × 3). The combined organic phase was dried over anhydrous Na_2SO_4 and concentrated *in vacuo*. The residue was further azeotropically dried with anhydrous toluene (1.0 mL × 3). The obtained glycal epoxide **S9** was assayed by ^{1}H NMR to get the diastereomeric ratio (*dr* > 20:1) and directly used in the next step.

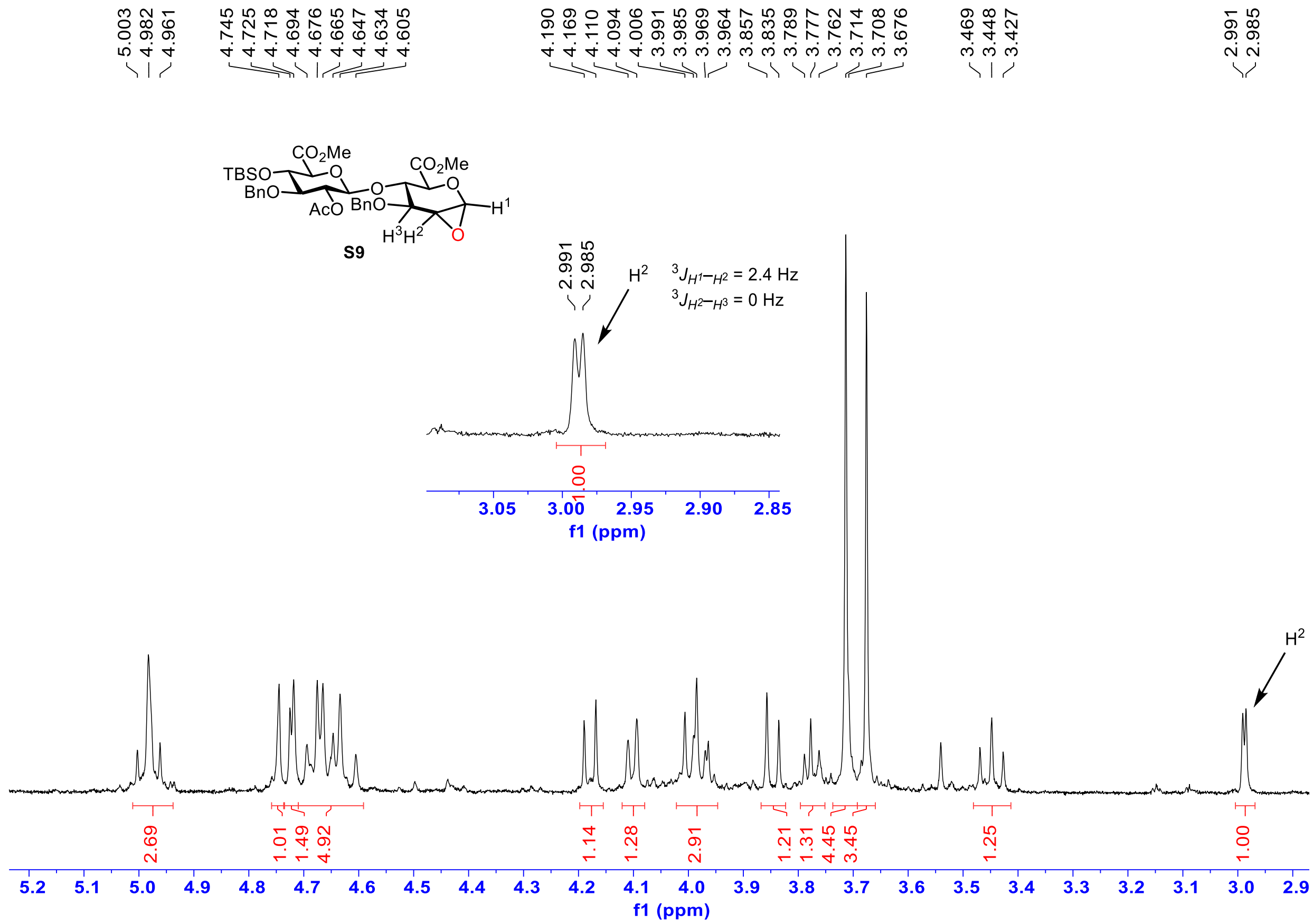

Figure S4. Stereochemistry Determination of Glycal Epoxide **S9**.

The stereochemistry of the glycal epoxide **S9** was determined by ^{1}H NMR analysis. It is known that the C(2)–H chemical shift of a glucal α-epoxide is around 3.0 ppm, whereas that of a glucal β-epoxide is around 3.3 ppm.[3]

Additionally, the $^3J_{H1-H2}$ value for a typical glucal α-epoxide is around 2.4 Hz, reflecting the dihedral angle ($\Phi_{1,2}$) that approaches 0°, whereas the $^3J_{H2-H3}$ value is < 0.5 Hz because the dihedral angle ($\Phi_{2,3}$) approaches 90°, characteristic of a *trans*-relationship between H^2 and H^3 that is pseudo-axial.[3] As a result, the H^2 signal of a glucal α-epoxide appears as a doublet.

In contrast, while a glucal β-epoxide retains a similar $\Phi_{1,2}$ and thus a comparable $^{3}J_{H1-H2}$, its $\Phi_{2,3}$ is around 45°, leading to a $^{3}J_{H2-H3}$ around 1.7 Hz.[3] This causes the H^{2} signal to appear as a doublet of doublets (though often observed as a triplet due to the broadening line width).

Based on these data, we have assigned glycal epoxide **S9** as an α-epoxide (*dr* > 20:1) with $^{3}J_{H1-H2}$ = 2.4 Hz and $^{3}J_{H2-H3}$ = 0 Hz. This assignment was further corroborated by the stereochemistry analysis of the glycosylation product **15**.

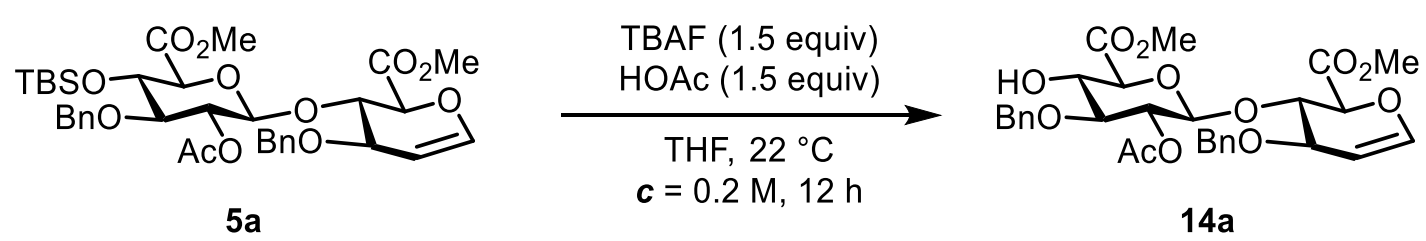

To a 50 mL round bottom flask equipped with a stir bar were added **5a** (1.0 g, 1.43 mmol, 1.0 equiv) and THF (3.0 mL). The flask was cooled to 0 °C, and a premixed solution of TBAF (1.0 M in THF, 2.14 mL, 2.14 mmol, 1.5 equiv) and AcOH (1.0 M in THF, 2.14 mL, 2.14 mmol, 1.5 equiv) was added. The reaction mixture was stirred for 12 h at room temperature, with progress monitored by TLC until completion. The reaction mixture was diluted with EtOAc (5 mL) and then quenched with saturated aqueous NH_4Cl solution (10 mL). The organic phase was separated from the aqueous phase, which was extracted with EtOAc (10 mL × 4). The combined organic phase was washed with brine (15 mL) and dried over Na_2SO_4. The desired product **14a** was isolated through column chromatography (hexanes/acetone: from 20:1 to 2:1) as white foam (0.75 g, 90% yield).

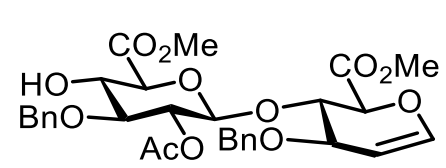

14a

Methyl 3-*O*-benzyl-4-*O*-(methyl 2-*O*-acetyl-3-*O*-benzyl-β-D-glucopyranosyluronate)-D-glucuronal (**14a**): IR ν_{max} (neat)/cm^{-1}: 3031 (w), 2953 (w), 2888 (w), 1746 (s), 1708 (s), 1362 (m), 1218 (s), 1059 (s), 698 (s); ^{1}H NMR (400 MHz, $CDCl_3$) δ 7.38 – 7.27 (m, 10H), 6.54 (d, *J* = 6.3 Hz, 1H), 4.99 – 4.93 (m, 2H), 4.76 (ABq, $\Delta\nu_{AB}$ = 30.5 Hz, *J* = 11.8 Hz, 2H), 4.68 – 4.62 (m,

2H), 4.53 (d, J = 11.4 Hz, 1H), 4.48 – 4.42 (m, 2H), 4.00 – 3.94 (m, 1H), 3.92 (ddd, J = 5.3, 2.7, 1.3 Hz, 1H), 3.88 (d, J = 9.8 Hz, 1H), 3.81 (s, 3H), 3.56 (t, J = 9.1 Hz, 1H), 3.55 (s, 3H), 3.08 (s, 1H), 1.98 (s, 3H); ^{13}C NMR (100 MHz, $CDCl_3$) δ 169.3, 169.2, 168.2, 144.8, 138.0, 137.8, 128.4 (2C), 128.3 (2C), 127.91 (2C), 127.86 (2C), 127.8, 127.6, 99.9, 98.4, 80.7, 74.4, 74.3, 73.0, 72.2, 71.8, 71.7, 69.6, 68.0, 52.8, 52.2, 20.7; HRMS: m/z (ESI) calcd for $C_{30}H_{35}O_{12}^+$, $[M + H]^+$, 587.2123, found 587.2137. $^1J_{^{13}C1–H1}$ = 162.5 Hz.

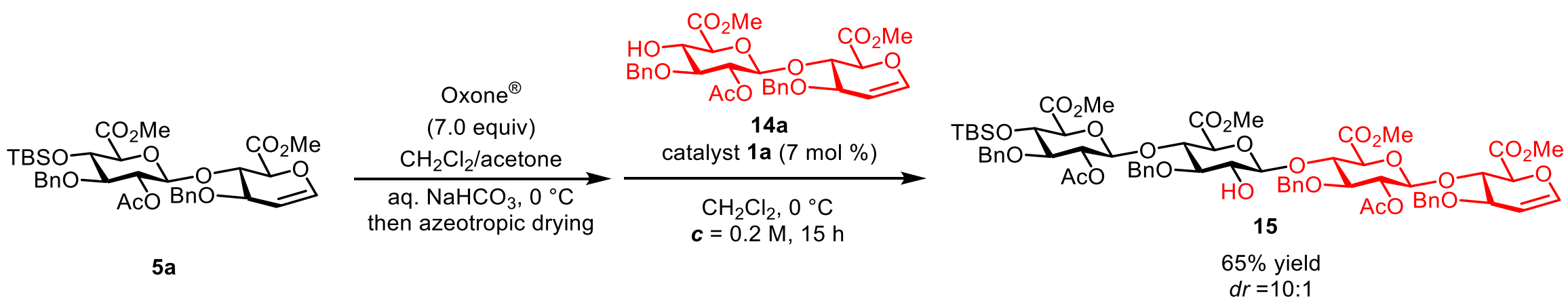

To a flame-dried sealable 50 mL round bottom flask equipped with a stir bar were added glycosyl acceptor **14a** (1.18 g, 2.0 mmol, 2.0 equiv), the aforementioned glycal epoxide **S9** (0.72 g, 1.0 mmol, 1.0 equiv), and freshly activated 5 Å molecular sieves, powder (*ca.* 1.0 g). After the flask was evacuated and backfilled with N_2, anhydrous CH_2Cl_2 (4.0 mL) was added. The iron(III) porphyrin catalyst **1a** (0.07 mmol, 7 mol %) solution in anhydrous CH_2Cl_2 (1.0 mL) was added to the flask and the resulting mixture was stirred at −40 °C for 5 min. The reaction mixture was kept at 0 °C for 15 h and then quenched with imidazole (70 mg) in CH_2Cl_2 (10 mL) at the same temperature. The mixture was filtered through a pad of Celite® silica gel and eluted with CH_2Cl_2 (20 mL). The organic layer was then concentrated *in vacuo* and *dr* of the glycosylation product determined based on crude reaction mixture is 10:1. The residue was purified through a silica gel flash column (hexanes/EtOAc: from 100:1 to 3:2) to afford the desired glycosylation product **15** (846.3 mg, 65% yield).

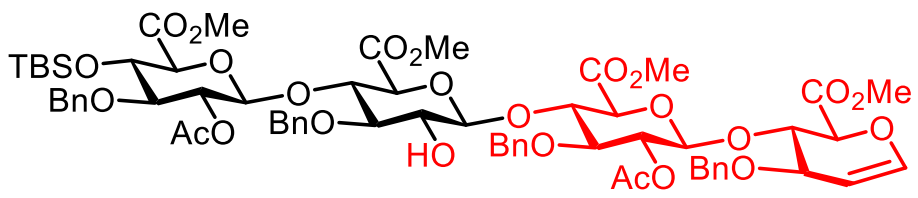

15

Methyl 3-*O*-benzyl-4-*O*-(methyl 2-*O*-acetyl-3-*O*-benzyl-4-*O*-*tert*-butyldimethylsilyl-β-D-glucopyranosyluronate)-(1→4)-(methyl 3-*O*-benzyl-β-D-glucopyranosyluronate)-(1→4)-(methyl 2-*O*-acetyl-3-*O*-benzyl-β-D-glucopyranosyluronate)-D-glucuronal (**15**): IR ν_{max} (neat)/cm^{-1}: 2953 (w), 2928 (w), 2888 (w), 2856 (w), 1747 (s), 1220 (s), 1066 (s), 1039 (s), 699 (m); ^{1}H NMR (400 MHz, $CDCl_3$) δ 7.42 – 7.19 (m, 20H), 6.52 (d, *J* = 6.3 Hz, 1H), 5.00 – 4.88 (m, 4H), 4.81 (d, *J* = 11.9 Hz, 1H), 4.77 – 4.65 (m, 2H), 4.64 – 4.57 (m, 4H), 4.54 – 4.45 (m, 2H), 4.44 – 4.38 (m, 3H), 4.09 (dd, *J* = 9.6, 8.6 Hz, 1H), 4.06 – 3.93 (m, 3H), 3.86 (ddd, *J* = 5.5, 2.7, 1.4 Hz, 1H), 3.82 (d, *J* = 8.8 Hz, 1H), 3.74 (d, *J* = 10.0 Hz, 1H), 3.73 (s, 3H), 3.66 – 3.60 (m, 1H), 3.63 (s, 3H), 3.59 (s, 3H), 3.53 (s, 3H), 3.51 – 3.39 (m, 3H), 2.55 (d, *J* = 1.5 Hz, 1H), 1.87 (s, 3H), 1.86 (s, 3H), 0.83 (s, 9H), -0.03 (s, 3H), -0.06 (s, 3H); ^{13}C NMR (100 MHz, $CDCl_3$) δ 169.5, 169.1, 168.6, 168.31, 168.29, 168.1, 144.8, 138.8, 138.3, 138.0, 137.8, 128.4 (2C), 128.28 (2C), 128.26 (2C), 128.2 (2C), 128.0 (2C), 127.9 (2C), 127.7 (two peaks overlapped, 3C), 127.54, 127.49, 127.45, 127.2 (2C), 103.5, 100.5, 99.8, 98.4, 82.4, 81.6, 79.9, 79.2, 78.0, 76.7, 74.83, 74.77, 74.5, 74.3, 74.2, 73.5, 73.0, 72.8, 72.1 (two peaks overlapped, 2C), 71.8, 69.6, 67.9, 53.0, 52.5, 52.2 (two peaks overlapped, 2C), 25.7 (3C), 20.7, 20.6, 17.8, -4.1, -5.3; HRMS: m/z (ESI) calcd for $C_{66}H_{83}O_{25}Si^{+}$, $[M + H]^{+}$, 1303.4987, found 1303.4999. $^{1}J^{13}_{C1-H1}$ = 162.7 Hz, 162.7 Hz, 162.4 Hz.

Stereochemistry Determination of Glycosylation Product 15

The stereochemistry of newly formed anomeric center (C1) of **15** was determined by measuring $^{1}J^{13}_{C1-H1}$ (162.4 Hz) through un-decoupled HSQC experiments. The C2 stereochemistry was determined by measuring $^{3}J_{H1-H2}$ (7.7 Hz).

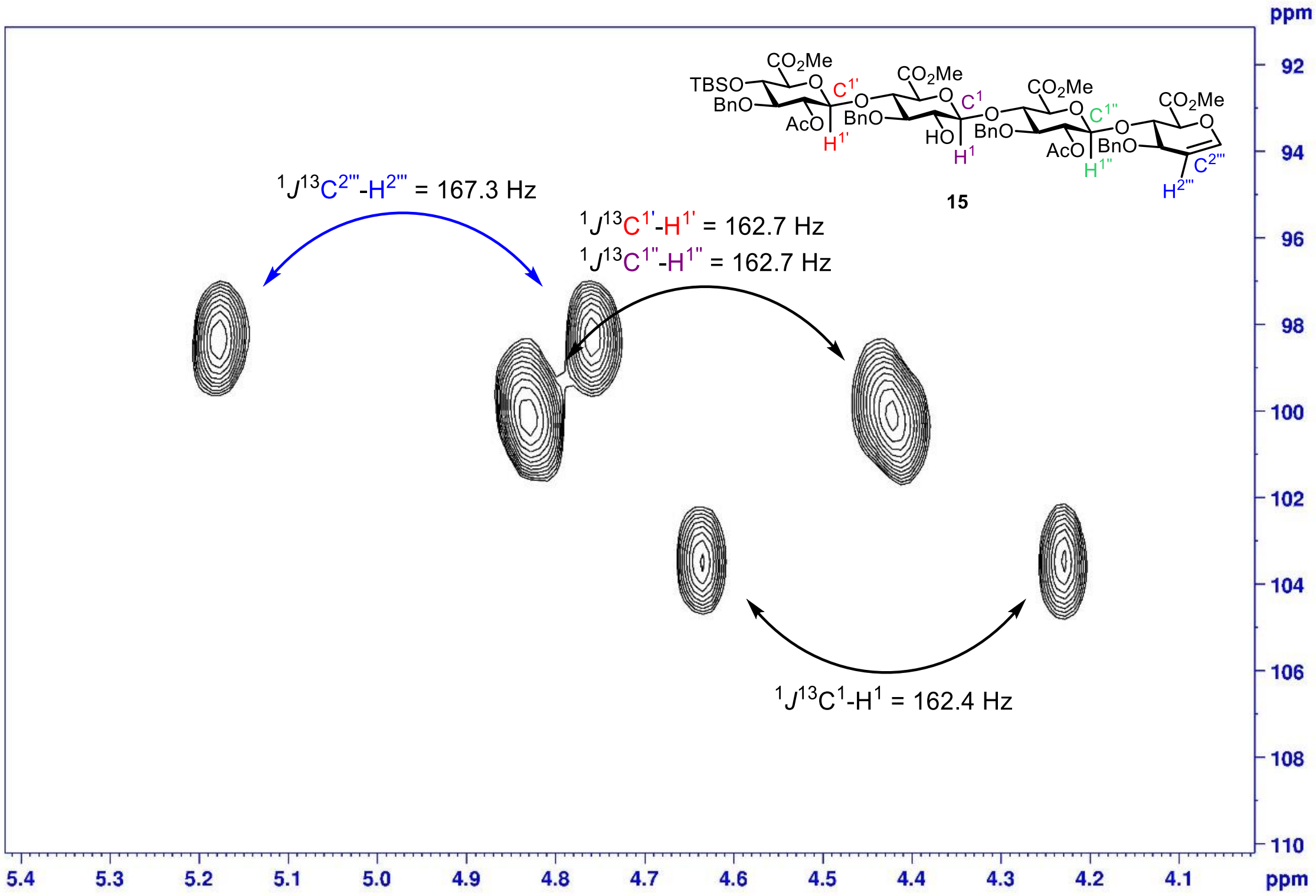

Figure S5. Un-decoupled HSQC Analysis to Determine Stereochemistry of **15**.

A $^1J^{13}{}_{C1-H1}$ value of 162.3 Hz suggested that the newly formed glycosidic bond is in equatorial position. The $^3J_{H1-H2}$ value of 7.6 Hz suggested that the H2 is in axial position and corroborated the exclusive formation of the α-epoxide in glycal epoxidation.

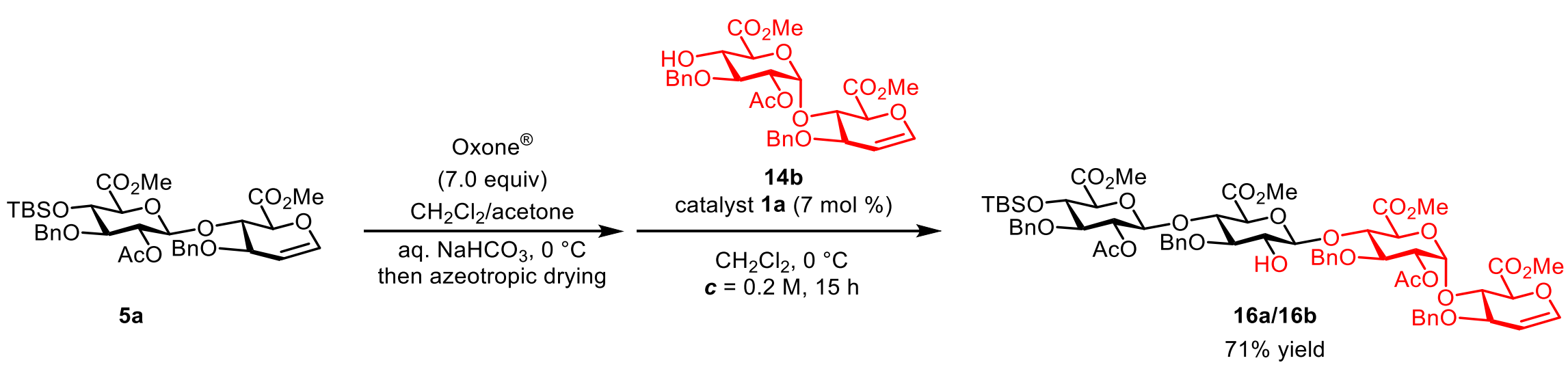

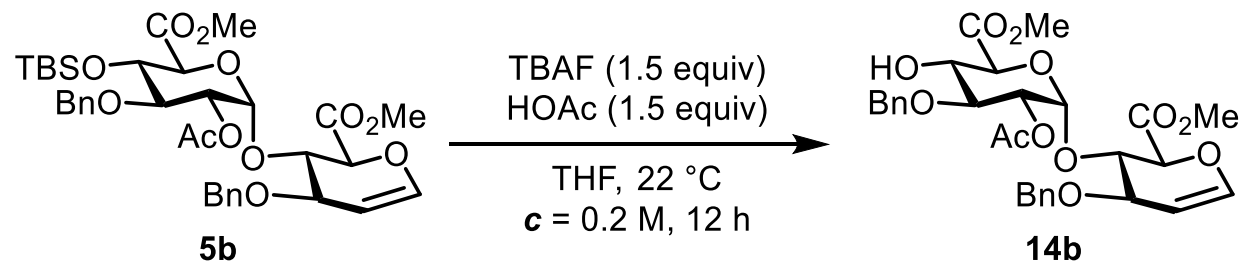

To a 50 mL round bottom flask equipped with a stir bar were added **5b** (0.50 g, 0.71 mmol, 1.0 equiv) and THF (4.0 mL). The flask was cooled to 0 °C, and a premixed solution of TBAF (1.07 mL, 1.07 mmol, 1.5 equiv) and AcOH (61 μL, 1.5 mmol, 1.5 equiv) was added. The reaction mixture was stirred for 12 h at room temperature, with progress monitored by TLC until completion. The reaction mixture was diluted with EtOAc (5 mL) and then quenched with saturated aqueous NH_4Cl solution (10 mL). The organic phase was separated from the aqueous phase, which was extracted with EtOAc (10 mL × 4). The combined organic phase was washed with brine (15 mL) and dried over Na_2SO_4. The desired product **14b** was isolated through column chromatography (hexanes/acetone: from 20:1 to 2:1) as white foam (378.6 mg, 91% yield).

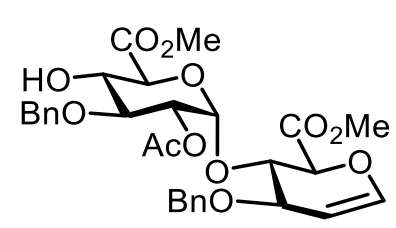

14b

Methyl 3-*O*-benzyl-4-*O*-(methyl 2-*O*-acetyl-3-*O*-benzyl-α-D-glucopyranosyluronate)-D-glucuronal (**14b**): IR ν_{max} (neat)/cm^{-1}: 3030 (w), 2955 (w), 2891 (w), 1743 (s), 1709 (s), 1366 (m), 1221 (s), 1057 (s), 698 (s); ^{1}H NMR (400 MHz, $CDCl_3$) δ 7.39 – 7.23 (m, 10H), 6.66 (d, *J* = 6.3 Hz, 1H), 5.42 (d, *J* = 3.8 Hz, 1H), 5.00 (ddd, *J* = 6.6, 5.3, 1.6 Hz, 1H), 4.96 (dd, *J* = 2.7, 1.4 Hz, 1H), 4.84 (d, *J* = 11.6 Hz, 1H), 4.79 – 4.71 (m, 2H), 4.46 (ABq, $\Delta\nu_{AB}$ = 29.3 Hz, *J* = 11.4 Hz, 2H), 4.38 (q, *J* = 2.2 Hz, 1H), 4.31 (d, *J* = 9.6 Hz, 1H), 3.97 – 3.86 (m, 1H), 3.83 (t, *J* = 9.4 Hz, 1H), 3.82 (s, 3H), 3.72 (dt, *J* = 5.2, 1.9 Hz, 1H), 3.55 (s, 3H), 3.09 (brs, 1H), 1.97 (s, 3H); ^{13}C NMR (100 MHz, $CDCl_3$) δ 170.4, 170.2, 167.9, 145.3, 138.4, 137.5, 128.4 (2C), 128.3 (2C), 127.9 (2C), 127.8, 127.7, 127.5 (2C), 98.2, 97.1, 78.4, 75.2, 73.9, 73.4, 72.4, 71.8, 70.7, 69.4, 66.7, 52.9, 52.1, 20.7; HRMS: m/z (ESI) calcd for $C_{30}H_{35}O_{12}^+$, $[M + H]^+$, 587.2123, found 587.2141. $^1J_{^{13}C1\text{–}H1}$ = 170.8 Hz.

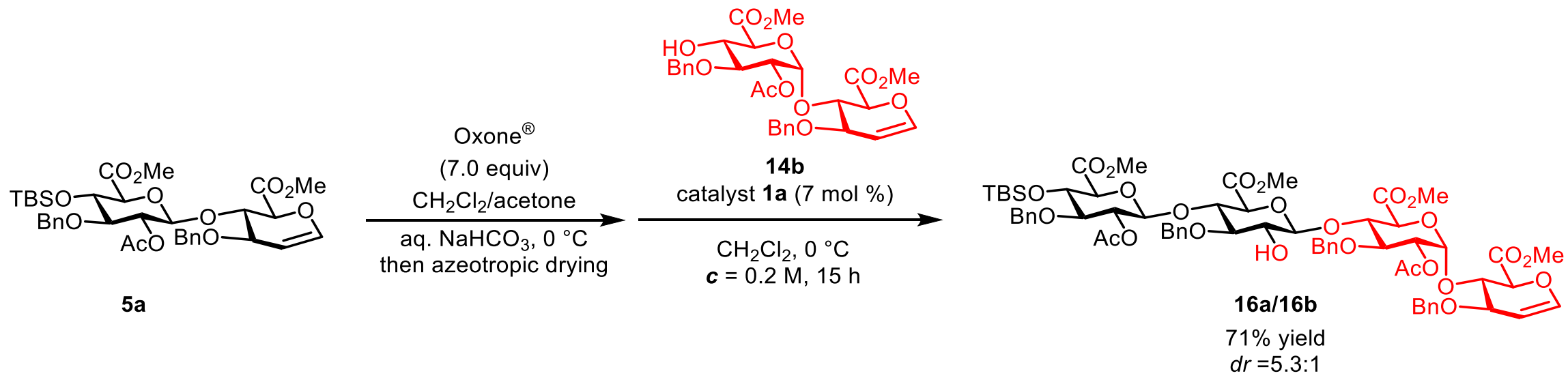

To a flame-dried sealable 2-dram vial equipped with a stir bar were added the above glycosyl acceptor **14b** (58.6 mg, 0.1 mmol, 2.0 equiv), the aforementioned glycal epoxide **S9** (30.8 mg, 0.05 mmol, 1.0 equiv), and freshly activated 5 Å molecular sieves, powder (*ca.* 50 mg). After the flask was evacuated and backfilled with N_2, anhydrous CH_2Cl_2 (0.15 mL) was added. The iron(III) porphyrin catalyst **1a** (0.0035 mmol, 7 mol %) solution in anhydrous CH_2Cl_2 (0.1 mL) was added to the vial and the resulting mixture was stirred at −40 °C for 5 min. The reaction mixture was kept at 0 °C for 15 h and then quenched with imidazole (3.5 mg) in CH_2Cl_2 (0.5 mL) at the same temperature. The mixture was filtered through a pad of Celite® silica gel and eluted with CH_2Cl_2 (5 mL). The organic layer was then concentrated *in vacuo* and *dr* of the glycosylation product determined based on crude reaction mixture is 5.3:1. The residue was purified through a silica gel flash column (hexanes/EtOAc: from 100:1 to 3:2) to afford the glycosylation product **16a/16b** as white foam (46.2 mg, 71% combined yield, *dr* : 5.3:1).

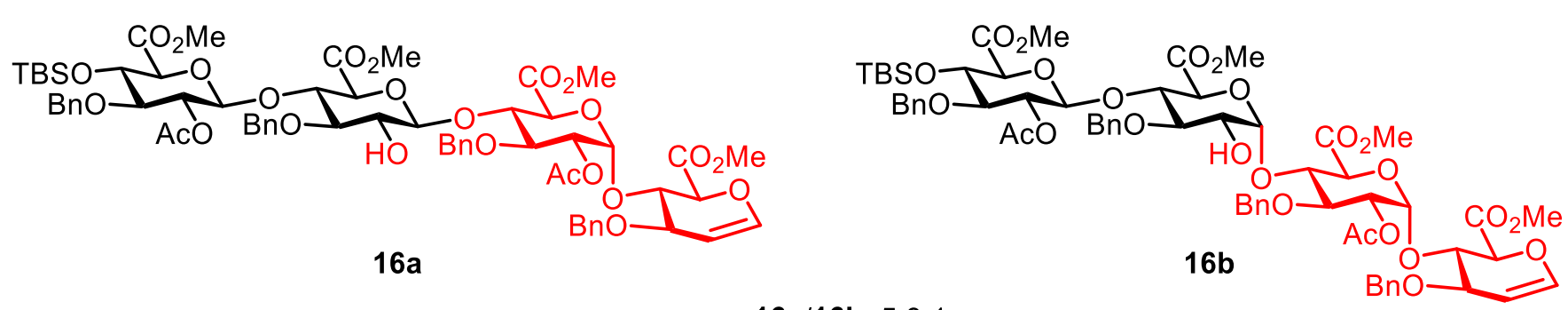

Compounds 16a and **16b**: IR ν_{max} (neat)/cm^{-1}: 2954 (w), 2926 (w), 2857 (w), 1747 (s), 1444 (m), 1220 (s), 1066 (s), 1039 (s), 699 (m); ^{1}H NMR for **16a**/**16b** (400 MHz, acetone-d_6) δ 7.45 (d, *J* = 6.9 Hz, 2H), 7.37 – 7.22 (m, 18H), 6.67 (**16b**, d, *J* = 6.3 Hz), 6.63 (**16a**, d, *J* = 6.3 Hz, 1H), 5.44 (**16b**, d, *J* = 3.3 Hz), 5.43 (**16b**, d, *J* = 3.6 Hz), 5.40 (d, *J* = 3.7 Hz, 1H), 5.02 (ddd, *J* = 6.5, 5.1, 1.6 Hz, 1H), 4.98 – 4.87 (m, 4H), 4.84 – 4.68 (m, 6H), 4.64 (d, *J* = 7.7 Hz, 1H), 4.57 – 4.46 (m, 4H), 4.38 (d, *J* = 9.2 Hz, 1H), 4.10 (t, *J* = 8.8 Hz, 1H), 4.01 – 3.90 (m, 4H), 3.85 (dd, *J* = 9.4, 8.3 Hz, 1H), 3.80 – 3.78 (m, 1H), 3.76 (s, 3H), 3.69 (s, 3H), 3.67 – 3.60 (m, 1H), 3.62 (s, 3H), 3.56 –

3.49 (m, 1H), 3.53 (s, 3H), 3.41 (t, J = 8.3 Hz, 1H), 2.00 (**16b**, s), 1.99 (**16b**, s), 1.93 (**16a**, s, 3H), 1.92 (**16a**, s, 3H), 0.84 (s, 9H), -0.01 (s, 3H), -0.02 (s, 3H); ^{13}C NMR for **16a** (100 MHz, acetone-d_6) δ 170.5, 169.94, 169.85, 169.4, 169.2, 168.3, 145.9, 140.5, 140.0, 139.35, 139.31, 129.04 (2C), 129.00 (2C), 128.9 (2C), 128.8 (2C), 128.6 (2C), 128.5 (2C), 128.4 (2C), 128.3, 128.2, 128.0 (two peaks overlapped, 3C), 127.8, 104.5, 101.4, 99.3, 97.5, 83.1 (two peaks overlapped, 2C), 83.0, 79.7, 79.4, 78.1, 77.1, 75.5, 75.3, 75.1, 75.0, 74.5, 74.3, 73.9, 73.2, 72.4, 71.9, 70.0, 68.2, 53.0, 52.9, 52.5, 52.2, 26.2 (3C), 21.0, 20.8, 18.5, -3.7, -5.0; HRMS: m/z (ESI) calcd for $C_{66}H_{83}O_{25}Si^+$, [M + H]$^+$, 1303.4987, found 1303.5012. $^1J^{13}{}_{C1-H1}$ (**16a**) = 172.0 Hz, 162.1 Hz, 162.8 Hz.

The stereochemistry of newly formed anomeric center (C1) of **16a** was determined by measuring $^1J^{13}{}_{C1-H1}$ (162.8 Hz) through un-decoupled HSQC experiments, which suggested the newly formed glycosidic bond is in equatorial position. The stereochemistry of newly formed anomeric center (C1) of **16b** was determined by measuring $^1J^{13}{}_{C1-H1}$ (170.6 Hz) through un-decoupled HSQC experiments, which suggested the newly formed glycosidic bond is in axial position.

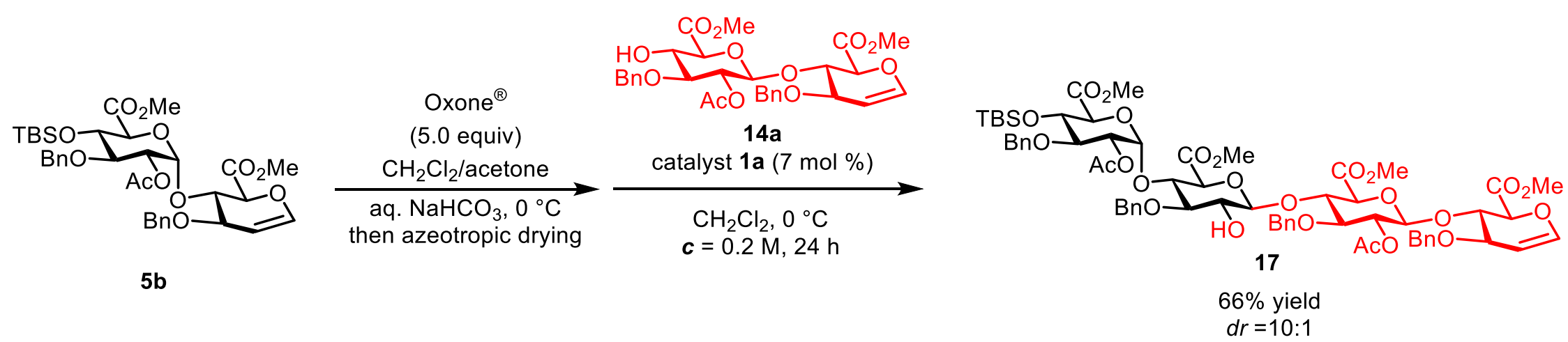

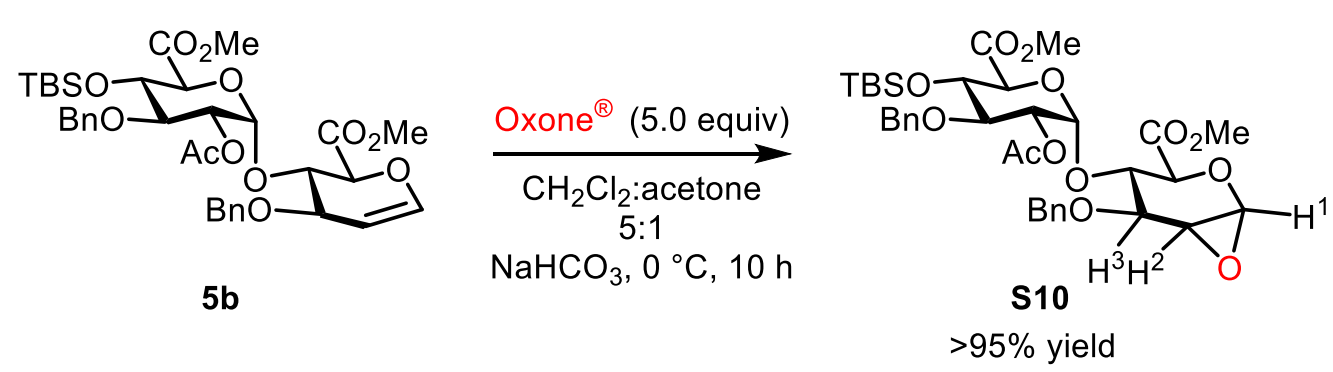

To a 25 mL flask equipped with a stir bar at 0 °C were added a glycal **5b** (0.7 g, 1.0 mmol, 1.0 equiv) in CH_2Cl_2/acetone mixture (v/v: 5:1, 10 mL) and saturated aqueous $NaHCO_3$ solution (24 mL), followed by addition of Oxone® ($KHSO_5 \cdot 0.5KHSO_4 \cdot 0.5K_2SO_4$) (2.35 g, 3.5 mmol, 3.5 equiv) in H_2O (11 mL) dropwise. After stirring vigorously at 0 °C for 5 h, the conversion was

determined as 76% by ^{1}H NMR analysis. Additional Oxone® ($KHSO_5 \cdot 0.5KHSO_4 \cdot 0.5K_2SO_4$) (1.01 g, 1.5 mmol, 1.5 equiv) in H_2O (5 mL) was added dropwise and stirred at 0 °C for another 5 h. The reaction mixture was extracted with CH_2Cl_2 (20 mL × 3). The combined organic phase was dried over anhydrous Na_2SO_4 and concentrated *in vacuo*. The residue was further azeotropically dried with anhydrous toluene (1.0 mL × 3). The obtained glycal epoxide **S10** was assayed by ^{1}H NMR to get the diastereomeric ratio (*dr* >20:1) and directly used in the next step.

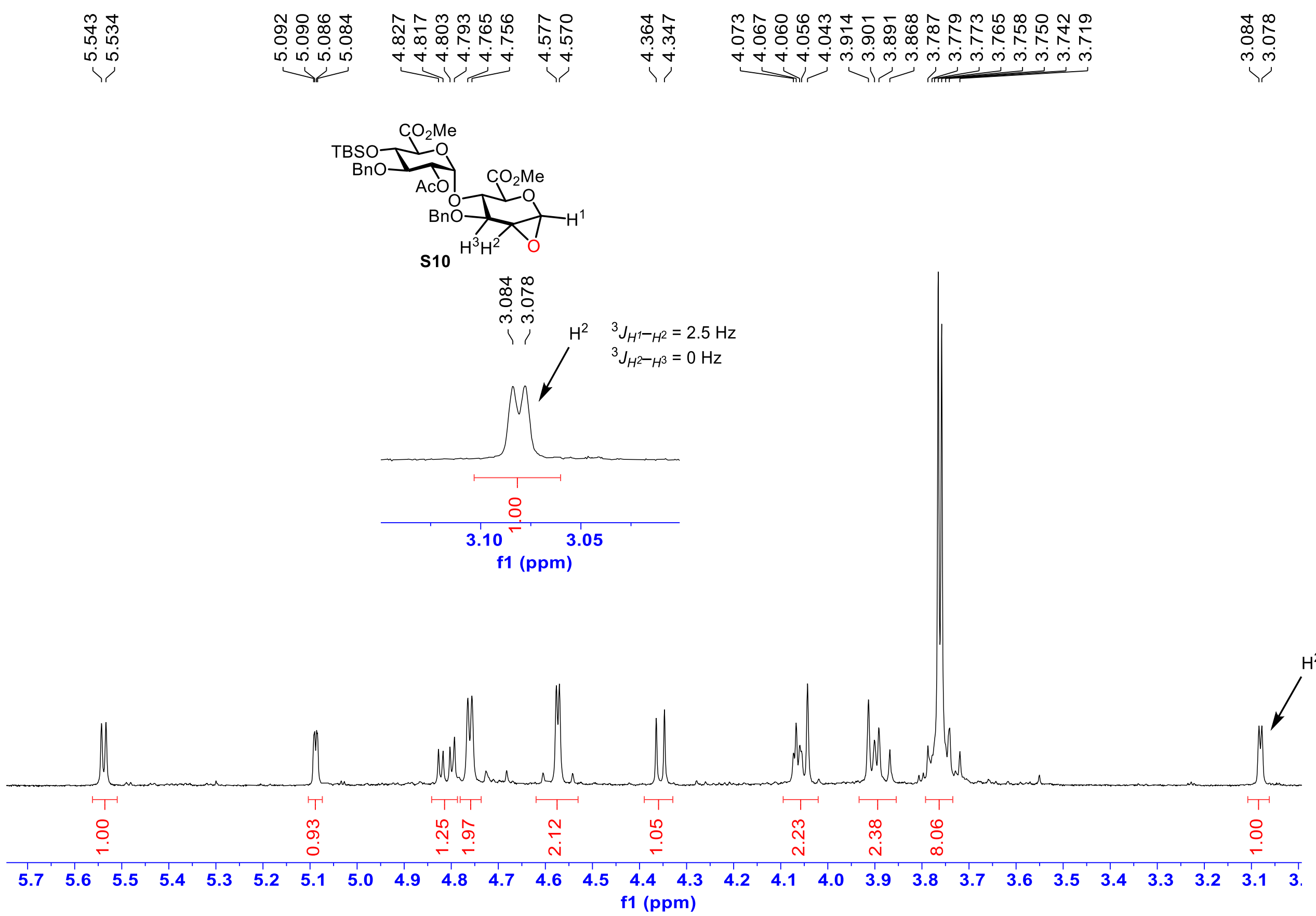

Figure S6. Stereochemistry Determination of Glycal Epoxide **S10**.

The stereochemistry of the glycal epoxide **S10** was determined by ^{1}H NMR analysis. It is known that the C(2)–H chemical shift of a glucal α-epoxide is around 3.2 ppm, whereas that of a glucal β-epoxide is around 3.3 ppm.[3]

Additionally, the $^3J_{H1-H2}$ value for a typical glucal α-epoxide is around 2.5 Hz, reflecting the dihedral angle ($\Phi_{1,2}$) that approaches 0°, whereas the $^3J_{H2-H3}$ value is < 0.5 Hz because the dihedral angle ($\Phi_{2,3}$) approaches 90°, characteristic of a *trans*-relationship between H^2 and H^3 that is pseudo-axial.[3] As a result, the H^2 signal of a glucal α-epoxide appears as a doublet.

In contrast, while a glucal β-epoxide retains a similar $\Phi_{1,2}$ and thus a comparable $^3J_{H1-H2}$, its $\Phi_{2,3}$ is around 45°, leading to a $^3J_{H2-H3}$ around 1.7 Hz.[3] This causes the H^2 signal to appear as a doublet of doublets (though often observed as a triplet due to the broadening line width).

Based on these data, we have assigned glycal epoxide **S10** as an α-epoxide (*dr* >20:1) with $^3J_{H1-H2}$ = 2.5 Hz and $^3J_{H2-H3}$ = 0 Hz. This assignment was further corroborated by the stereochemistry analysis of glycosylation product **17**.

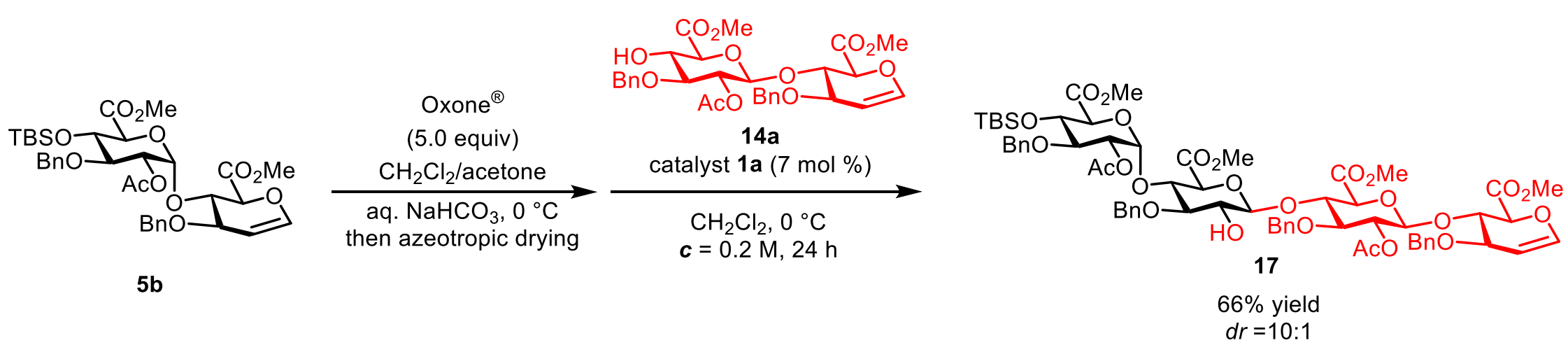

To a flame-dried sealable 2-dram vial equipped with a stir bar were added the above glycosyl acceptor **14b** (58.6 mg, 0.1 mmol, 2.0 equiv), the aforementioned glycal epoxide **S10** (30.8 mg, 0.05 mmol, 1.0 equiv), and freshly activated 5 Å molecular sieves, powder (*ca.* 50 mg). After the flask was evacuated and backfilled with N_2, anhydrous CH_2Cl_2 (0.15 mL) was added. The iron(III) porphyrin catalyst **1a** (0.0035 mmol, 7 mol %) solution in anhydrous CH_2Cl_2 (0.1 mL) was added to the vial and the resulting mixture was stirred at −40 °C for 5 min. The reaction mixture was kept at 0 °C for 24 h and then quenched with imidazole (3.5 mg) in CH_2Cl_2 (0.5 mL) at the same temperature. The mixture was filtered through a pad of Celite® silica gel and eluted with CH_2Cl_2 (5 mL). The organic layer was then concentrated *in vacuo* and *dr* of the glycosylation product determined based on crude reaction mixture is 10:1. The residue was

purified through a silica gel flash column (hexanes/EtOAc: from 100:1 to 3:2) to afford the desired glycosylation product **17** as white foam (42.9 mg, 66% yield).

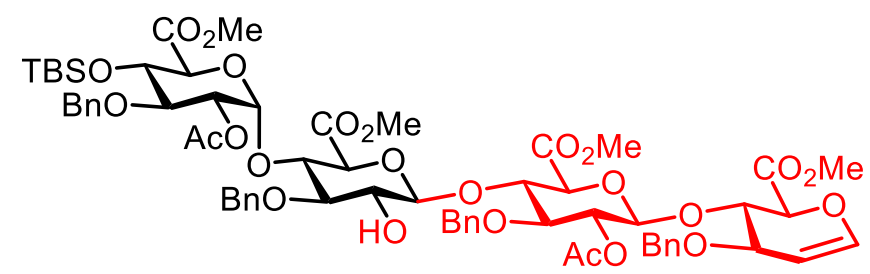

17

Methyl 3-*O*-benzyl-4-*O*-(methyl 2-*O*-acetyl-3-*O*-benzyl-4-*O*-*tert*-butyldimethylsilyl-α-D-glucopyranosyluronate)-(1→4)-(methyl 3-*O*-benzyl-β-D-glucopyranosyluronate)-(1→4)-(methyl 2-*O*-acetyl-3-*O*-benzyl-β-D-glucopyranosyluronate)-D-glucuronal (**17**): IR ν_{max} (neat)/cm^{-1}: 2953 (w), 2927 (w), 2878 (w), 1747 (s), 1366 (m), 1228 (s), 1065 (s), 1045 (s), 835 (m); ^{1}H NMR (400 MHz, $CDCl_3$) δ 7.36 – 7.17 (m, 20H), 6.53 (d, *J* = 6.3 Hz, 1H), 5.47 (d, *J* = 3.6 Hz, 1H), 4.99 – 4.89 (m, 3H), 4.89 – 4.82 (m, 2H), 4.76 (d, *J* = 11.8 Hz, 1H), 4.73 – 4.66 (m, 2H), 4.65 – 4.61 (m, 2H), 4.59 – 4.48 (m, 3H), 4.46 – 4.38 (m, 2H), 4.11 (t, *J* = 9.1 Hz, 1H), 4.04 (t, *J* = 8.9 Hz, 1H), 4.00 – 3.95 (m, 2H), 3.94 – 3.83 (m, 3H), 3.78 (s, 3H), 3.74 (s, 3H), 3.73 – 3.65 (m, 3H), 3.63 (s, 3H), 3.56 – 3.52 (m, 1H), 3.54 (s, 3H), 2.85 (d, *J* = 1.8 Hz, 1H), 1.84 (s, 3H), 1.71 (s, 3H), 0.85 (s, 9H), -0.02 (s, 3H), -0.06 (s, 3H); ^{13}C NMR (100 MHz, $CDCl_3$) δ 169.9, 169.24, 169.15, 168.9, 168.1, 167.9, 144.8, 138.4, 138.3, 138.1, 137.8, 128.4 (2C), 128.31 (2C), 128.28 (two peaks overlapped, 4C), 127.9 (2C), 127.73, 127.68, 127.66 (2C), 127.6 (2C), 127.5, 127.4, 126.8 (2C), 103.7, 99.8, 98.3, 96.6, 82.8, 80.3, 79.3, 79.2, 76.0, 75.1, 75.0, 74.7, 74.4, 74.2, 74.0, 73.0, 72.9, 72.3, 72.2, 72.1, 71.9, 69.6, 67.9, 53.1, 52.6, 52.3 (two peaks overlapped, 2C), 25.7 (3C), 20.6, 20.5, 17.9, -4.1, -5.4; HRMS: m/z (ESI) calcd for $C_{66}H_{83}O_{25}Si^+$, $[M + H]^+$, 1303.4987, found 1303.4996. $^1J_{^{13}C1\text{–}H1}$ = 175.6 Hz, 161.5 Hz, 162.0 Hz.

The stereochemistry of newly formed anomeric center (C1) of **17** was determined by measuring $^1J_{^{13}C1\text{–}H1}$ (162.9 Hz) through un-decoupled HSQC experiments, which suggested the newly formed glycosidic bond is in equatorial position. The C2 stereochemistry was determined by measuring $^3J_{H1\text{–}H2}$ (7.6 Hz), which suggested that the H2 is in axial position and corroborated the exclusive formation of the α-epoxide in glycal epoxidation.

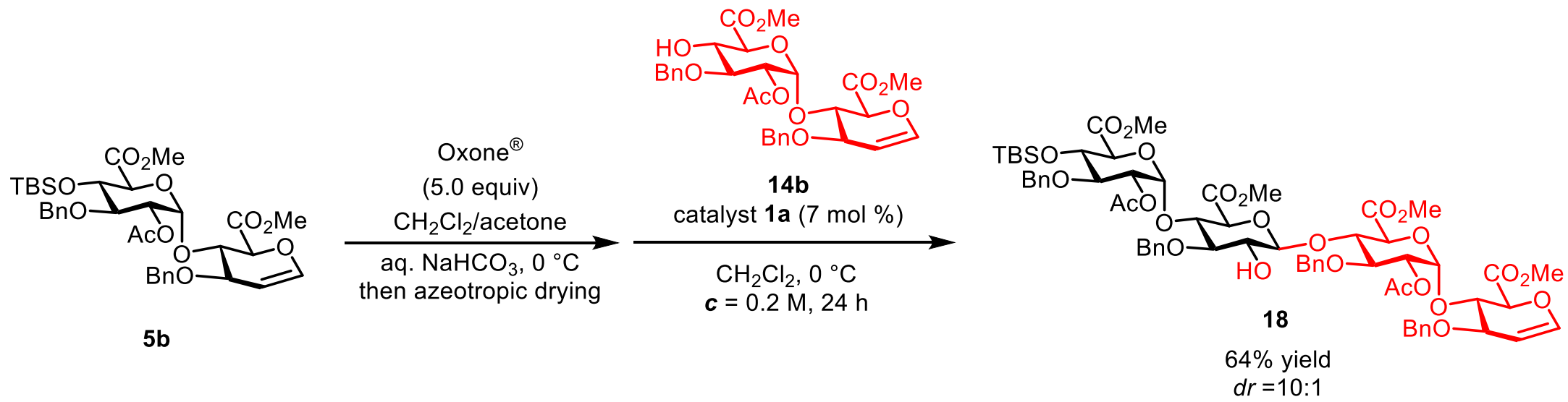

To a flame-dried sealable 2-dram vial equipped with a stir bar were added the above glycosyl acceptor **14b** (58.6 mg, 0.1 mmol, 2.0 equiv), the aforementioned glycal epoxide **S10** (30.8 mg, 0.05 mmol, 1.0 equiv), and freshly activated 5 Å molecular sieves, powder (*ca.* 50 mg). After the flask was evacuated and backfilled with N_2, anhydrous CH_2Cl_2 (0.15 mL) was added. The iron(III) porphyrin catalyst **1a** (0.0035 mmol, 7 mol %) solution in anhydrous CH_2Cl_2 (0.1 mL) was added to the vial and the resulting mixture was stirred at −40 °C for 5 min. The reaction mixture was kept at 0 °C for 24 h and then quenched with imidazole (3.5 mg) in CH_2Cl_2 (0.5 mL) at the same temperature. The mixture was filtered through a pad of Celite® silica gel and eluted with CH_2Cl_2 (5 mL). The organic layer was then concentrated *in vacuo* and *dr* of the glycosylation product determined based on crude reaction mixture is 10:1. The residue was purified through a silica gel flash column (hexanes/EtOAc: from 100:1 to 3:2) to afford the desired glycosylation product **18** as white foam (41.7 mg, 64% yield).

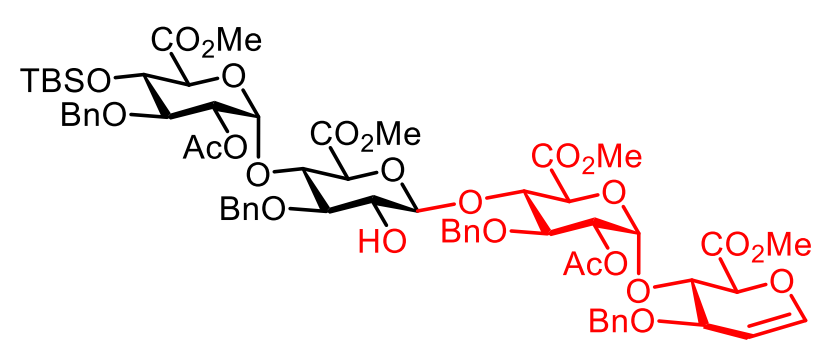

18

Methyl 3-*O*-benzyl-4-*O*-(methyl 2-*O*-acetyl-3-*O*-benzyl-4-*O*-*tert*-butyldimethylsilyl-α-D-glucopyranosyluronate)-(1→4)-(methyl 3-*O*-benzyl-β-D-glucopyranosyluronate)-(1→4)-(methyl 2-*O*-acetyl-3-*O*-benzyl-α-D-glucopyranosyluronate)-D-glucuronal (**18**): IR ν_{max} (neat)/cm^{-1}: 2952 (w), 2928 (w), 2856 (w), 1747 (s), 1438 (m), 1364 (m), 1232 (m), 1053 (s), 838 (m); ^1H NMR (400 MHz, $CDCl_3$) δ 7.38 – 7.21 (m, 20H), 6.66 (d, J = 6.3 Hz, 1H), 5.48 (d, J = 3.7 Hz, 1H), 5.35 (d, J = 3.8 Hz, 1H), 5.02 – 4.97 (m, 1H), 4.96 – 4.87 (m, 4H), 4.79 – 4.65 (m,

4H), 4.59 (d, J = 11.9 Hz, 1H), 4.55 (d, J = 7.3 Hz, 1H), 4.45 (ABq, $\Delta\nu_{AB}$ = 31.3 Hz, J = 11.4 Hz, 2H), 4.39 – 4.32 (m, 2H), 4.09 – 3.99 (m, 2H), 3.98 – 3.90 (m, 2H), 3.88 – 3.82 (m, 2H), 3.79 (s, 3H), 3.73 (s, 3H), 3.72 – 3.64 (m, 2H), 3.62 (s, 3H), 3.58 – 3.51 (m, 2H), 3.55 (s, 3H), 3.19 (d, J = 1.7 Hz, 1H), 1.83 (s, 3H), 1.70 (s, 3H), 0.85 (s, 9H), -0.03 (s, 3H), -0.06 (s, 3H); ^{13}C NMR (100 MHz, $CDCl_3$) δ 170.2, 170.0, 169.9, 169.3, 167.93, 167.91, 145.5, 138.8, 138.4, 138.1, 137.5, 128.42 (2C), 128.40 (2C), 128.32 (2C), 128.30 (2C), 128.0 (2C), 127.9, 127.7, 127.6 (2C), 127.39, 127.37, 126.9 (2C), 126.8 (2C), 103.9, 98.1, 97.0, 96.5, 82.8, 79.2, 78.8, 78.2, 75.9, 75.1, 75.03, 75.02, 74.5, 74.3, 74.2, 73.4, 73.0, 72.8, 72.3, 72.2, 70.0, 69.4, 66.7, 53.2, 52.6, 52.3, 52.2, 25.7 (3C), 20.57, 20.56, 17.9, -4.1, -5.3; HRMS: m/z (ESI) calcd for $C_{66}H_{83}O_{25}Si^+$, $[M + H]^+$, 1303.4987, found 1303.5005. $^1J_{^{13}C1\text{–}H1}$ = 178.8 Hz, 171.3 Hz, 162.8 Hz.

The stereochemistry of newly formed anomeric center (C1) of **18** was determined by measuring $^1J_{^{13}C1\text{–}H1}$ (162.3 Hz) through un-decoupled HSQC experiments, which suggested the newly formed glycosidic bond is in equatorial position. The C2 stereochemistry was determined by measuring $^3J_{H1\text{–}H2}$ (7.3 Hz), which suggested that the H2 is in axial position and corroborated the exclusive formation of the α-epoxide in glycal epoxidation.

E. Iron-Catalyzed Stereospecific Glycosylation with Glycal Epoxides for PGA Octasaccharide Synthesis

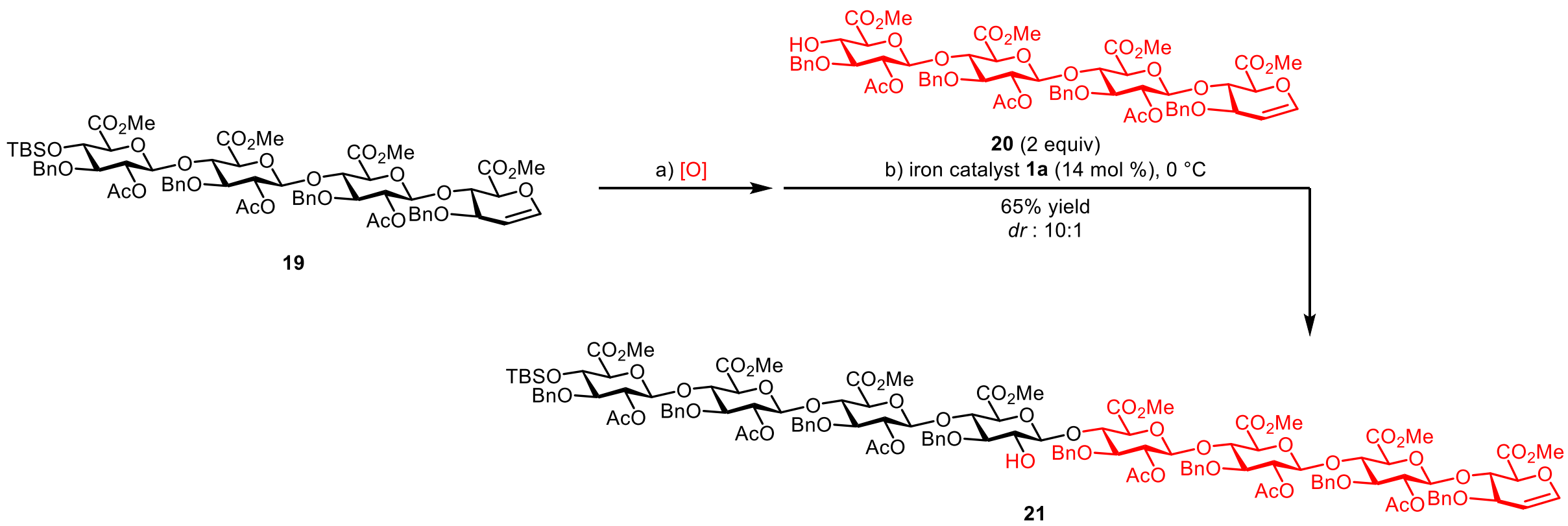

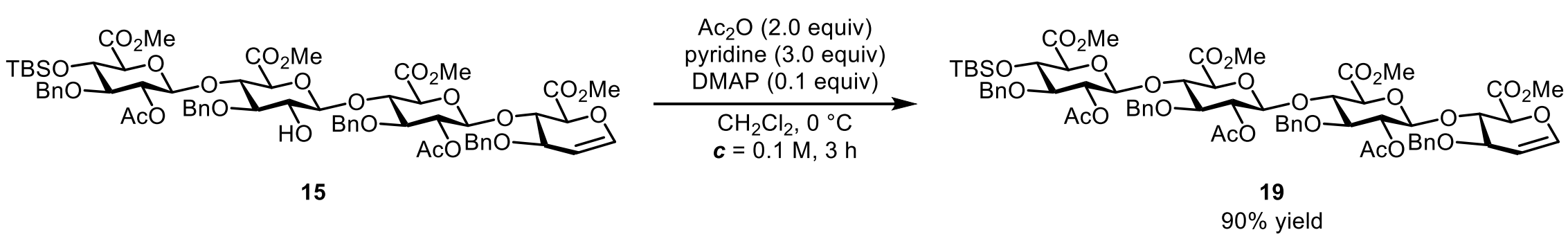

To a solution of **15** (350.0 mg, 0.27 mmol, 1.0 equiv) and DMAP (3.3 mg, 0.027 mmol, 0.1 equiv) in anhydrous CH_2Cl_2 (2.7 mL) was added pyridine (65 μL, 0.80 mmol, 3.0 equiv) at 0 °C. After stirring at 0 °C for 10 min, Ac_2O (50 μL, 0.54 mmol, 2.0 equiv) was added dropwise. The reaction mixture was kept at 0 °C and stirred for 3 h, with progress monitored by TLC until completion. The reaction was then quenched with saturated aqueous $NaHCO_3$ solution (5 mL). The organic phase was separated from the aqueous one, which was then extracted with EtOAc (5 mL × 3). The combined organic layer was washed with brine, dried over anhydrous Na_2SO_4, and then concentrated *in vacuo*. The desired product **19** was separated through column chromatography (hexanes/acetone: from 20:1 to 2:1) as white foam (324.1 mg, 90% yield).

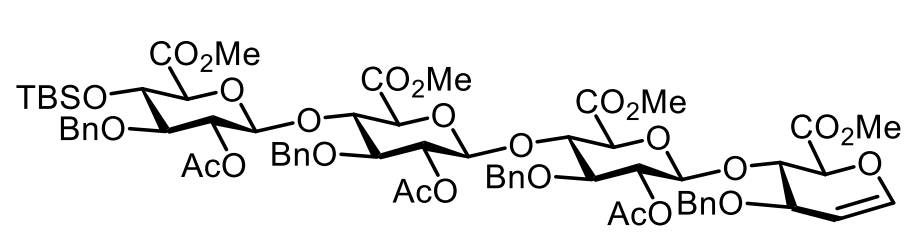

19

Methyl 3-*O*-benzyl-4-*O*-(methyl 2-*O*-acetyl-3-*O*-benzyl-4-*O*-*tert*-butyldimethylsilyl-β-D-glucopyranosyluronate)-(1→4)-(methyl 2-*O*-acetyl-3-*O*-benzyl-β-D-glucopyranosyluronate)-(1→4)-(methyl 2-*O*-acetyl-3-*O*-benzyl-β-D-glucopyranosyluronate)-D-glucuronal (**19**): IR ν_{max} (neat)/cm^{-1}: 2953 (w), 2931 (w), 2894 (w), 2858 (w), 1751 (s), 1371 (m), 1224 (s), 1068 (s), 839 (m); ^{1}H NMR (400 MHz, $CDCl_3$) δ 7.38 – 7.24 (m, 20H), 6.55 (d, *J* = 6.3 Hz, 1H), 5.02 (dd, *J* = 9.1, 8.0 Hz, 1H), 5.00 – 4.95 (m, 2H), 4.95 – 4.90 (m, 2H), 4.85 (d, *J* = 11.9 Hz, 1H), 4.73 (d, *J* = 11.6 Hz, 1H), 4.67 – 4.61 (m, 3H), 4.61 – 4.50 (m, 5H), 4.46 – 4.40 (m, 2H), 4.18 – 4.09 (m, 2H), 4.00 (t, *J* = 8.6 Hz, 1H), 3.91 – 3.84 (m, 3H), 3.81 – 3.76 (m, 1H), 3.79 (s, 3H), 3.64 – 3.54 (m, 2H), 3.62 (s, 3H), 3.61 (s, 3H), 3.56 (s, 3H), 3.47 (t, *J* = 8.7 Hz, 1H), 1.913 (s, 3H), 1.907 (s, 3H), 1.89 (s, 3H), 0.86 (s, 9H), -0.00 (s, 3H), -0.04 (s, 3H); ^{13}C NMR (100 MHz, $CDCl_3$) δ 169.5, 169.4, 169.2, 168.2, 168.1, 168.02, 167.97, 144.8, 138.6, 138.4, 138.0, 137.8, 128.3 (2C), 128.24 (2C), 128.19 (two peaks overlapped, 4C), 127.9 (two peaks overlapped, 4C), 127.7 (2C), 127.6, 127.5, 127.4, 127.3, 127.2 (2C), 100.9, 100.7, 99.7, 98.4, 82.4, 79.8, 79.5, 78.7 (two peaks overlapped, 2C), 76.7, 74.83, 74.78, 74.4, 74.34, 74.25, 72.9, 72.8, 72.13, 72.10, 72.0, 71.5, 69.6, 67.9, 52.7, 52.5, 52.20, 52.17, 25.6 (3C), 20.7, 20.64, 20.61, 17.8, -4.2, -5.3; HRMS: m/z (ESI) calcd for $C_{68}H_{85}O_{26}Si^{+}$, $[M + H]^{+}$, 1345.5093, found 1345.5108. $^{1}J_{^{13}C1–H1}$ = 162.6 Hz, 162.5 Hz, 162.0 Hz.

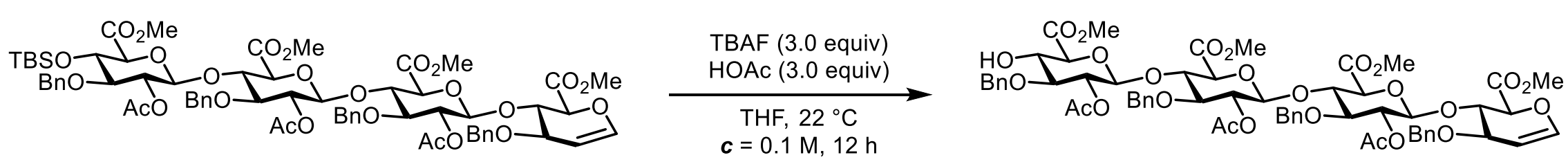

19

20
86% yield

To a 25 mL round bottom flask equipped with a stir bar were added **19** (350.1 mg, 0.26 mmol, 1.0 equiv) and THF (1.2 mL). The flask was cooled to 0 °C, and a premixed solution of TBAF (1.0 M in THF, 0.78 mL, 0.78 mmol, 3.0 equiv) and AcOH (1.0 M in THF, 0.78 mL, 0.78 mmol, 3.0 equiv) was added. The reaction mixture was stirred for 12 h at room temperature, with progress monitored by TLC until completion. The reaction mixture was diluted with EtOAc (5 mL) and then quenched with saturated aqueous NH_4Cl solution (10 mL). The organic phase was separated from the aqueous phase, which was extracted with EtOAc (10 mL × 4). The combined organic phase was washed with brine (15 mL) and dried over Na_2SO_4. The desired product **20**

was isolated through column chromatography (hexanes/acetone: from 20:1 to 2:1) as white foam (276.1 mg, 86% yield).

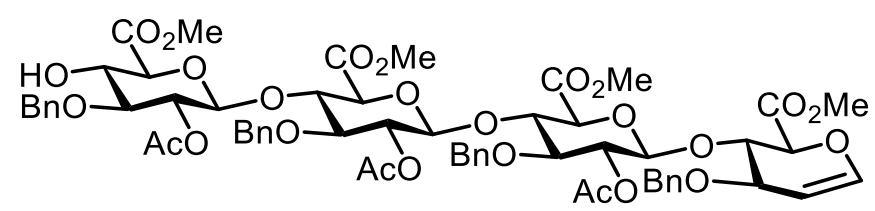

20

Methyl 3-*O*-benzyl-4-*O*-(methyl 2-*O*-acetyl-3-*O*-benzyl-β-D-glucopyranosyluronate)-(1→4)-(methyl 2-*O*-acetyl-3-*O*-benzyl-β-D-glucopyranosyluronate)-(1→4)-(methyl 2-*O*-acetyl-3-*O*-benzyl-β-D-glucopyranosyluronate)-D-glucuronal (**20**): IR ν_{max} (neat)/cm^{-1}: 3030 (w), 2954 (w), 1746 (s), 1708 (s), 1362 (m), 1220 (s), 1060 (s), 1036 (s), 739 (m); ^{1}H NMR (400 MHz, acetone-d_6) δ 7.38 – 7.21 (m, 20H), 6.51 (d, *J* = 6.3 Hz, 1H), 5.03 (d, *J* = 7.9 Hz, 1H), 4.98 (d, *J* = 4.0 Hz, 1H), 4.96 (d, *J* = 2.0 Hz, 1H), 4.94 – 4.89 (m, 2H), 4.89 – 4.82 (m, 4H), 4.81 – 4.76 (m, 2H), 4.71 (d, *J* = 3.0 Hz, 1H), 4.68 (d, *J* = 6.6 Hz, 1H), 4.58 – 4.53 (m, 2H), 4.51 (d, *J* = 11.6 Hz, 1H), 4.48 – 4.41 (m, 2H), 4.17 – 4.01 (m, 4H), 3.98 (d, *J* = 9.8 Hz, 1H), 3.88 (td, *J* = 9.3, 5.6 Hz, 1H), 3.84 – 3.79 (m, 1H), 3.81 (s, 3H), 3.78 – 3.70 (m, 2H), 3.68 (s, 3H), 3.64 (d, *J* = 9.3 Hz, 1H), 3.60 (s, 3H), 3.50 (s, 3H), 1.98 (s, 3H), 1.93 (s, 3H), 1.91 (s, 3H); ^{13}C NMR (100 MHz, acetone-d_6) δ 169.8, 169.7, 169.45, 169.42, 168.99, 168.97, 168.4, 145.7, 140.0, 139.9, 139.8, 139.4, 129.00 (2C), 128.96 (2C), 128.93 (2C), 128.92 (2C), 128.73 (2C), 128.67 (2C), 128.54 (2C), 128.51 (2C), 128.24, 128.20, 128.08, 128.06, 101.74, 101.67, 100.5, 99.1, 82.5, 80.78, 80.76, 79.9, 79.8, 76.4, 75.3, 75.2 (two peaks overlapped. 2C), 75.02, 74.99, 74.0, 73.2, 73.1, 73.0, 72.8, 72.5, 70.0, 69.0, 53.1, 53.0, 52.4, 52.1, 21.02, 20.96, 20.8; HRMS: m/z (ESI) calcd for $C_{62}H_{71}O_{26}^{+}$, $[M + H]^{+}$, 1231.4228, found 1231.4255. $^{1}J_{^{13}C1-H1}$ = 159.5 Hz, 162.8 Hz, 160.5 Hz.

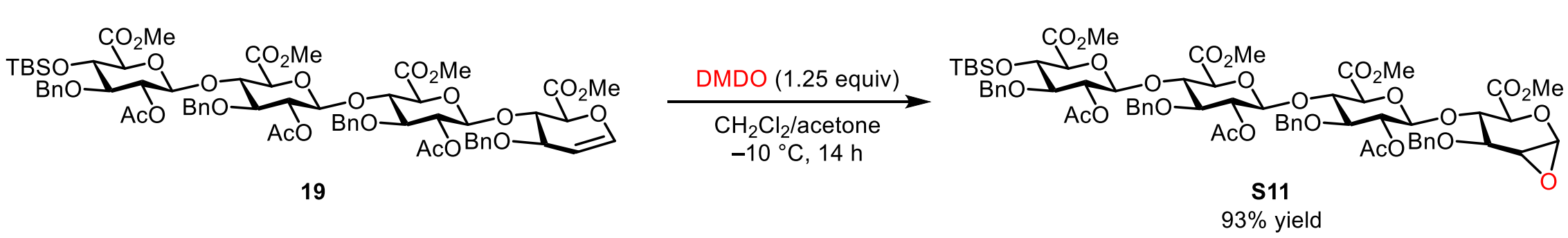

To a 25 mL flask equipped with a stir bar at –10 °C were added glycal **19** (134.5 mg, 0.1 mmol, 1.0 equiv) in CH_2Cl_2, followed by the addition of freshly prepared dimethyldioxirane (DMDO in

acetone, 0.06 M, 2.1 mL, 0.125 mmol, 1.25 equiv)[5] dropwise. After stirring at –10 °C for 14 h, the reaction mixture was concentrated *in vacuo*, then the residue was re-dissolved in anhydrous CH_2Cl_2 (2 mL) and dried over anhydrous Na_2SO_4. The organic phase concentrated *in vacuo*. The residue was further dried azeotropically with anhydrous toluene (1.0 mL × 3). The obtained glycal epoxide **S11** was assayed by ^{1}H NMR to get the diastereomeric ratio (*dr* >20:1) and directly used in the next step.

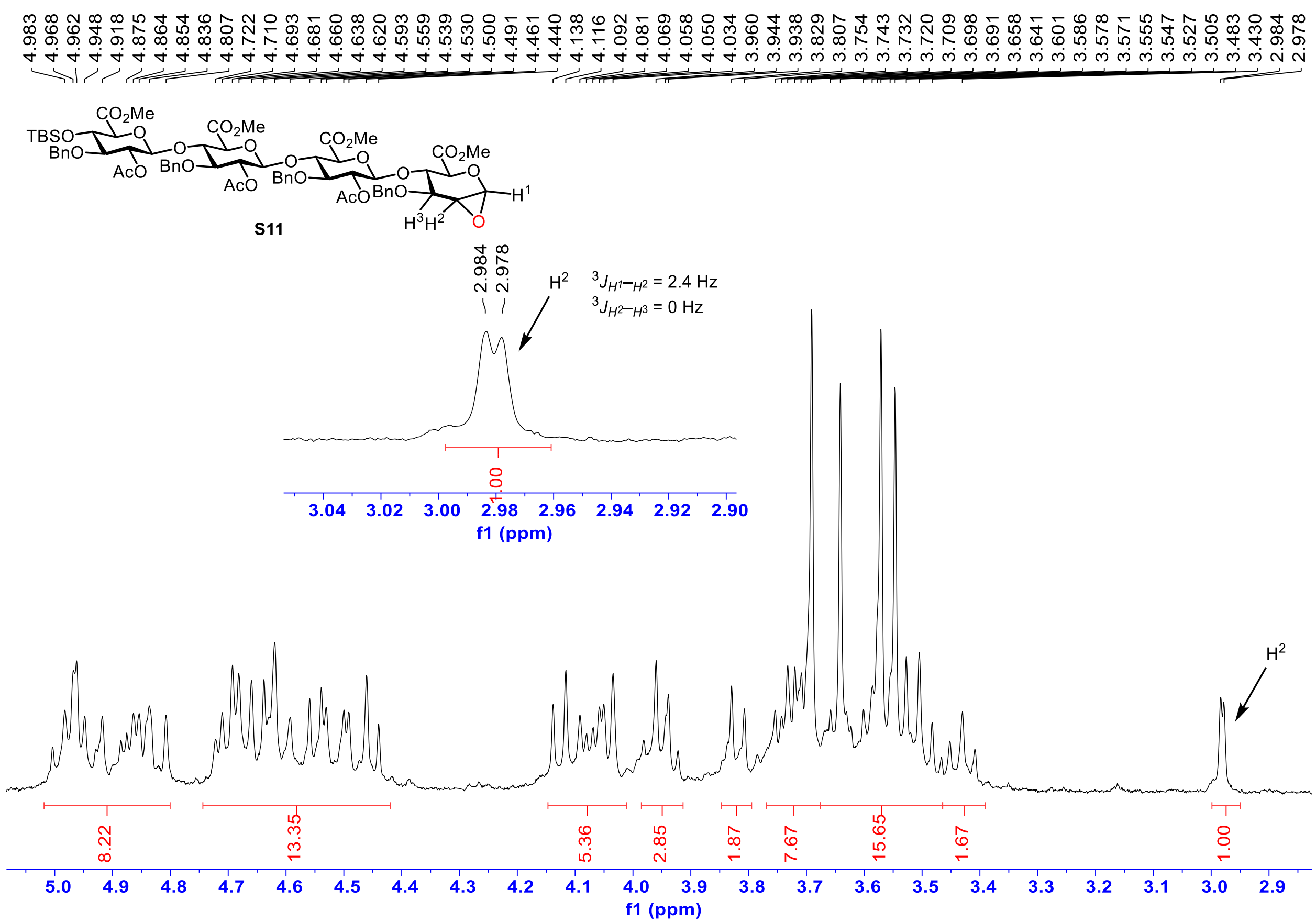

Figure S7. Stereochemistry Determination of Glycal Epoxide **S11**.

The stereochemistry of the glycal epoxide **S11** was determined by ^{1}H NMR analysis. It is known that the C(2)–H chemical shift of a glucal α-epoxide is around 3.0 ppm, whereas that of a glucal β-epoxide is around 3.3 ppm.[3]

Additionally, the $^3J_{H1-H2}$ value for a typical glucal α-epoxide is around 2.4 Hz, reflecting the dihedral angle ($\Phi_{1,2}$) that approaches 0°, whereas the $^3J_{H2-H3}$ value is < 0.5 Hz because the dihedral angle ($\Phi_{2,3}$) approaches 90°, characteristic of a *trans*-relationship between H^2 and H^3 that is pseudo-axial.[3] As a result, the H^2 signal of a glucal α-epoxide appears as a doublet.

In contrast, while a glucal β-epoxide retains a similar $\Phi_{1,2}$ and thus a comparable $^3J_{H1-H2}$, its $\Phi_{2,3}$ is around 45°, leading to a $^3J_{H2-H3}$ around 1.7 Hz.[3] This causes the H^2 signal to appear as a doublet of doublets (though often observed as a triplet due to the broadening line width).

Based on these data, we have assigned glycal epoxide **S11** as an α-epoxide (*dr* >20:1) with $^3J_{H1-H2}$ = 2.4 Hz and $^3J_{H2-H3}$ = 0 Hz. This assignment was further corroborated by the stereochemistry analysis of the glycosylation product **21**.

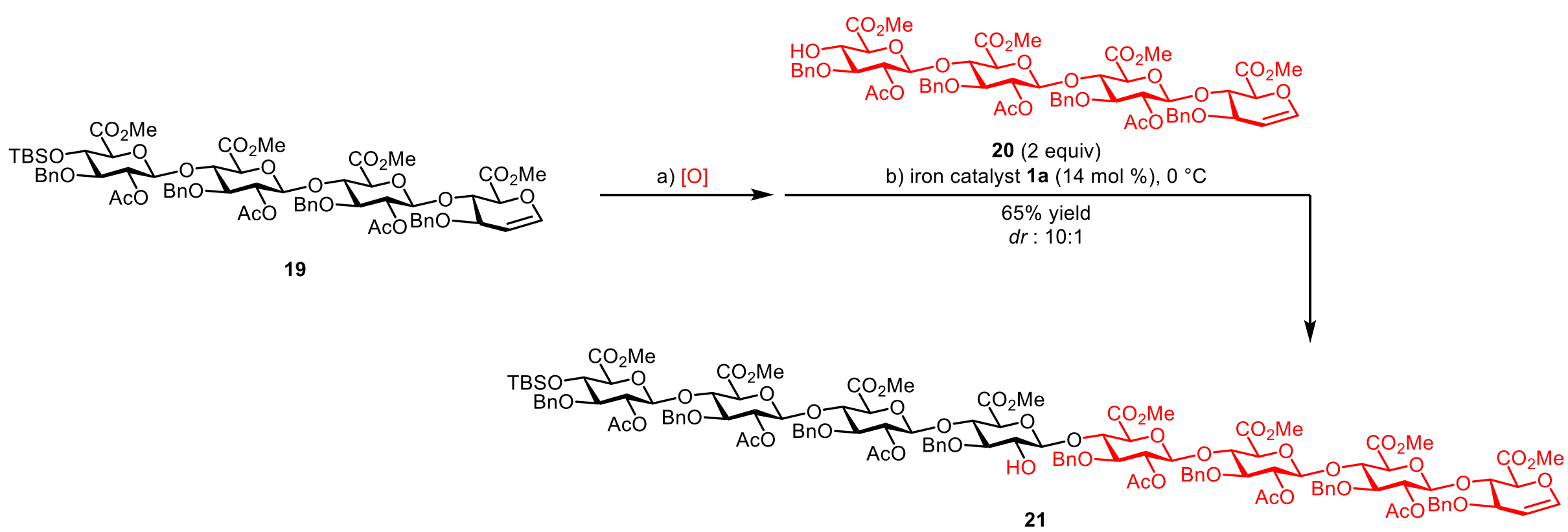

To a flame-dried sealable 2-dram vial equipped with a stir bar were added the above glycosyl acceptor **20** (196.8 mg, 0.16 mmol, 2.0 equiv), the aforementioned glycal epoxide **S11** (108.8 mg, 0.08 mmol, 1.0 equiv), and freshly activated 5 Å molecular sieves, powder (*ca.* 100 mg). After the flask was evacuated and backfilled with N_2, anhydrous CH_2Cl_2 (0.3 mL) was added. The iron(III) porphyrin catalyst **1a** (0.0112 mmol, 14 mol %) solution in anhydrous CH_2Cl_2 (0.1 mL) was added to the vial and the resulting mixture was stirred at −40 °C for 5 min. The reaction mixture was kept at 0 °C for 24 h and then quenched with imidazole (7.0 mg) in CH_2Cl_2 (1.0 mL) at the same temperature. The mixture was filtered through a pad of Celite® silica gel

and eluted with CH_2Cl_2 (5 mL). The organic layer was then concentrated *in vacuo* and *dr* of the glycosylation product determined based on crude reaction mixture is 10:1. The residue was purified through a silica gel flash column (hexanes/acetone: from 100:1 to 2:1) to afford the desired glycosylation product **21** as white foam (134.7 mg, 65% yield).

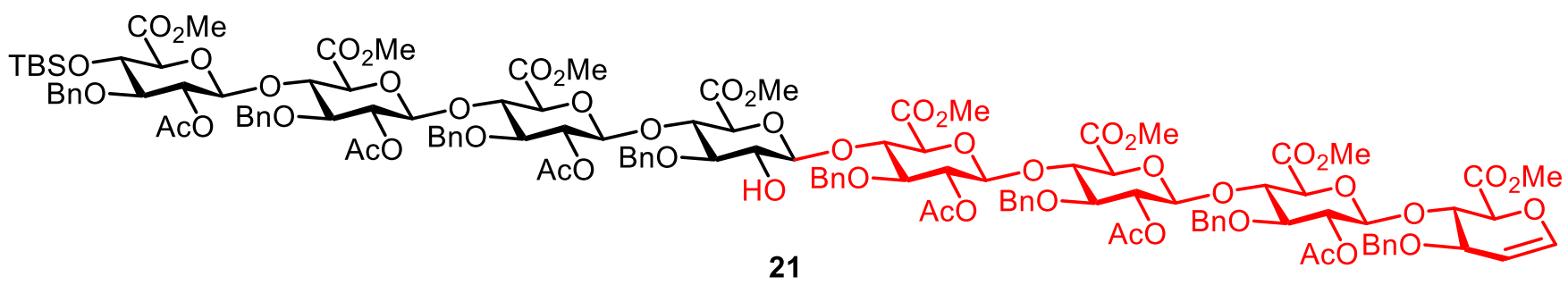

21

Methyl 3-*O*-benzyl-4-*O*-(methyl 2-*O*-acetyl-3-*O*-benzyl-4-*O*-*tert*-butyldimethylsilyl-β-D-glucopyranosyluronate)-(1→4)-(methyl 2-*O*-acetyl-3-*O*-benzyl-β-D-glucopyranosyluronate)-(1→4)-(methyl 2-*O*-acetyl-3-*O*-benzyl-β-D-glucopyranosyluronate)-(1→4)-(methyl 3-*O*-benzyl-β-D-glucopyranosyluronate)-(1→4)-(methyl 2-*O*-acetyl-3-*O*-benzyl-β-D-glucopyranosyluronate)-(1→4)-(methyl 2-*O*-acetyl-3-*O*-benzyl-β-D-glucopyranosyluronate)-(1→4)-(methyl 2-*O*-acetyl-3-*O*-benzyl-β-D-glucopyranosyluronate)-D-glucuronal (**21**): IR ν_{max} (neat)/cm^{-1}: 2954 (w), 2928 (w), 1746 (s), 1709 (m), 1362 (s), 1220 (s), 1062 (s), 1035 (s), 699 (m); ^{1}H NMR (400 MHz, acetone-d_6) δ 7.40 (d, *J* = 7.1 Hz, 2H), 7.37 – 7.22 (m, 38H), 6.50 (d, *J* = 6.3 Hz, 1H), 5.02 (d, *J* = 7.9 Hz, 1H), 4.98 – 4.88 (m, 8H), 4.88 – 4.67 (m, 15H), 4.60 – 4.42 (m, 9H), 4.16 – 3.87 (m, 15H), 3.83 – 3.75 (m, 1H), 3.81 (s, 3H), 3.75 – 3.69 (m, 4H), 3.69 – 3.66 (m, 1H), 3.68 (s, 3H), 3.67 (s, 3H), 3.65 (s, 3H), 3.63 (s, 3H), 3.61 (s, 6H), 3.53 – 3.48 (m, 1H), 3.50 (s, 3H), 3.39 (t, *J* = 8.3 Hz, 1H), 1.93 (s, 3H), 1.92 (s, 3H), 1.913 (s, 3H), 1.907 (s, 3H), 1.90 (s, 3H), 1.88 (s, 3H), 0.84 (s, 9H), -0.01 (s, 3H), -0.03 (s, 3H); ^{13}C NMR (100 MHz, acetone-d_6) δ 169.9, 169.70 (two peaks overlapped, 2C), 169.67, 169.6, 169.4, 169.23, 169.16, 169.1, 169.0, 168.94, 168.92, 168.8, 168.4, 145.7, 140.4, 139.95, 139.89 (two peaks overlapped, 2C), 139.88 (two peaks overlapped, 2C), 139.4, 139.3, 129.00 (2C), 128.98 (2C), 128.91 (four peaks overlapped, 8C), 128.89 (two peaks overlapped, 4C), 128.79 (2C), 128.76 (two peaks overlapped, 4C), 128.70 (2C), 128.68 (2C), 128.65 (2C), 128.5 (2C), 128.3 (2C), 128.23, 128.19, 128.1 (three peaks overlapped, 3C), 128.0 (two peaks overlapped, 2C), 127.7, 104.3, 101.64, 101.57 (two peaks overlapped, 2C), 101.5, 101.4, 100.4, 99.1, 83.0 (two peaks overlapped, 2C), 80.9, 80.75, 80.70, 80.66 (two peaks overlapped, 2C),

80.2, 79.93 (three peaks overlapped, 3C), 79.88, 79.4, 77.1, 75.44 (two peaks overlapped, 2C), 75.37 (two peaks overlapped, 2C), 75.29, 75.26, 75.14, 75.06, 74.9 (six peaks overlapped, 6C), 73.9, 73.8, 73.3, 73.0, 72.9 (two peaks overlapped, 2C), 72.83, 72.81, 72.5, 70.0, 69.0, 53.1, 53.02 (two peaks overlapped, 2C), 52.96, 52.9, 52.8, 52.5, 52.1, 26.1 (3C), 21.0 (two peaks overlapped, 2C), 20.94, 20.92 (two peaks overlapped, 2C), 20.8, 18.5, -3.7, -5.0; HRMS: m/z (ESI) calcd for $C_{130}H_{154}NaO_{53}Si^{+}$, $[M + Na]^{+}$, 2613.9017, found 2613.9045. $^{1}J^{13}{}_{C1-H1}$ = 162.9 Hz, 162.3 Hz, 162.3 Hz, 162.3 Hz, 162.3 Hz, 163.2 Hz, 162.5 Hz.

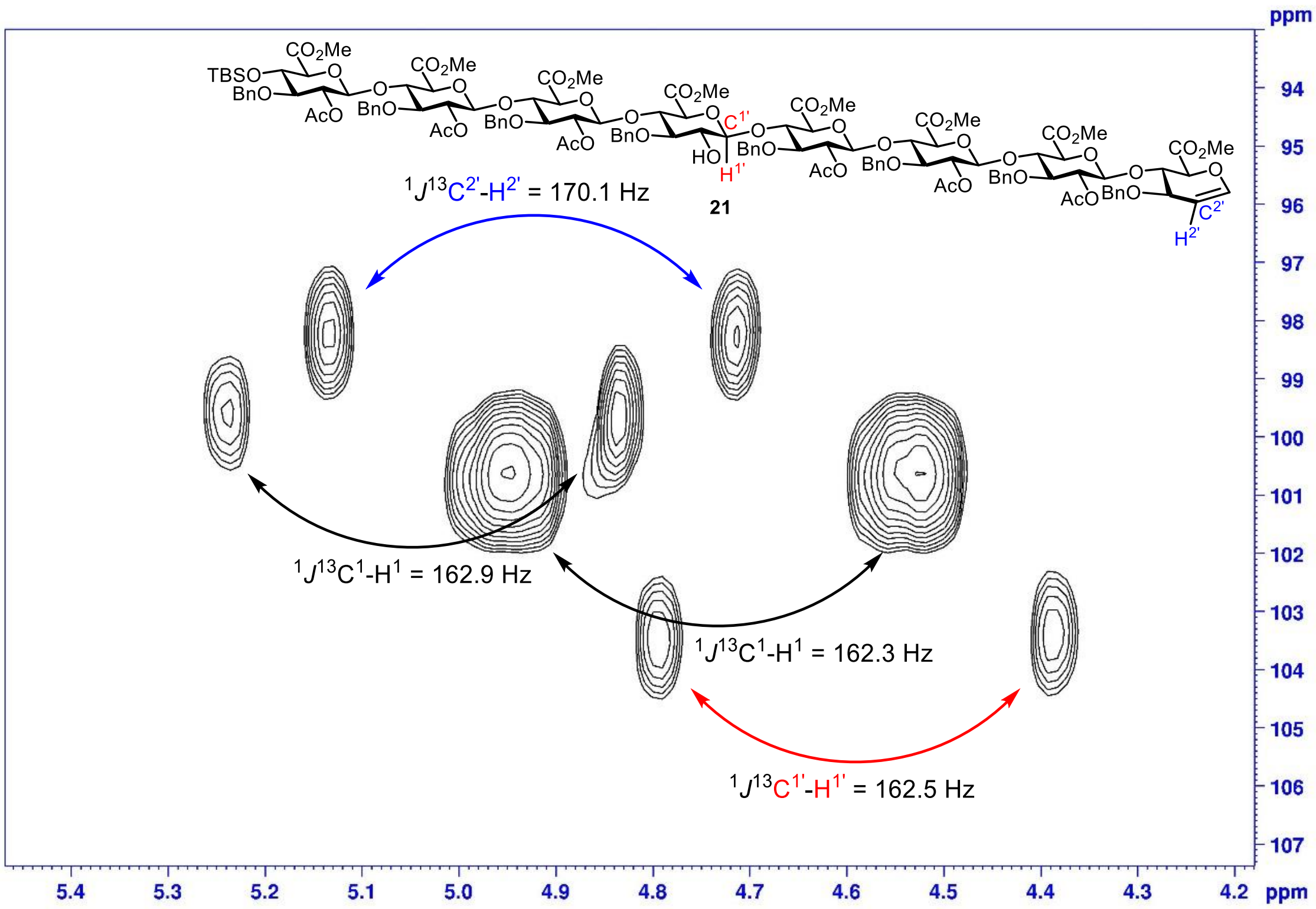

Figure S8. Un-decoupled HSQC Analysis to Determine Stereochemistry of **21**.

The stereochemistry of newly formed anomeric center (C1) of **21** was determined by measuring $^{1}J^{13}{}_{C1-H1}$ (162.5 Hz) through un-decoupled HSQC experiments, which suggested the newly formed glycosidic bond is in equatorial position.

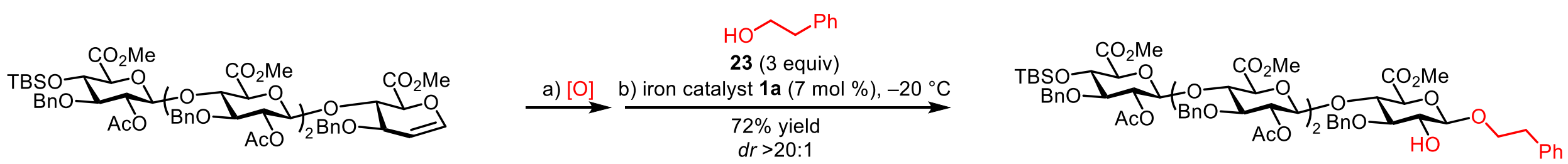

To a flame-dried sealable 2-dram vial equipped with a stir bar were added aforementioned glycal epoxide **S11** (142.0 mg, 0.106 mmol, 1.0 equiv), and freshly activated 5 Å molecular sieves, powder (*ca*. 100 mg). After the flask was evacuated and backfilled with N_2, 2-phenylethanol **23** (38 μL, 0.318 mmol, 3.0 equiv) and anhydrous CH_2Cl_2 (0.4 mL) was added. The iron(III) porphyrin catalyst **1a** (0.007 mmol, 7 mol %) solution in anhydrous CH_2Cl_2 (0.1 mL) was added to the vial and the resulting mixture was stirred at −40 °C for 5 min. The reaction mixture was kept at –20 °C for 14 h and then quenched with imidazole (7.0 mg) in CH_2Cl_2 (1.0 mL) at the same temperature. The mixture was filtered through a pad of Celite® silica gel and eluted with CH_2Cl_2 (5 mL). The organic layer was then concentrated *in vacuo* and *dr* of the glycosylation product determined based on crude reaction mixture is >20:1. The residue was purified through a silica gel flash column (hexanes/acetone: from 100:1 to 2:1) to afford the desired glycosylation product **24** as white foam (113.1 mg, 72% yield).

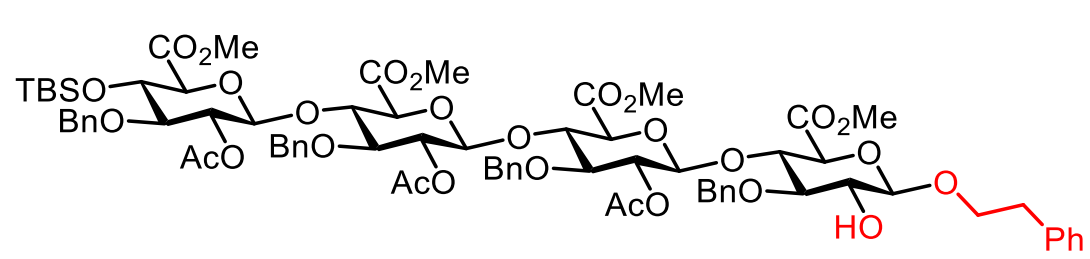

24

Methyl (methyl 2-*O*-acetyl-3-*O*-benzyl-4-*O*-*tert*-butyldimethylsilyl-β-D-glucopyranosyluronate)-(1→4)-(methyl 2-*O*-acetyl-3-*O*-benzyl-β-D-glucopyranosyluronate)-(1→4)-(methyl 2-*O*-acetyl-3-*O*-benzyl-β-D-glucopyranosyluronate)-(1→4)-(2-phenylethyl 3-*O*-benzyl-β-D-glucopyranosid)uronate (**24**): IR ν_{max} (neat)/cm^{-1}: 2954 (w), 2927 (w), 2856 (w), 1750 (s), 1371 (m), 1224 (s), 1067 (s), 839 (m), 699 (m); ^{1}H NMR (400 MHz, $CDCl_3$) δ 7.40 – 7.17 (m, 25H), 5.09 – 4.80 (m, 6H), 4.74 – 4.67 (m, 2H), 4.63 (d, *J* = 11.5 Hz, 1H), 4.60 – 4.55 (m, 2H), 4.51 (d, *J* = 8.5 Hz, 1H), 4.46 (d, *J* = 8.5 Hz, 1H), 4.28 (d, *J* = 7.6 Hz, 1H), 4.16 – 4.06 (m, 3H), 4.07 – 3.94 (m, 2H), 3.74 – 3.67 (m, 3H), 3.76 (s, 3H), 3.87 – 3.78 (m, 3H), 3.61 (s, 3H), 3.59 (s, 3H), 3.55 (s, 3H), 3.54 – 3.40 (m,

5H), 2.92 (t, J = 6.6 Hz, 2H), 2.13 (brs, 1H), 1.91 (s, 3H), 1.89 (s, 3H), 1.84 (s, 3H), 0.84 (s, 9H), -0.02 (s, 3H), -0.05 (s, 3H); ^{13}C NMR (100 MHz, $CDCl_3$) δ 169.5 (two peaks overlapped, 2C), 169.4, 168.4, 168.2, 168.0, 167.9, 138.7, 138.6, 138.5, 138.1, 138.0, 128.9 (2C), 128.44 (2C), 128.36 (2C), 128.3 (2C), 128.2 (two peaks overlapped, 4C), 127.9 (2C), 127.7 (2C), 127.6 (2C), 127.53, 127.50, 127.33, 127.31, 127.2 (2C), 126.4, 103.1, 100.9, 100.8, 100.7, 82.4, 81.6, 79.73, 79.68, 78.7, 78.6, 78.4, 76.7, 74.83, 74.82, 74.7, 74.6, 74.3, 74.23, 74.17, 73.5, 72.9, 72.15, 72.05, 71.9, 70.9, 52.7, 52.6, 52.5, 52.2, 36.0, 25.6 (3C), 20.71, 20.67, 20.6, 17.8, -4.1, -5.3; HRMS: m/z (ESI) calcd for $C_{76}H_{95}O_{28}Si^+$, $[M + H]^+$, 1483.5774, found 1483.5791. $^1J^{13}{}_{C1-H1}$ = 162.4 Hz, 162.4 Hz, 162.1 Hz, 162.5 Hz.

The stereochemistry of newly formed anomeric center (C1) of **24** was determined by measuring $^1J^{13}{}_{C1-H1}$ (162.5 Hz) through un-decoupled HSQC experiments, which suggested the newly formed glycosidic bond is in equatorial position. The C2 stereochemistry was determined by measuring $^3J_{H1-H2}$ (7.6 Hz), which suggested the H2 is in axial position and corroborated the exclusive formation of the α-epoxide in glycal epoxidation.

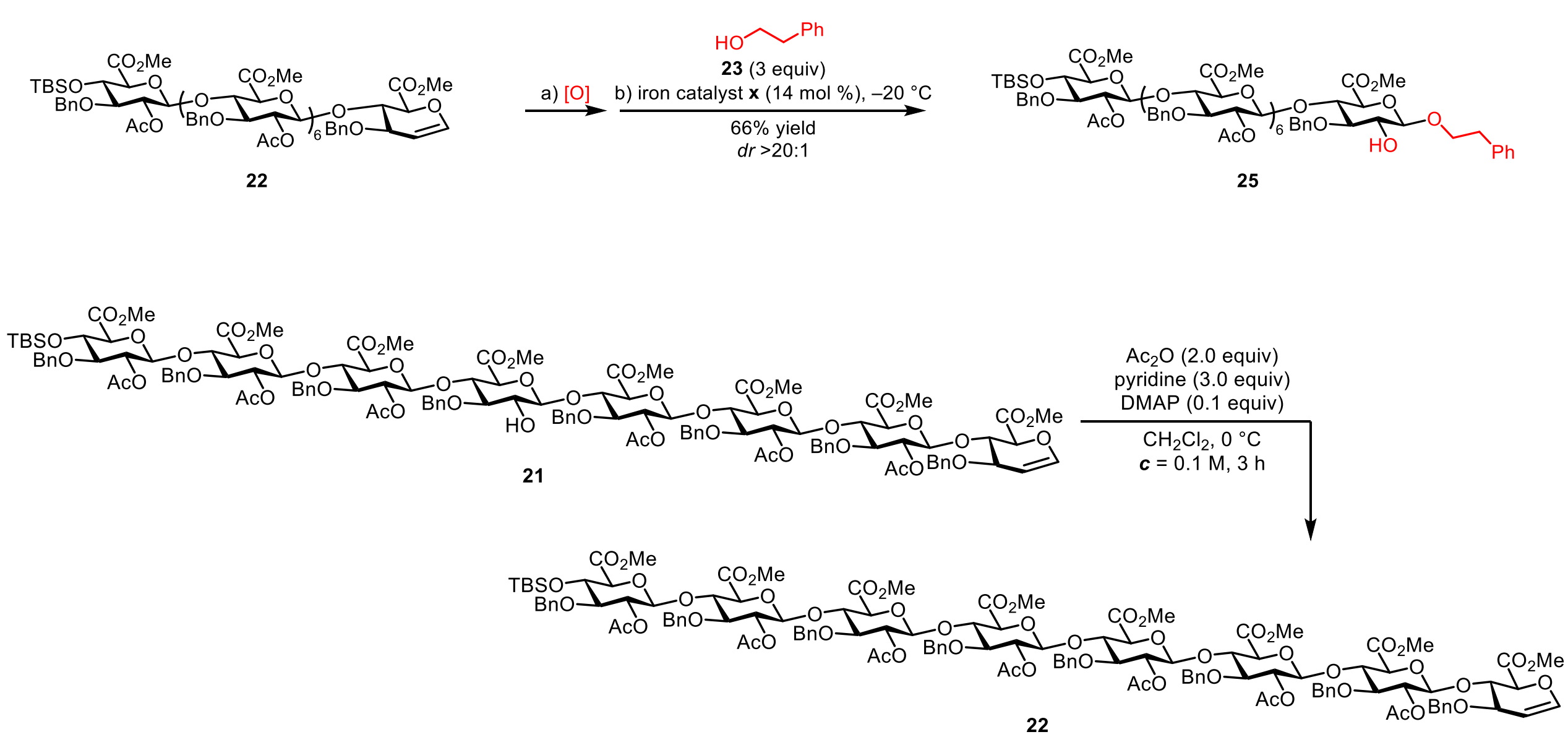

22

25

21

22
96% yield

To a solution of **21** (87.0 mg, 0.0336 mmol, 1.0 equiv) and DMAP (0.4 mg, 0.00336 mmol, 0.1 equiv) in anhydrous CH_2Cl_2 (0.1 mL) was added pyridine (8 μL, 0.101 mmol, 3.0 equiv) at 0 °C. After stirring at 0 °C for 10 min, Ac_2O (7 μL, 0.0672 mmol, 2.0 equiv) was added dropwise. The reaction mixture was kept at 0 °C and stirred for 3 h, with progress monitored by TLC until completion. The reaction was then quenched with saturated aqueous $NaHCO_3$ solution (5 mL). The organic phase was separated from the aqueous one, which was then extracted with EtOAc (5 mL × 3). The combined organic layer was washed with brine, dried over anhydrous Na_2SO_4, and then concentrated *in vacuo*. The desired product **22** was separated through column chromatography (hexanes/acetone: from 20:1 to 2:1) as white foam (85.1 mg, 96% yield).

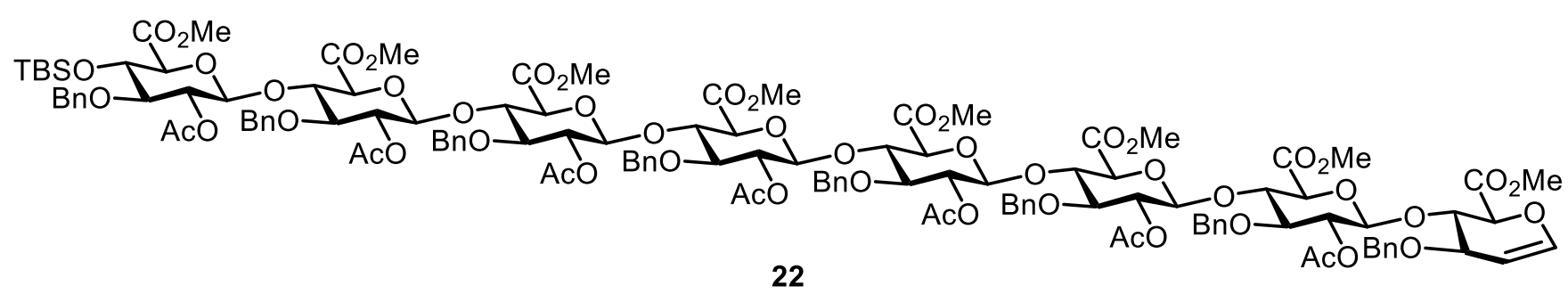

22

Methyl 3-*O*-benzyl-4-*O*-(methyl 2-*O*-acetyl-3-*O*-benzyl-4-*O*-*tert*-butyldimethylsilyl-β-D-glucopyranosyluronate)-(1→4)-(methyl 2-*O*-acetyl-3-*O*-benzyl-β-D-glucopyranosyluronate)-(1→4)-(methyl 2-*O*-acetyl-3-*O*-benzyl-β-D-glucopyranosyluronate)-(1→4)-(methyl 2-*O*-acetyl-3-*O*-benzyl-β-D-glucopyranosyluronate)-(1→4)-(methyl 2-*O*-acetyl-3-*O*-benzyl-β-D-glucopyranosyluronate)-(1→4)-(methyl 2-*O*-acetyl-3-*O*-benzyl-β-D-glucopyranosyluronate)-(1→4)-(methyl 2-*O*-acetyl-3-*O*-benzyl-β-D-glucopyranosyluronate)-D-glucuronal (**22**): IR ν_{max} (neat)/cm^{-1}: 2971 (w), 2940 (w), 1745 (s), 1721 (m), 1350 (s), 1222 (s), 1068 (s), 1038 (s), 835 (m); ^{1}H NMR (400 MHz, acetone-d_6) δ 7.36 – 7.22 (m, 40H), 6.50 (d, J = 6.3 Hz, 1H), 5.02 (d, J = 7.9 Hz, 1H), 5.00 – 4.87 (m, 8H), 4.88 – 4.76 (m, 7H), 4.79 – 4.67 (m, 8H), 4.55 (d, J = 4.7 Hz, 1H), 4.54 – 4.42 (m, 8H), 4.18 – 3.96 (m, 13H), 3.94 (t, J = 8.8 Hz, 1H), 3.82 – 3.78 (m, 1H), 3.80 (s, 3H), 3.74 – 3.67 (m, 7H), 3.68 (s, 3H), 3.66 (s, 3H), 3.64 (s, 9H), 3.60 (s, 3H), 3.49 (s, 3H), 1.93 (s, 3H), 1.92 (s, 3H), 1.909 (s, 3H), 1.905 (s, 3H), 1.903 (s, 3H), 1.901 (s, 3H), 1.88 (s, 3H), 0.84 (s, 9H), -0.01 (s, 3H), -0.03 (s, 3H); ^{13}C NMR (100 MHz, acetone-d_6) δ 169.0, 168.8 (five peaks overlapped, 5C), 168.6, 168.3, 168.07, 168.05, 167.97, 167.95, 167.94, 167.93, 167.5, 144.8, 139.1, 139.01 (two peaks overlapped, 2C), 139.00 (two peaks overlapped, 3C), 138.54, 138.46, 128.13 (2C), 128.10 (2C),

128.0 (six peaks overlapped, 12C), 127.9 (2C), 127.80 (four peaks overlapped, 8C), 127.77 (2C), 127.6 (2C), 127.4, 127.3, 127.2 (six peaks overlapped, 6C), 127.1 (2C), 100.8, 100.70 (four peaks overlapped, 4C), 100.65, 99.6, 98.2, 82.1, 79.9, 79.83, 79.76 (four peaks overlapped, 4C), 79.1 (five peaks overlapped, 5C), 79.0, 76.2, 74.6, 74.5, 74.4 (four peaks overlapped, 4C), 74.3, 74.1, 74.0 (five peaks overlapped, 5C), 73.1, 73.0, 72.4, 72.1, 72.01 (three peaks overlapped, 3C), 71.98, 71.9, 71.6, 69.1, 68.1, 52.2, 52.14 (two peaks overlapped, 2C), 52.11 (three peaks overlapped, 3C), 51.6, 51.3, 25.3 (3C), 20.09, 20.06, 20.0 (four peaks overlapped, 4C), 19.9, 17.6, -4.6, -5.9; HRMS: m/z (ESI) calcd for $C_{132}H_{156}NaO_{54}Si^+$, $[M + Na]^+$, 2655.9122, found 2655.9151. $^1J_{^{13}C1–H1}$ = 162.8 Hz, 162.3 Hz, 162.3 Hz, 162.3 Hz, 162.3 Hz, 163.3 Hz, 162.1 Hz.

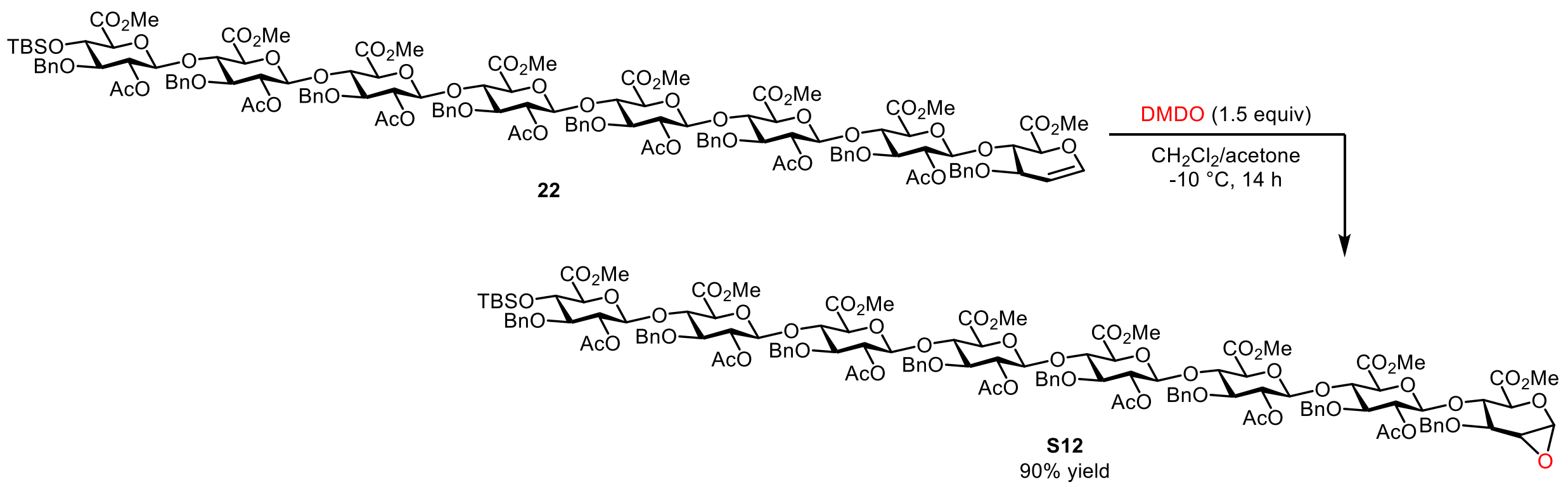

To a sealable 2-dram vial equipped with a stir bar at –10 °C were added a glycal **22** (40.0 mg, 0.0152 mmol, 1.0 equiv) in CH_2Cl_2, followed by the addition of freshly prepared dimethyldioxirane (DMDO in acetone, 0.06 M, 0.38 mL, 0.15 mmol, 1.5 equiv)[5] dropwise. After stirring at –10 °C for 14 h, the reaction mixture was concentrated *in vacuo*, then the residue was re-dissolved in anhydrous CH_2Cl_2 (2 mL) and dried over anhydrous Na_2SO_4. The organic phase concentrated *in vacuo*. The residue was further dried azeotropically with anhydrous toluene (1.0 mL × 3). The obtained glycal epoxide **S12** was assayed by ^{1}H NMR to get the diastereomeric ratio (*dr* > 20:1) and directly used in the next step.

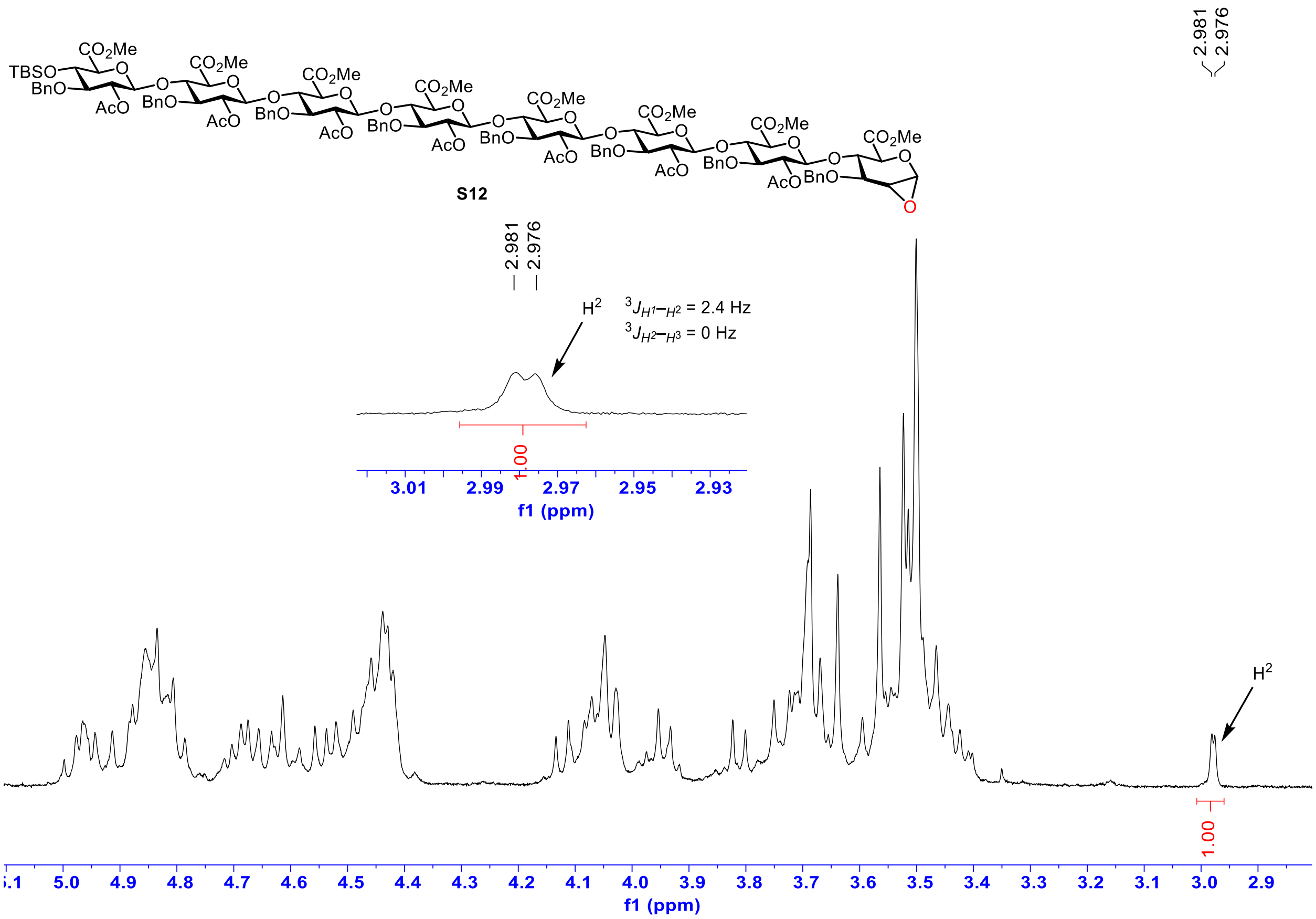

Figure S9. Stereochemistry Determination of Glycal Epoxide **S12**.

The stereochemistry of the glycal epoxide **S12** was determined by ^{1}H NMR analysis. It is known that the C(2)–H chemical shift of a glucal α-epoxide is around 3.0 ppm, whereas that of a glucal β-epoxide is around 3.3 ppm.[3]

Additionally, the $^3J_{H1-H2}$ value for a typical glucal α-epoxide is around 2.4 Hz, reflecting the dihedral angle ($\Phi_{1,2}$) that approaches 0°, whereas the $^3J_{H2-H3}$ value is < 0.5 Hz because the dihedral angle ($\Phi_{2,3}$) approaches 90°, characteristic of a *trans*-relationship between H^2 and H^3 that is pseudo-axial.[3] As a result, the H^2 signal of a glucal α-epoxide appears as a doublet.

In contrast, while a glucal β-epoxide retains a similar $\Phi_{1,2}$ and thus a comparable $^{3}J_{H1-H2}$, its $\Phi_{2,3}$ is around 45°, leading to a $^{3}J_{H2-H3}$ around 1.7 Hz.[3] This causes the H^2 signal to appear as a doublet of doublets (though often observed as a triplet due to the broadening line width).

Based on these data, we have assigned glycal epoxide **S12** as an α-epoxide (*dr* >20:1) with $^{3}J_{H1-H2}$ = 2.4 Hz and $^{3}J_{H2-H3}$ = 0 Hz. This assignment was further corroborated by the stereochemistry analysis of the glycosylation product **25**.

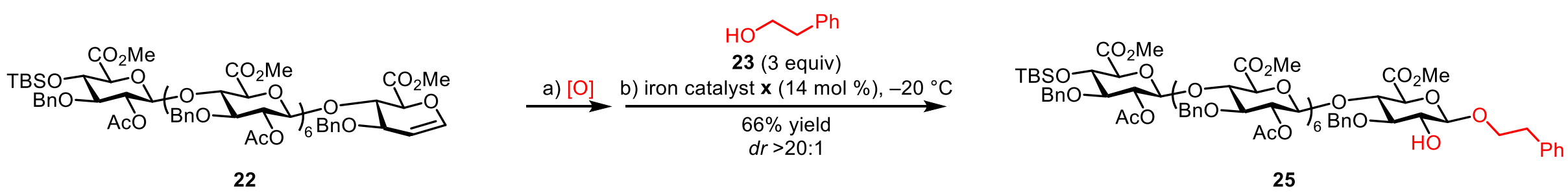

To a flame-dried sealable 2-dram vial equipped with a stir bar were added the aforementioned glycal epoxide **S12** (0.0152 mmol, 1.0 equiv), and freshly activated 5 Å molecular sieves, powder (*ca.* 50 mg). After the flask was evacuated and backfilled with N_2, 2-phenylethanol **23** (5.5 μL, 0.0456 mmol, 3.0 equiv) and anhydrous CH_2Cl_2 (0.05 mL) was added. The iron(III) porphyrin catalyst **1a** (0.00213 mmol, 14 mol %) solution in anhydrous CH_2Cl_2 (0.1 mL) was added to the vial and the resulting mixture was stirred at −40 °C for 5 min. The reaction mixture was kept at –20 °C for 14 h and then quenched with imidazole (3.5 mg) in CH_2Cl_2 (0.5 mL) at the same temperature. The mixture was filtered through a pad of Celite® silica gel and eluted with CH_2Cl_2 (5 mL). The organic layer was then concentrated *in vacuo* and *dr* of the glycosylation product determined based on crude reaction mixture is >20:1. The residue was purified through a silica gel flash column (hexanes/acetone: from 100:1 to 2:1) to afford the desired glycosylation product **25** as white foam (27.8 mg, 66% yield).

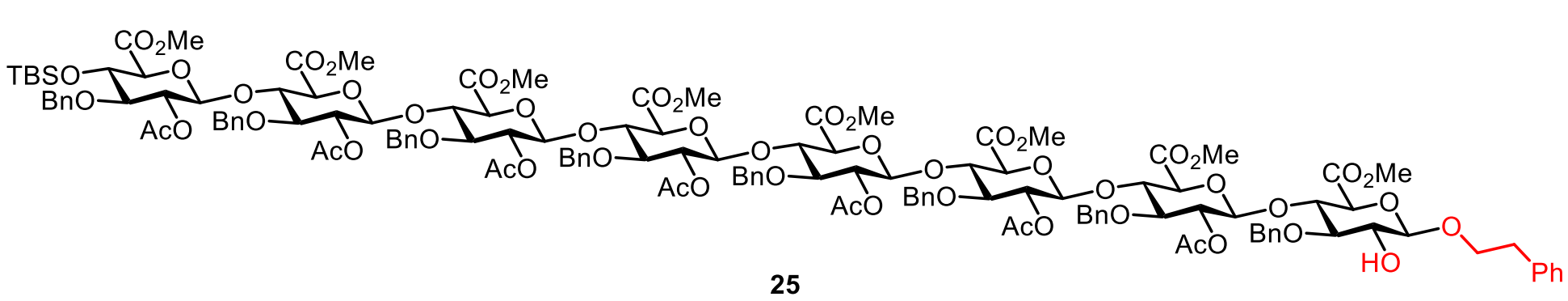

25

Methyl (methyl 2-*O*-acetyl-3-*O*-benzyl-4-*O*-*tert*-butyldimethylsilyl-β-D-glucopyranosyluronate)-(1→4)-(methyl 2-*O*-acetyl-3-*O*-benzyl-β-D-glucopyranosyluronate)-(1→4)-(methyl 2-*O*-acetyl-3-*O*-benzyl-β-D-glucopyranosyluronate)-(1→4)-(methyl 2-*O*-acetyl-3-*O*-benzyl-β-D-glucopyranosyluronate)-(1→4)-(methyl 2-*O*-acetyl-3-*O*-benzyl-β-D-glucopyranosyluronate)-(1→4)-(methyl 2-*O*-acetyl-3-*O*-benzyl-β-D-glucopyranosyluronate)-(1→4)-(methyl 2-*O*-acetyl-3-*O*-benzyl-β-D-glucopyranosyluronate)-(1→4)-(2-phenylethyl 3-*O*-benzyl-β-D-glucopyranosid)uronate (**25**): IR ν_{max} (neat)/cm^{-1}: 2961 (w), 2929 (w), 2856 (w), 1732 (s), 1361 (m), 1222 (s), 1069 (s), 1039 (s), 698 (m); ^{1}H NMR (400 MHz, acetone-d_6) δ 7.46 – 7.37 (m, 2H), 7.35 – 7.21 (m, 43H), 4.99 – 4.86 (m, 8H), 4.86 – 4.75 (m, 8H), 4.75 – 4.67 (m, 8H), 4.57 – 4.44 (m, 8H), 4.07 – 3.94 (m, 13H), 3.97 – 3.90 (m, 3H), 3.89 (d, *J* = 9.6 Hz, 1H), 3.75 (s, 3H), 3.73 – 3.62 (m, 8H), 3.68 (s, 3H), 3.66 (s, 3H), 3.63 (s, 12H), 3.60 (s, 3H), 3.54 – 3.47 (m, 1H), 3.44 – 3.37 (m, 1H), 2.88 (td, *J* = 7.2, 1.5 Hz, 2H), 1.92 (s, 3H), 1.91 (s, 3H), 1.901 (s, 6H), 1.900 (s, 3H), 1.89 (s, 3H), 1.88 (s, 3H), 0.84 (s, 9H), -0.01 (s, 3H), -0.03 (s, 3H); ^{13}C NMR (100 MHz, acetone-d_6) δ 170.0, 169.75, 169.72 (five peaks overlapped, 5C), 169.5, 169.2, 168.94, 168.87, 168.84, 168.82 (three peaks overlapped, 3C), 140.5, 140.0, 139.89 (two peaks overlapped, 2C), 139.87 (three peaks overlapped, 3C), 139.7, 139.3, 129.8 (2C), 129.1 (2C), 129.0 (2C), 128.93 (2C), 128.91 (six peaks overlapped, 12C), 128.80 (2C), 128.77 (2C), 128.7 (five peaks overlapped, 10C), 128.24 (2C), 128.19, 128.08, 128.06 (three peaks overlapped, 3C), 128.0 (two peaks overlapped, 2C), 127.7, 127.0, 104.3, 101.6 (four peaks overlapped, 4C), 101.5 (three peaks overlapped, 3C), 83.3, 83.0, 80.7 (three peaks overlapped, 3C), 80.6 (four peaks overlapped, 4C), 80.0 (five peaks overlapped, 5C), 79.9, 79.6, 77.1, 75.44, 75.40, 75.31 (five peaks overlapped, 5C), 75.27, 75.0 (three peaks overlapped, 3C), 74.9 (three peaks overlapped, 3C), 74.8, 73.9, 73.3, 73.0, 72.89 (three peaks overlapped, 3C), 72.87 (two peaks overlapped, 2C), 71.4, 53.03, 53.00 (four peaks overlapped, 4C), 52.97, 52.8, 52.5, 36.7, 26.1 (3C), 20.99, 20.98, 20.94, 20.92 (four peaks overlapped, 4C), 18.5, -3.7, -5.0; HRMS: m/z (ESI) calcd for $C_{140}H_{166}NaO_{56}Si^{+}$, [M + Na]$^{+}$, 2793.9803, found 2793.9833. $^{1}J_{^{13}C1-H1}$ = 162.1 Hz, 162.1 Hz, 162.1 Hz, 162.1 Hz, 162.1 Hz, 163.1 Hz, 163.1 Hz, 162.7 Hz.

The stereochemistry of newly formed anomeric center (C1) of **25** was determined by measuring $^{1}J^{13}_{C1–H1}$ (162.7 Hz) through un-decoupled HSQC experiments, which suggested the newly formed glycosidic bond is in equatorial position. The C2 stereochemistry was determined by measuring $^{3}J_{H1–H2}$ (7.5 Hz), which suggested the H2 is in axial position and corroborated the exclusive formation of the α-epoxide in glycal epoxidation.

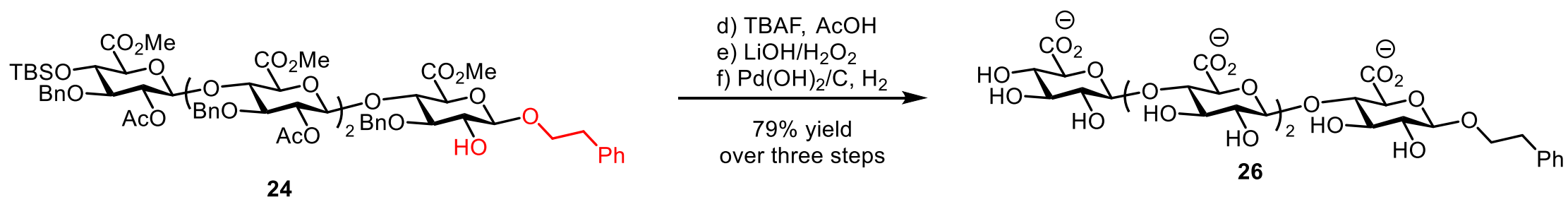

To a flame-dried sealable 2-dram vial equipped with a stir bar were added **24** (102.8 mg, 0.067 mmol, 1.0 equiv) and THF (0.1 mL). The flask was cooled to 0 °C, and a premixed solution of TBAF (1.0 M in THF, 0.2 mL, 0.20 mmol, 3.0 equiv) and AcOH (1.0 M in THF, 0.2 mL, 0.20 mmol, 3.0 equiv) was added. The reaction mixture was stirred for 24 h at room temperature, with progress monitored by TLC until completion. The reaction mixture was diluted with EtOAc (5 mL) and then quenched with saturated aqueous NH_4Cl solution (10 mL). The organic phase was separated from the aqueous phase, which was extracted with EtOAc (10 mL × 4). The combined organic phase was washed with brine (10 mL) and dried over Na_2SO_4. The desilylation product was isolated through column chromatography (hexanes/acetone: from 20:1 to 3:2) as white foam (82.1 mg, 87% yield).

To flame-dried sealable 2-dram vial equipped with a stir bar were added the aforementioned product (24.0 mg, 0.0176 mmol, 1.0 equiv) and THF (0.6 mL). The flask was cooled to 0 °C, and a premixed solution of LiOH (1.0 M in H_2O, 0.438 mL, 0.438 mmol, 25.0 equiv) and H_2O_2 (30 wt % in H_2O, 135 μL, 1.31 mmol, 75.0 equiv) was added. The reaction mixture was stirred for 12 h at room temperature. The reaction mixture was diluted with H_2O (3 mL), cooled to 0 °C, quenched with saturated aqueous $NaHSO_3$ solution and the progress was monitored using MQuant® Peroxide Test strips. The organic phase was separated from the aqueous phase, which was extracted with EtOAc (2 mL × 4). The organic phase was concentrated *in vacuo* to afford the hydrolysis product (20.7 mg, quantitative yield) as a white solid.

To the hydrolysis product azeotropically dried with toluene (1.0 mL × 3), $Pd(OH)_2/C$ (30 wt %, 6.2 mg) was added. After the vial was evacuated and backfilled with N_2, MeOH/NaH_2PO_4-Na_2HPO_4 buffer (*p*H = 7, 1:1, 1.2 mL) was added. The vial was then evacuated and backfilled with H_2 three times. After the mixture was stirred for 12 h at room temperature, the reaction mixture was then filtered through cotton (eluted with H_2O), and the filtrate was concentrated *in vacuo*. The desired product **26** was purified with Sephadex G-25 column chromatography (eluted with H_2O) as white foam (13.2 mg, 91% yield, 79% yield over three steps from **24**).

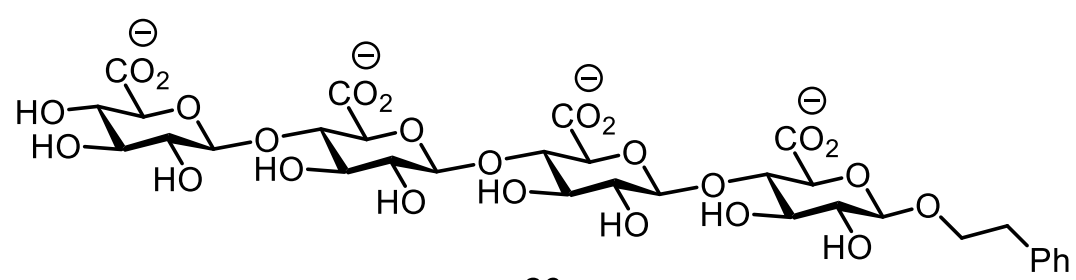

26

2-Phenylethyl (β-D-glucopyranosyluronic acid)-(1→4)-(β-D-glucopyranosyluronic acid)-(1→4)-(β-D-glucopyranosyluronic acid)-(1→4)-β-D- glucopyranosyluronic acid (**26**): IR ν_{max} (neat)/cm^{-1}: 3458 (s), 2930 (m), 2633 (w), 1743 (s), 1621 (s), 1415 (m), 1126 (s), 1035 (s), 614 (m); ^{1}H NMR (400 MHz, D_2O) δ 7.45 – 7.26 (m, 5H), 4.55 – 4.46 (m, 4H), 4.18 – 4.10 (m, 1H), 3.97 – 3.84 (m, 3H), 3.80 (d, *J* = 9.5 Hz, 1H), 3.75 (d, *J* = 9.0 Hz, 1H), 3.71 – 3.56 (m, 6H), 3.55 – 3.46 (m, 2H), 3.42 – 3.27 (m, 4H), 2.96 (t, *J* = 6.9 Hz, 2H); ^{13}C NMR (100 MHz, D_2O) δ 175.6, 175.1, 175.0, 174.9, 138.7, 129.0 (2C), 128.6 (2C), 126.5, 102.4, 102.2 (two peaks overlapped, 2C), 101.9, 80.9, 80.8, 80.7, 75.8, 75.6, 75.3 (two peaks overlapped, 2C), 75.2, 74.3, 74.2, 74.1, 73.0, 72.7 (two peaks overlapped, 2C), 72.5, 71.6, 70.8, 35.1; HRMS: m/z (ESI) calcd for $C_{32}H_{41}O_{25}^-$, $[M – H]^-$, 825.1942, found 825.1961.

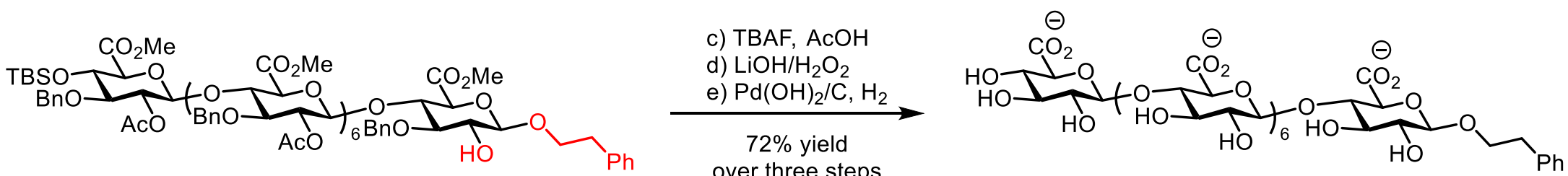

25 **27**

To a flame-dried sealable 2-dram vial equipped with a stir bar were added **25** (21.1 mg, 0.00756 mmol, 1.0 equiv) and THF (0.05 mL). The flask was cooled to 0 °C, and a premixed solution of TBAF (1.0 M in THF, 23 μL, 0.0227 mmol, 3.0 equiv) and AcOH (1.0 M in THF, 23 μL, 0.0227

mmol, 3.0 equiv) was added. The reaction mixture was stirred for 24 h at room temperature, with progress monitored by TLC until completion. The reaction mixture was diluted with EtOAc (3 mL) and then quenched with saturated aqueous NH_4Cl solution (5 mL). The organic phase was separated from the aqueous phase, which was extracted with EtOAc (5 mL × 4). The combined organic phase was washed with brine (5 mL) and dried over Na_2SO_4. The desilylation was isolated through column chromatography (hexanes/acetone: from 20:1 to 1:1) as white foam (16.2 mg, 81% yield).

To flame-dried sealable 2-dram vial equipped with a stir bar were added the aforementioned desilylation product (10.6 mg, 0.00399 mmol, 1.0 equiv) and THF (0.2 mL). The flask was cooled to 0 °C, and a premixed solution of LiOH (1.0 M in H_2O, 0.20 mL, 0.20 mmol, 50.0 equiv) and H_2O_2 (30 wt % in H_2O, 62 μL, 0.598 mmol, 150.0 equiv) was added. The reaction mixture was stirred for 12 h at room temperature. The reaction mixture was diluted with H_2O (1 mL), cooled to 0 °C, quenched with saturated aqueous $NaHSO_3$ solution and the progress was monitored using MQuant® Peroxide Test strips. The organic phase was separated from the aqueous phase, which was extracted with EtOAc (2 mL × 4). The organic phase was concentrated *in vacuo* to afford the hydrolysis product (9.0 mg, quantitative yield) as white foam.

To the hydrolysis product was azeotropically dried with toluene (1.0 mL × 3), $Pd(OH)_2/C$ (30 wt %, 2.7 mg) was added. After the vial was evacuated and backfilled with N_2, MeOH/ NaH_2PO_4-Na_2HPO_4 buffer (*p*H = 7, 1:1, 0.4 mL) was added. The vial was then evacuated and backfilled with H_2 three times. After the mixture was stirred for 12 h at room temperature, the reaction mixture was then filtered through cotton (eluted with H_2O), and the filtrate was concentrated *in vacuo*. The desired product **27** was purified by Sephadex G-25 column chromatography (eluted with H_2O) as white foam (5.4 mg, 89% yield, 72% yield over three steps from **25**).

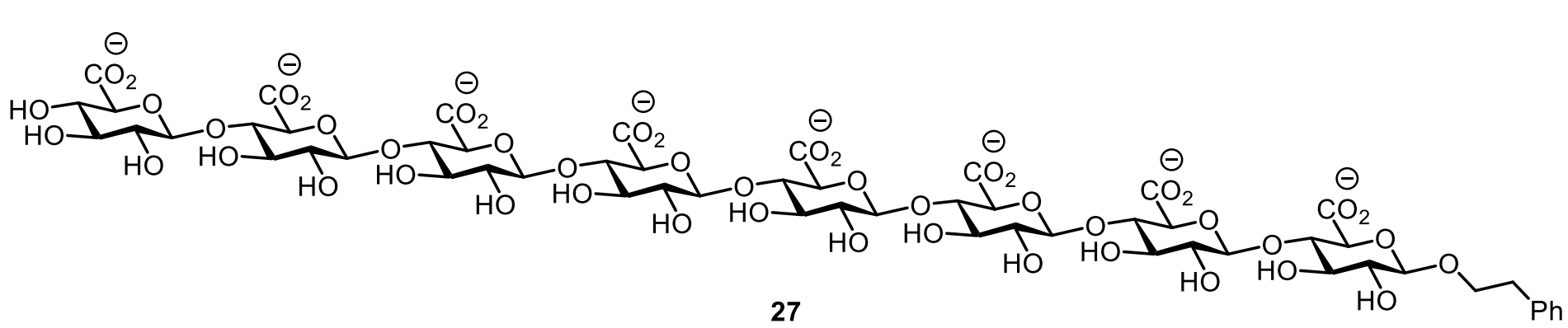

27

2-Phenylethyl (β-D-glucopyranosyluronic acid)-(1→4)-(β-D-glucopyranosyluronic acid)-(1→4)-(β-D-glucopyranosyluronic acid)-(1→4)-(β-D-glucopyranosyluronic acid)-(1→4)-(β-D-glucopyranosyluronic acid)-(1→4)-(β-D-glucopyranosyluronic acid)-(1→4)-(β-D-glucopyranosyluronic acid)-(1→4)-β-D- glucopyranosyluronic acid (**27**): IR ν_{max} (neat)/cm^{-1}: 3466 (s), 2953 (m), 2639 (w), 1744 (s), 1621 (s), 1414 (m), 1130 (s), 1036 (s), 622 (m); ^{1}H NMR (400 MHz, D_2O) δ 7.26 – 7.18 (m, 4H), 7.16 – 7.11 (m, 1H), 4.45 – 4.25 (m, 8H), 4.03 – 3.94 (m, 1H), 3.82 – 3.69 (m, 7H), 3.65 (d, *J* = 9.5 Hz, 1H), 3.60 (d, *J* = 9.1 Hz, 1H), 3.56 – 3.41 (m, 14H), 3.40 – 3.30 (m, 2H), 3.27 – 3.10 (m, 8H), 2.81 (t, *J* = 6.9 Hz, 2H); ^{13}C NMR (100 MHz, D_2O) δ 174.8 (eight peaks overlapped, 8C), 138.7, 129.0 (2C), 128.6 (2C), 126.5, 102.4, 102.3 (six peaks overlapped, 6C), 102.0, 80.92, 80.85, 80.7 (five peaks overlapped, 5C), 75.8, 75.6, 75.2 (five peaks overlapped, 5C), 74.3, 74.2, 74.1 (five peaks overlapped, 5C), 73.1, 72.7 (eight peaks overlapped, 8C), 72.5, 71.6, 70.8, 35.1; HRMS: m/z (ESI) calcd for $C_{56}H_{73}O_{49}^{-}$, $[M – H]^{-}$, 1529.3226, found 1529.3258 & m/z (ESI) calcd for $C_{56}H_{72}O_{49}^{2-}$, $[M – 2H]^{2-}$, 764.1577, found 764.1592.

F. References

1. Zhang, X.-W.; Yin, L.; Zhang, D.; Jiang, Z.; Wang, P.; Xu, H., Iron-Catalyzed Highly Stereospecific Glycosylation with Glycal Epoxides. *Angew. Chem. Int. Ed.* **2025**, *64*, e202517634.

2. Cheshev, P.; Marra, A.; Dondoni, A., Direct Epoxidation of D-Glucal and D-Galactal Derivatives with in situ Generated DMDO. *Carbohydr. Res.* **2006**, *341*, 2714–2716.

3. Eby, R.; Srivastava, V. K., Conformational analysis of 1,2-anhydro-3,4,6-tri-O-benzyl-α-d-glucopyranose and -β-d-mannopyranose. *Carbohydr. Res.* **1982**, *102*, 1-9.

4. Zhang, D.; Jiang, Z.; Yin, L.; Zhang, X.-W.; Yang, H.; Wang, P.; Xie, C.; Stancanelli, E.; Xu, Y.; Wang, H.; Sun, J.; Liu, J.; Xu, H., Merging Enzymatic Catalysis with Iron Catalysis for Highly Stereoselective Heparan Sulfate Oligosaccharide Assembly. *J. Am. Chem. Soc.* **2026**, *148*, 1766-1778.

5. Taber, D. F.; DeMatteo, P. W.; Hassan, R. A., Simplified Preparation of Dimethyldioxirane (DMDO). In *Organic Syntheses*, pp 350-357.

6. Zhang, X.-W.; Zhang, D.; Jiang, Z.; Xu, H., Iron-Catalyzed Stereospecific Heterocycle N-Glycosylation with Glycal Epoxides. *Org. Lett.* **2026**, *28*, 2859-2864.

7. Li, H.; Zhang, D.; Li, C.; Yin, L.; Jiang, Z.; Luo, Y.; Xu, H., Stereoselective Glycosylation for 1,2-*cis*-Aminoglycoside Assembly by Cooperative Atom Transfer Catalysis. *J. Am. Chem. Soc.* **2024**, *146*, 33316–33323.

G. NMR Spectra

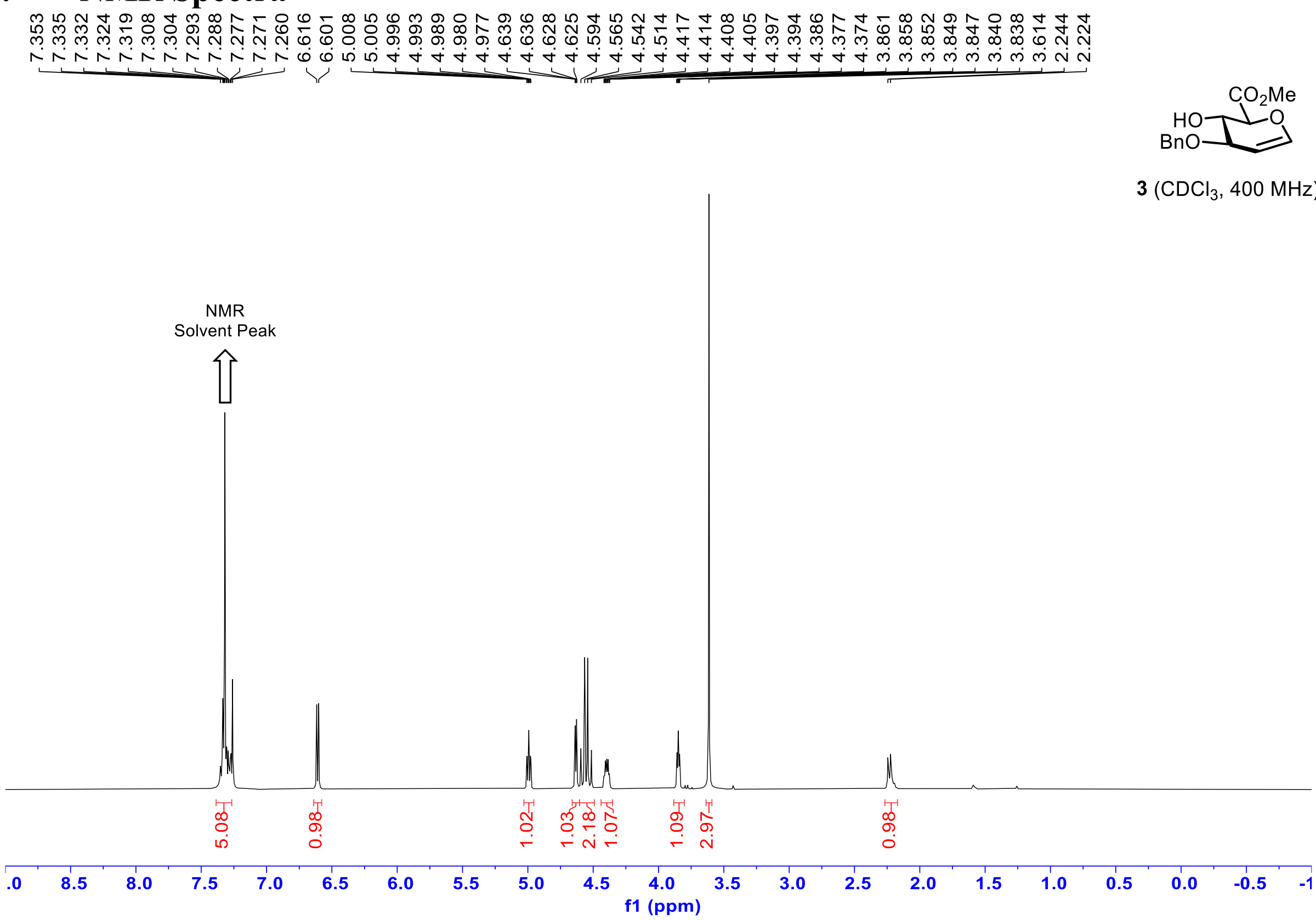

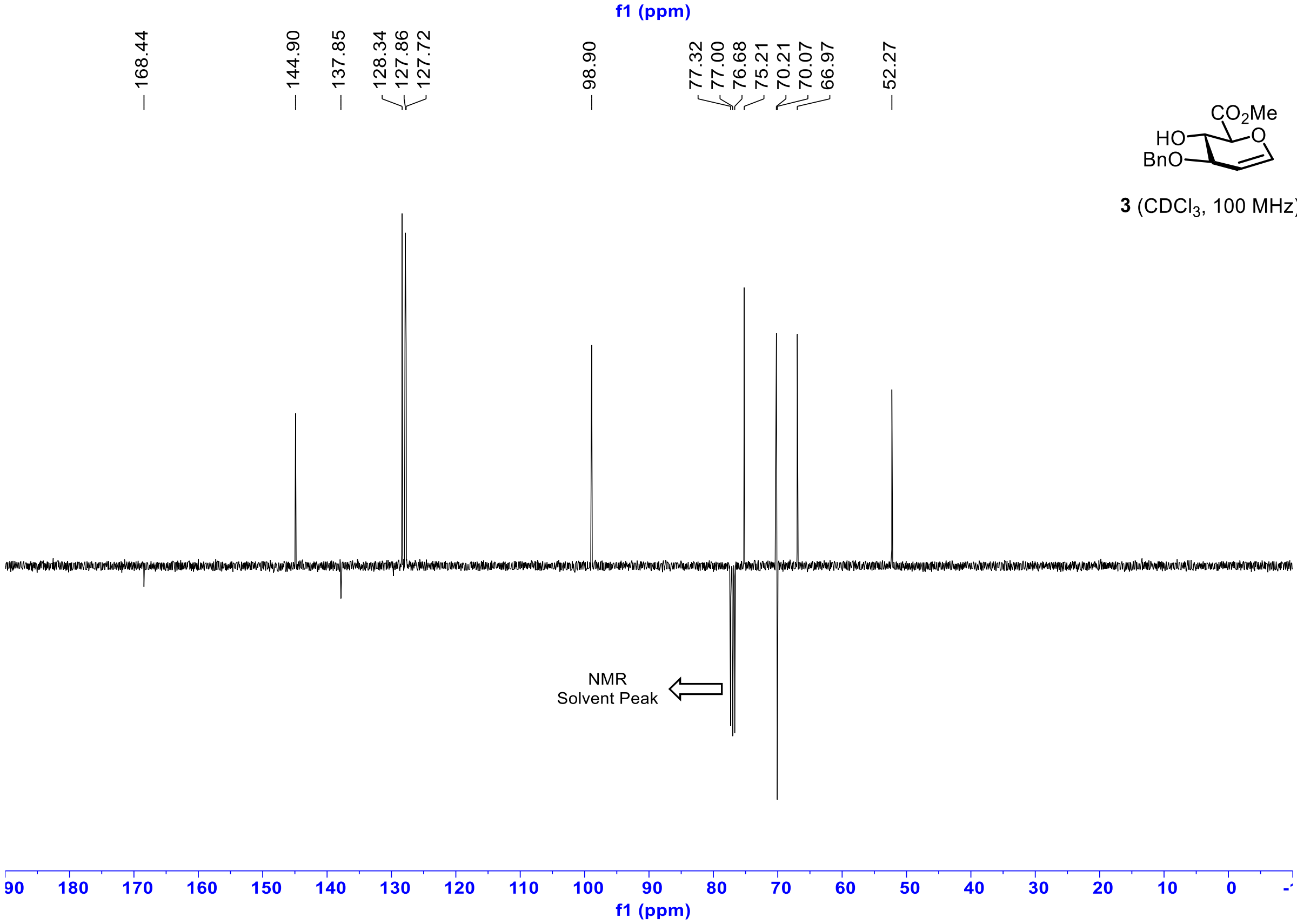

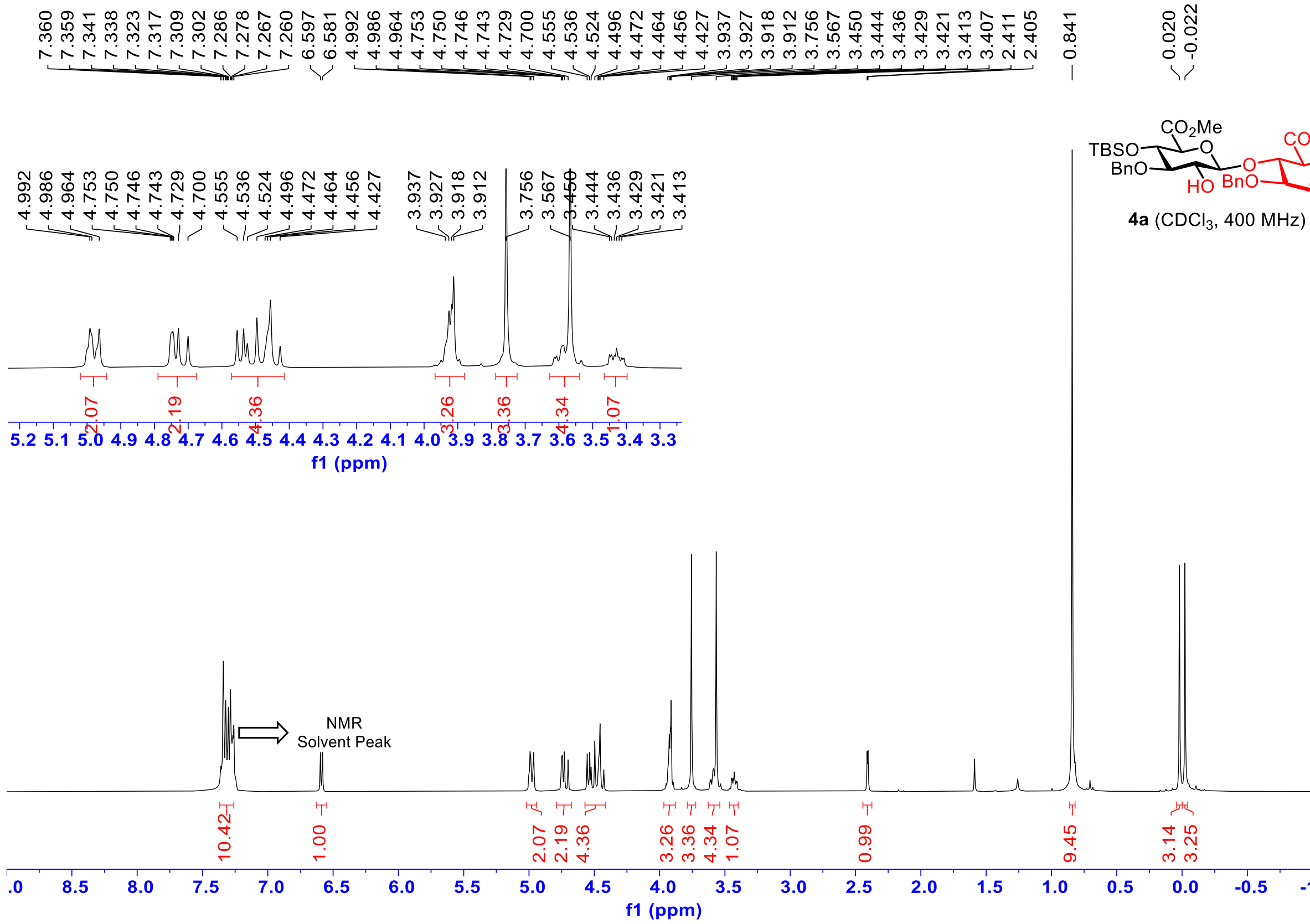

4a (CDCl$_3$, 400 MHz)
NMR Solvent Peak
f1 (ppm)

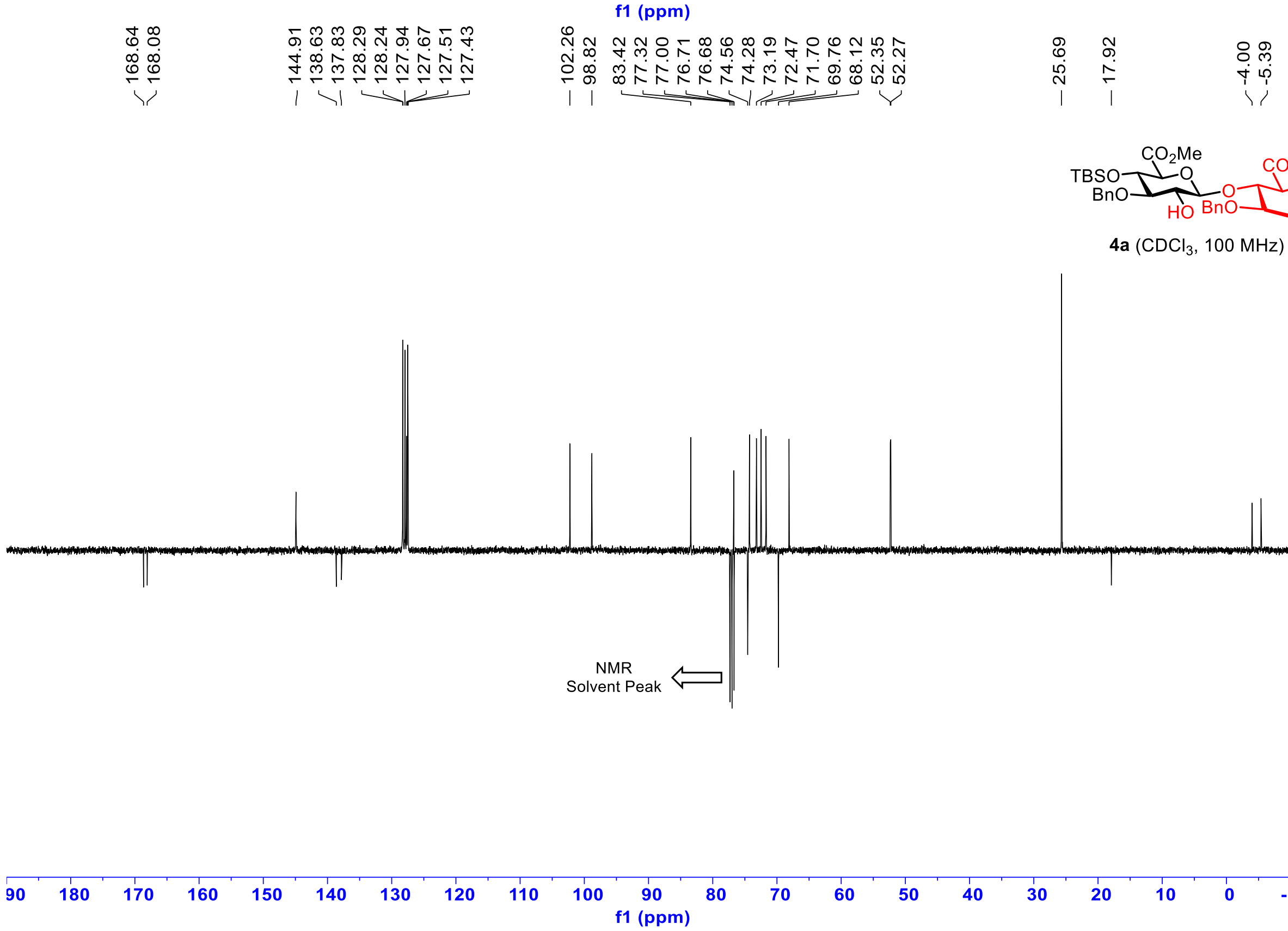

4a (CDCl$_3$, 100 MHz)
NMR Solvent Peak
f1 (ppm)

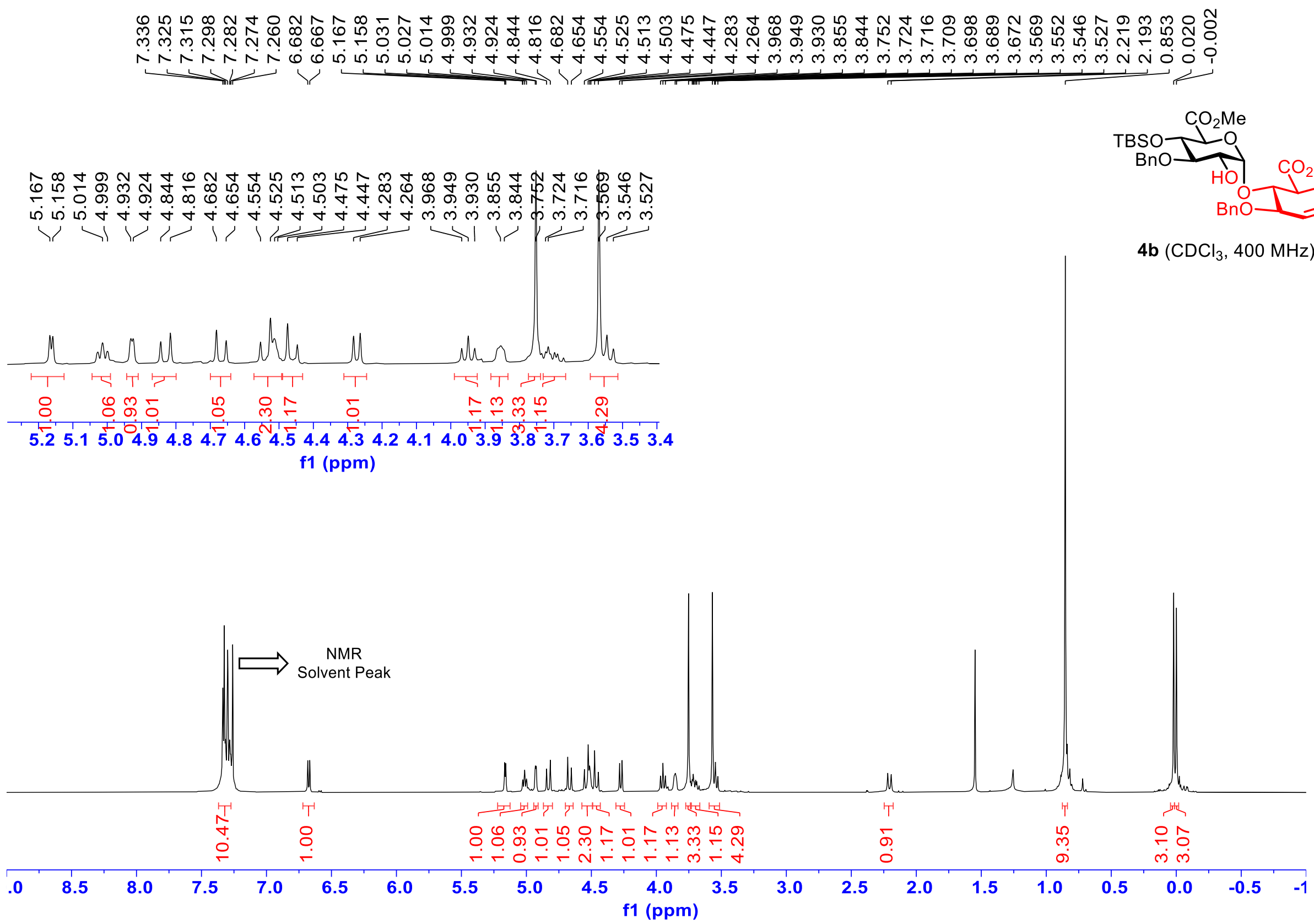
7.336
7.325
7.315
7.298
7.282
7.274
7.260
6.682
6.667
5.167
5.158
5.031
5.027
5.014
4.999
4.932
4.924
4.844
4.816
4.682
4.654
4.554
4.525
4.513
4.503
4.475
4.447
4.283
4.264
3.968
3.949
3.930
3.855
3.844
3.752
3.724
3.716
3.709
3.698
3.689
3.672
3.569
3.552
3.546
3.527
2.219
2.193
0.853
0.020
-0.002
TBSO
BnO
CO2Me
HO
O
BnO
4b (CDCl3, 400 MHz)
NMR
Solvent Peak
f1 (ppm)

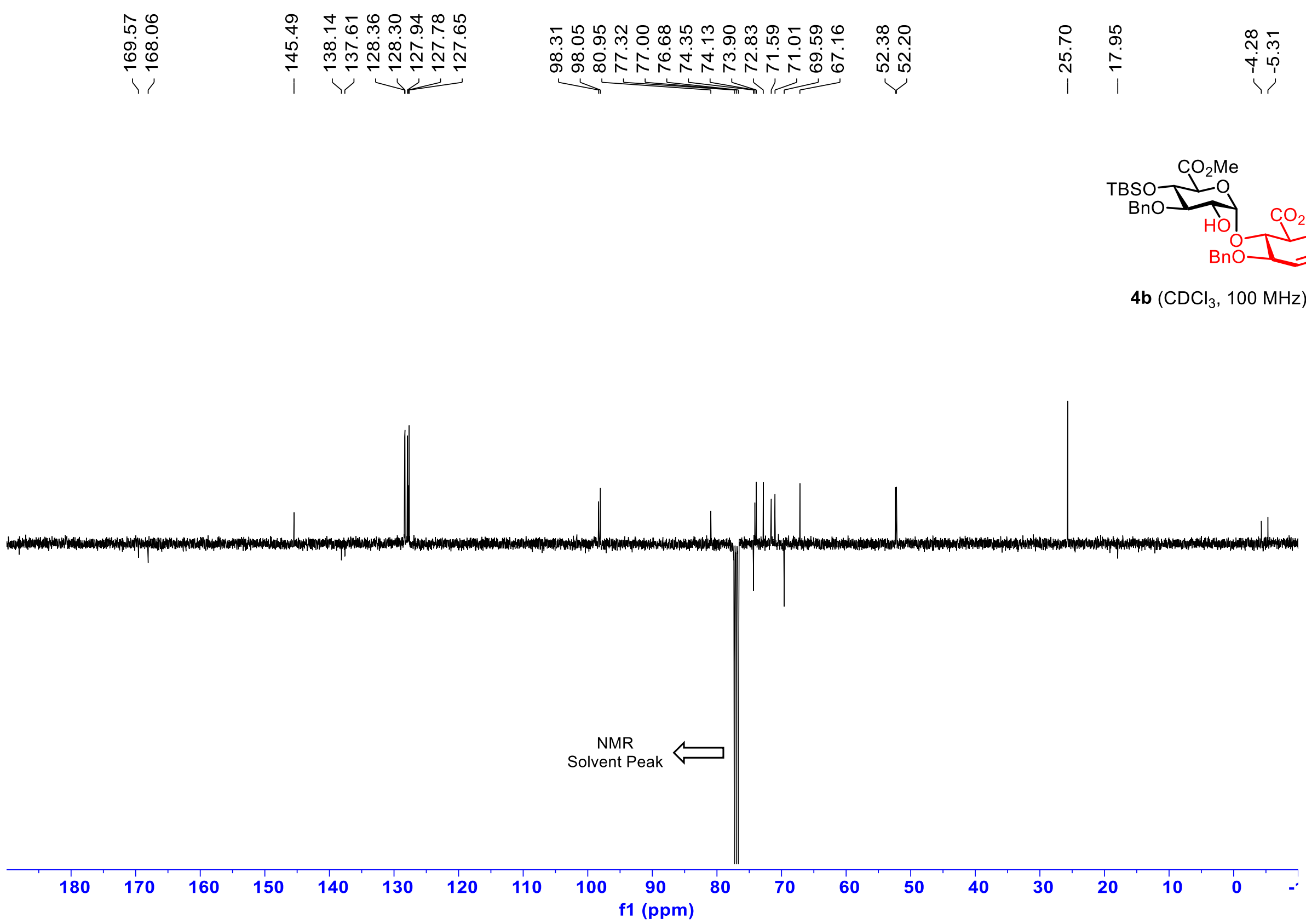
169.57
168.06
145.49
138.14
137.61
128.36
128.30
127.94
127.78
127.65
98.31
98.05
80.95
77.32
77.00
76.68
74.35
74.13
73.90
72.83
71.59
71.01
69.59
67.16
52.38
52.20
25.70
17.95
-4.28
-5.31
TBSO
BnO
CO2Me
HO
O
BnO
4b (CDCl3, 100 MHz)
NMR
Solvent Peak
f1 (ppm)

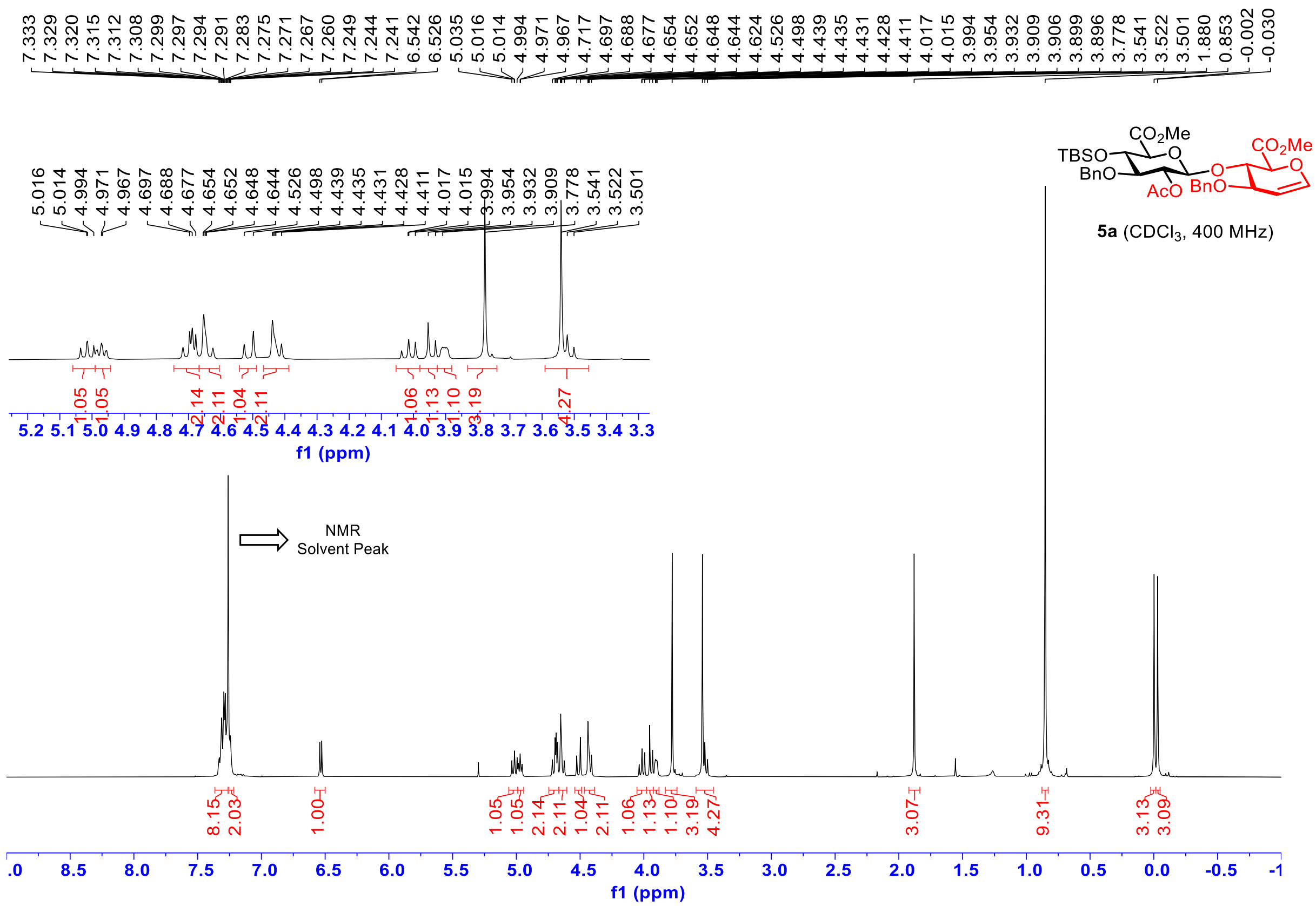
7.333 7.329 7.320 7.315 7.312 7.308 7.299 7.297 7.294 7.291 7.283 7.275 7.271 7.267 7.260 7.249 7.244 7.241 6.542 6.526 5.035 5.016 5.014 4.994 4.971 4.967 4.717 4.697 4.688 4.677 4.654 4.652 4.648 4.644 4.624 4.526 4.498 4.439 4.435 4.431 4.428 4.411 4.017 4.015 3.994 3.954 3.932 3.909 3.906 3.899 3.896 3.778 3.541 3.522 3.501 1.880 0.853 -0.002 -0.030
TBSO
BnO
CO2Me
AcO
BnO
CO2Me
5a (CDCl3, 400 MHz)
NMR
Solvent Peak
f1 (ppm)

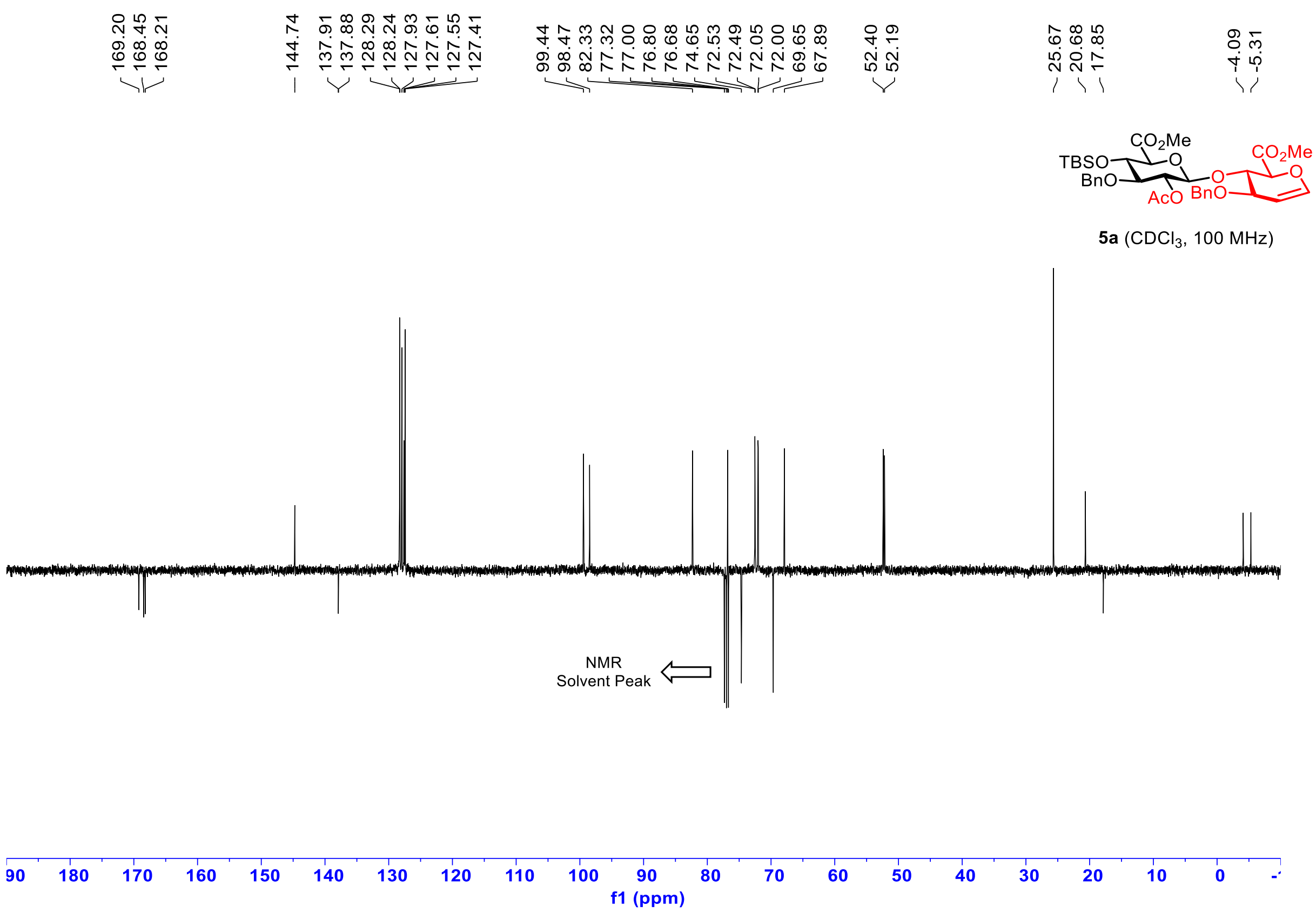
169.20 168.45 168.21 144.74 137.91 137.88 128.29 128.24 127.93 127.61 127.55 127.41 99.44 98.47 82.33 77.32 77.00 76.80 76.68 74.65 72.53 72.49 72.05 72.00 69.65 67.89 52.40 52.19 25.67 20.68 17.85 -4.09 -5.31
TBSO
BnO
CO2Me
AcO
BnO
CO2Me
5a (CDCl3, 100 MHz)
NMR
Solvent Peak
f1 (ppm)

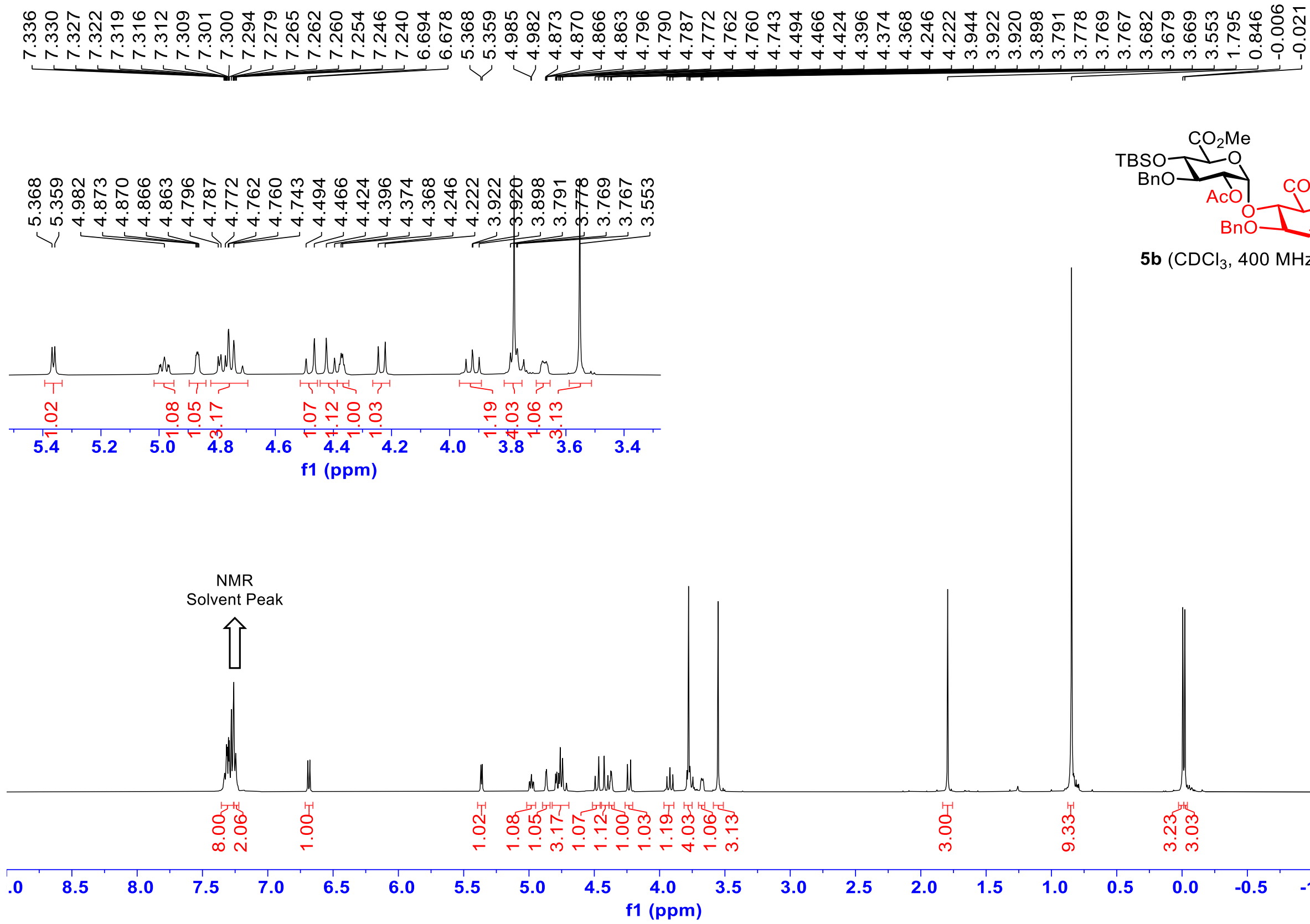

5b ($CDCl_3$, 400 MHz)

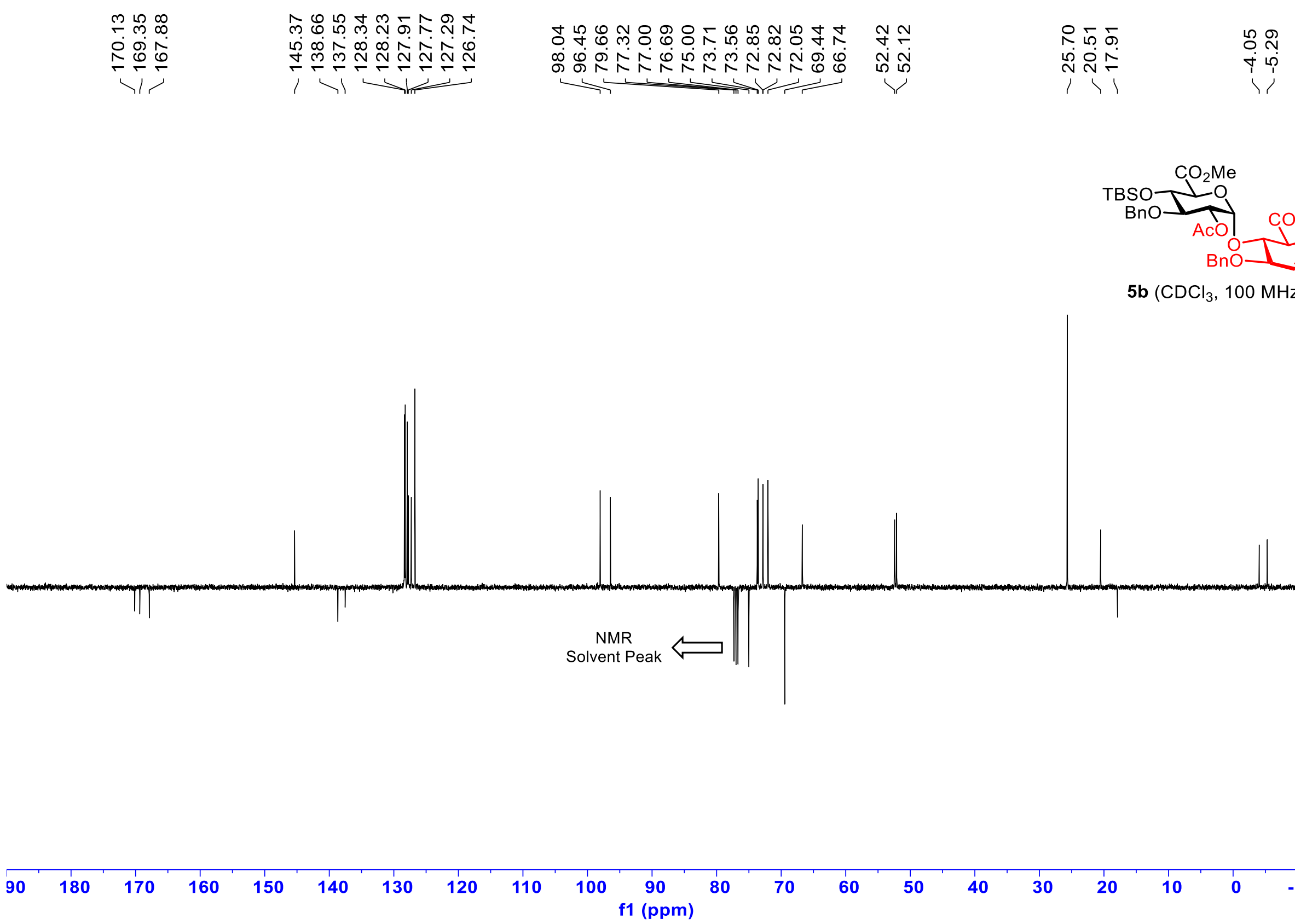

5b ($CDCl_3$, 100 MHz)

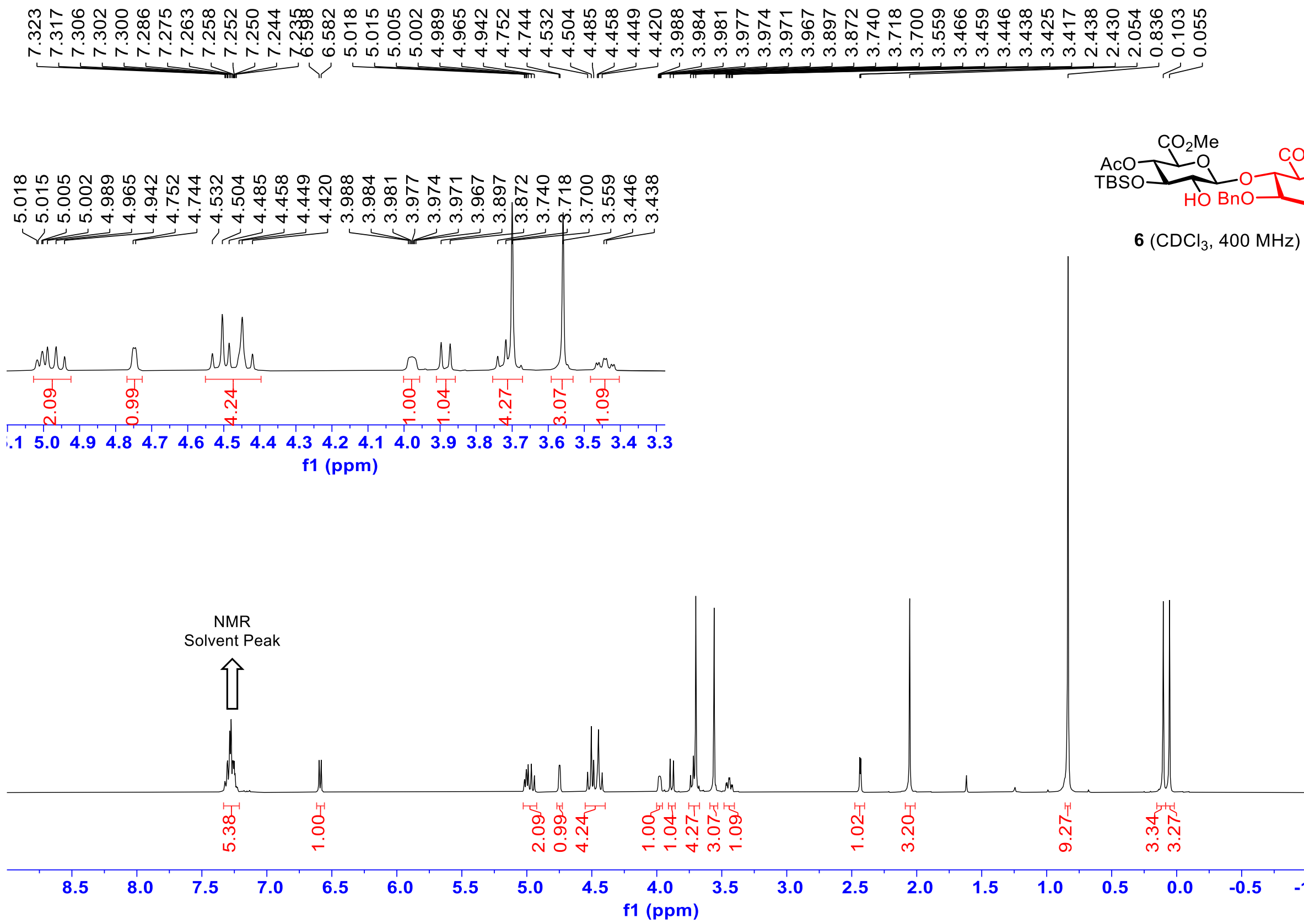
7.323 7.317 7.306 7.302 7.300 7.286 7.275 7.263 7.258 7.252 7.250 7.244 7.235 6.588 6.582 5.018 5.015 5.005 5.002 4.989 4.965 4.942 4.752 4.744 4.532 4.504 4.485 4.458 4.449 4.420 3.988 3.984 3.981 3.977 3.974 3.971 3.967 3.897 3.872 3.740 3.718 3.700 3.559 3.466 3.459 3.446 3.438 3.425 3.417 2.438 2.430 2.054 0.836 0.103 0.055
CO2Me
AcO
TBSO
O
HO
BnO
CO2Me
O
6 (CDCl3, 400 MHz)
NMR
Solvent Peak
f1 (ppm)

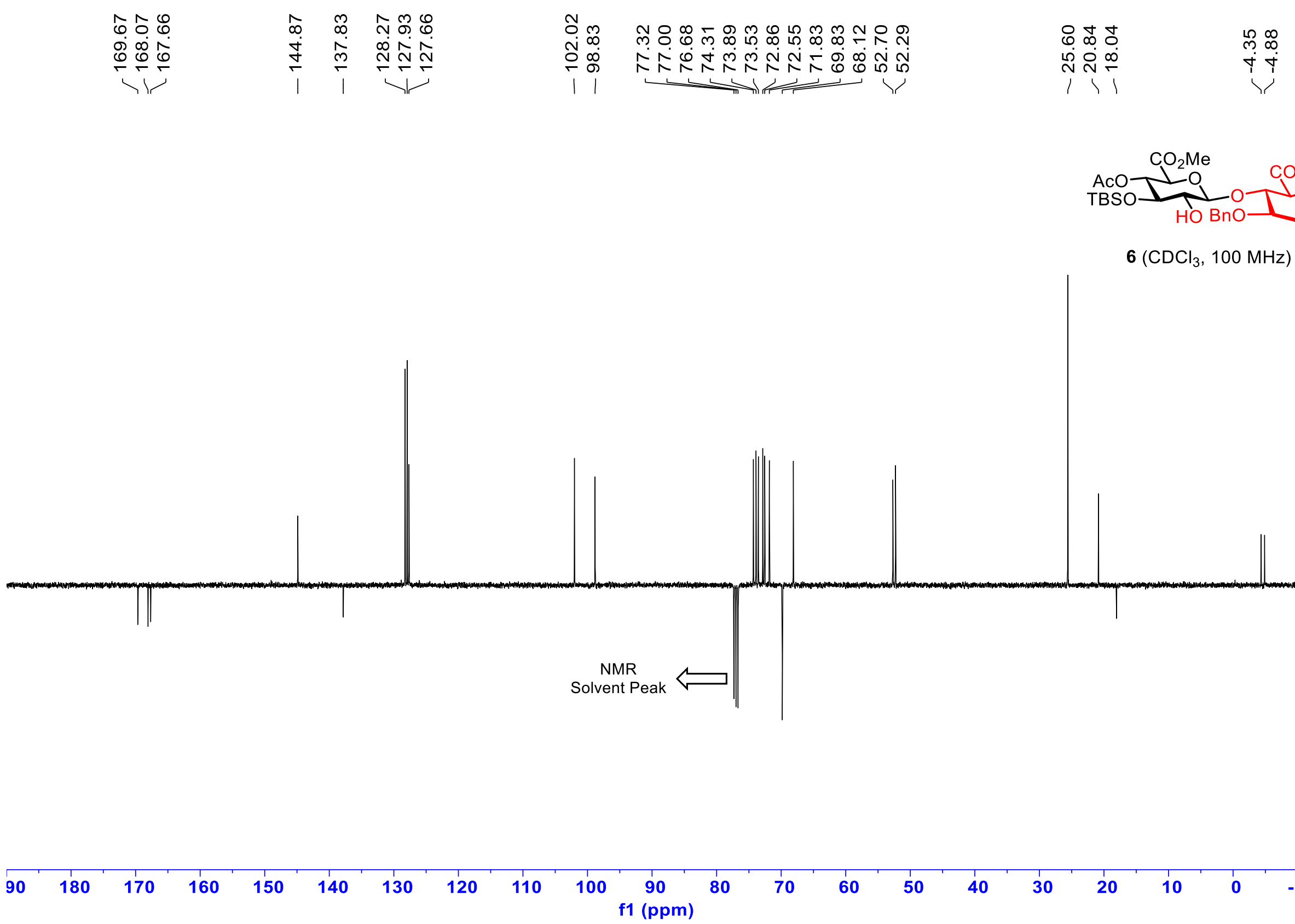
169.67 168.07 167.66 144.87 137.83 128.27 127.93 127.66 102.02 98.83 77.32 77.00 76.68 74.31 73.89 73.53 72.86 72.55 71.83 69.83 68.12 52.70 52.29 25.60 20.84 18.04 -4.35 -4.88
CO2Me
AcO
TBSO
O
HO
BnO
CO2Me
O
6 (CDCl3, 100 MHz)
NMR
Solvent Peak
f1 (ppm)

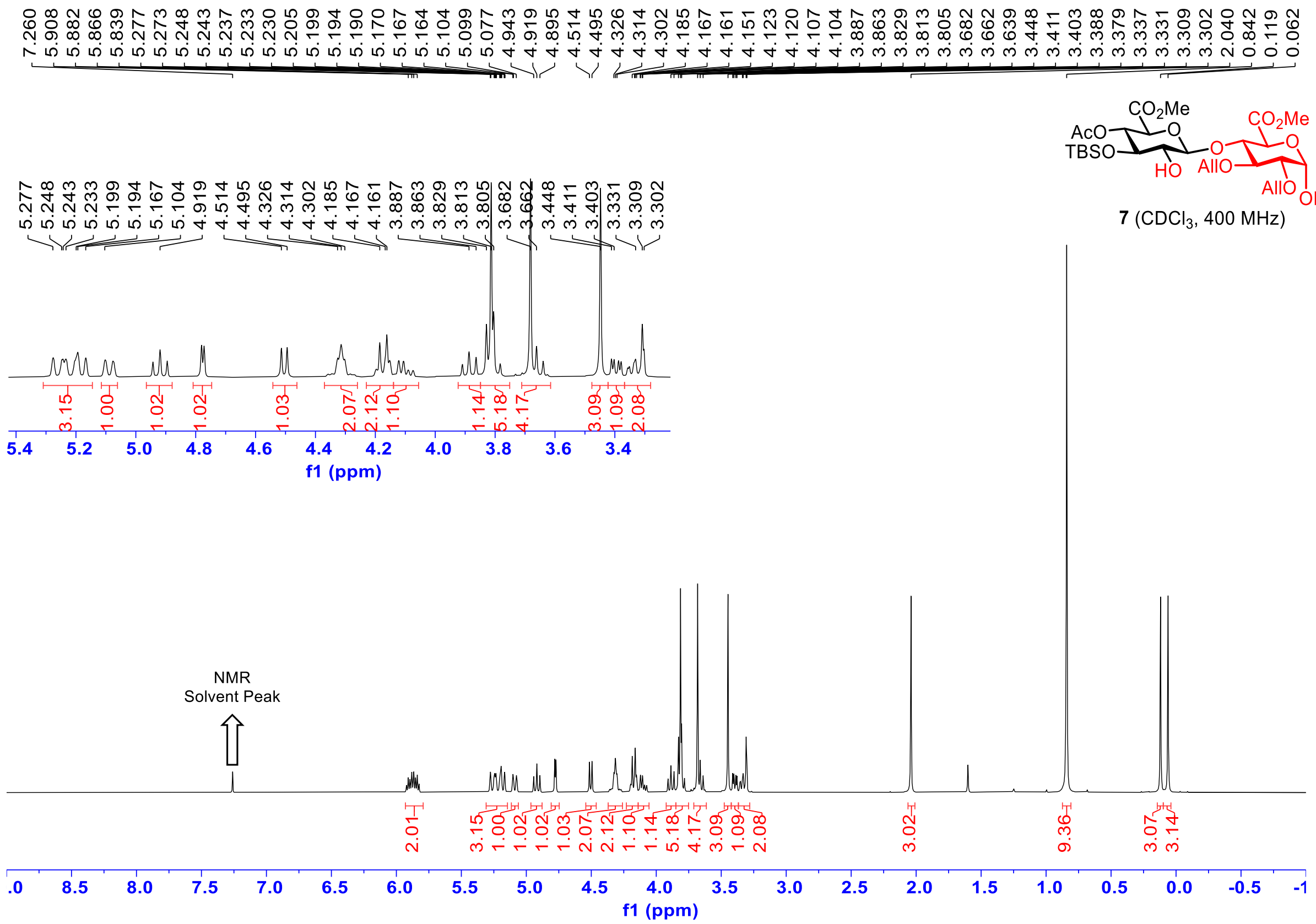
7.260 5.908 5.882 5.866 5.839 5.277 5.273 5.248 5.243 5.237 5.233 5.230 5.205 5.199 5.194 5.190 5.170 5.167 5.164 5.104 5.099 5.077 4.943 4.919 4.895 4.514 4.495 4.326 4.314 4.302 4.185 4.167 4.161 4.151 4.123 4.120 4.107 4.104 3.887 3.863 3.829 3.813 3.805 3.682 3.662 3.639 3.448 3.411 3.403 3.388 3.379 3.337 3.331 3.309 3.302 2.040 0.842 0.119 0.062
AcO
TBSO
CO2Me
HO
CO2Me
AllO
AllO
OMe
7 (CDCl3, 400 MHz)
NMR
Solvent Peak
f1 (ppm)
f1 (ppm)

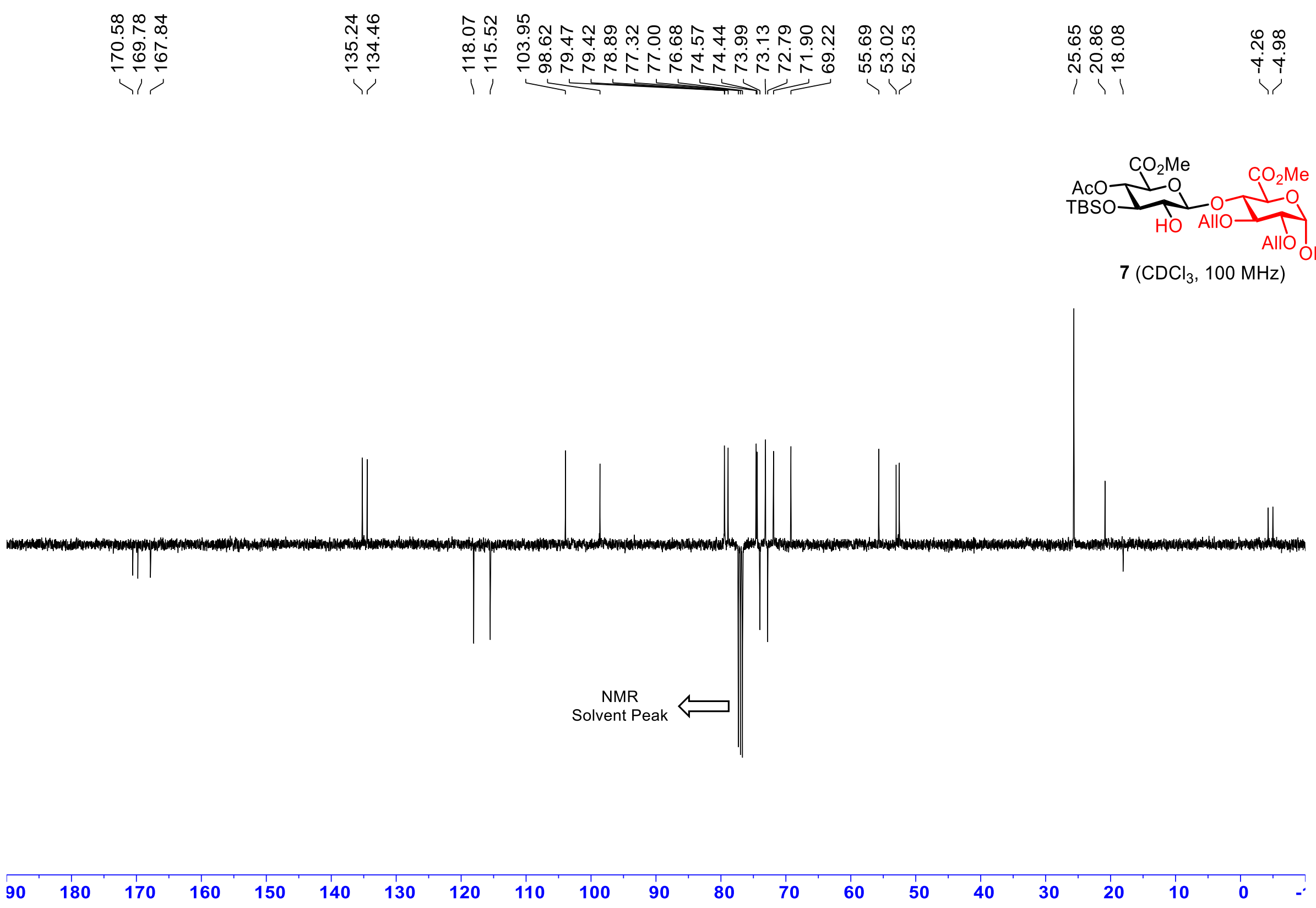
170.58 169.78 167.84 135.24 134.46 118.07 115.52 103.95 98.62 79.47 79.42 78.89 77.32 77.00 76.68 74.57 74.44 73.99 73.13 72.79 71.90 69.22 55.69 53.02 52.53 25.65 20.86 18.08 -4.26 -4.98
AcO
TBSO
CO2Me
HO
CO2Me
AllO
AllO
OMe
7 (CDCl3, 100 MHz)
NMR
Solvent Peak
f1 (ppm)

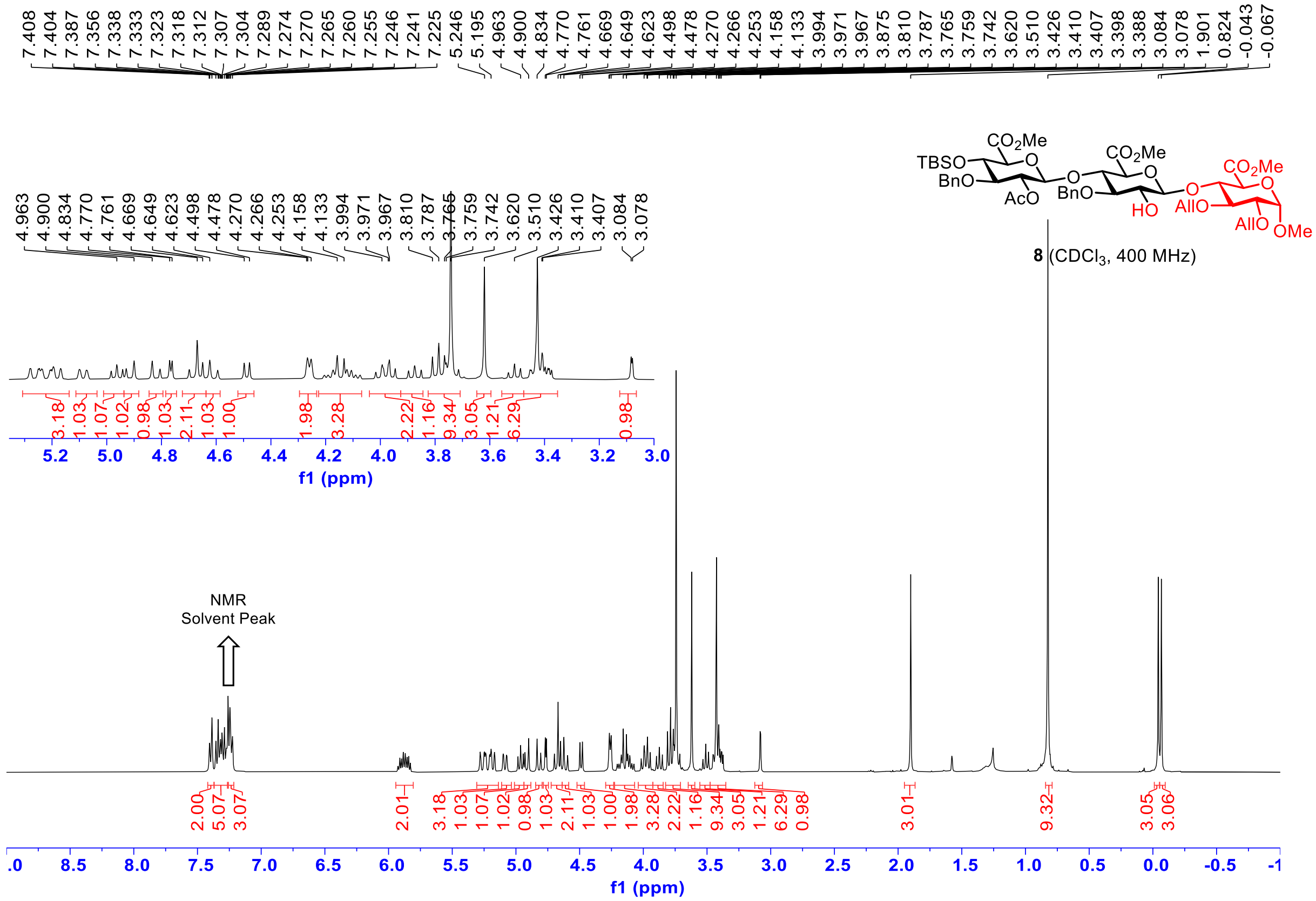
7.408 7.404 7.387 7.356 7.338 7.333 7.323 7.318 7.312 7.307 7.304 7.289 7.274 7.270 7.265 7.260 7.255 7.246 7.241 7.225 5.246 5.195 4.963 4.900 4.834 4.770 4.761 4.669 4.649 4.623 4.498 4.478 4.270 4.266 4.253 4.158 4.133 3.994 3.971 3.967 3.875 3.810 3.787 3.765 3.759 3.742 3.620 3.510 3.426 3.410 3.407 3.398 3.388 3.084 3.078 1.901 0.824 -0.043 -0.067
TBSO
BnO
CO2Me
AcO
BnO
HO
AllO
AllO
OMe
8 (CDCl3, 400 MHz)
NMR Solvent Peak
f1 (ppm)
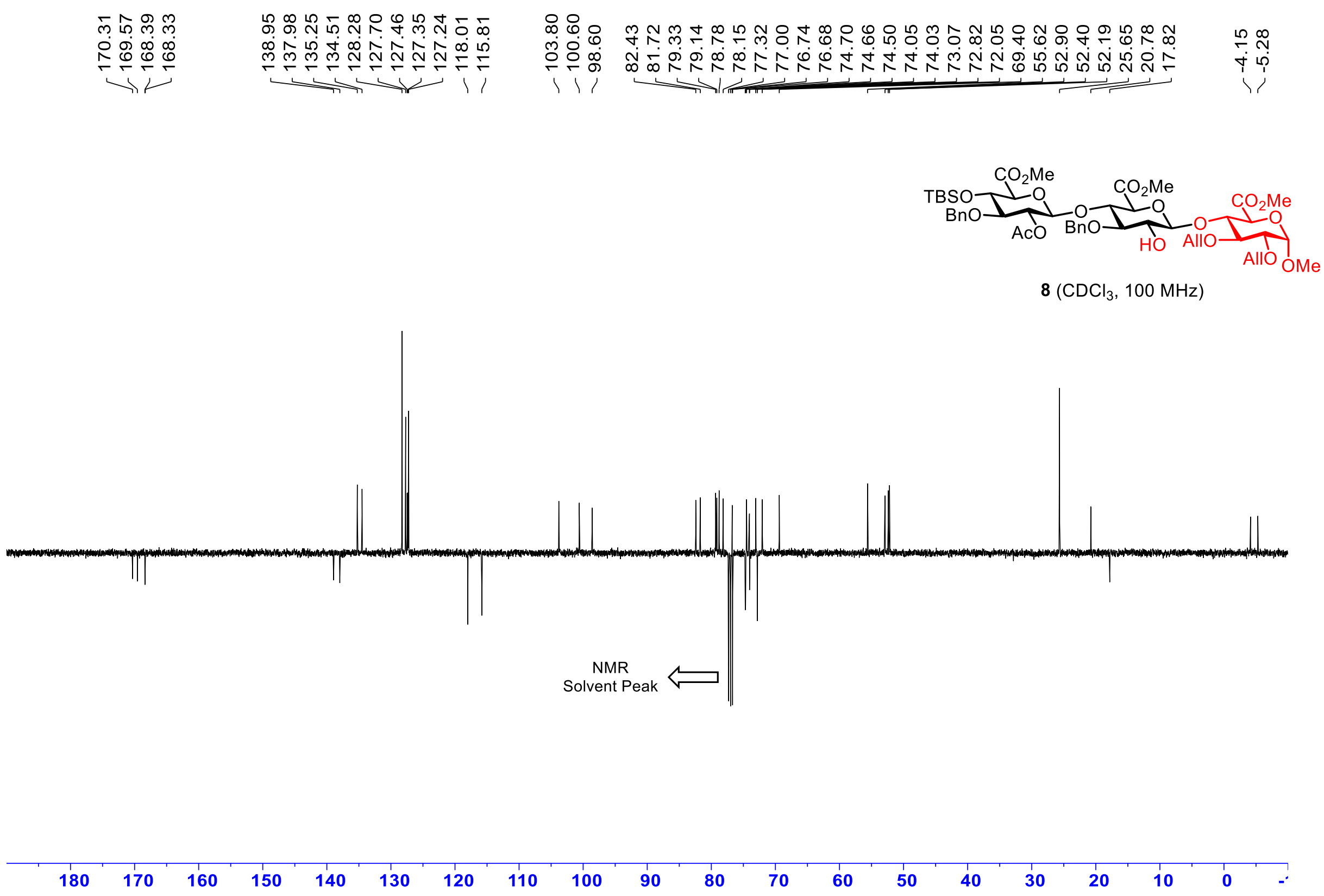
170.31 169.57 168.39 168.33 138.95 137.98 135.25 134.51 128.28 127.70 127.46 127.35 127.24 118.01 115.81 103.80 100.60 98.60 82.43 81.72 79.33 79.14 78.78 78.15 77.32 77.00 76.74 76.68 74.70 74.66 74.50 74.05 74.03 73.07 72.82 72.05 69.40 55.62 52.90 52.40 52.19 25.65 20.78 17.82 -4.15 -5.28
TBSO
BnO
CO2Me
AcO
BnO
HO
AllO
AllO
OMe
8 (CDCl3, 100 MHz)
NMR Solvent Peak
f1 (ppm)

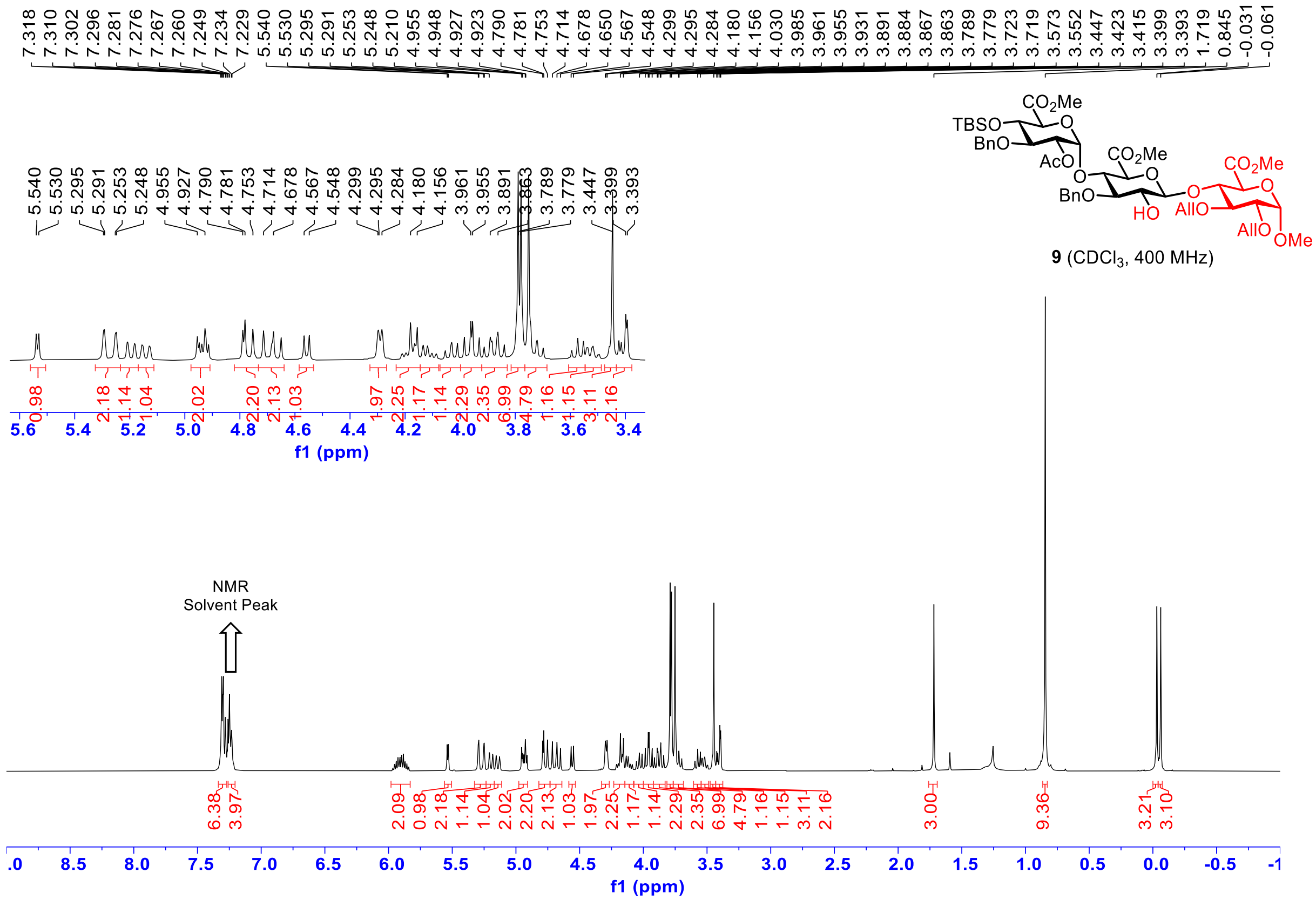
7.318 7.310 7.302 7.296 7.281 7.276 7.267 7.260 7.249 7.234 7.229 5.540 5.530 5.295 5.291 5.253 5.248 5.210 4.955 4.948 4.927 4.923 4.790 4.781 4.753 4.714 4.678 4.650 4.567 4.548 4.299 4.295 4.284 4.180 4.156 4.030 3.985 3.961 3.955 3.931 3.891 3.884 3.867 3.863 3.789 3.779 3.723 3.719 3.573 3.552 3.447 3.423 3.415 3.399 3.393 1.719 0.845 -0.031 -0.061
TBSO
BnO
CO_2Me
AcO
BnO
HO
AllO
AllO
OMe
9 ($CDCl_3$, 400 MHz)
NMR
Solvent Peak
f1 (ppm)

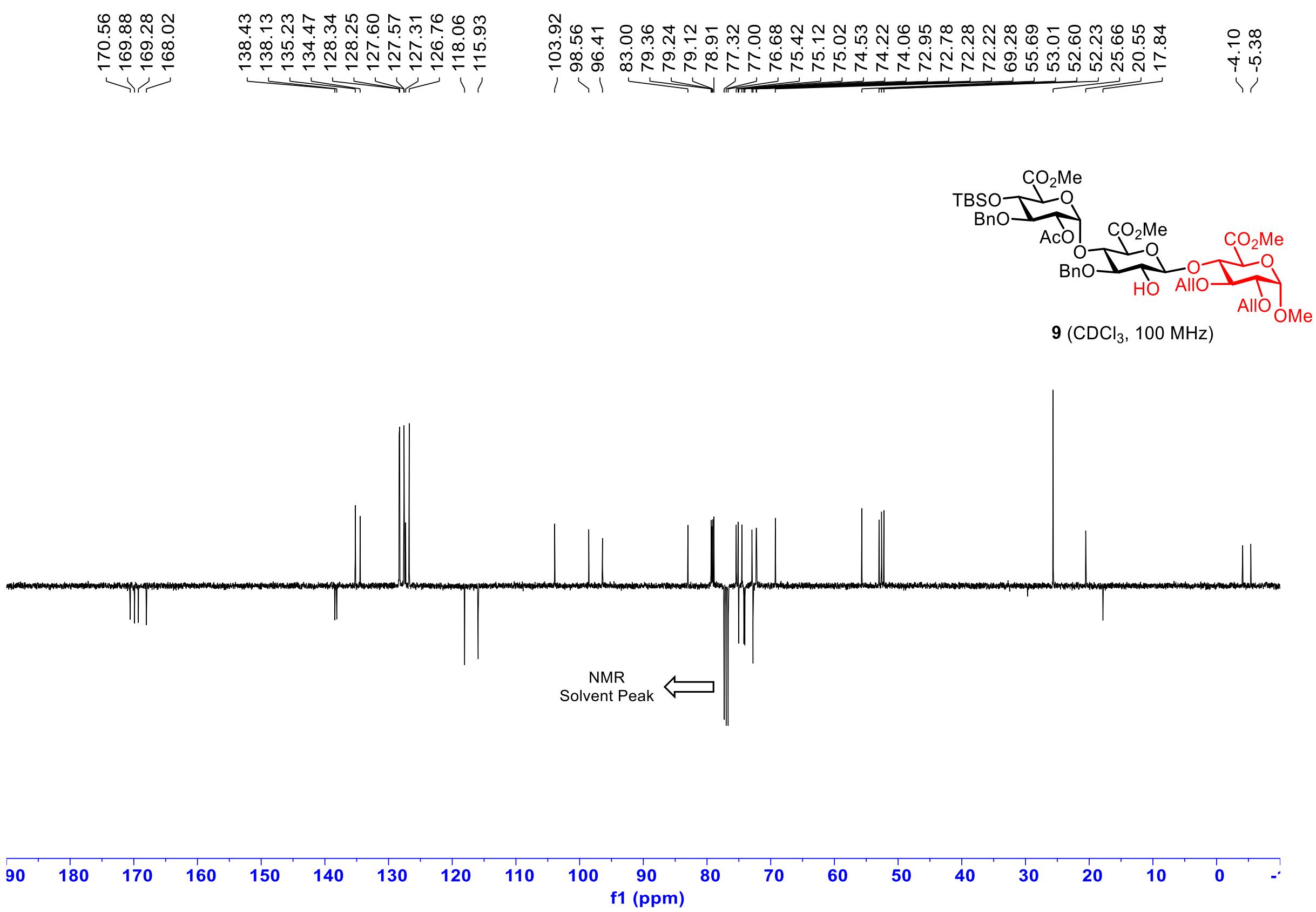
170.56 169.88 169.28 168.02 138.43 138.13 135.23 134.47 128.34 128.25 127.60 127.57 127.31 126.76 118.06 115.93 103.92 98.56 96.41 83.00 79.36 79.24 79.12 78.91 77.32 77.00 76.68 75.42 75.12 75.02 74.53 74.22 74.06 72.95 72.78 72.28 72.22 69.28 55.69 53.01 52.60 52.23 25.66 20.55 17.84 -4.10 -5.38
TBSO
BnO
CO_2Me
AcO
BnO
HO
AllO
AllO
OMe
9 ($CDCl_3$, 100 MHz)
NMR
Solvent Peak
f1 (ppm)

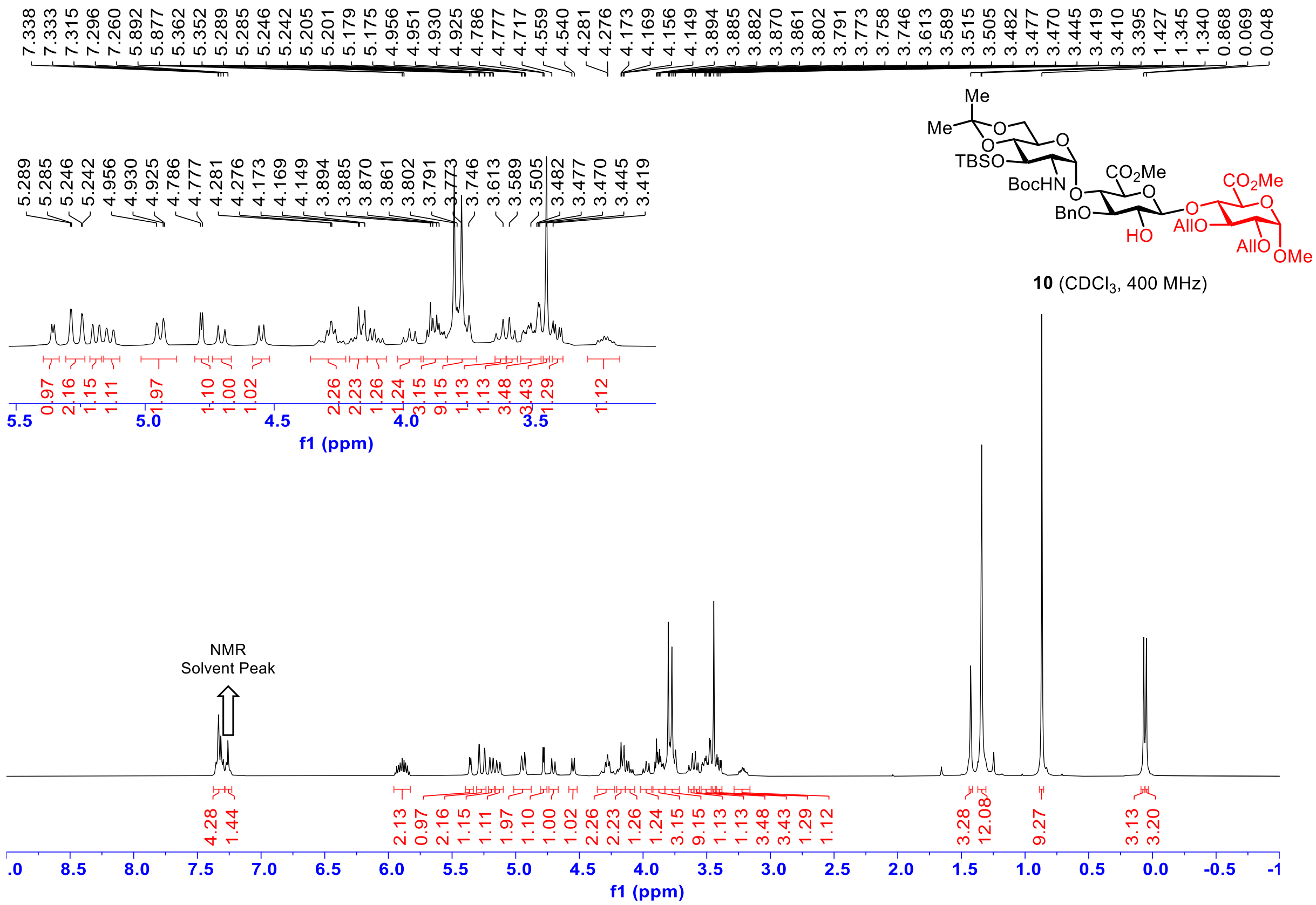

10 ($CDCl_3$, 400 MHz)

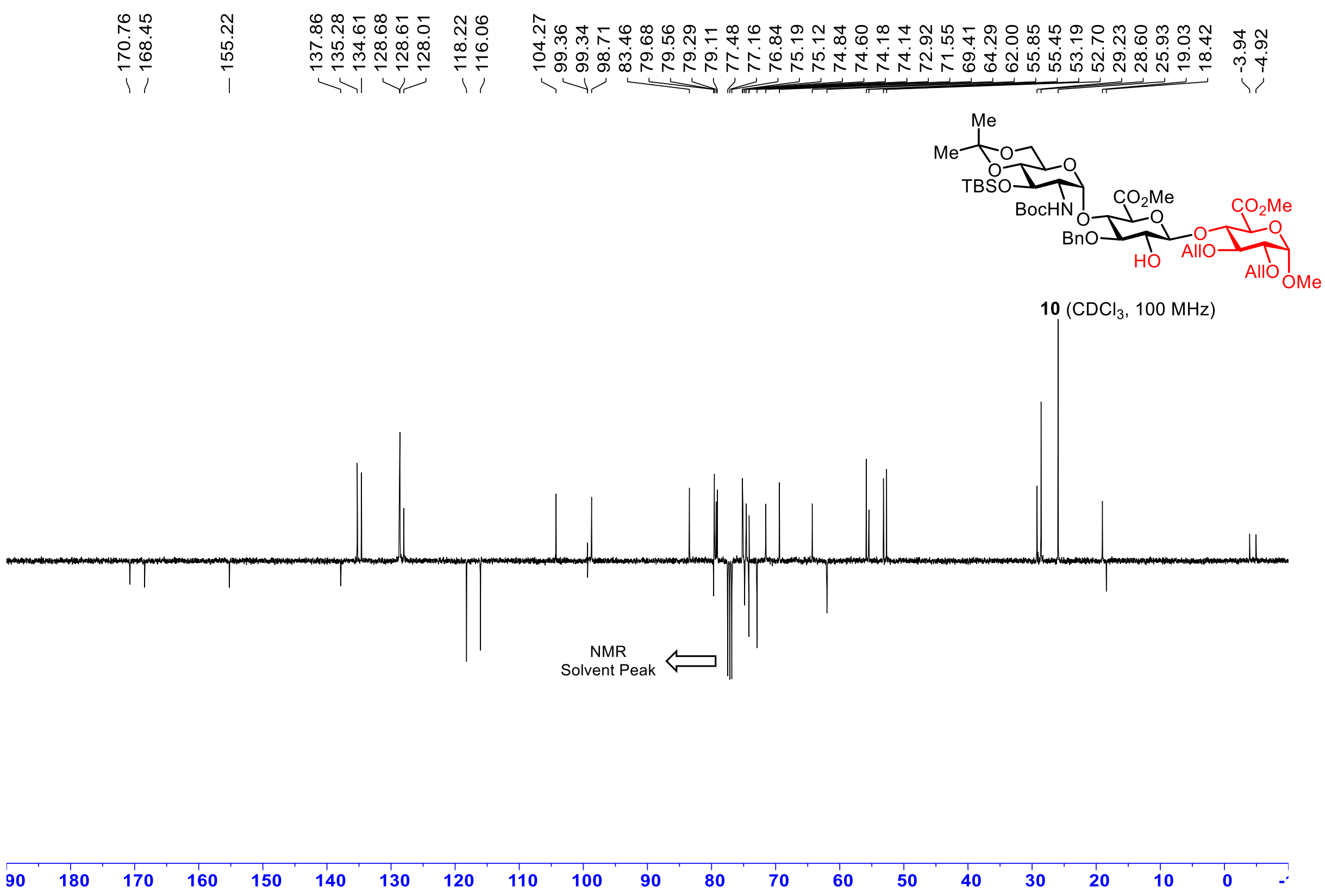

10 ($CDCl_3$, 100 MHz)

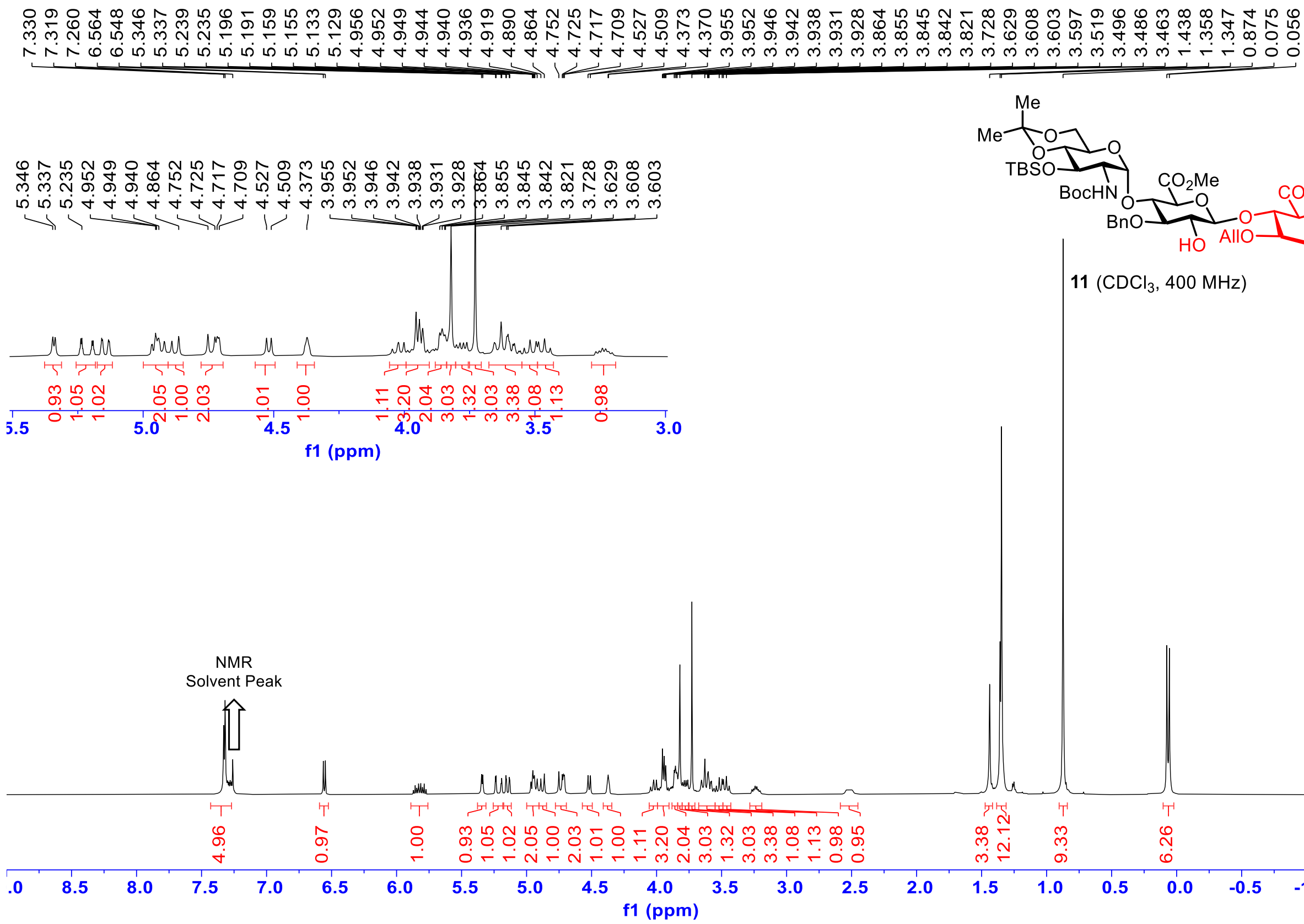
11 (CDCl$_3$, 400 MHz)
NMR Solvent Peak
f1 (ppm)

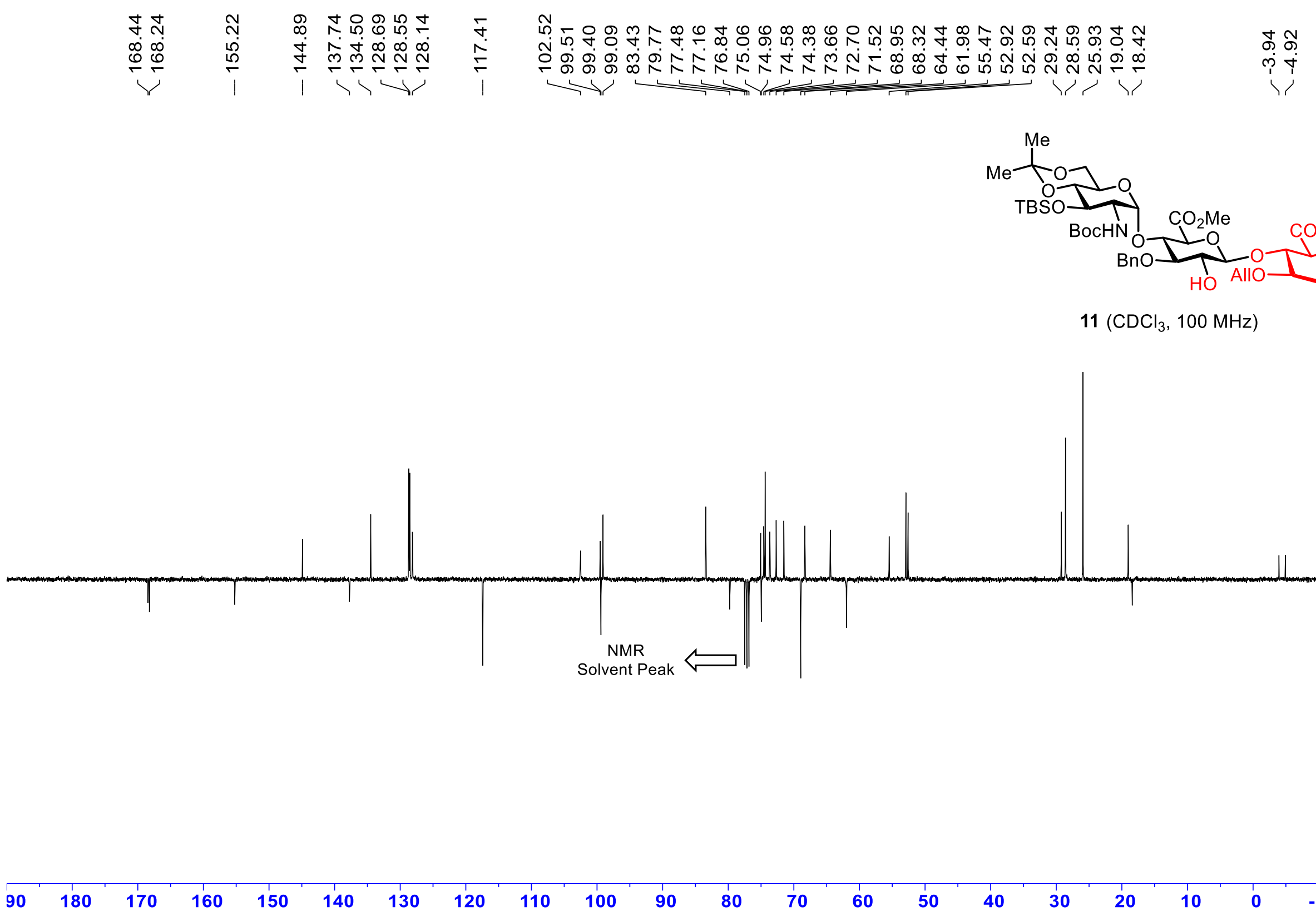
11 (CDCl$_3$, 100 MHz)
NMR Solvent Peak
f1 (ppm)

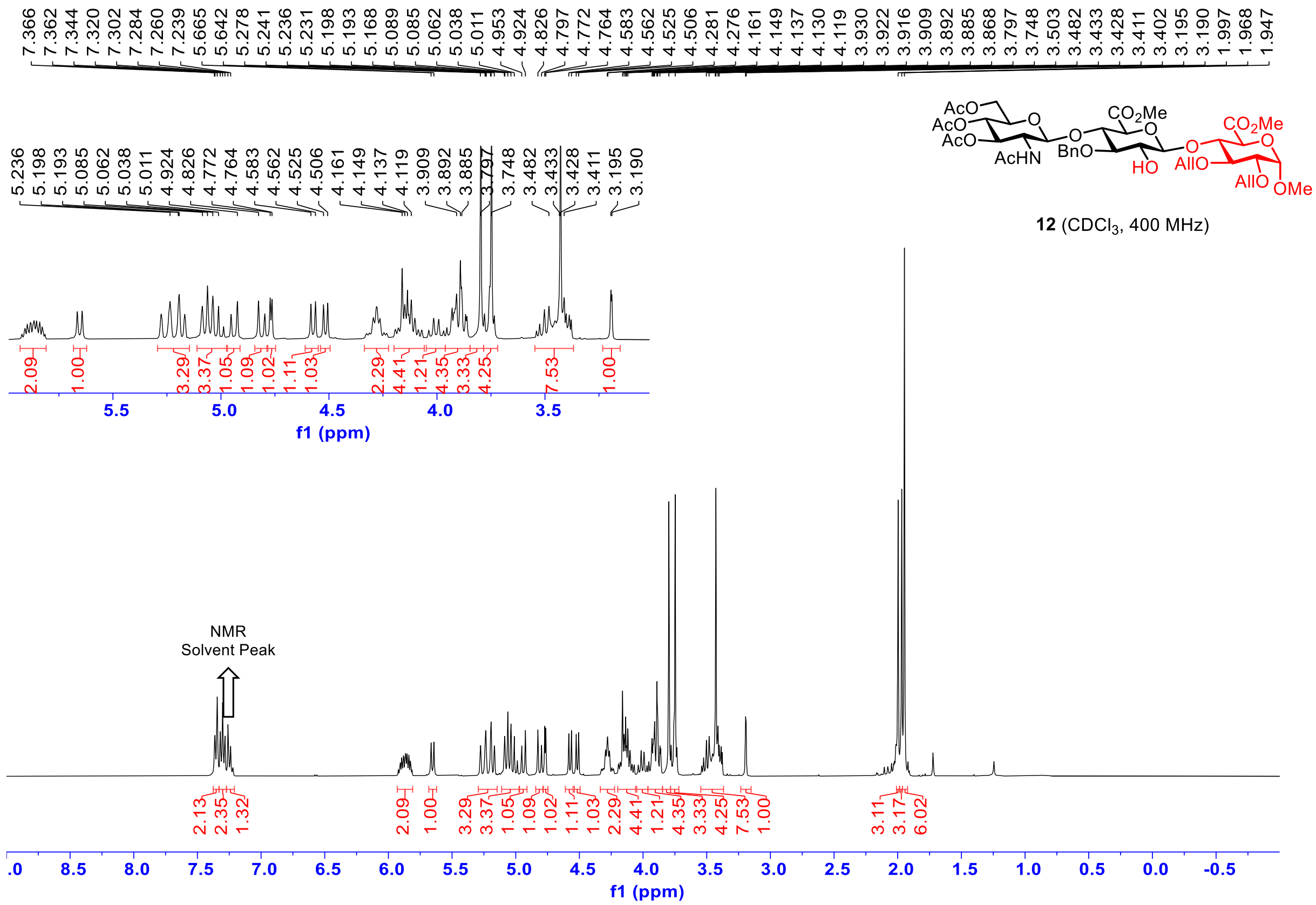

12 ($CDCl_3$, 400 MHz)

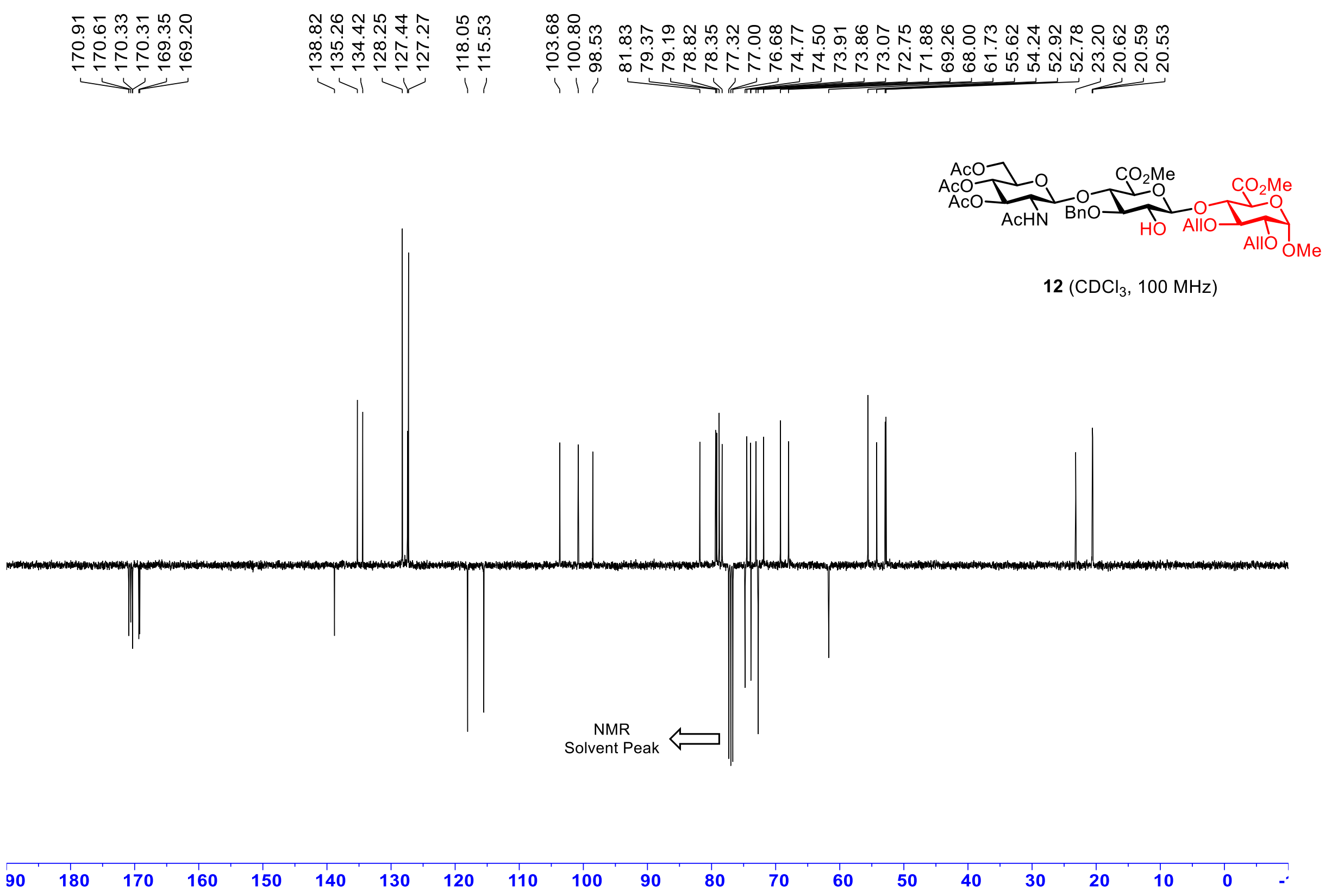

12 ($CDCl_3$, 100 MHz)

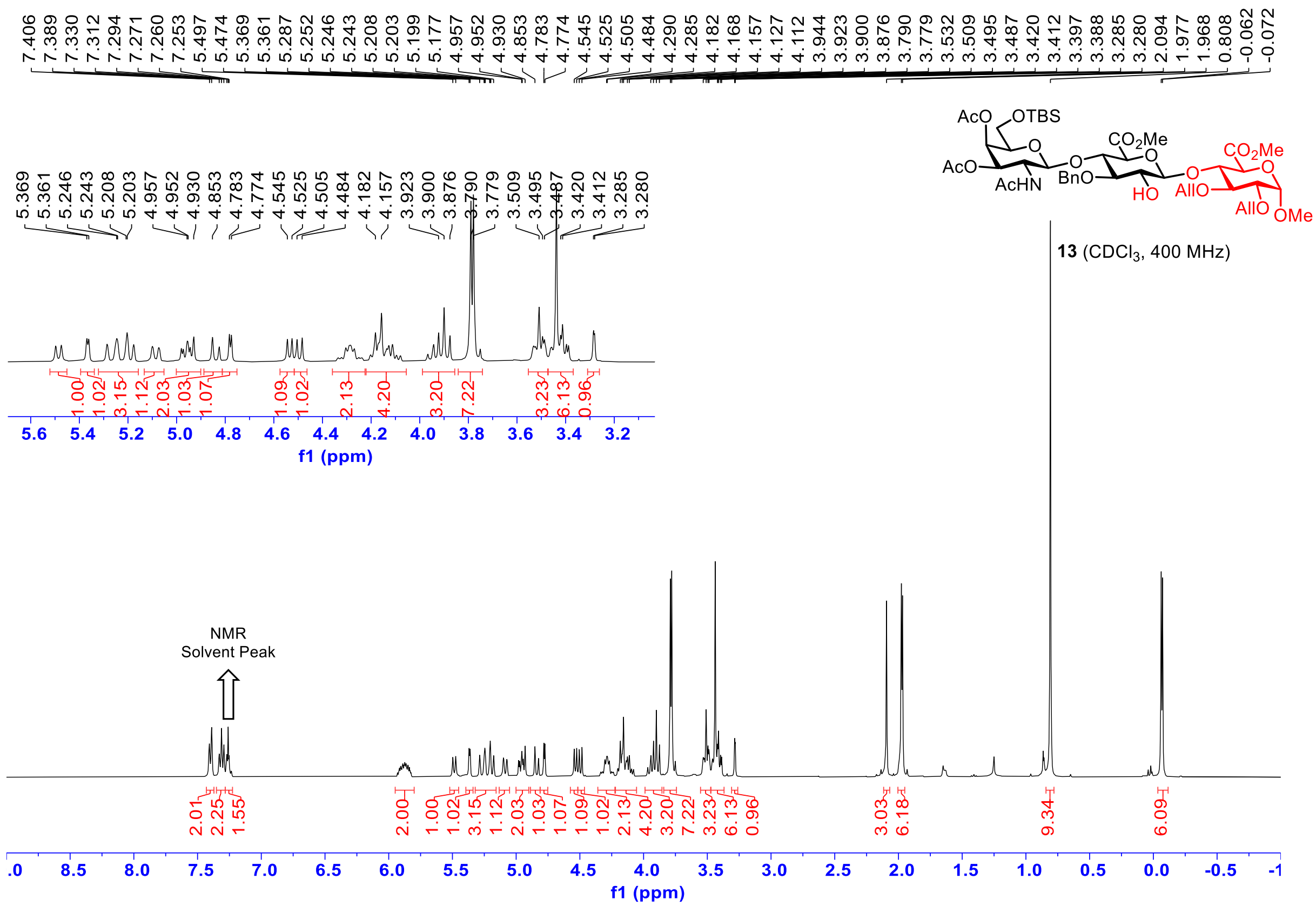

13 ($CDCl_3$, 400 MHz)

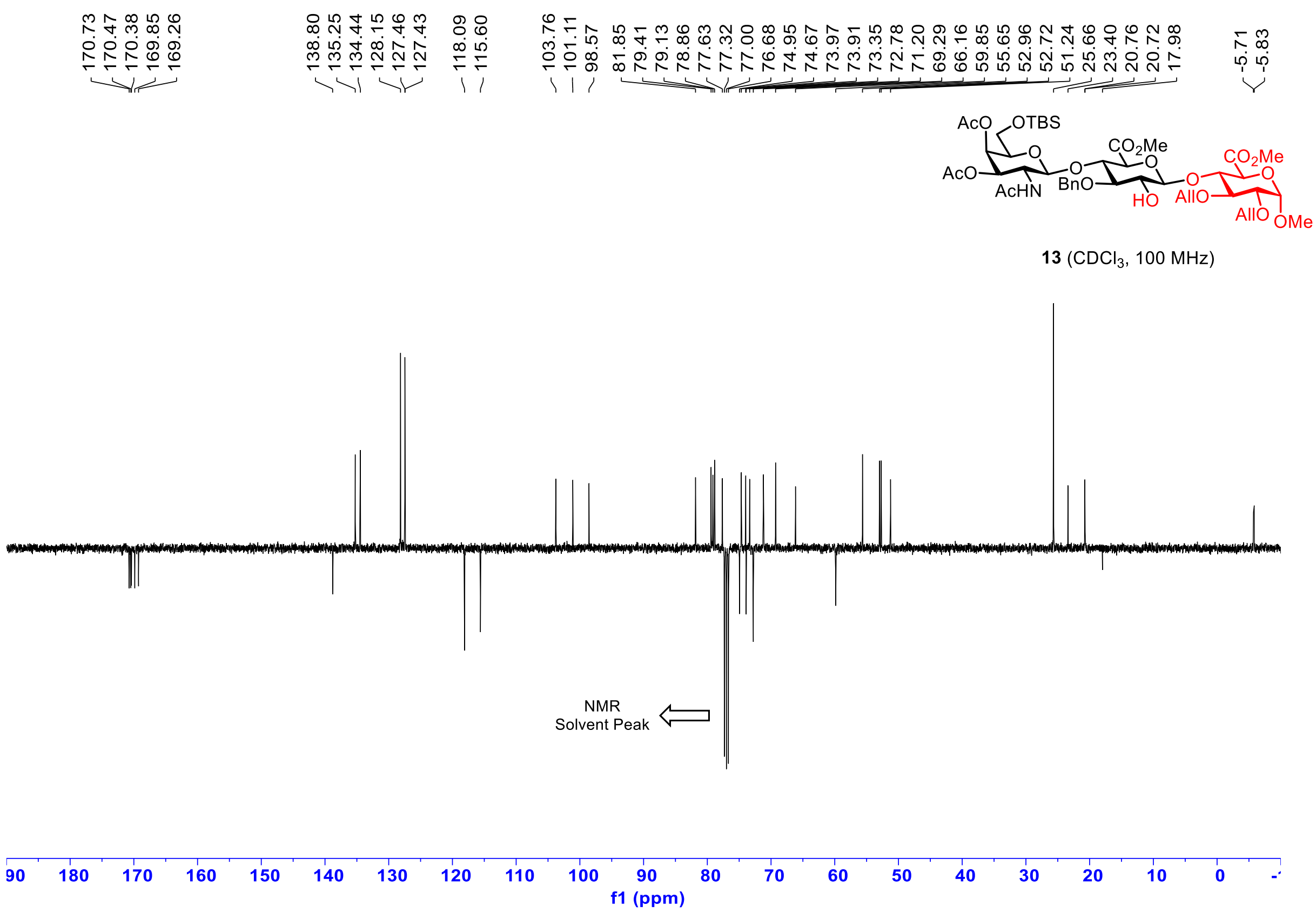

13 ($CDCl_3$, 100 MHz)

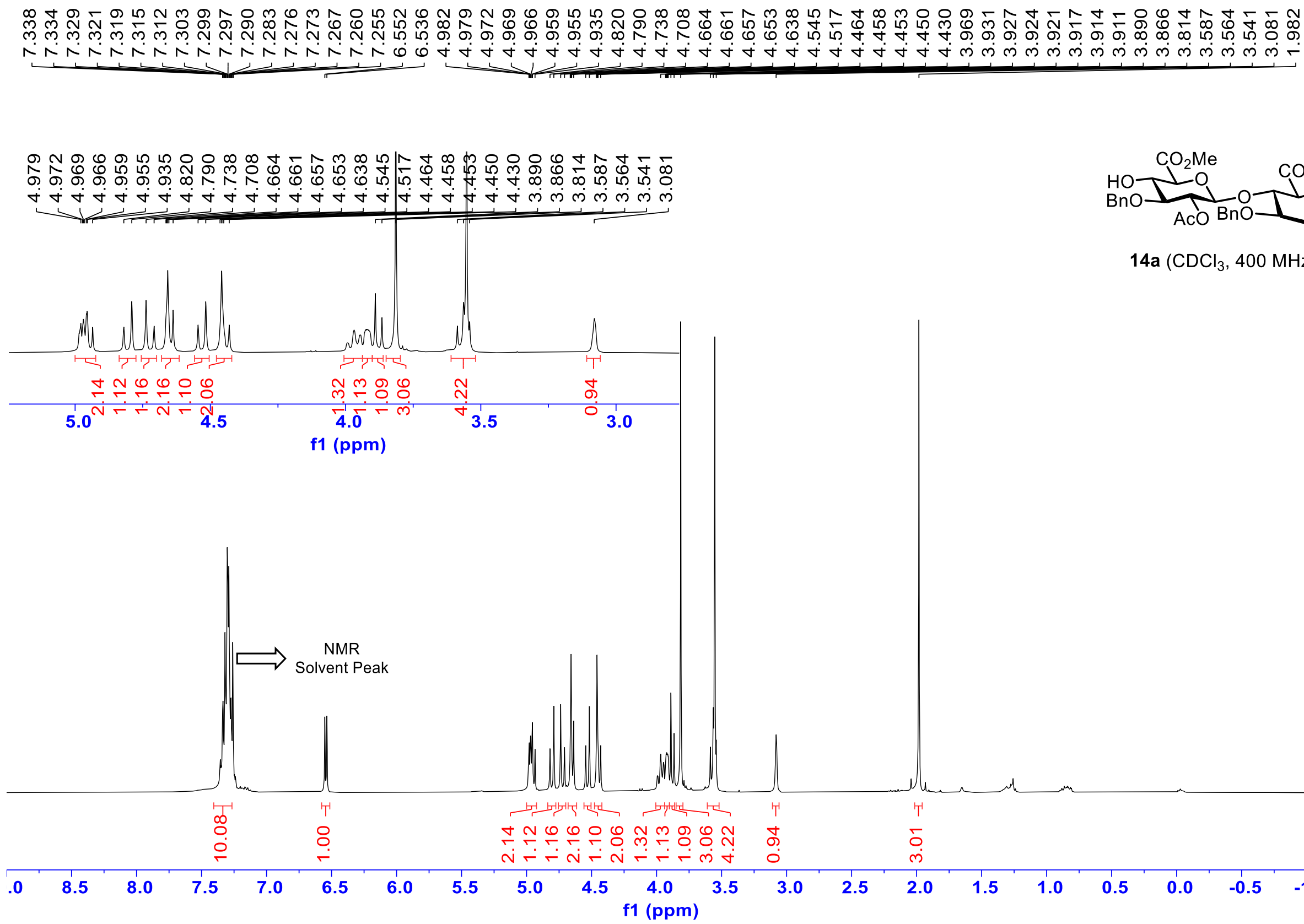
14a (CDCl3, 400 MHz)
NMR
Solvent Peak
f1 (ppm)

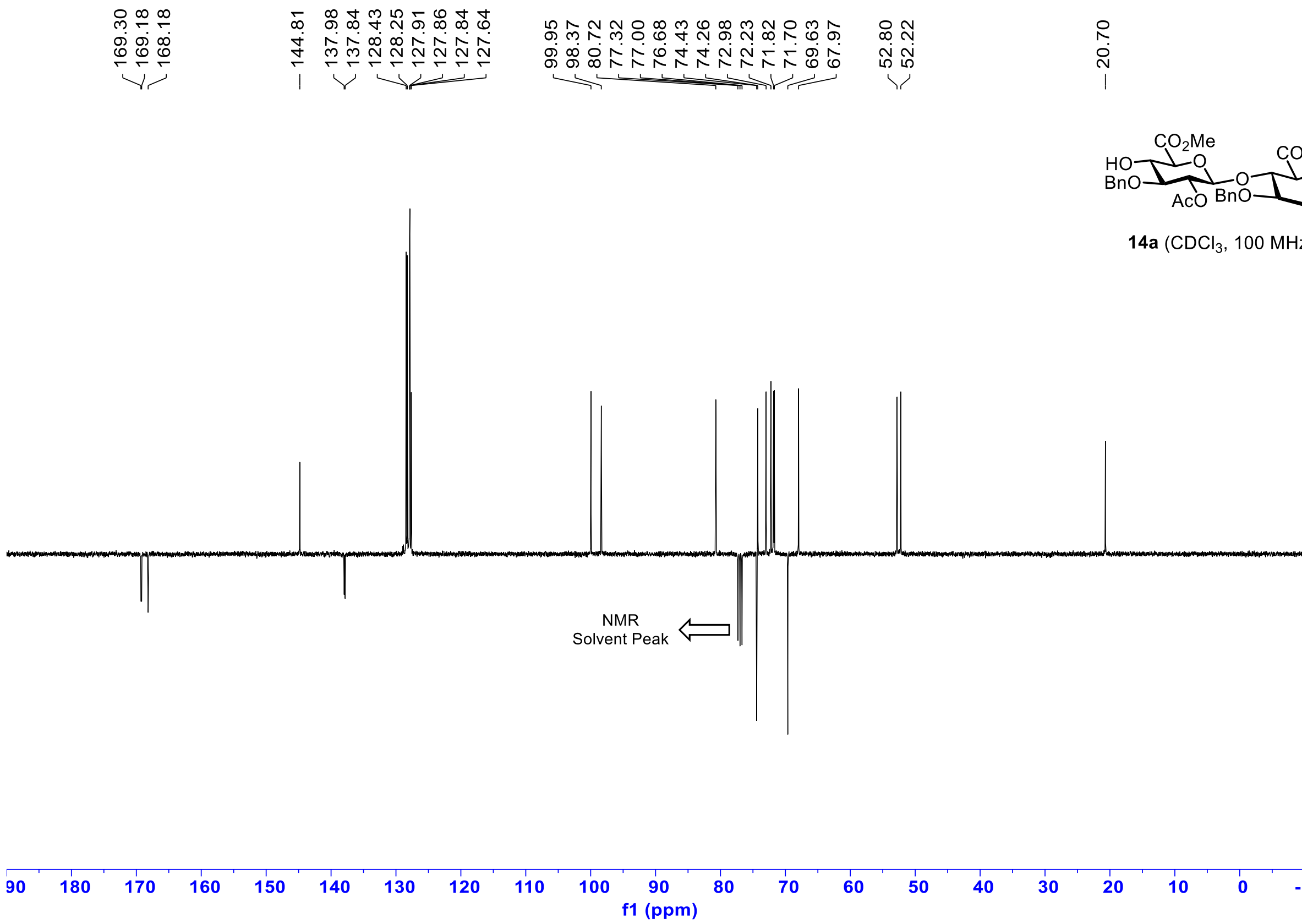
14a (CDCl3, 100 MHz)
NMR
Solvent Peak
f1 (ppm)

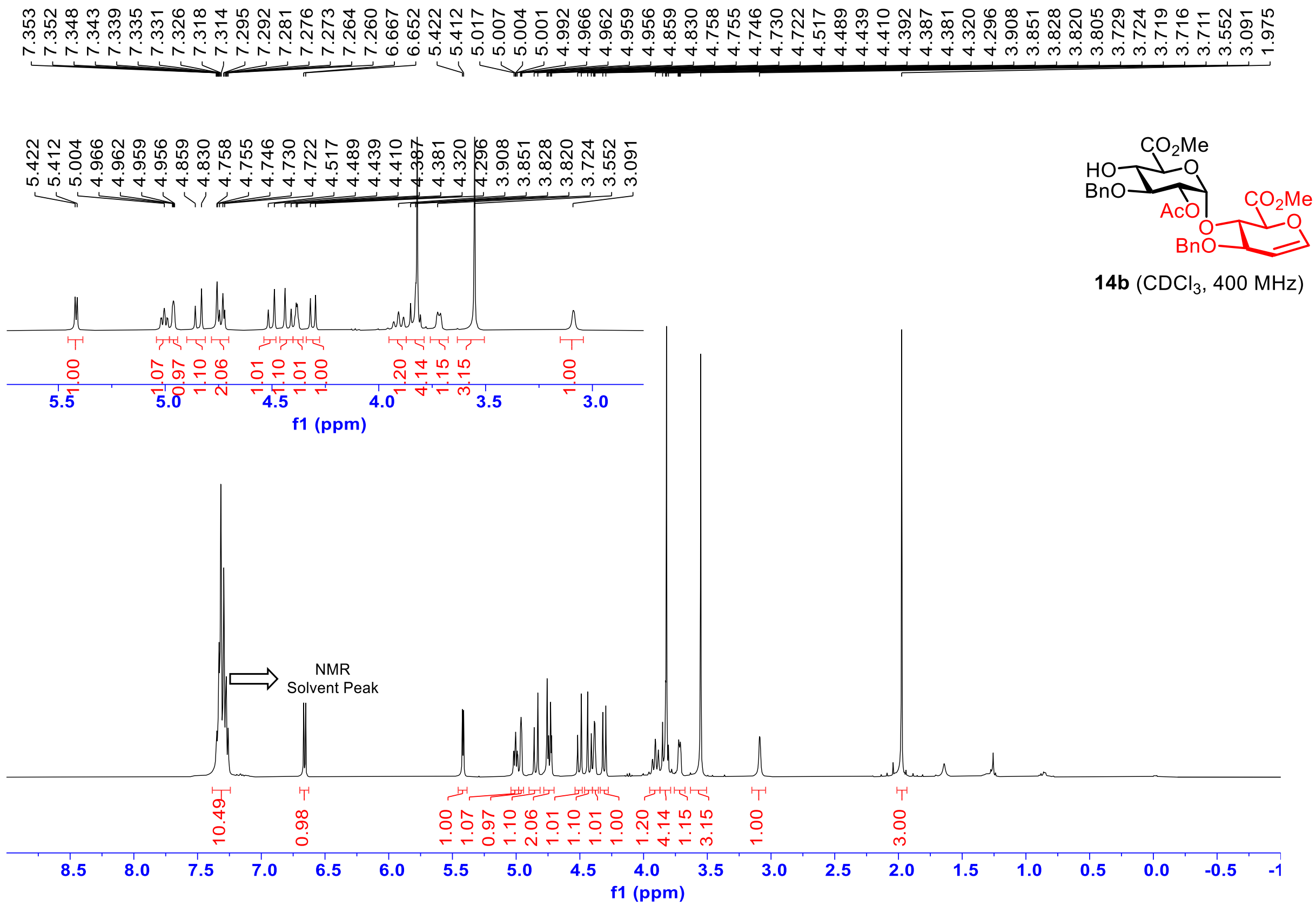
7.353 7.352 7.348 7.343 7.339 7.335 7.331 7.326 7.318 7.314 7.295 7.292 7.281 7.276 7.273 7.264 7.260 6.667 6.652 5.422 5.412 5.017 5.007 5.004 5.001 4.992 4.966 4.962 4.959 4.956 4.859 4.830 4.758 4.755 4.746 4.730 4.722 4.517 4.489 4.439 4.410 4.392 4.387 4.381 4.320 4.296 3.908 3.851 3.828 3.820 3.805 3.729 3.724 3.719 3.716 3.711 3.552 3.091 1.975
5.422 5.412 5.004 4.966 4.962 4.956 4.859 4.830 4.758 4.755 4.746 4.730 4.722 4.517 4.489 4.439 4.410 4.387 4.381 4.320 4.296 3.908 3.851 3.828 3.820 3.724 3.552 3.091
1.00 1.07 0.97 1.10 2.06 1.01 1.10 1.01 1.00 1.20 4.14 1.15 3.15 1.00
f1 (ppm)
NMR Solvent Peak
10.49 0.98 1.00 1.07 0.97 1.10 2.06 1.01 1.10 1.01 1.00 1.20 4.14 1.15 3.15 1.00 3.00
f1 (ppm)
HO BnO CO2Me AcO O BnO CO2Me
14b (CDCl3, 400 MHz)

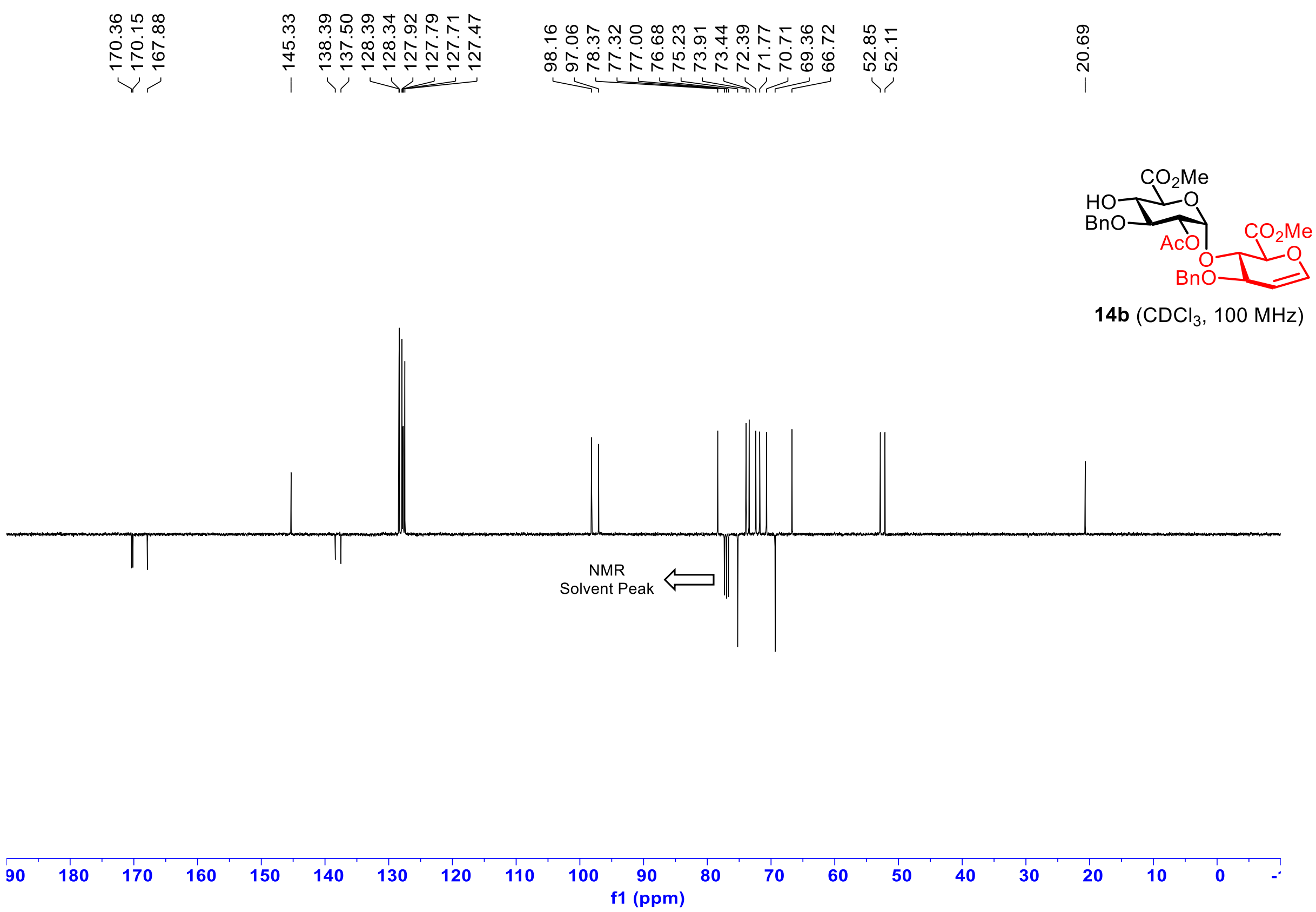
170.36 170.15 167.88 145.33 138.39 137.50 128.39 128.34 127.92 127.79 127.71 127.47 98.16 97.06 78.37 77.32 77.00 76.68 75.23 73.91 73.44 72.39 71.77 70.71 69.36 66.72 52.85 52.11 20.69
HO BnO CO2Me AcO O BnO CO2Me
14b (CDCl3, 100 MHz)
NMR Solvent Peak
f1 (ppm)

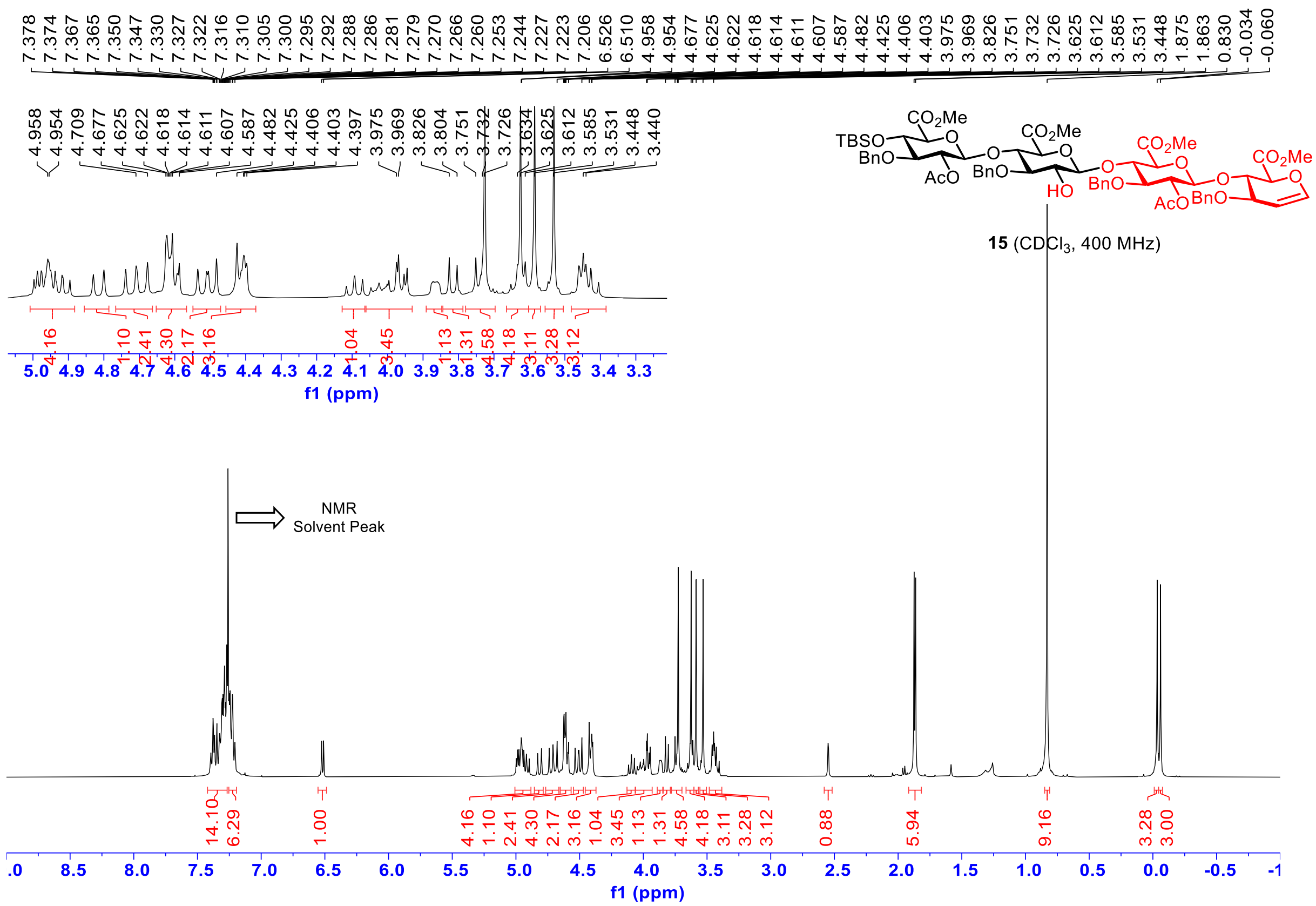

15 ($CDCl_3$, 400 MHz)
NMR
Solvent Peak
f1 (ppm)

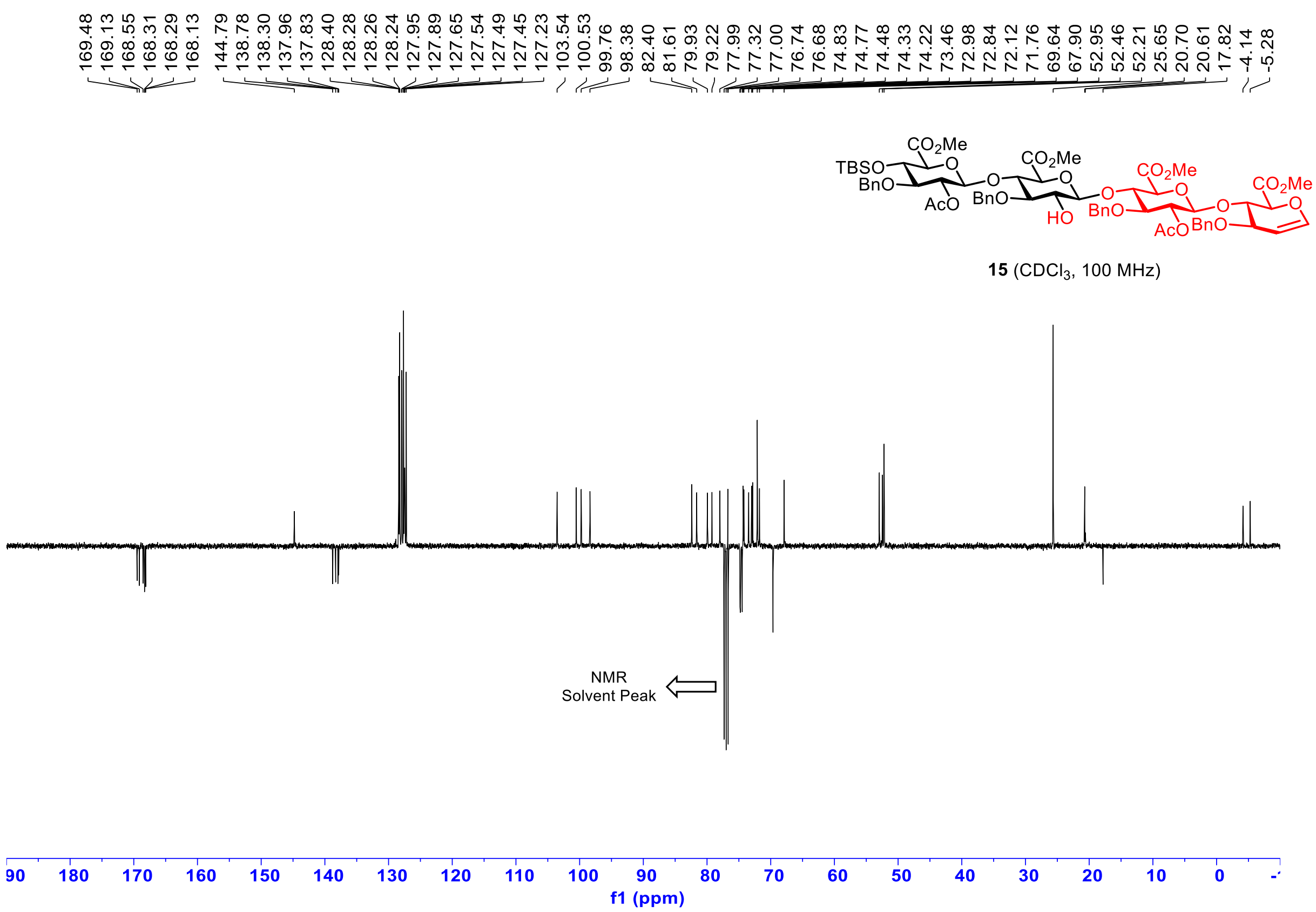

15 ($CDCl_3$, 100 MHz)
NMR
Solvent Peak
f1 (ppm)

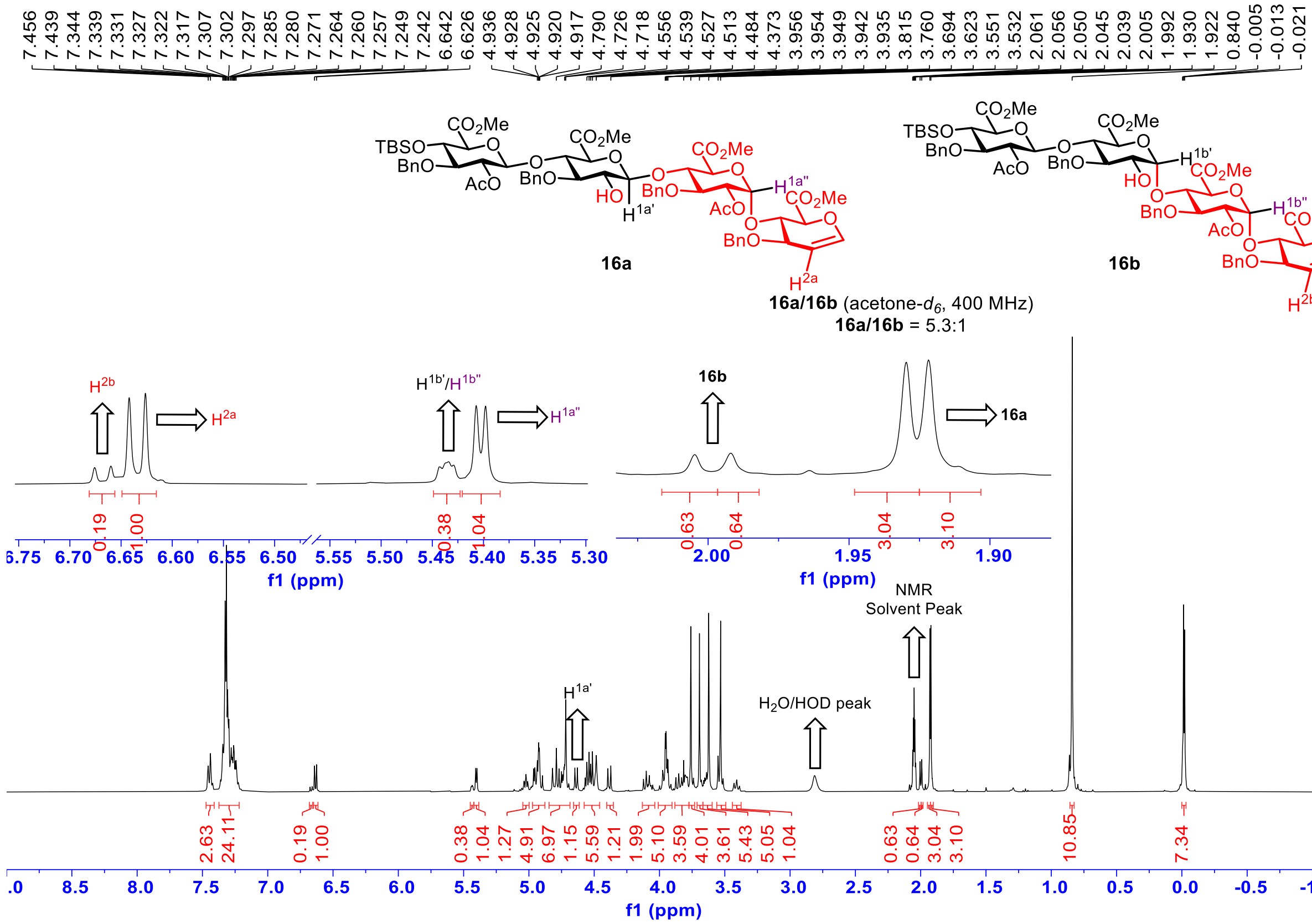
7.456 7.439 7.344 7.339 7.331 7.327 7.322 7.317 7.307 7.302 7.297 7.285 7.280 7.271 7.264 7.260 7.257 7.249 7.242 6.642 6.626 4.936 4.928 4.925 4.920 4.917 4.790 4.726 4.718 4.556 4.539 4.527 4.513 4.484 4.373 3.956 3.954 3.949 3.942 3.935 3.815 3.760 3.694 3.623 3.551 3.532 2.061 2.056 2.050 2.045 2.039 2.005 1.992 1.930 1.922 0.840 -0.005 -0.013 -0.021
16a
16b
16a/16b (acetone-d_6, 400 MHz)
16a/16b = 5.3:1
NMR Solvent Peak
H₂O/HOD peak
f1 (ppm)
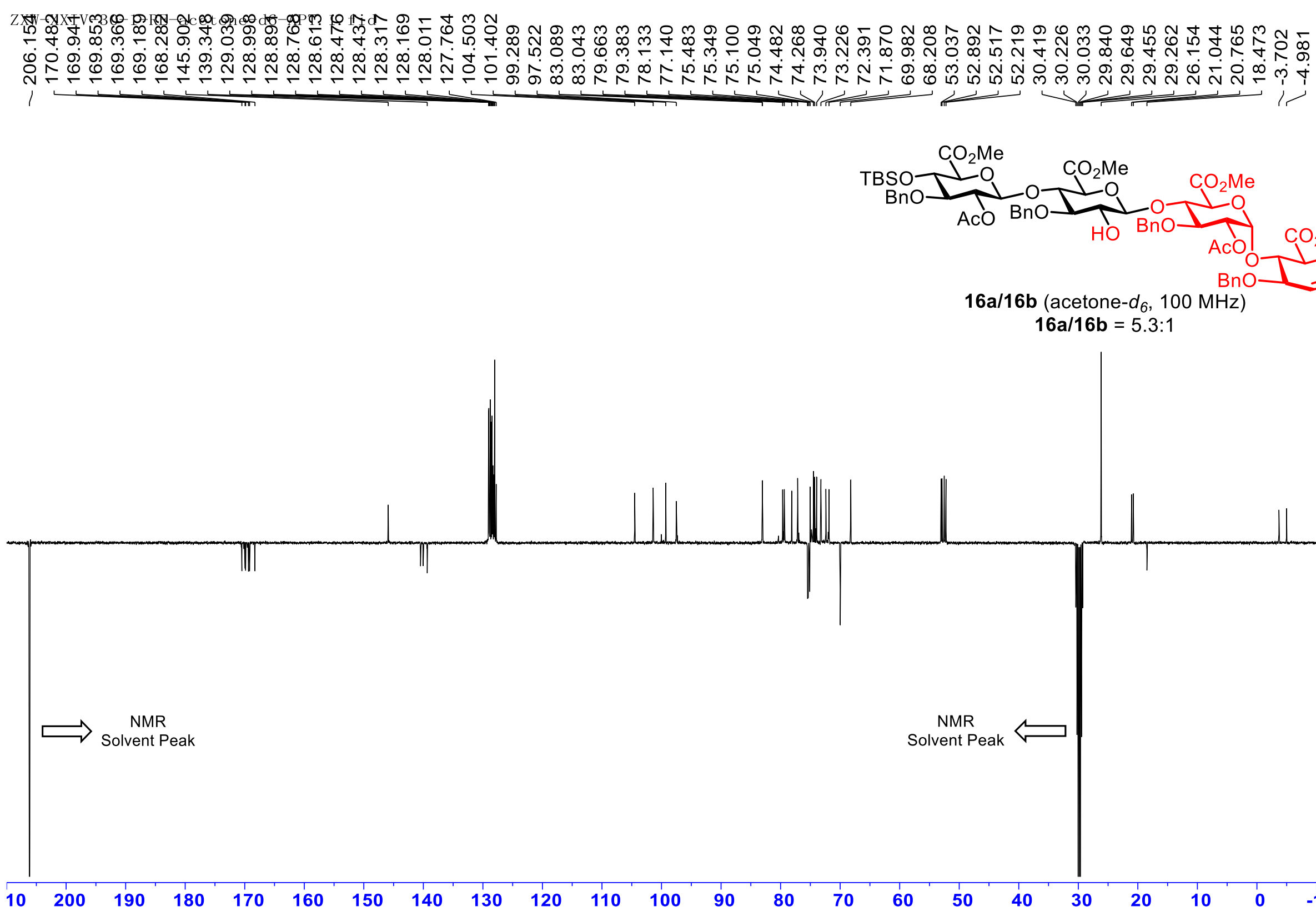
206.154 170.482 169.941 169.853 169.366 169.189 168.282 145.902 139.348 129.039 128.998 128.893 128.763 128.613 128.476 128.437 128.317 128.169 128.011 127.764 104.503 101.402 99.289 97.522 83.089 83.043 79.663 79.383 78.133 77.140 75.483 75.349 75.100 75.049 74.482 74.268 73.940 73.226 72.391 71.870 69.982 68.208 53.037 52.892 52.517 52.219 30.419 30.226 30.033 29.840 29.649 29.455 29.262 26.154 21.044 20.765 18.473 -3.702 -4.981
16a/16b (acetone-d_6, 100 MHz)
16a/16b = 5.3:1
NMR Solvent Peak
NMR Solvent Peak
f1 (ppm)

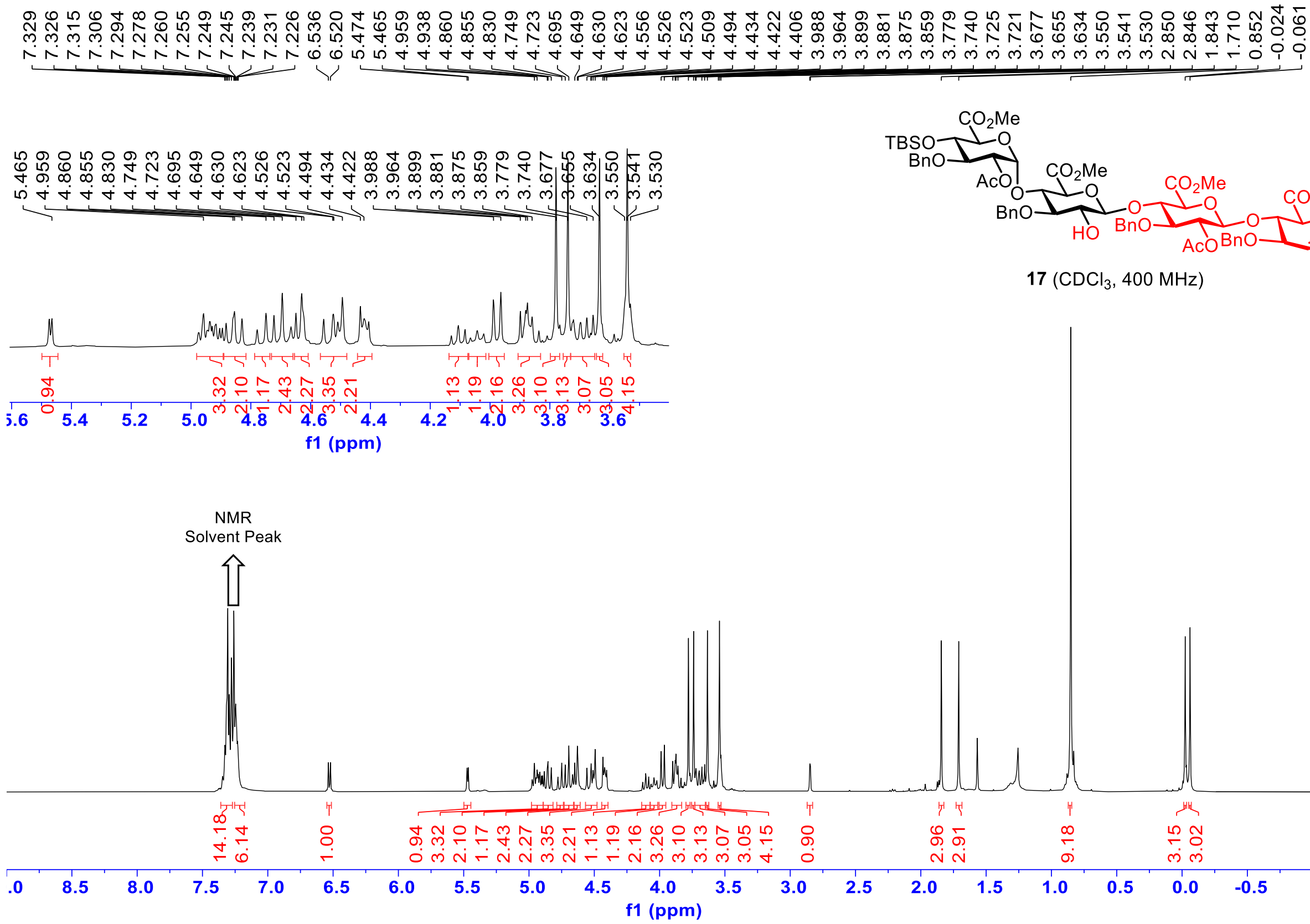

17 ($CDCl_3$, 400 MHz)

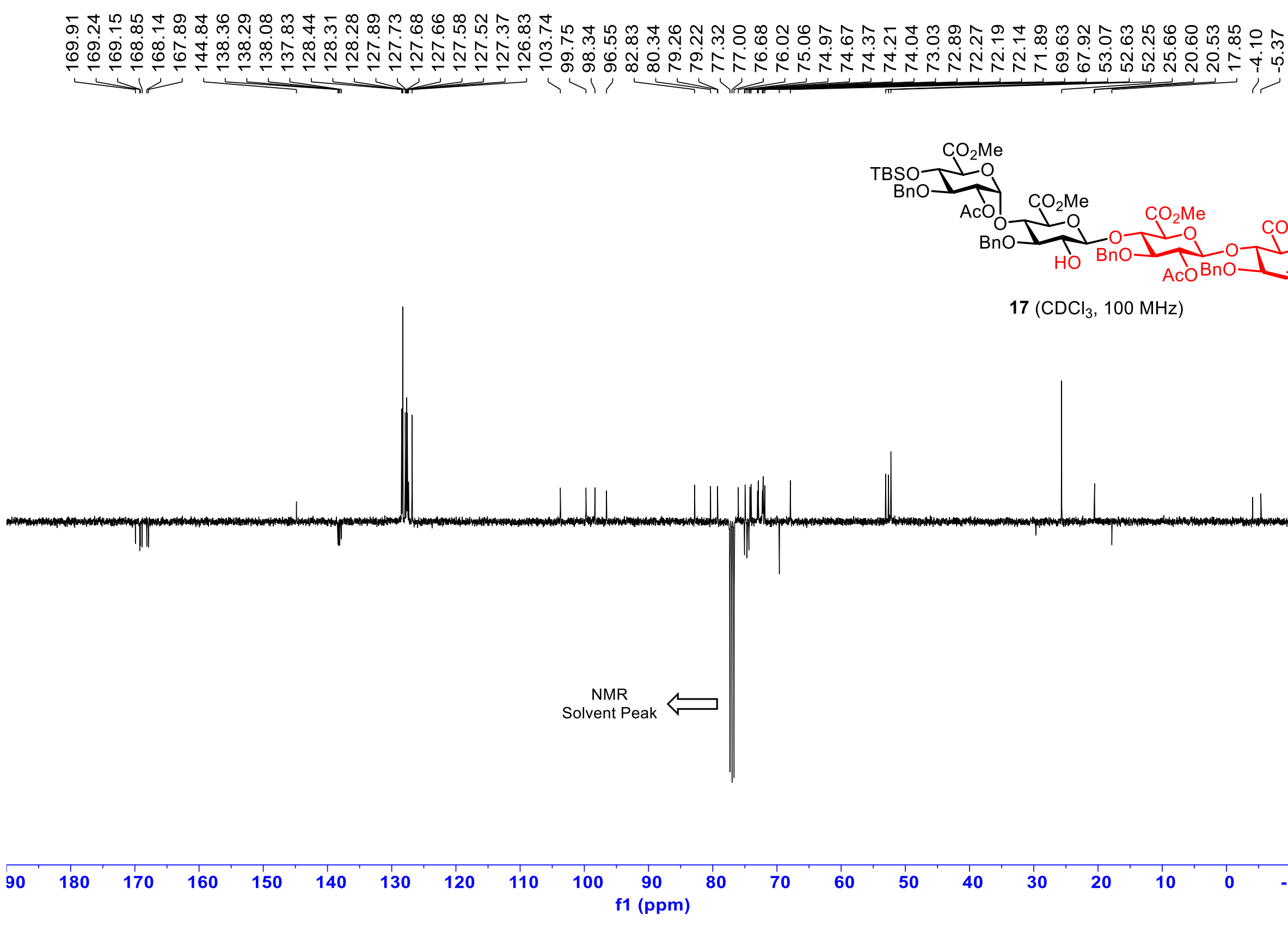

17 ($CDCl_3$, 100 MHz)

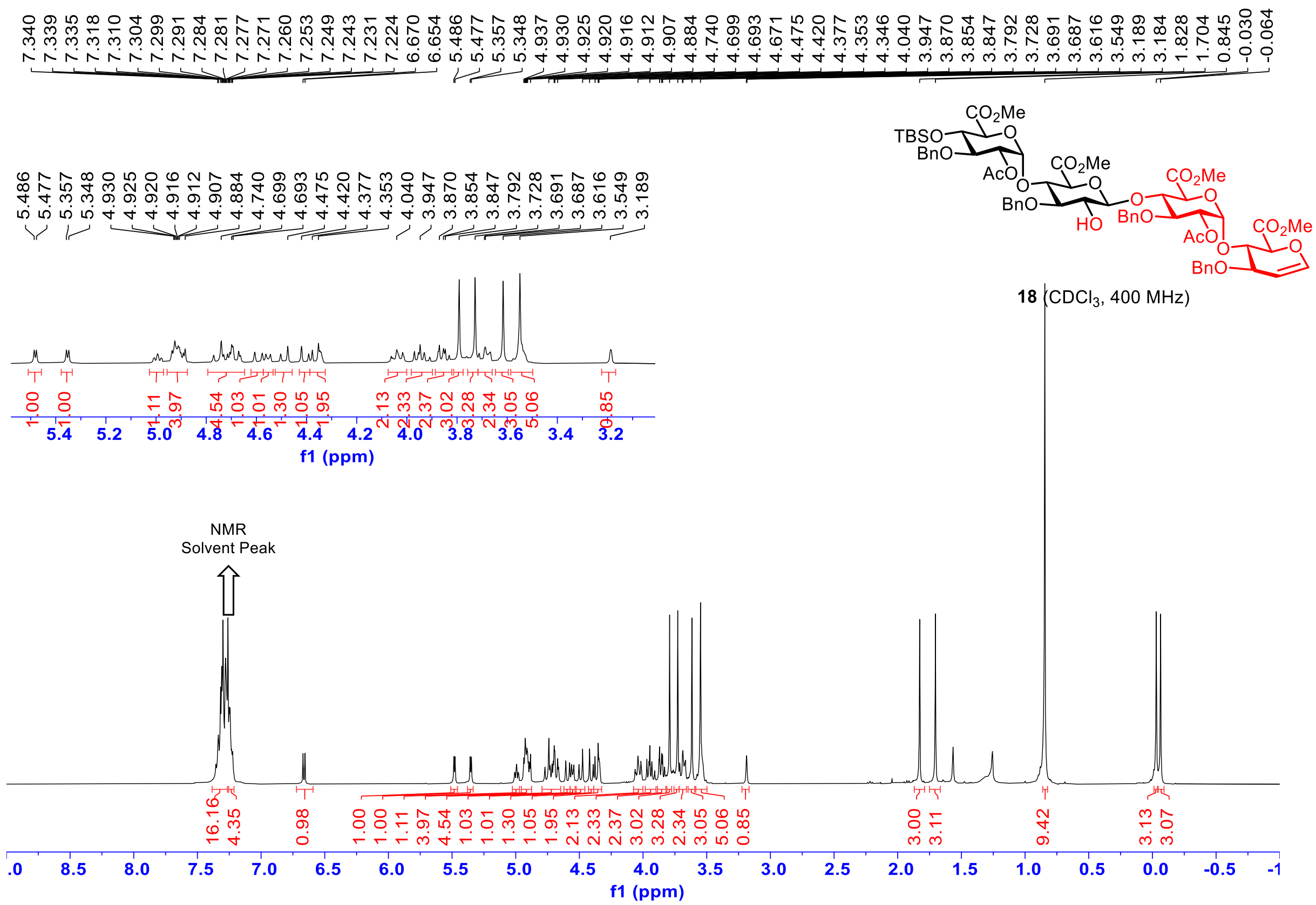

18 ($CDCl_3$, 400 MHz)

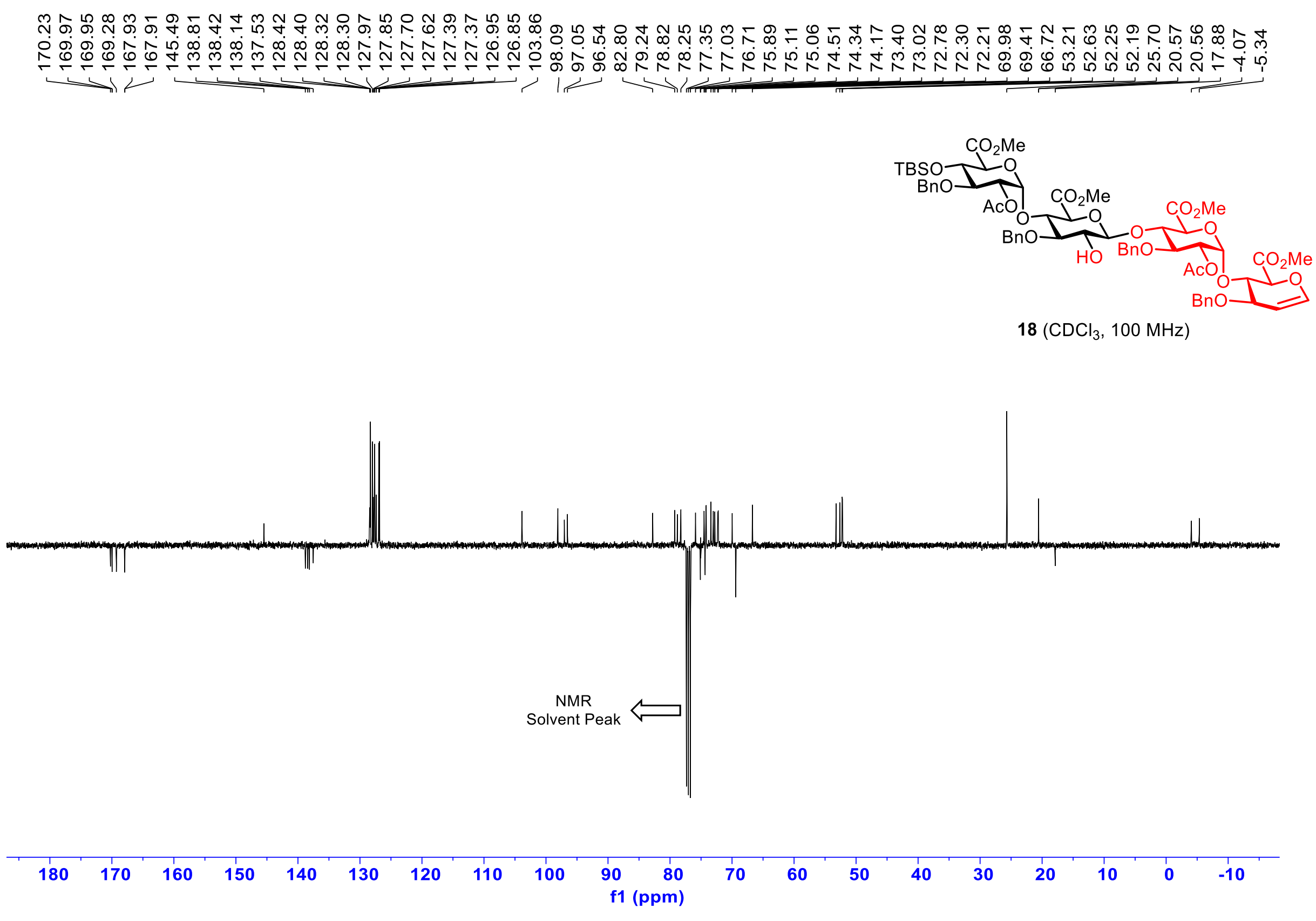

18 ($CDCl_3$, 100 MHz)

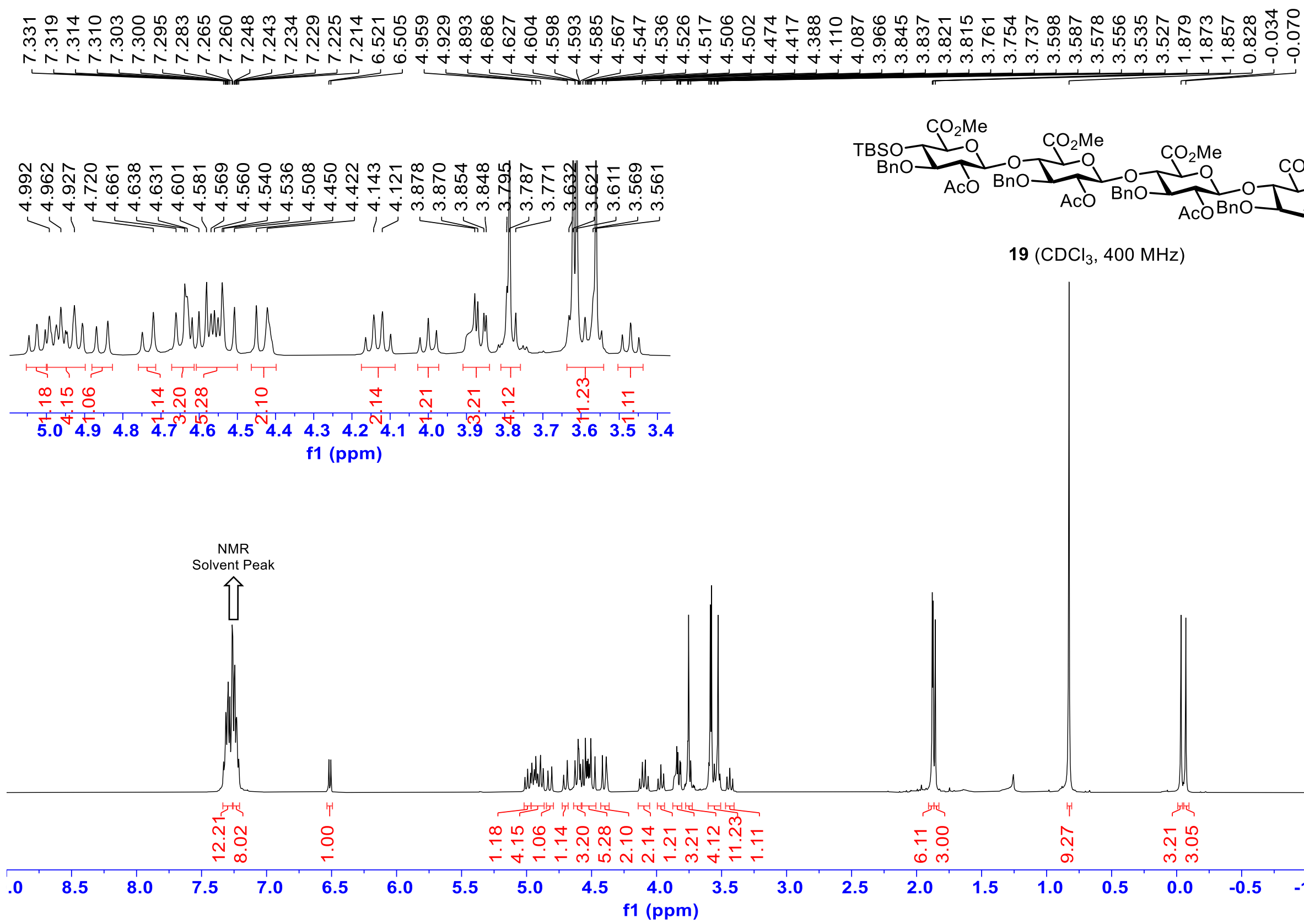
19 (CDCl3, 400 MHz)
NMR Solvent Peak
f1 (ppm)

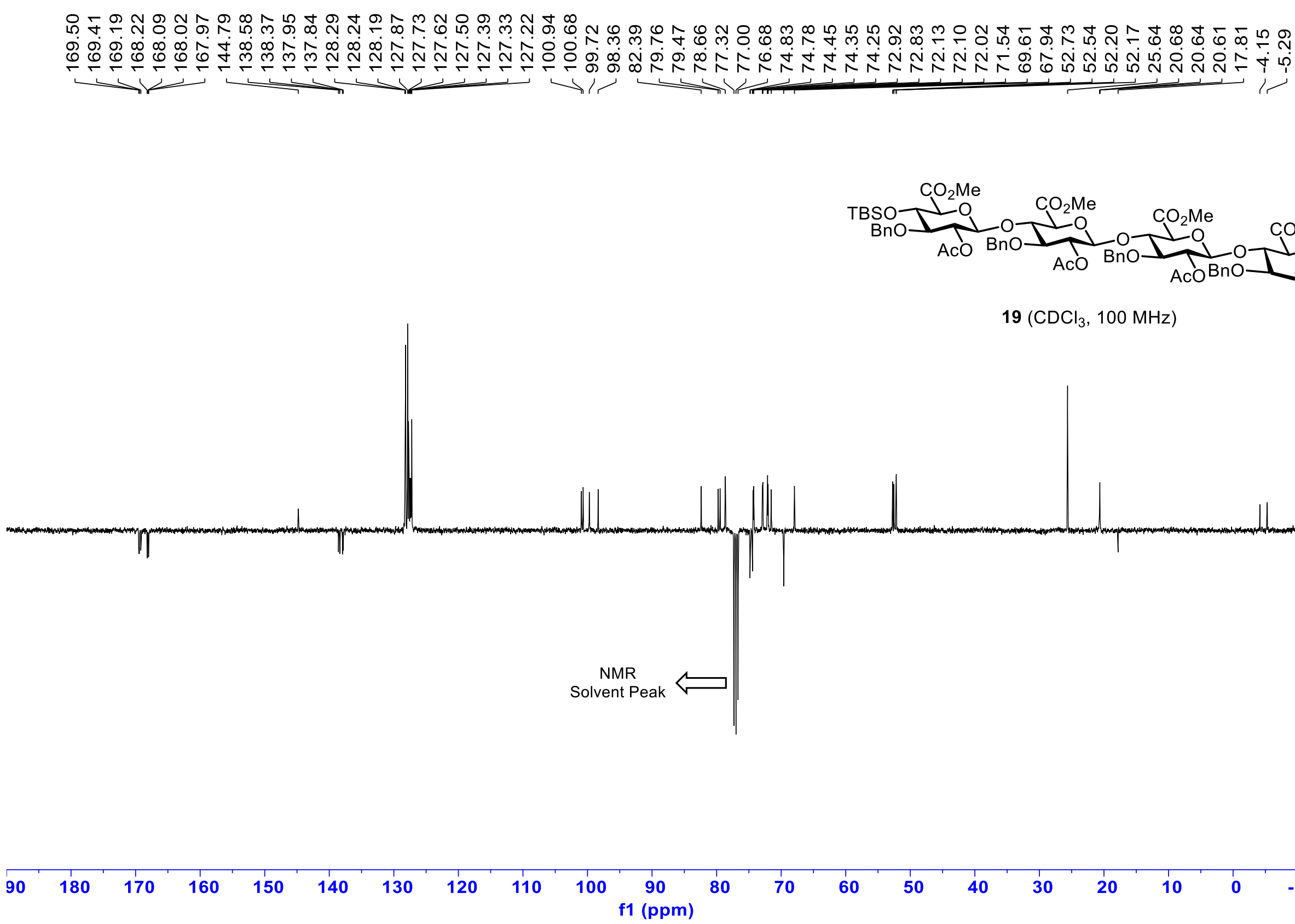
19 (CDCl3, 100 MHz)
NMR Solvent Peak
f1 (ppm)

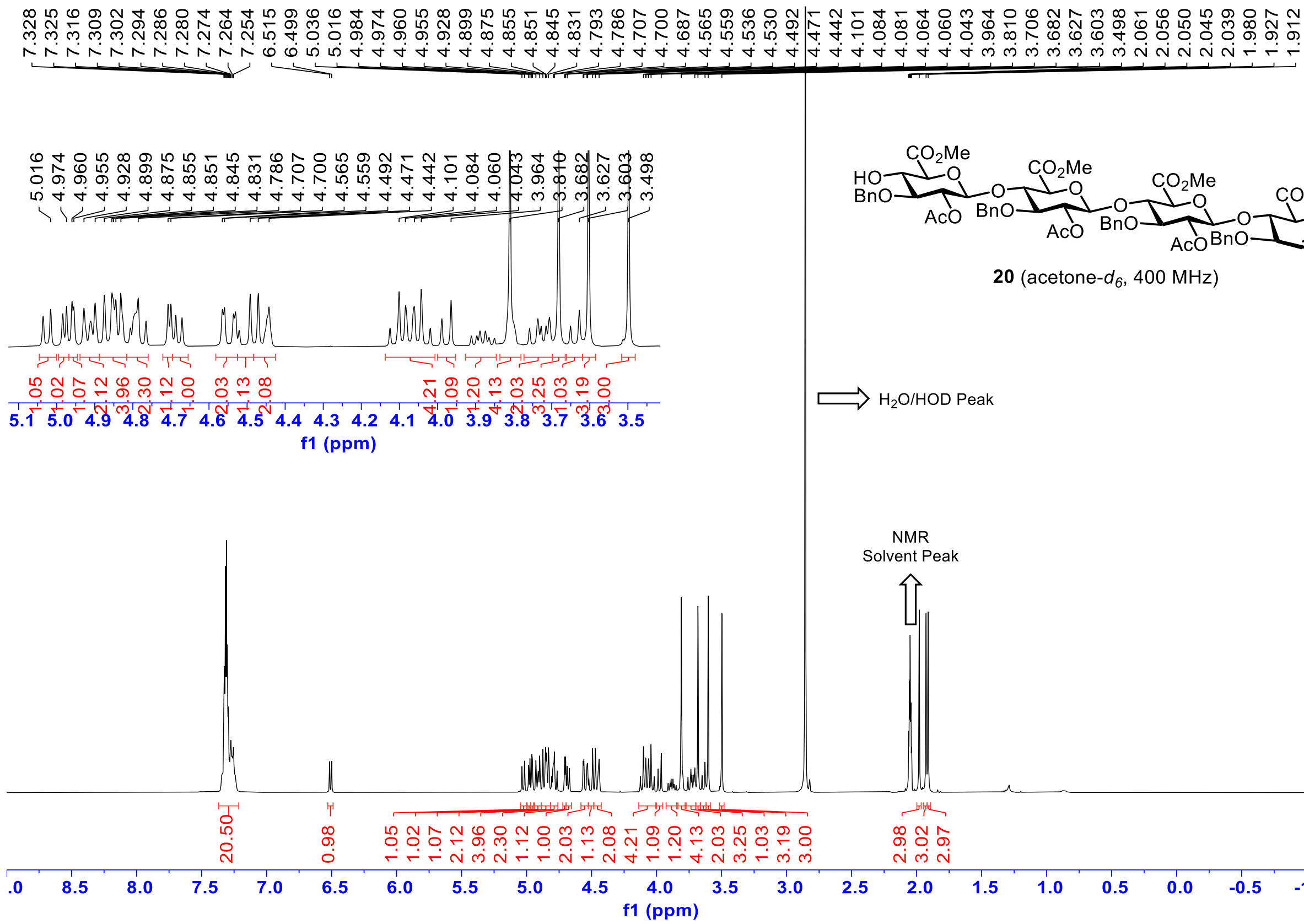

20 (acetone-d_6, 400 MHz)

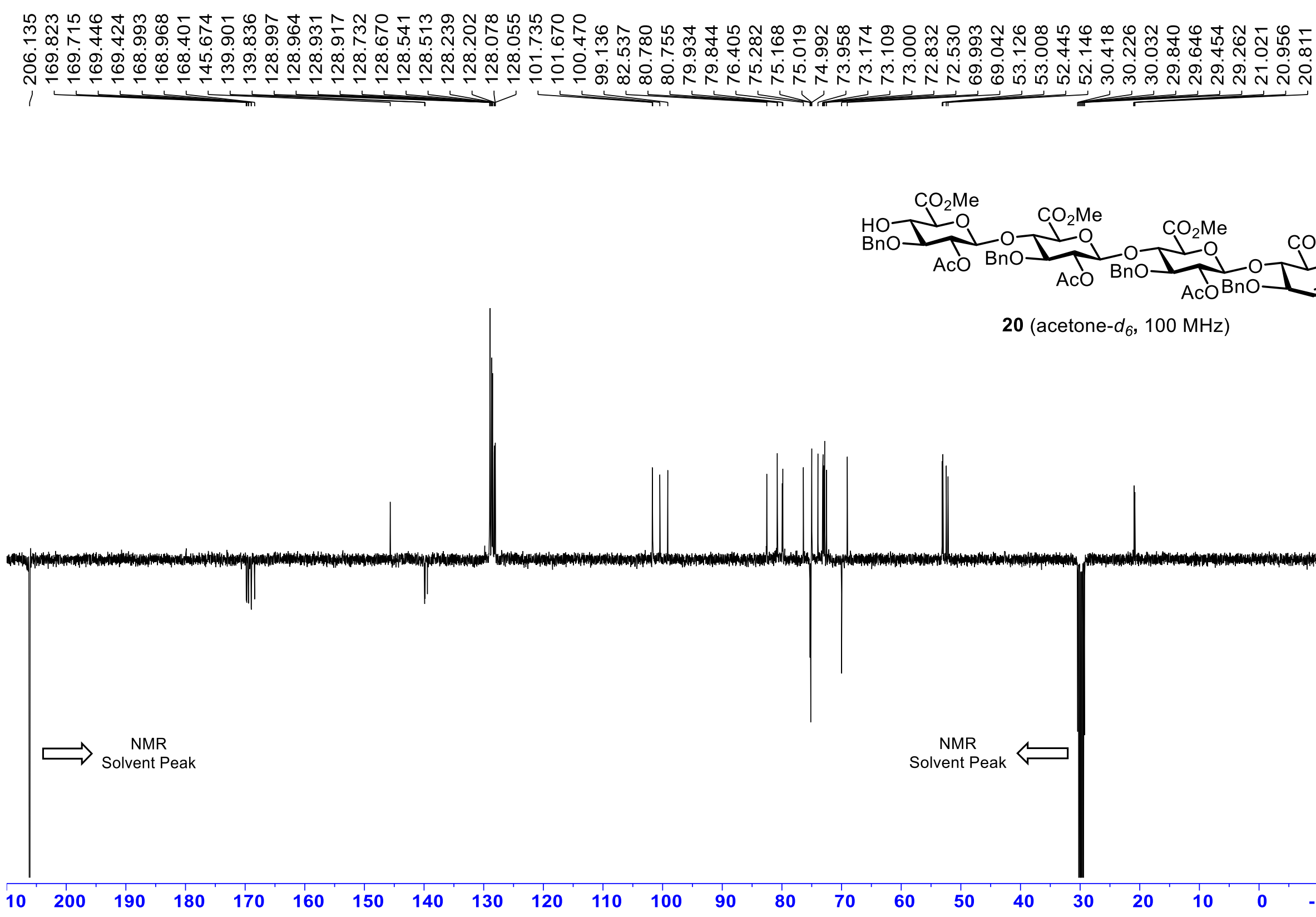

20 (acetone-d_6, 100 MHz)

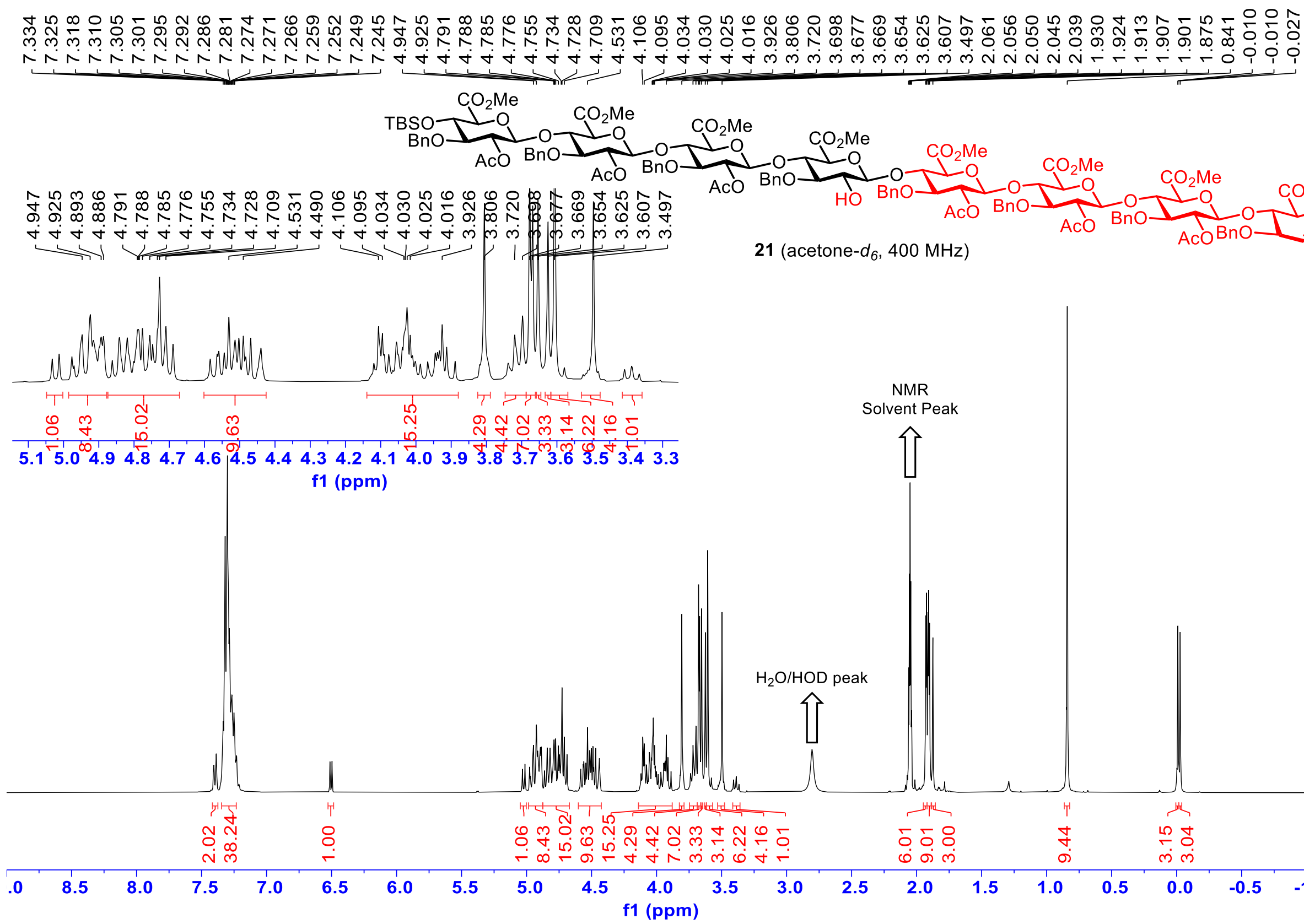
21 (acetone-d_6, 400 MHz)
NMR
Solvent Peak
H_2O/HOD peak
f1 (ppm)

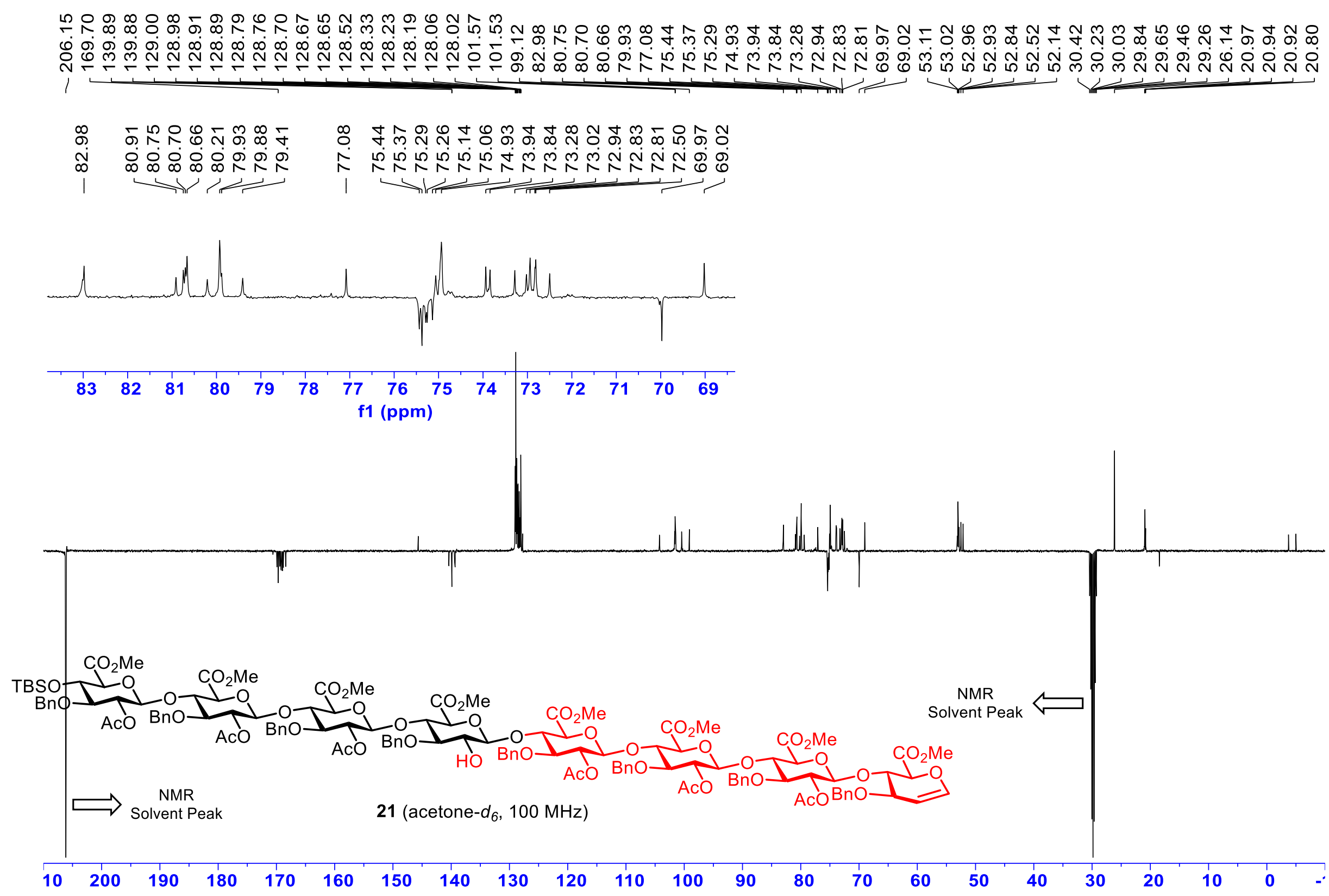
21 (acetone-d_6, 100 MHz)
NMR
Solvent Peak
f1 (ppm)

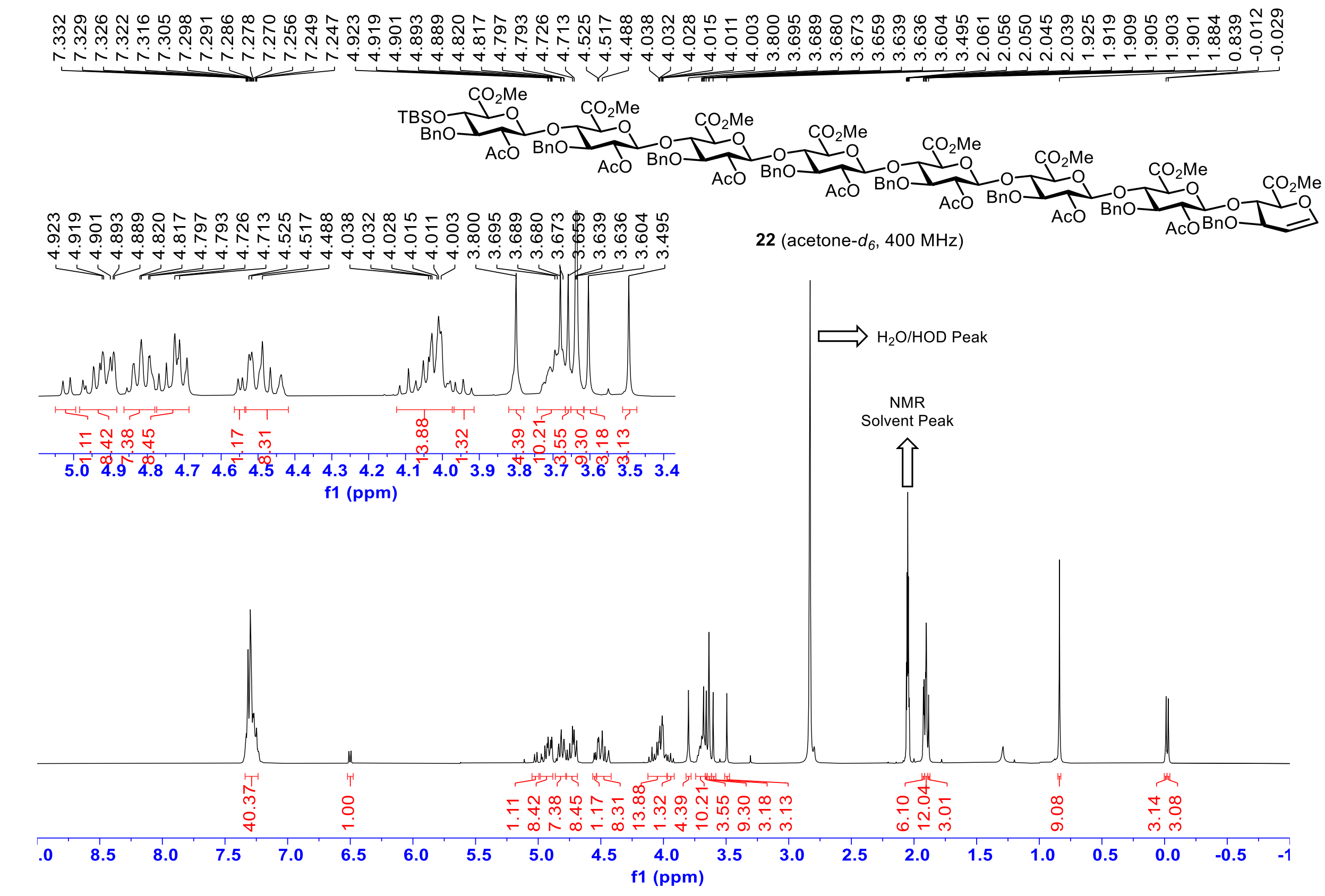
7.332 7.329 7.326 7.322 7.316 7.305 7.298 7.291 7.286 7.278 7.270 7.256 7.249 7.247 4.923 4.919 4.901 4.893 4.889 4.820 4.817 4.797 4.793 4.726 4.713 4.525 4.517 4.488 4.038 4.032 4.028 4.015 4.011 4.003 3.800 3.695 3.689 3.680 3.673 3.659 3.639 3.636 3.604 3.495 2.061 2.056 2.050 2.045 2.039 1.925 1.919 1.909 1.905 1.903 1.901 1.884 0.839 -0.012 -0.029
22 (acetone-d_6, 400 MHz)
H2O/HOD Peak
NMR Solvent Peak
40.37 1.00 1.11 8.42 7.38 8.45 1.17 8.31 13.88 1.32 4.39 10.21 3.55 9.30 3.18 3.13 6.10 12.04 3.01 9.08 3.14 3.08
f1 (ppm)

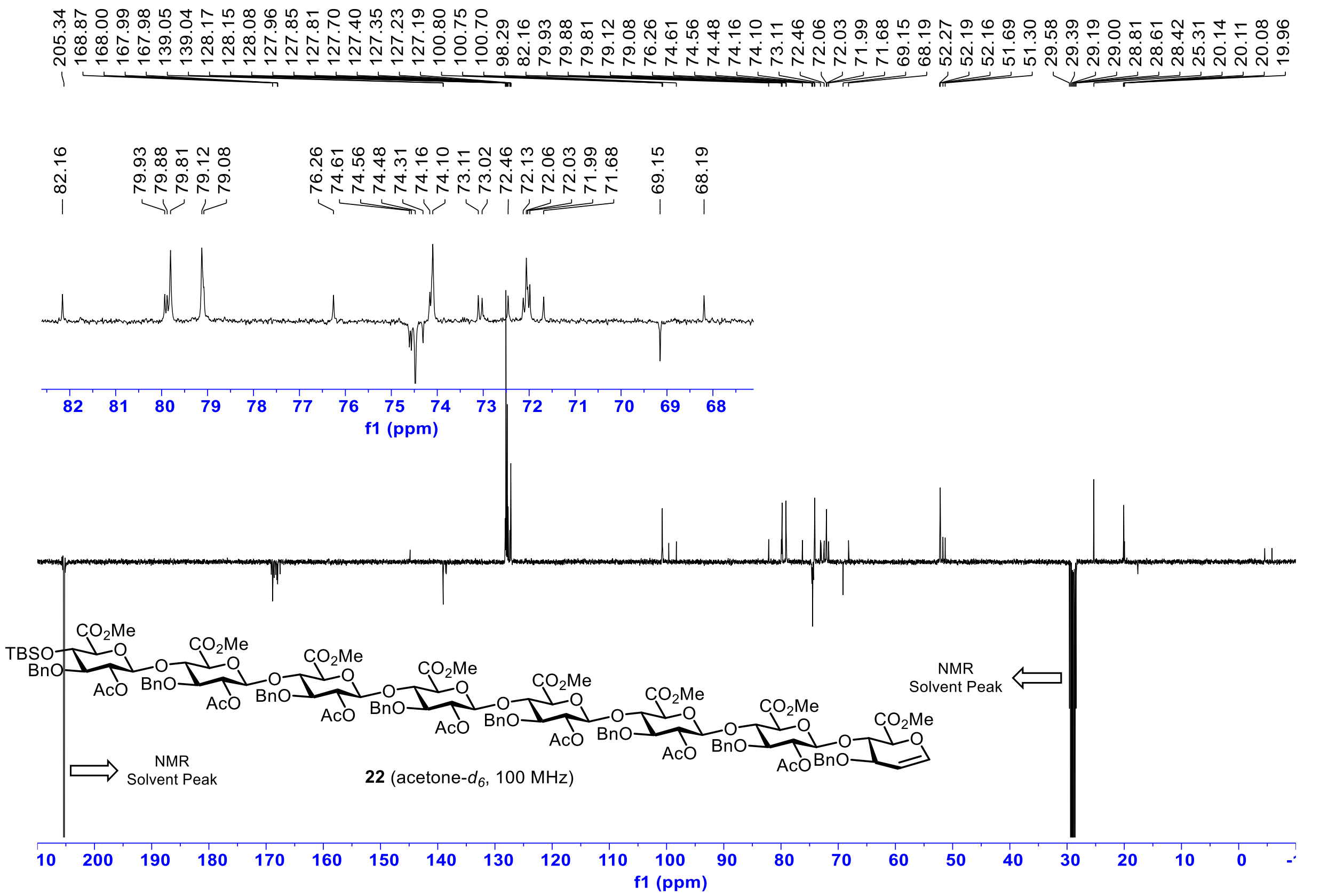
205.34 168.87 168.00 167.99 167.98 139.05 139.04 128.17 128.15 128.08 127.96 127.85 127.81 127.70 127.40 127.35 127.23 127.19 100.80 100.75 100.70 98.29 82.16 79.93 79.88 79.81 79.12 79.08 76.26 74.61 74.56 74.48 74.16 74.10 73.11 72.46 72.06 72.03 71.99 71.68 69.15 68.19 52.27 52.19 52.16 51.69 51.30 29.58 29.39 29.19 29.00 28.81 28.61 28.42 25.31 20.14 20.11 20.08 19.96
82.16 79.93 79.88 79.81 79.12 79.08 76.26 74.61 74.56 74.48 74.31 74.16 74.10 73.11 73.02 72.46 72.13 72.06 72.03 71.99 71.68 69.15 68.19
NMR Solvent Peak
22 (acetone-d_6, 100 MHz)
f1 (ppm)

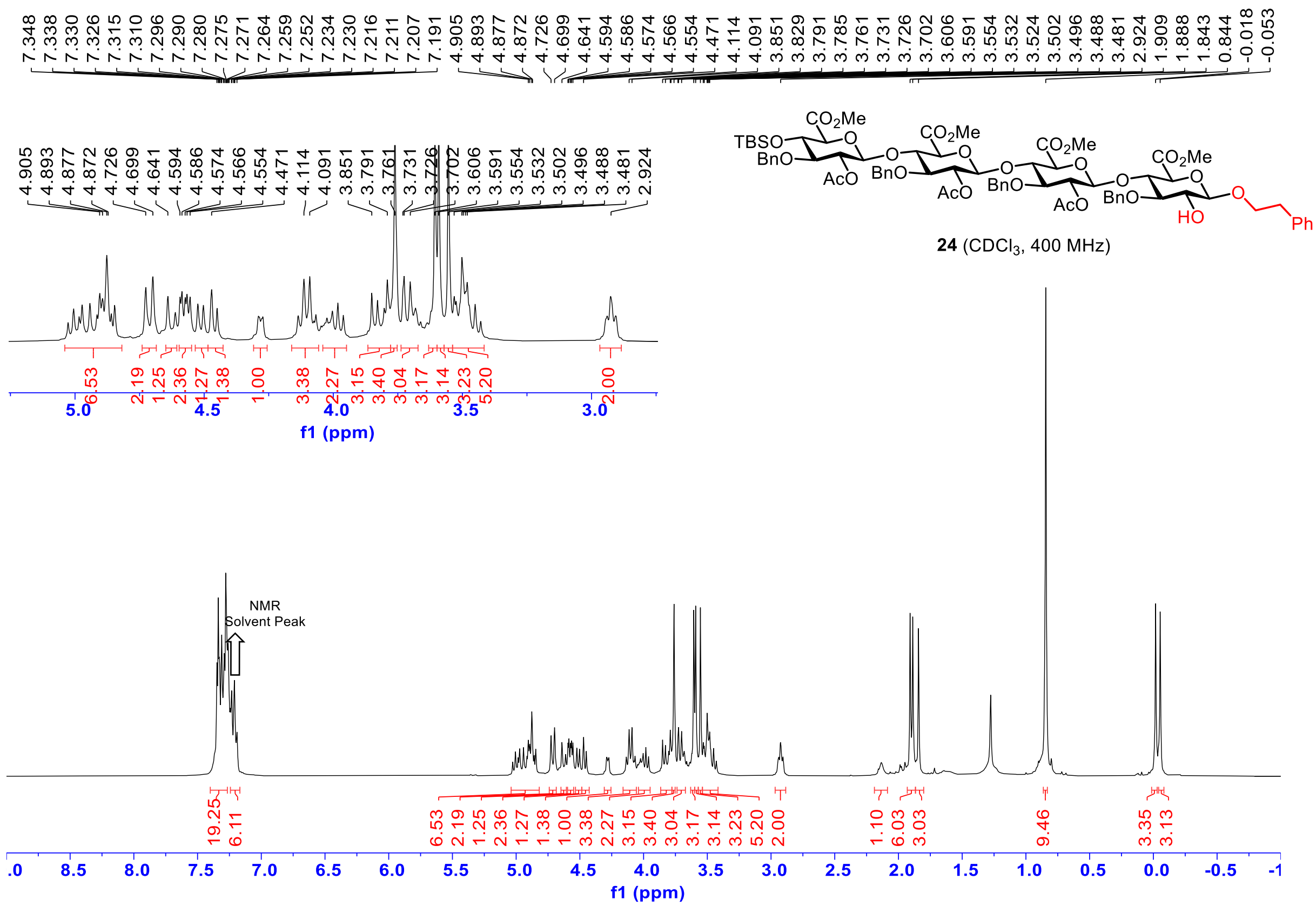

24 ($CDCl_3$, 400 MHz)

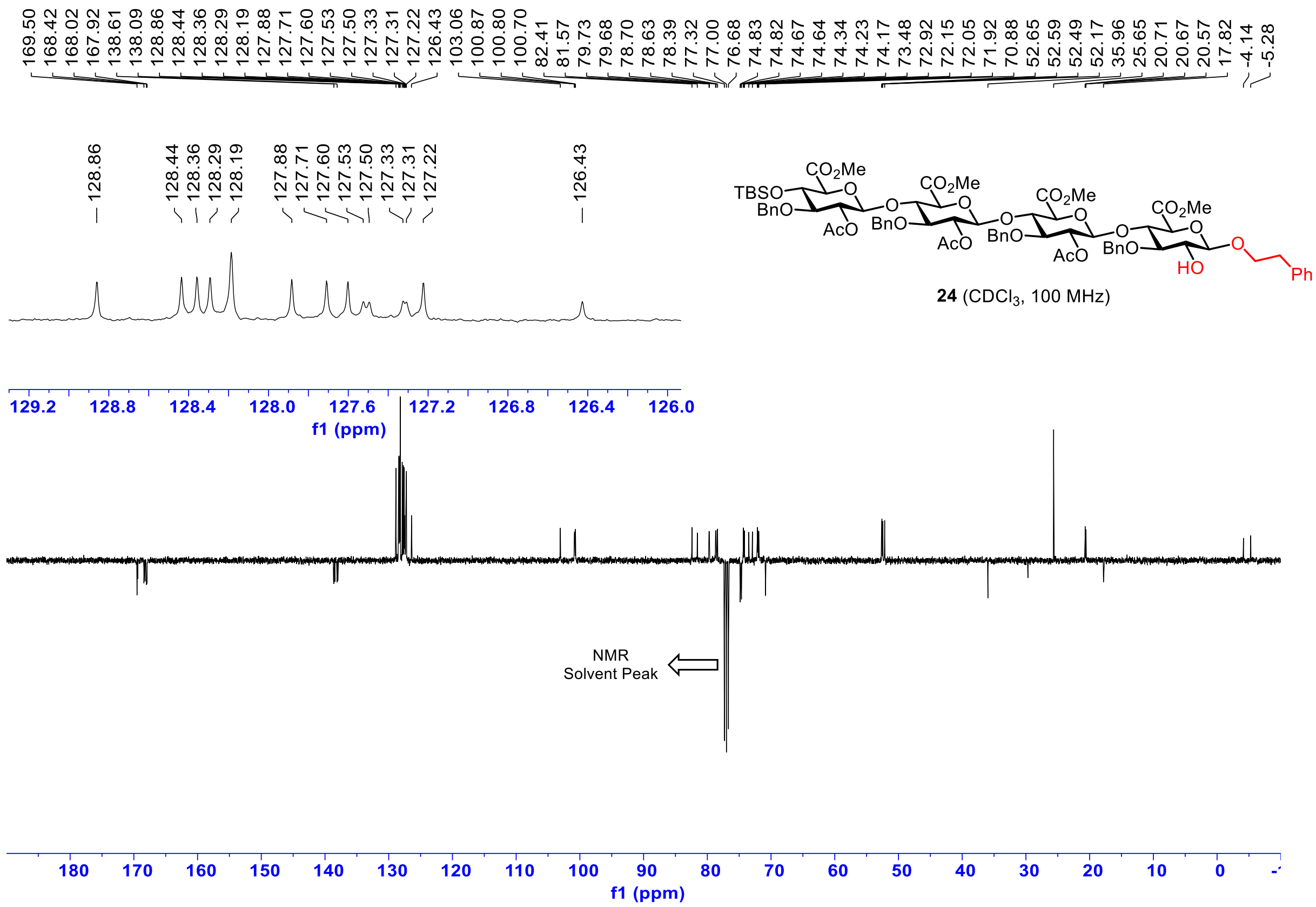

24 ($CDCl_3$, 100 MHz)

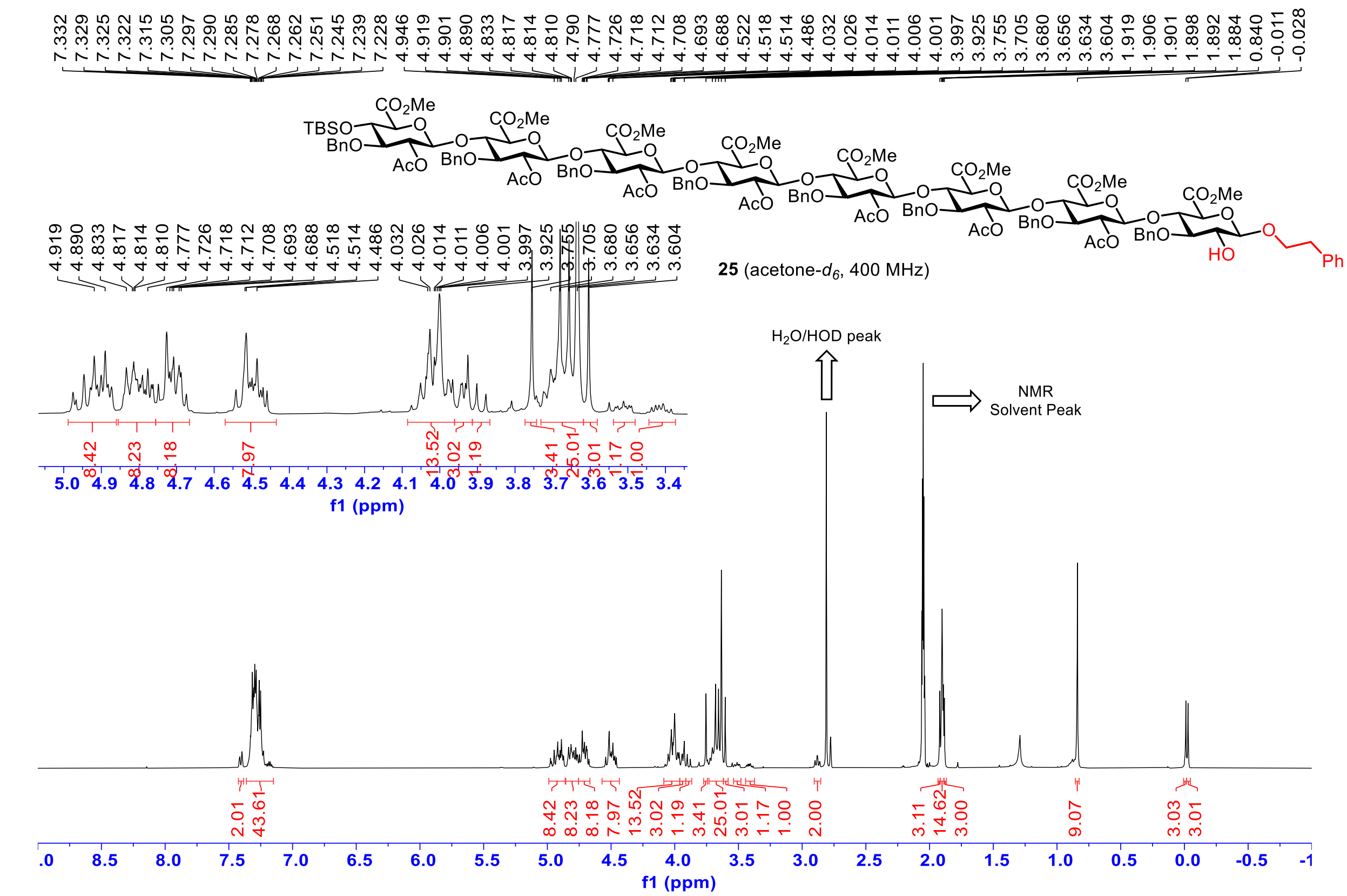

25 (acetone-d_6, 400 MHz)

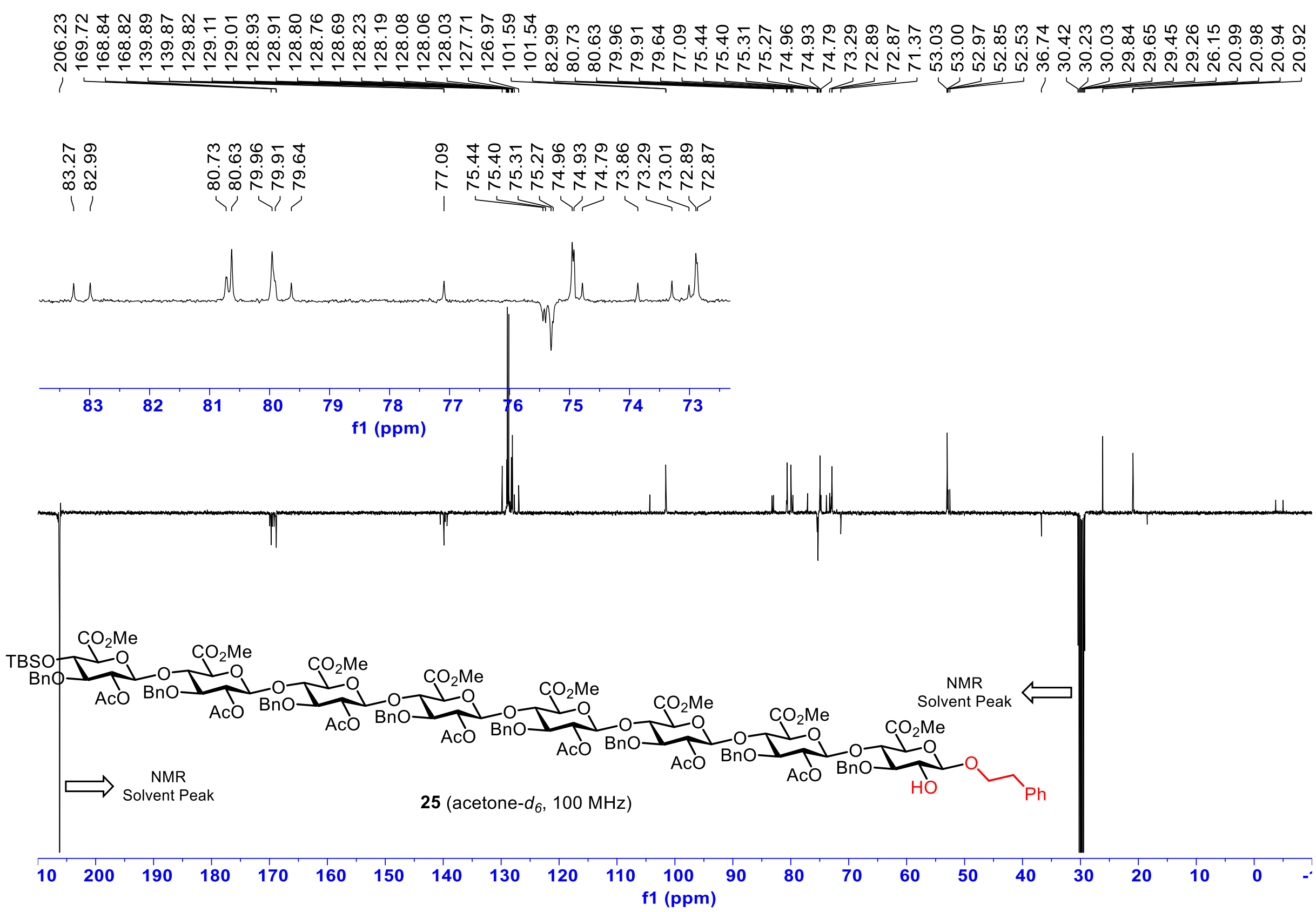

25 (acetone-d_6, 100 MHz)

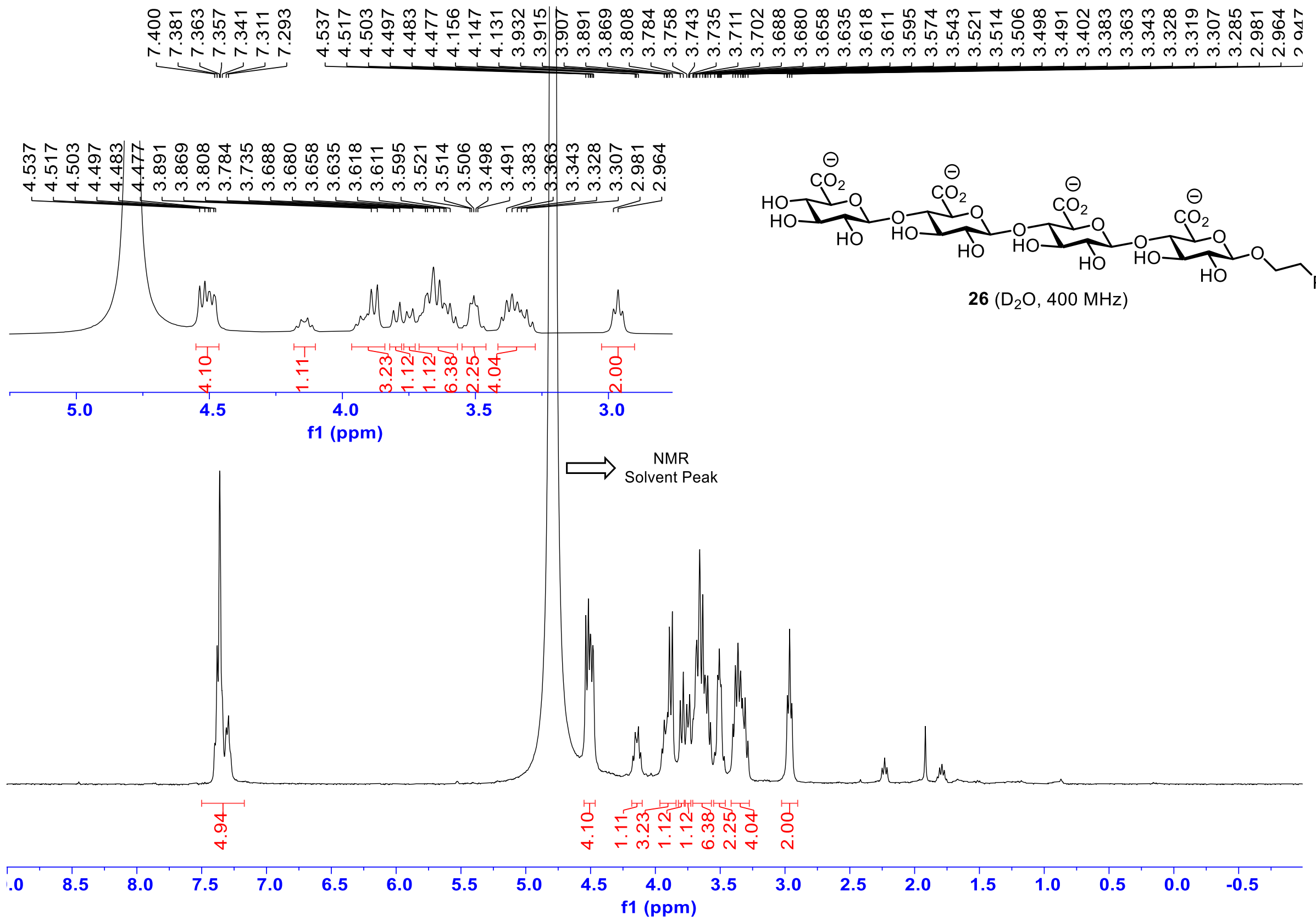

26 (D_2O, 400 MHz)

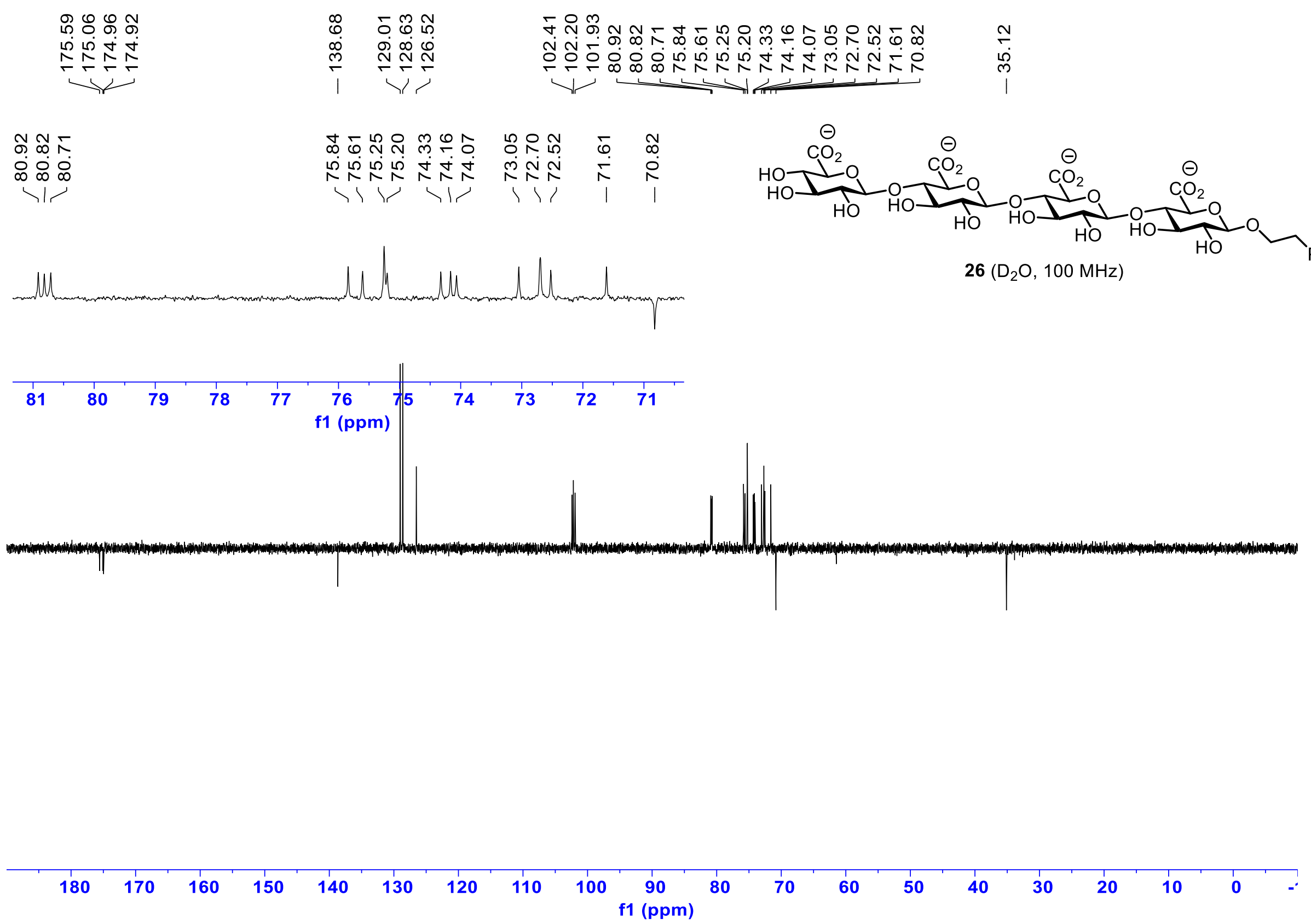

26 (D_2O, 100 MHz)

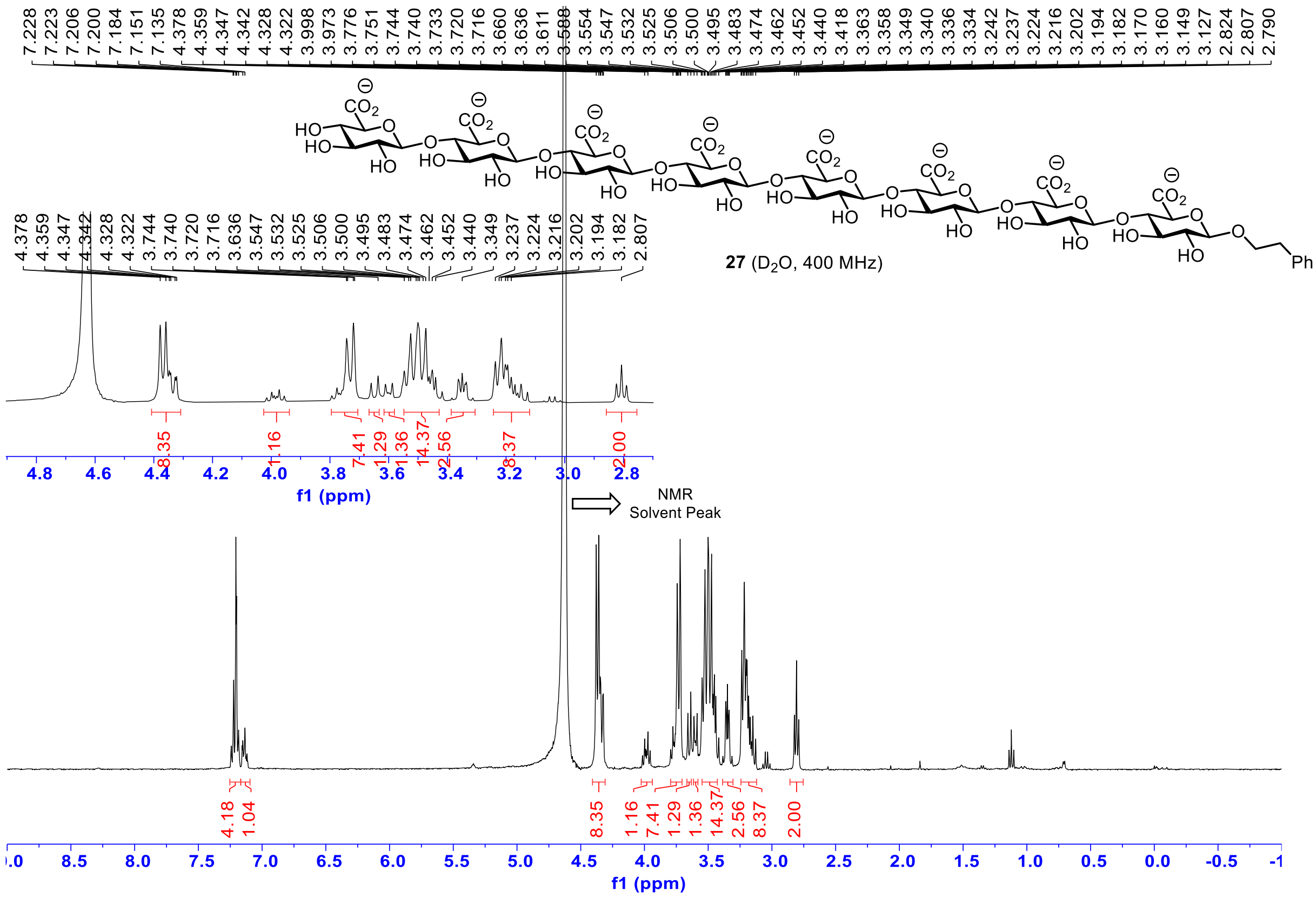

27 (D_2O, 400 MHz)

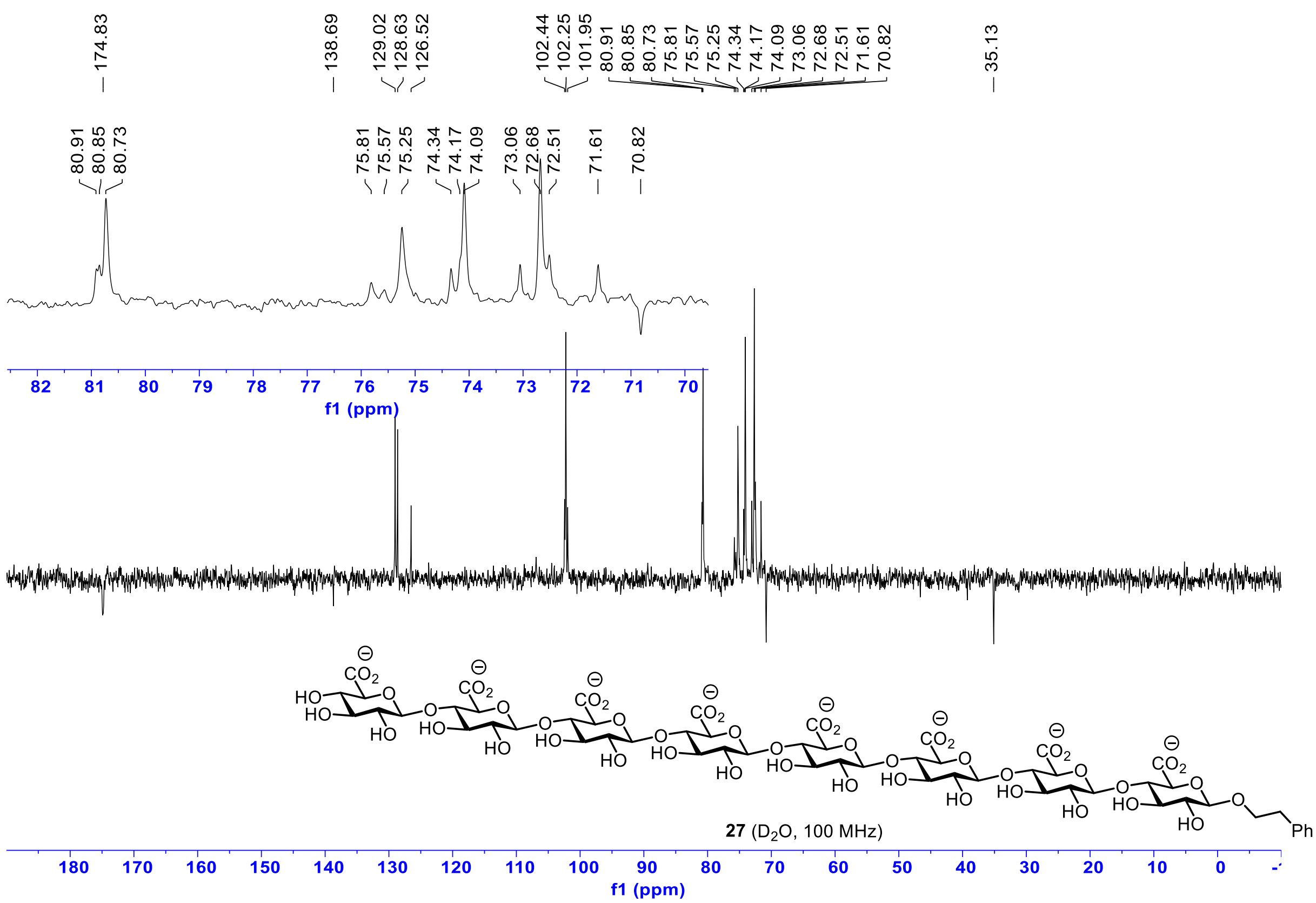

27 (D_2O, 100 MHz)
